# NCBP1 stress signaling drives alternative S6K1 splicing inhibiting translation

**DOI:** 10.1038/s41589-025-02135-4

**Published:** 2026-02-10

**Authors:** Dalu Chang, Mahdi Assari, Chananya Suwathep, Khomkrit Sappakhaw, Chayasith Uttamapinant, Marcus J. C. Long, Yimon Aye

**Affiliations:** 1 University of Oxford, Oxford, UK; 2 British Heart Foundation (BHF) Oxford Centre of Research Excellence (CRE), Oxford, UK; 3 University of Chicago, Chicago, IL USA; 4 Vidyasirimehdi Institute of Science and Technology (VISTEC), Rayong, Thailand; 5 University of Lausanne (UNIL), Epalinges, Switzerland

**Keywords:** Post-translational modifications, Synthetic biology

## Abstract

Subcellular stress profoundly influences protein synthesis. However, both the nature of spatiotemporally restricted chemical cues and local protein responders to these cues remain elusive. Unlocking these mechanisms requires the ability to functionally map in living systems locale-specific stress responder proteins and interrogate how chemical modification of each responder impacts proteome synthesis. We resolved this problem by integrating precision localized electrophile generation and genetic code expansion tools. Upon examination of four distinct subcellular locales, only nuclear-targeted electrophile stress stalled translation. We discovered that NCBP1—a nuclear-resident protein with multifaceted roles in eukaryotic mRNA biogenesis—propagated this nuclear stress signal through a single cysteine (C436) from among its 19 conserved cysteines. This NCBP1(C436)-specific modification elicited alternative splicing of more than 250 genes. Mechanistically, global protein synthesis stall was choreographed by impaired association between electrophile-modified NCBP1(C436) and SF3A1, an essential component of spliceosome, triggering the production of alternatively spliced S6 kinase, whose expression was sufficient to dominantly inhibit protein translation.

**Figure:**
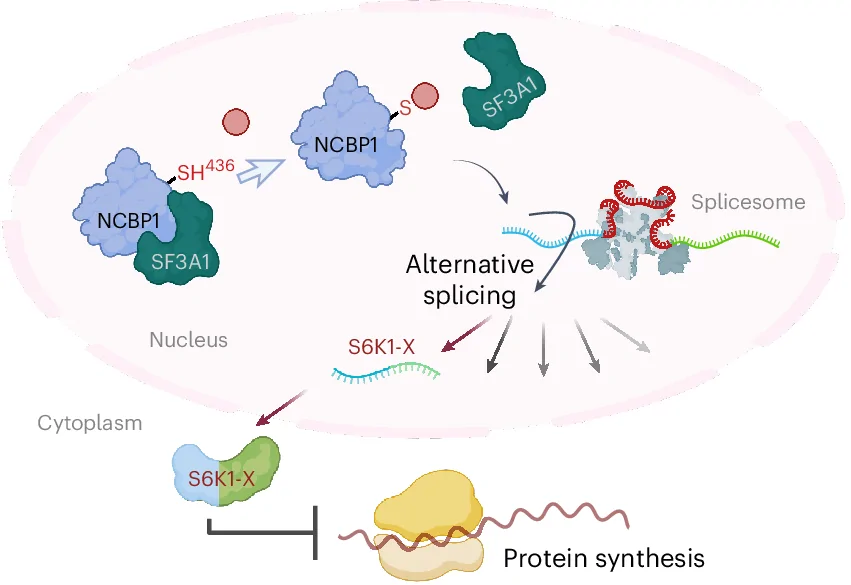

## Abstract (web summary)

Chang et al. found that nuclear reactive electrophile stress suppresses translation through HNEylation at C436 of NCBP1, triggering alternative splicing of S6K1, a key regulator of translation.

## Main

The canonical protein code contains 20 amino acids, equipping nascent proteins with a versatile array of building blocks. When positioned appropriately, these elements alone can perform tremendous feats, including proteolysis, sigmatropic shifts and redox relays. Combined with co-factors, more possibilities arise—for example, decarboxylation and proton-coupled electron transfer. Nonetheless, the pressures of selection have frequently forced nature to accessorize its canonical amino acid toolbox. These embellishments occur classically in the form of posttranslational modifications (PTMs)^1^ of specific residues that change chemical properties, fundamentally altering regulatory modes, and oftentimes function, of proteins.

Numerous efforts have been expended into decoding PTM signaling modes at the pathway and protein-specific levels, giving way to the ubiquitin and histone codes and our understanding of phosphosignaling networks. These signals implicate themselves—often in a synergistic manner—in almost all cellular processes. Such regulatory understanding has, in turn, had important ramifications for drug design and disease etiology. Indeed, these canonical PTMs are enacted by specific proteins, ‘writers’, that code these modification-specific signals. These messages are, in turn, interpreted by specific downstream proteins, ‘readers’. However, over the years, an alternative PTM-driven signaling paradigm has arisen, whereby signaling occurs when electrophilic small-molecule metabolites—without the involvement of specific writer proteins—covalently label specific proteins^2–4^.

A protein’s intrinsic electrophile reactivity and the signaling consequences of electrophile-driven PTMs are still mostly unpredictable. However, several pathways have emerged to be responsive to non-enzyme-assisted PTMs orchestrated by innate electrophilic metabolites, oftentimes resulting in dominant loss-of-function or gain-of-function signaling through modulation of a specific protein of interest (POI)’s function/activity. Examples include kinase isoform-specific signaling, transcription, antioxidant response and mitochondria-associated programmed cell death^5–7^. Nevertheless, despite attempts to decode these non-canonical PTM signaling events, scant paradigmatic examples remain. Locale-specific examples are particularly rare. We remain unaware of how specific localized reactive chemical signal inputs propagate along specific pathways involving defined mediators, leading to functional biological outputs. Accumulating evidence indicates that electrophile-responsive pathways are triggered even at low electrophile modification stoichiometry (hereafter ‘ligand occupancy’ (LO)) on a single electrophile responder protein. We also disclosed organ-specific electrophile sensitivity of an otherwise multiorgan-resident protein and how this tissue-specific electrophile responsivity regulates global stress management in live nematode worms^8^.

Among the limited instances of biological processes directly influenced by such locale-specific substoichiometric electrophile sensing and signaling, protein translation remains a largely unexplored arena. This is despite the emerging phenomenon surrounding dynamical changes in translation efficiency in response to localized subcellular stress^9,10^. In the present study, we used an unbiased proteome-wide screen to identify novel locale-specific electrophilic stress responders, in tandem with a genetic code expansion-based^11^ translation reporter platform. This approach identified the nuclear protein NCBP1 (CBC80) as a novel sensor of the native electrophilic lipid-derived metabolite 4-hydroxynonenal (HNE). Despite numerous cysteines within NCBP1 sensing HNE, only HNEylation through one specific site (C436) downregulated translation. Translational stall was choreographed by reduced interaction between HNEylated NCBP1 and an essential splicesome component (SF3A1). This event triggered splicing deregulation of numerous genes. One affected gene was S6K1, which formed a new alternatively spliced isoform, which we termed S6K1-X. S6K1-X was dominant negative for translation, explaining the majority of HNEylated NCBP1-induced translation inhibition and how NCBP1(C436)-specific HNEylation impaired translation. We thus provide one of the first examples of a specific protein−electrophile labeling event impacting translation. We further document an intricate molecular mechanism of translation regulation that proceeds through a gain-of-function splicing event. Interestingly, S6K1-X was selectively upregulated in cell-based Huntington’s disease models that also manifested increased HNEylated proteomes. These data overall display the vast richness of reactive small-molecule signaling and highlight the unpredictability of important non-canonical PTM signaling events.

## Results

### Nuclear electrophile release suppresses protein synthesis

**Fig. 1:**
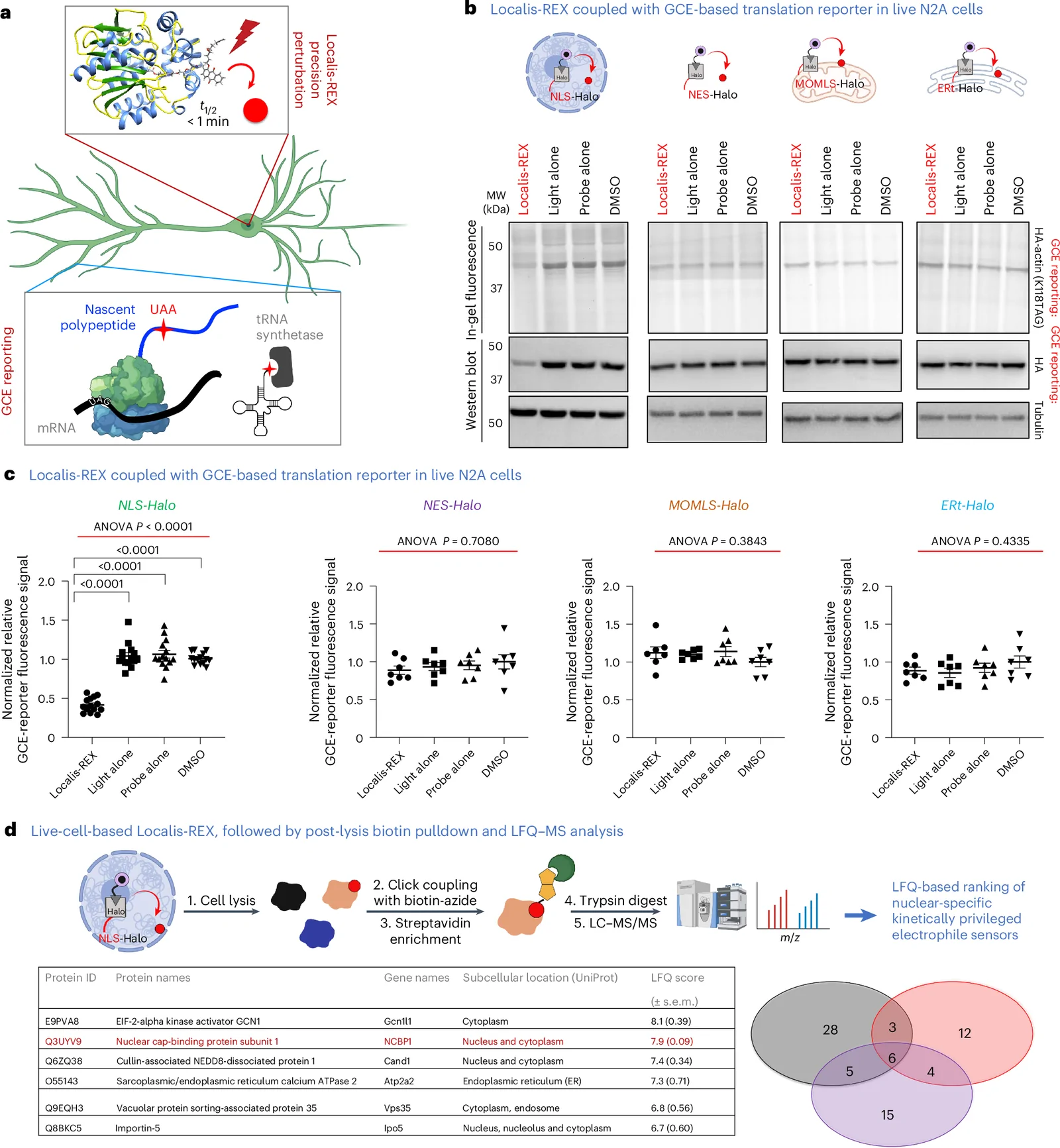
Global protein synthesis is inhibited by nuclear-targeted electrophile delivery. **a**, Schematic of Localis-REX−GCER. Top box (see also Supplementary Fig. 1a): the small-molecule electrophilic stressor (red sphere) is liberated rapidly (*t*_1/2_ < 1 minute) upon illumination (5 mW cm^−^^2^, 365 nm) from the photocaged electrophile covalently bound to locale-restricted Halo. Electrophiles released compete among the best electrophile sensors within Halo’s proximity, enabling mapping of kinetically privileged first responders with controled timing, chemotype, dosage and locale. Lower box: GCER^11,12^ assesses perturbation to protein synthesis (see also Supplementary Fig. 1a,b). **b**, Differentiated mouse neuroblasts (N2A cells) expressing Halo fused to different localization sequences—NLS, NES, MOMLS or ERt—were subjected to Localis-REX−GCER (see also Supplementary Tables 1 and 2 and Supplementary Fig. 1a–d). In-gel Cy5 fluorescence and anti-HA blot report expression levels of HA-actin(K118TAG). Anti-tubulin antibody serves as loading control (*n* = 7) (see also Extended Data Fig. 1 and Supplementary Fig. 2). **c**, Quantification of **b** and Supplementary Fig. 2. ‘Normalized relative GCE reporter signal’ denotes anti-HA signal normalized by anti-tubulin. *P* values were calculated by Tukey’s multiple comparisons test. Data show mean ± s.e.m. (*n* = 14 independent experiments for NLS-Halo, *n* = 7 for rest). **d**, Localis-REX coupled to LFQ-based proteomics (top row). The six top-ranked kinetically privileged nuclear-specific electrophile senor proteins (table, bottom left) from Localis-REX performed in differentiated mouse N2A cells ectopically expressing nuclear-restricted Halo (see also Supplementary Data 1). Venn diagram (bottom right) shows the number of statistically significant enriched hits from each replicate. A stringent threshold was set to limit hits to focus on proteins with highest probability of having phenotypic relevance (Extended Data Fig. 3); lowering the threshold could yield more hits. Note: the proteins identified are of mouse origin but are conserved in humans (Supplementary Figs. 6–8 and 10). LC−MS/MS, liquid chromatography−tandem mass spectrometry. Source data

To cast a new lens over how localized small-molecule stressors reprogram translation, we set out to unite genetic code expansion-based translation reporting (GCER)^11,12^ with our recently developed function-guided proximity mapping (Localis-REX)^6^ (Fig. 1a). Localis-REX deploys the self-labeling protein Halo as a permanent anchor for a photocaged electrophile—in this instance, 4-HNE. The photocaged electrophile (hereafter ‘Ht-PreHNE’ (Supplementary Fig. 1d)) releases native HNE for experiments studying signaling, or an alkyne-functionalized analog, HNE(alkyne), to enrich HNEylated proteins or validate labeling. The REX setup thus enables generation of HNE in a specific locale (organ/cell type, subcellular locale, etc.) in live cells/animals with high spatiotemporal resolution (Supplementary Fig. 1). After Halo expression at a specific locale (Supplementary Fig. 1a), treatment with Ht-PreHNE(alkyne), washout of excess unbound probe and light exposure liberates HNE(alkyne) (*t*_1/2_^photouncaging^ < 1 minute) in Halo’s vicinity and, hence, at markedly elevated concentrations within this locale. After lysis, enrichment of HNE(alkyne)ylated native proteins enables target ID of local electrophile responder proteins. Conversely, GCER measures dynamic changes in translation (Supplementary Fig. 1b) in response to electrophile buildup in a defined locale. Comparing effects across Localis-REX-assisted HNE buildup in different locales furnishes precision information about HNE’s locale-specific effects on translation.

**Extended Data Fig. 1:**
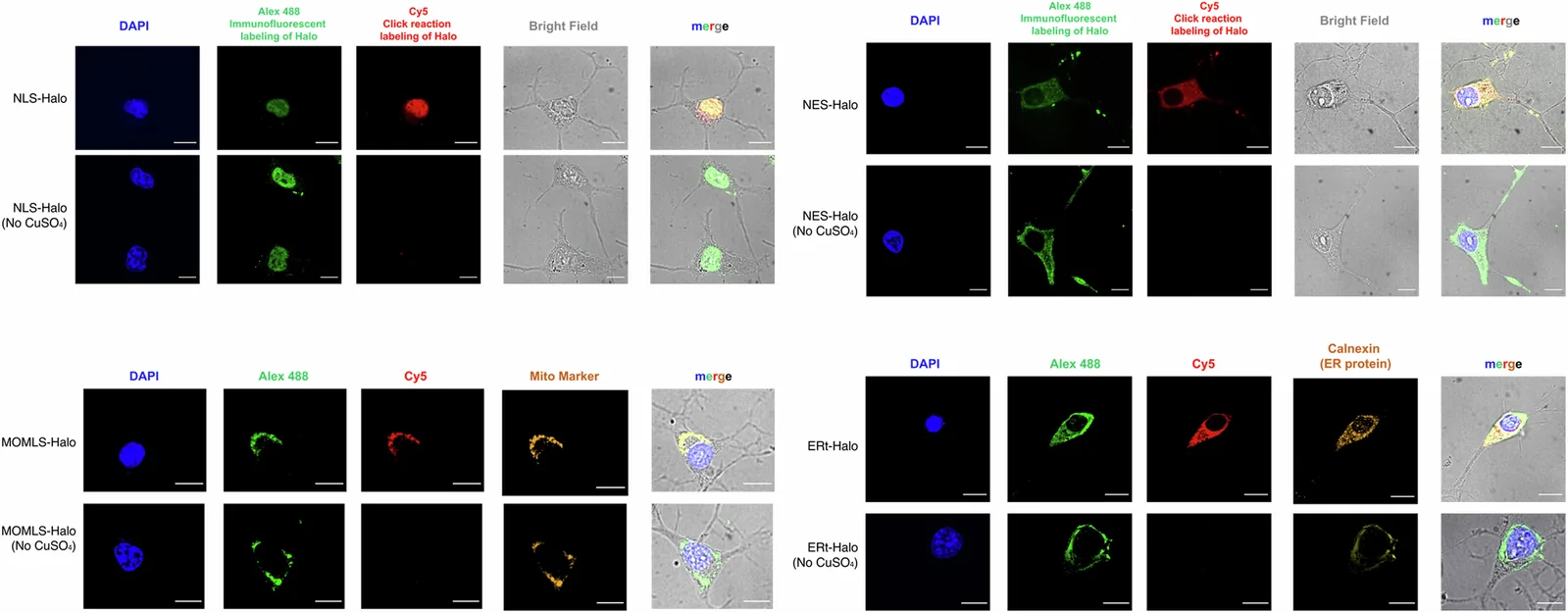
Immunofluorescence imaging validates expected localization of Ht-PreHNE(alkyne) covalently bound to Halo expressed at designated subcellular locales. Differentiated N2A cells ectopically expressing either NLS-Halo, NELS-Halo, MOMLS-Halo, or ERt-Halo were incubated with Ht-PreHNE(alkyne) (10 µM) for 2 h, followed by rinsing, permeabilization and fixing. Localization of Ht-PreHNE(alkyne)-bound Halo was analyzed by Click coupling to Cy5-azide, using anti-Halo antibody and anti-mouse Alex 488 as primary and second antibodies, respectively. DAPI and MitoTracker Red (CaM7512, Thermo Fisher) were used as a nuclear and mitochondria marker, respectively. ER-resident Calnexin (sc-23954, Santa Cruz) was used as an ER marker. Conditions without Cu (hence, no Click coupling) were used as control for background labeling with Cy5-azide. Imaging was performed using a Nikon spinning disk confocal microscope equipped with a 100X objective. Scale bar, 10 µm.

To probe effects in different locales, we fused Halo to four distinct localization sequences—nuclear localization signal (NLS), nuclear export signal (NES), mitochondrial outer membrane localization sequence (MOMLS) and endoplasmic reticulum targeting sequence (ERt) (Supplementary Tables 1 and 2)—directing Halo (hence, localized HNE upregulation), respectively, to the nucleus, cytoplasm, mitochondrion outer membrane or endoplasmic reticulum (Supplementary Fig. 1a). In differentiated mouse neuro 2A cells (hereafter ‘N2A cells’) (Supplementary Fig. 1c and Methods), after treatment with Ht-PreHNE and washout, correct localization of probe-bound Halo was observed in all four instances (Extended Data Fig. 1). The resultant locale-specific effects on global protein synthesis were assessed in N2A cells ectopically expressing established GCER components^11,12^ (Supplementary Fig. 1b,e).

**Extended Data Fig. 2:**
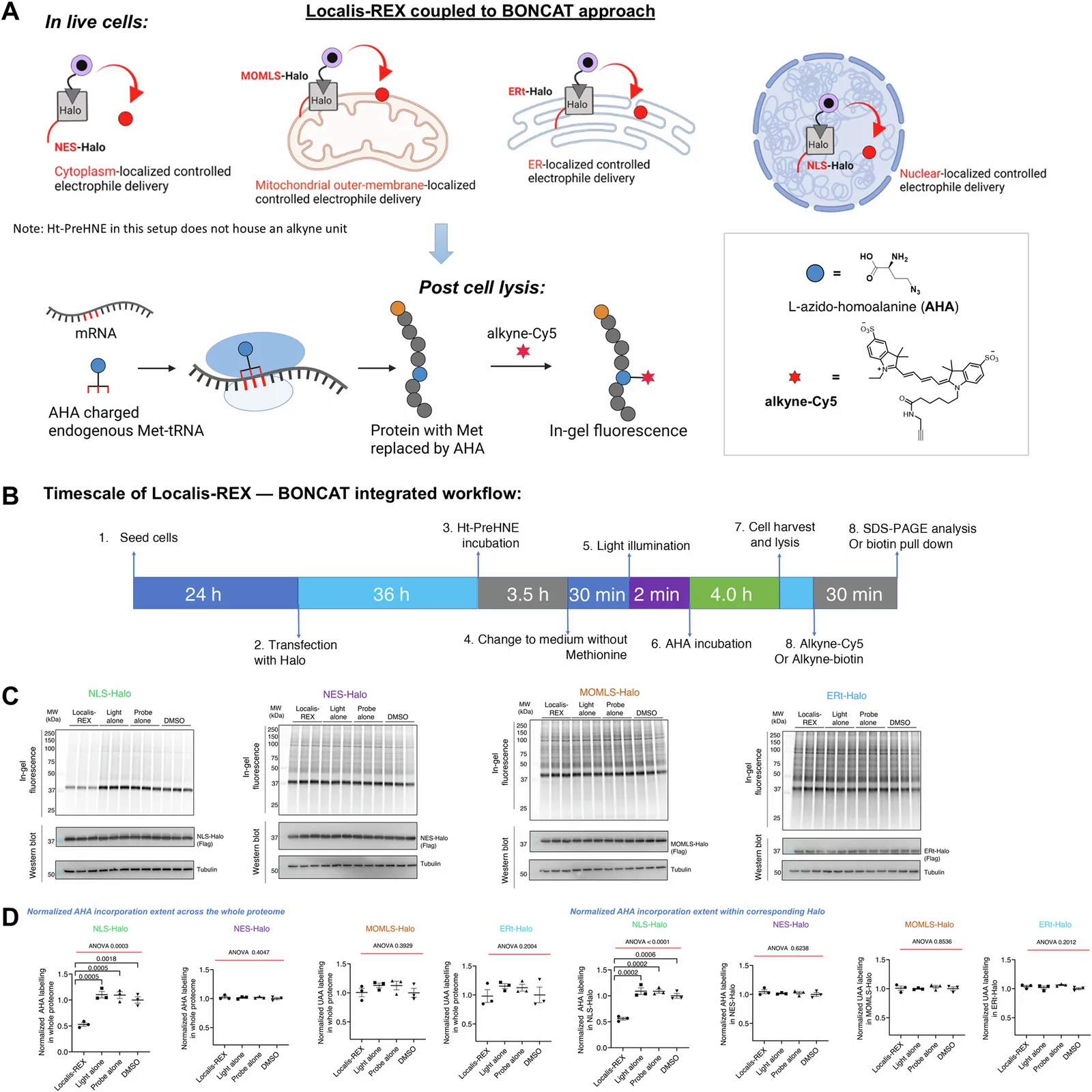
BONCAT experiment coupled with Localis-REX at different subcellular locales, shows reduction of nascent proteome synthesis in response to nuclear targeted electrophile delivery. **A**) and **B**) Schematic diagram and workflow of Localis-REX coupled to BONCAT assay. **C**) Localis-REX was performed similarly to Supplementary Fig. 2, except that the cells were incubated in media without Methionine for 30 min prior to light illumination (2 min), triggering localised electrophile generation. The cells were next subjected to media containing L-Azido-homoalanine (AHA, 50 µM, 4 h), to allow AHA incorporation into nascent proteome (see Methods). Post cell lysis, Cy5-Click coupling allowed quantification of AHA incorporation, correlating to translation efficiency. **D**) ‘Normalized AHA labelling in whole proteome’ (y-axis for plots in upper panel) corresponds to relative values derived from the in-gel fluorescence signal of Cy5, normalized by that of anti-tubulin; ‘Normalized AHA labelling in corresponding Halo’ (Y-axis for plots in lower panel) corresponds to relative values derived from the in-gel fluorescence signal of Cy5 under corresponding halo band, normalized by that of anti-tubulin; *p* values were calculated with Tukey’s multiple comparisons test. All data present mean ± SEM (*n* = 3). Source data

Initially, we used site-specific incorporation of a bicyclononyne-lysine (BCNK)^12^ into synthetic HA-tagged actin(K118TAG), by amber suppression, to assay translation efficiency (Supplementary Fig. 1b). Using in-gel fluorescence analysis, tracking the Cy5 signal arising from actin(K118)-incorporated BCNK (after Diels−Alder coupling in cell lysate) or western blot measuring anti-HA signal corresponding to full-length (FL) HA-actin (45 kDa), protein synthesis was prominently affected only when electrophile release occurred in the nucleus. No effect was observed when HNE was released in other locales (Fig. 1b,c and Supplementary Fig. 2). An orthogonal whole proteome labeling-based GCER approach^11–13^ validated the same outcome (Supplementary Figs. 3 and 4). Independently, bioorthogonal non-canonical amino acid (azidohomoalanine (AHA)) tagging (BONCAT)^14^ also gave the same results (Extended Data Fig. 2): AHA incorporation reporting translation efficiency for both the whole proteome and Halo itself was reduced after nuclear HNE buildup but not other locales (Extended Data Fig. 2). Thus, three independent readouts indicated that nuclear HNE stress inhibits translation (Supplementary Fig. 5). The fact that release of similar amounts of HNE in three other locales did not cause translation inhibition agrees with our previous data that HNE diffusion between cytosol and nucleus is minimal^6^ and further underscores a nucleus-specific effect. This short diffusion distance of HNE is likely due to cellular detoxifying enzymes that restrict HNE diffusion^4,15^.

### NCBP1 electrophile signaling stalls translation

**Extended Data Fig. 3:**
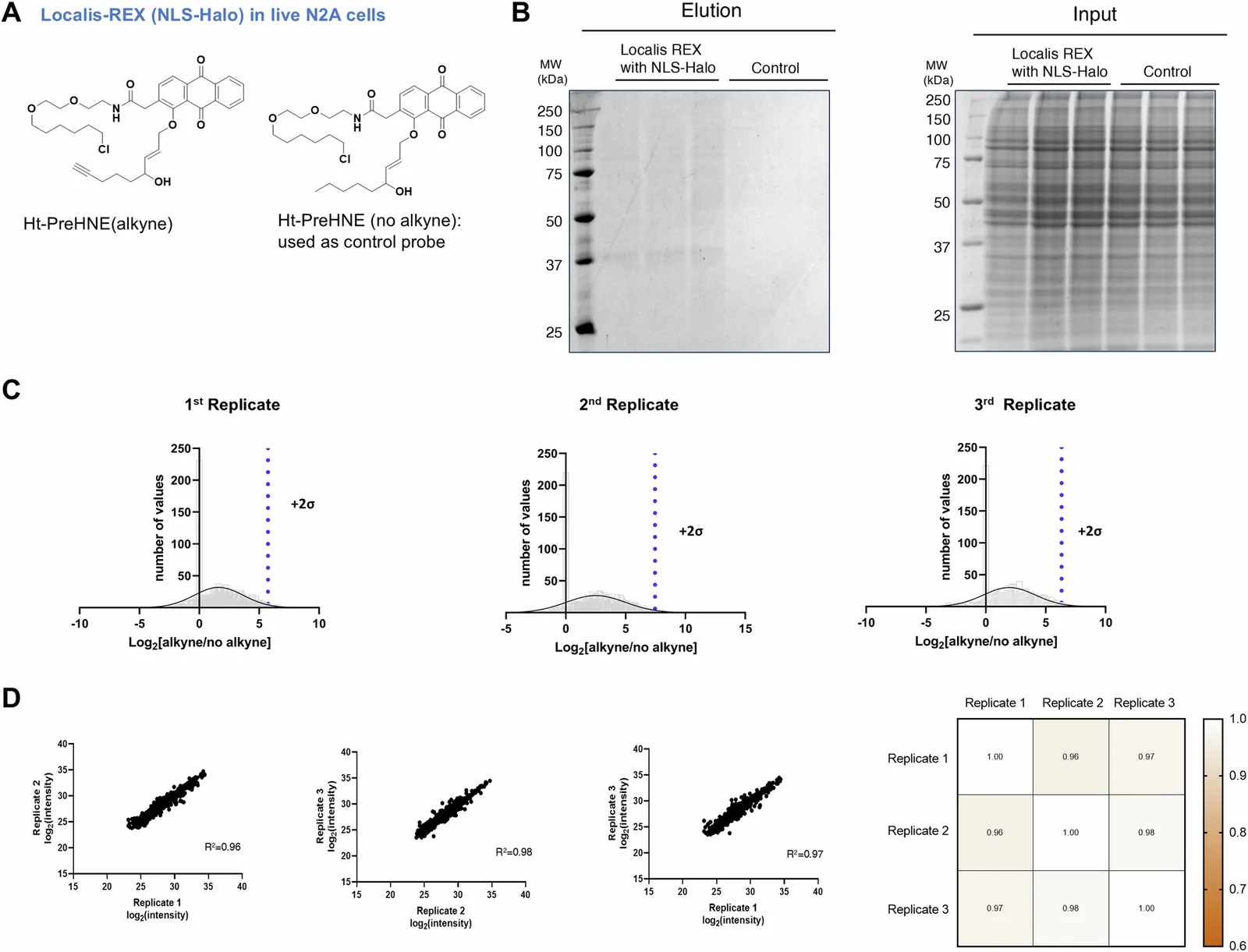
Localis-REX coupled with Click-biotin pulldown and LFQ-based quantitative proteomics, identifies nuclear-specific kinetically-privileged electrophile-sensor proteins. See also Fig. 1d and Supplementary Data 1. **A**) Chemical structures of REX-probes used in the assay. **B**) Representative datasets. Briefly, post Localis-REX execution in differentiated N2A cells expressing NLS-Halo, cells were lysed and subjected to Click-biotin-pulldown (see Methods). Both elution and input samples were resolved by SDS-PAGE and stained with colloidal Coomassie to detect proteins. ‘Control’ indicates an identical experiment except that no-alkyne-functionalized version of REX-probe was used. **C**) Results from 3 independent biological replicates stemming from Localis-REX coupled to differential enrichment-based proteomics target-ID involving label-free quantitation (LFQ) (see Supplementary Data 1), were subjected to Gaussian fit analysis examining LFQ ratios distribution as indicated. The x-axis indicates log_2_ [alkyne: no-alkyne control] of each hit detected in each replicate, and the y-axis indicates the number of proteins. Proteins of log_2_ [alkyne: no-alkyne control] more than two σ of Gaussian filter were considered as significant hits in each replicate. ‘alkyne’ and ‘no-alkyne control’ indicate the two REX-probes used for differential enrichment proteomics workflow. **D**) Correlation plots of the proteomics datasets: comparisons were performed between indicated independent replicates, with the resultant R^2^ greater than or equal to 0.96 in all cases. Heat map representation of R^2^ was shown on the *left*. Source data

We proposed that (a) specific nuclear protein(s) is(are) responsible for propagating this nucleus-specific signal to drive translation downregulation. Localis-REX function-guided proximity mapping generating HNE(alkyne) (Supplementary Fig. 1a), coupled with enrichment proteomics, can quantitively index endogenous locale-specific HNE responder proteins^6^. In nuclear-restricted Halo-expressing N2A cells, after Localis-REX-assisted HNE(alkyne) upregulation and lysis (Supplementary Fig. 1d), HNE(alkyne)ylated proteins were biotin modified via Cu(I)-catalyzed click coupling with biotin azide and enriched using streptavidin. After elution, HNE(alkyne)-modified nuclear first responder proteins were identified using label-free quantification (LFQ)-based proteomics target ID (Fig. 1d). We note that this workflow is different from some of our previously published protocols^5–7,16–20^ in which we compared responsivity of nuclear and cytosolic proteins^6^. This difference—and the fact that the cell line used in this instance was different from our previous reports—likely explains why our previously reported hits were not discovered. Localis-REX function-guided mapping revealed six protein hits (Fig. 1d, Extended Data Fig. 3 and Supplementary Data 1). We note that previous global mapping of HNE-responsive proteins using state-of-the-art isotopic tandem orthogonal proteolysis−activity-based protein profiling (isoTOP-ABPP) uncovered a similar number of hits, albeit from lysates in non-organelle-specific contexts^21^.

Among these six proteins, nuclear cap-binding protein subunit 1 (NCBP1), Cand1 and IPO5 feature the nucleus as one of their resident subcellular components (Fig. 1d) based on UniProt annotations, but the nucleus is not an annotated locale for Gcn1l1, Atp2a2 and Vps35. Nonetheless, Atp2a2 and Vps35 are endoplasmic reticulum and endosome associated. Noting also that the endoplasmic reticulum is in continuity with the nucleus^22^ and endosomes can associate with the nuclear envelope^23^, these proteins may be ‘minority’ privileged electrophile responders at locales distinct from canonical environs—a phenomenon recently unmasked by Localis-REX^6,24,25^. We chose to focus on NCBP1, Cand1 and IPO5 where the UniProt database clearly indicates nuclear pools. Because human and mouse POIs were highly homologous for all three proteins (Supplementary Figs. 6–8), we focused on human POIs.

**Fig. 2:**
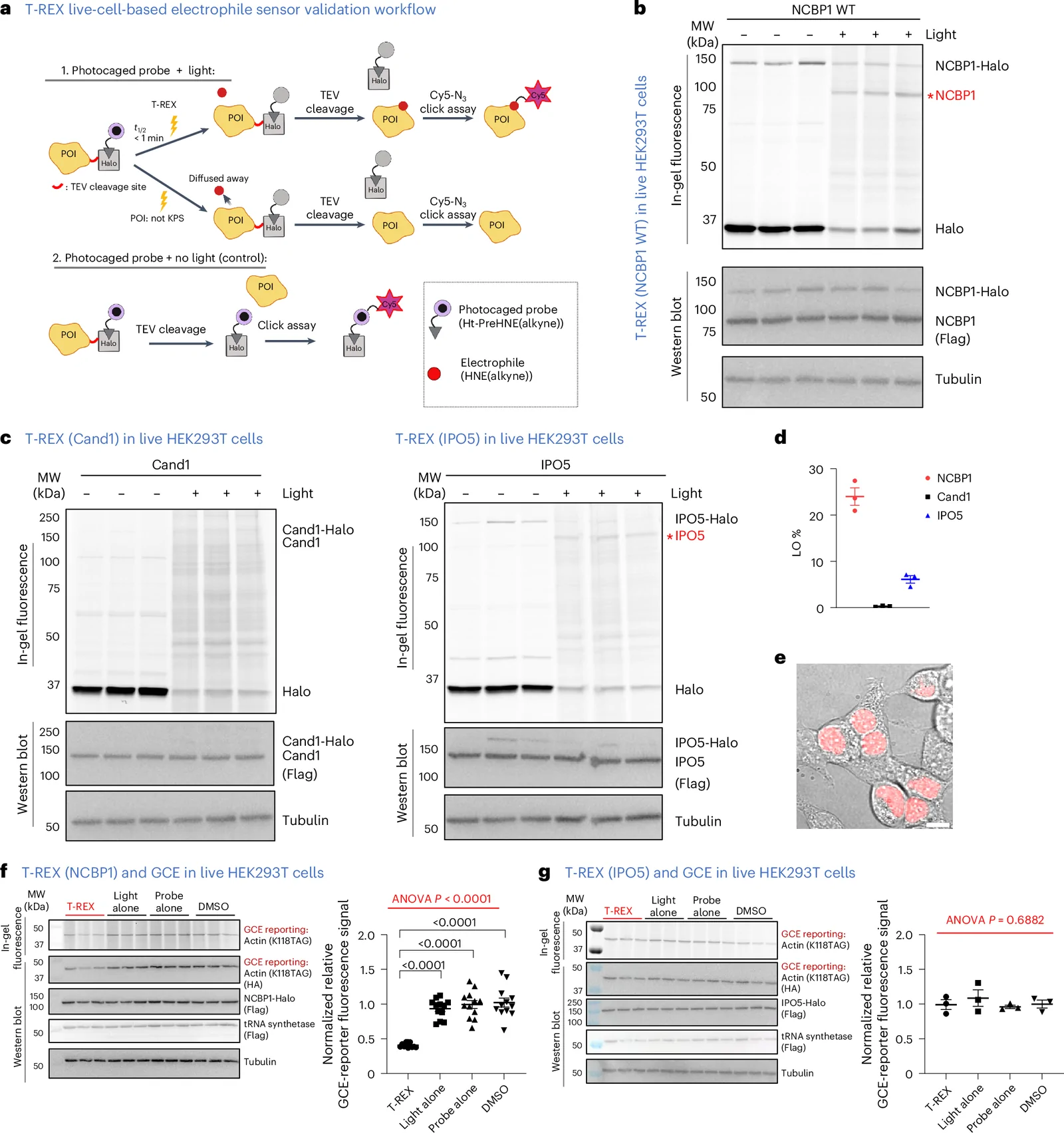
NCBP1 is a kinetically privileged electrophile sensor whose HNEylation downregulates translation. **a**, T-REX^16,17^ workflow: cells expressing Halo-fused POIs are treated with photocaged-(alkyne-functionalized)HNE. After washout, light triggers HNE-photouncaging (*t*_1/2_ < 1 minute). Providing that POI is a HNE(alkyne) kinetically privileged sensor (KPS), it is HNEylated prior to HNE diffusion. Unreacted HNE is captured by glutathione (5–10 mM) or enzymatically degraded^4,15^ but also elevates background by labeling other proteins. HNEylation can be quantified by in-gel fluorescence after cell lysis and Cy5-azide-click coupling (Methods and Supplementary Fig. 1d). Extended Data Fig. 4b,c shows a higher sensitivity readout. **b**, T-REX in HEK293T cells expressing NCBP1−Flag−TEV−Halo (‘NCBP1-Halo’) (Fig. 2a) validated that NCBP1 is a KPS. Samples not exposed to light but treated, after lysis, with TeV protease that separates NCBP1 and Halo are negative controls and define maximum HNE signal. Asterisk (*) indicates NCBP1 after TeV cleavage (92 kDa). **c**, Similar to **b** on IPO5 (124 kDa, after TeV cleavage) and Cand1 (136 kDa, after TeV cleavage). **d**, Quantification of HNE-LO after T-REX on the indicated POIs, from **b** and **c** (ref. ^18^). LO = (HNE(alkyne) signal on POI band, after TeV cleavage, under T-REX conditions) / (Ht-PreHNE(alkyne) signal on Halo without light exposure). Data present mean ± s.e.m. (*n* = 3). **e**, NCBP1−Flag−TeV−Halo localized to the nucleus in HEK293T cells treated with Halo-targetable TMR fluorescent dye. Scale bar, 10 µm. *n* = 3 (Extended Data Figs. 4e and 6a). **f**, HEK293T cells were co-transfected with NCBP1−Flag−TEV−Halo, HA-actin(K118TAG) and tRNA synthetase MmPylRS (Methods and Supplementary Fig. 1e). T-REX was performed, followed by BCNK incubation (50 µM, 4 hours). Cell lysates were incubated with tetrazine-Cy5 (1 µM, 30 minutes). In-gel Cy5 fluorescence and anti-HA blot showed translation suppression after NCBP1-HNEylation but not IPO5-HNEylation. *P* values were calculated with Tukey’s multiple comparisons test. Data present mean ± s.e.m. (*n* = 12). See Supplementary Fig. 9a for additional replicates. **g**, As in **f** except that IPO5−Flag−TeV−Halo was used (see also Extended Data Fig. 4c). Source data

**Extended Data Fig. 4:**
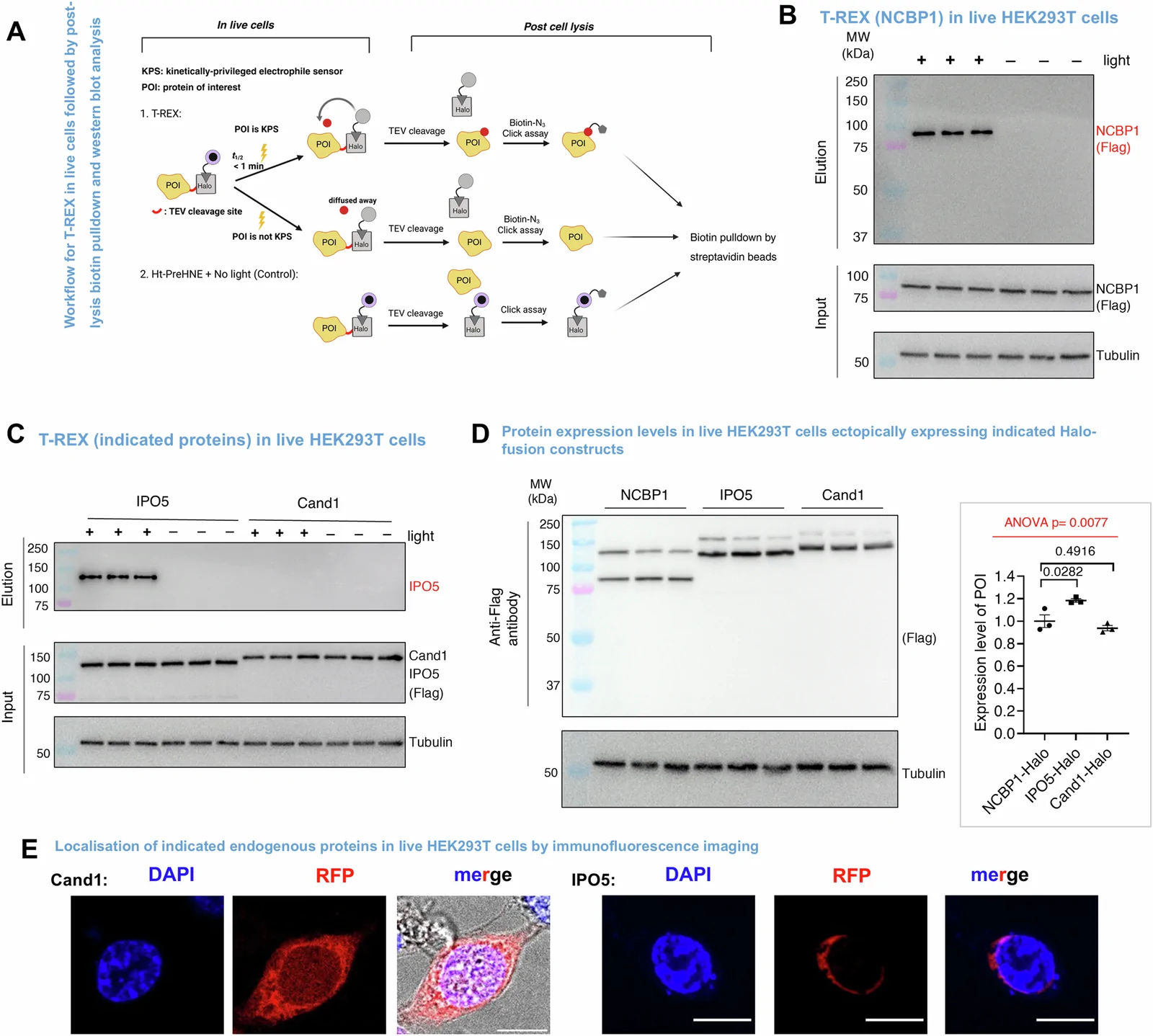
T-REX coupled with Click biotin pulldown shows only NCBP1 and IPO5, but not Cand1, manifest HNE responsivity following localised electrophile delivery. **A**) Schematic of T-REX coupled with Click biotin pulldown to validate electrophile-responsive proteins. **B**) and **C**) T-REX was performed in HEK293T expressing NCBP1-Flag(Tev-site)-Halo, IPO5-Flag(Tev-site)-Halo, or Cand1-Flag(Tev-site)-Halo (Methods). Post lysis, all samples (except Input samples), were subjected to TeV-cleavage [separating Halo and flag-tagged protein of interest (POI)]. All samples, including Input samples, were next subjected to Click biotin-azide coupling, and streptavidin-bead enrichment (Methods). Both Input and Elution samples were resolved by SDS-PAGE, blotted and analyzed using indicated antibodies. The band from Anti-Flag blot in the Elution represents HNE(alkyne)-modified POI (post TeV cleavage). This band was observed only in light-exposed samples. Conversely, the band in Input represents Ht-PreHNE(alkyne) photocaged probe-bound Halo-POI in no light-exposed controls, or in light-exposed samples, residual photocaged probe-bound Halo-POI, or HNE(alkyne)-modified POI fused to Halo with residual photocaged probe on Halo. **D**) Relative expression of NCBP1-Flag(Tev-site)-Halo, Cand1-Flag(Tev-site)-Halo and IPO5-Flag(Tev-site)-Halo was assessed by anti-Flag blot, normalized by anti-tubulin. Samples were derived from HEK293T cells transfected with the corresponding constructs: POI-Flag(Tev-site)-Halo, followed by TeV protease treatment in cell lysate—performed to replicate the TeV-treatment step deployed in Fig. 2b, c. Consistent with Fig. 2b, TeV-cleavage was considerably limited for POI-Flag(TeV-site)-Halo when POI was NCBP1. Quantification is shown in the *Right Panel*. *p* values were calculated using Tukey’s multiple comparison test. All data present mean ± SEM (*n* = 3). **E**) Immunofluorescence was performed in HEK293T (Methods). Primary antibodies: anti-Cand1 (mAb, Santa Cruz, sc137055, 1:100) and anti-IPO5 (pAb, Abcam# ab187175, 1:100). Secondry antibodies: anti-ms IgG Alex Fluor 568 and anti-rabbit IgG Alex Fluor 568 1:2000. DAPI was used for nuclear staining. Images were taken using a Nikon spining-disk confocal microscope equipped with a 100X objective. Scale bar, 10 µm. Source data

We used T-REX^16,18^ to validate electrophile sensitivity. The key difference between Localis-REX (Fig. 1a,b) and T-REX is that Halo is fused to a specific POI in T-REX (Fig. 2a versus Fig. 1a,b). As liberated HNE occurs in the vicinity of the POI, T-REX assesses the POI’s electrophile sensitivity. Our previous studies show that parameters derived from T-REX are independent of specific POI expression levels^16,18^: a POI achieving a higher percentage (%) LO (that is, modification stoichiometry) under T-REX is more sensitive to a specific electrophile than another POI with a lower % LO. After T-REX in live cells, % LO can be assessed by click assay in lysates followed by in-gel fluorescence analysis (Fig. 2a). Alternatively, a more sensitive readout is achieved via first enriching the electrophile-modified POI via click-biotin pulldown, followed by western blot (Extended Data Fig. 4b,c).

All three Halo-fused POIs exhibited similar expression levels (Extended Data Fig. 4d). T-REX in live HEK293T cells revealed that among the three candidate POIs, only NCBP1 and IPO5 were kinetically privileged^15^ electrophile responders (Fig. 2b,c). Cand1, which houses 30 cysteines (Supplementary Fig. 8), was not electrophile sensitive. Notably, % LO(NCBP1) (Fig. 2d) was on par with the best electrophile responder proteins that we have examined^5–7,15–20,26–29^. Live imaging validated that NCBP1-Halo was chiefly nuclear restricted (Fig. 2e), consistent with NCBP1 housing an intrinsic nuclear localization signal. The % LO(IPO5) was more similar to weakly reactive proteins such as HuR^16,29,30^. Such proteins have not always shown phenotypic outputs when HNEylated under T-REX. IPO5 localized primarily to the nuclear periphery (Extended Data Fig. 4e). On the other hand, the non-electrophile-sensitive Cand1 largely resided in the cytoplasmic fraction (Extended Data Fig. 4e), providing interesting spatial contexts to protein hits from Localis-REX electrophile responder mapping. We also deployed the alternative biotin pulldown after T-REX, yielding a more sensitive readout: a similar trend of % LO across NCBP1, Cand1 and IPO5 was observed (Extended Data Fig. 4a–c).

**Extended Data Fig. 5:**
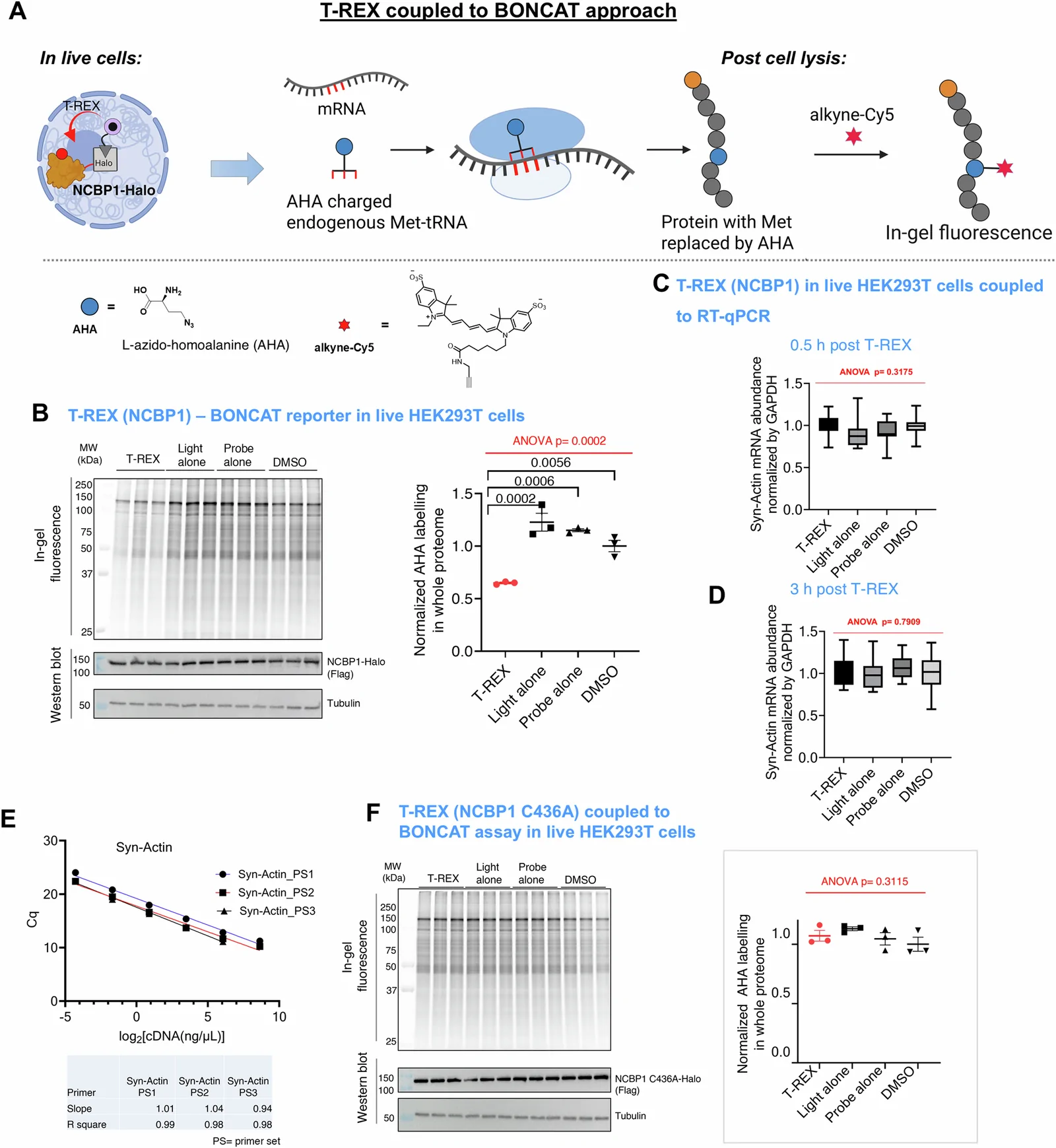
T-REX coupled to BONCAT, shows reduction of nascent proteome synthesis in response to NCBP1-HNEylation, but not NCBP1(C436A)-HNEylation. **A**) Schematic of T-REX coupled with BONCAT, followed by in-gel fluoresccence. **B**) T-REX was performed similarly to Fig. 2b, except cells were incubated in media without Methionine for 30 min prior to 2-min light exposure that triggers localised HNE (no-alkyne) generation within the vicinity of NCBP1(wt)-Halo. Post light exposure, cells were subjected to media containing L-Azido-homoalanine (AHA, 50 µM, 4 h) allowing AHA incorporation into nascent proteome (see Methods). Post lysis, samples were subjected to Cy5-alkyne Click-coupling reaction. Inset shows quantification. ‘Normalized AHA labelling in whole proteome’ (y-axis) shows relative values derived from the in-gel fluorescence signal of Cy5, normalized by anti-tubulin blot. *p* values were calculated with Tukey’s multiple comparisons test. Data present mean ± SEM (*n* = 3). **C**) qRT-PCR analysis (Methods and Supplementary Table 3) validated that NCBP1- HNEylation does not affect synthetic actin (Actin (K118TAG)) mRNA, compared to controls; ruling out that Syn-Actin suppression (for example, Fig. 2d) is an off-target consequence of the experimental setup deployed. T-REX was performed in HEK293T expressing NCBP1-Halo, Actin(K118TAG), and tRNA synthetase, and RNA isolation post T-REX-assisted NCBP1-specific HNEylation (see Methods) was carried out at: **C**) 0.5 h and **D**) 3 h. *P* values were calculated with Tukey’s multiple comparison test. All data present mean ± SEM (*n* = 12). **E**) Primer efficiency tests (Methods) [Data present mean ± SEM (n = 6; 3 biological replicates, 2 technical replicates each). **F**) T-REX-BONCAT experiments were peformed as in Extended Data Fig. 5A, except cells expressed NCBP1(C436A)-Halo over NCBP1-wt-Halo. Inset shows quantification. ‘Normalized AHA labelling in whole proteome’ (y-axis) corresponds to relative values derived from Cy5 in-gel fluorescence, normalized to anti-tubulin signal. *p* values were calculated with Tukey’s multiple comparisons test. Data present mean ± SEM (*n* = 3). Source data

To evaluate the extent to which the two HNE-sensitive POIs regulate translation, we coupled T-REX to GCER (Supplementary Fig. 1e). This workflow unveiled that HNEylation of NCBP1 suppressed translation, whereas IPO5-HNEylation did not (Fig. 2f,g and Supplementary Fig. 9a,b). Translation inhibition induced by NCBP1-HNEylation was verified using BONCAT (Extended Data Fig. 5a,b). These data indicated that NCBP1’s HNEylation was responsible for translation inhibition during nuclear Localis-REX.

### Of NCBP1’s 19 cysteines, only C436 labeling halts translation

**Fig. 3:**
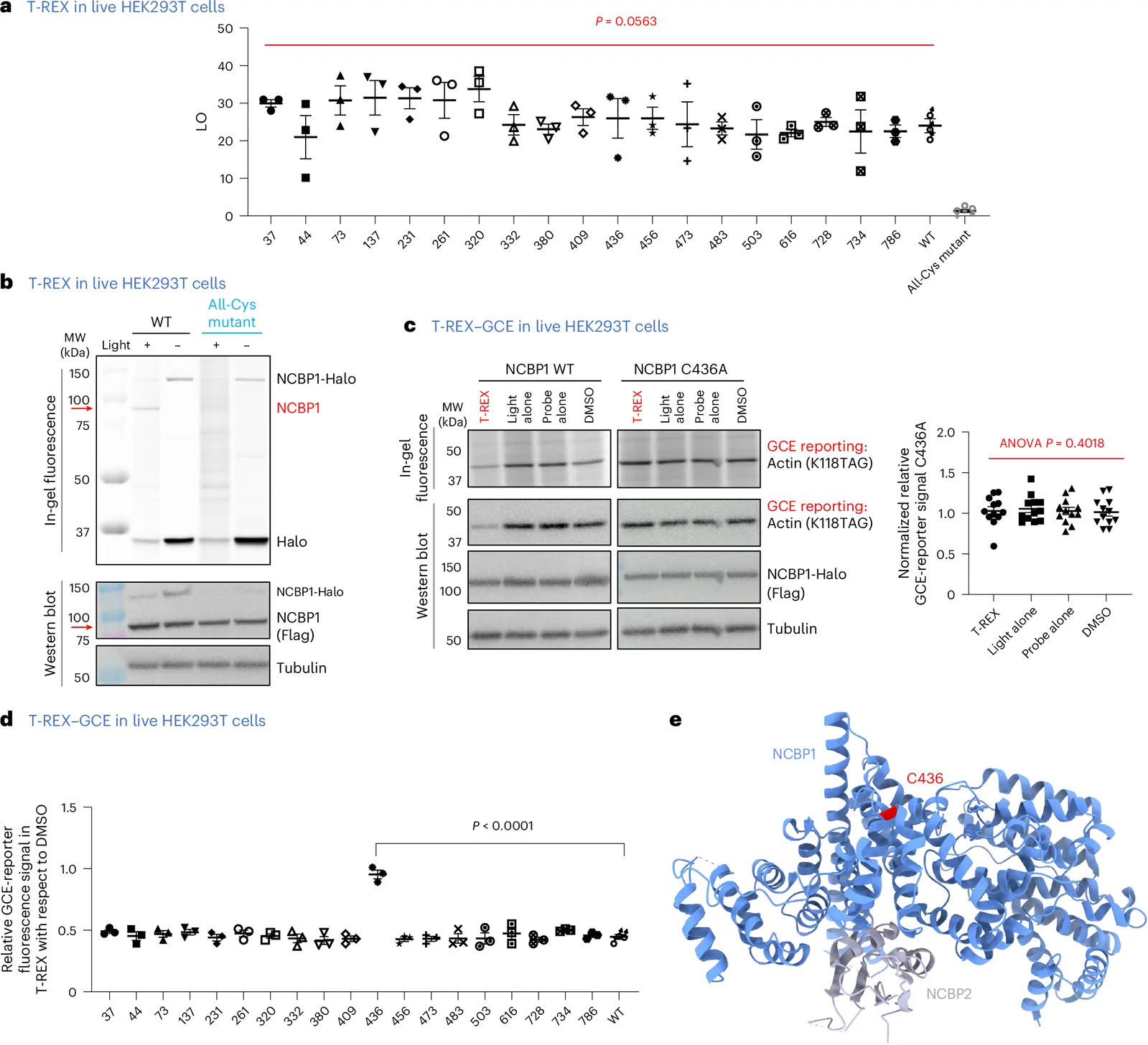
No single NCBP1-cysteine is responsible for sensing; only C436-specific HNEylation suppresses translation. **a**, HEK293T cells expressing NCBP1−Flag−TEV−Halo (WT or Cys-to-Ala single mutant) were subjected to T-REX (Fig. 2a), and HNE-LO was quantified as reported^16–18^. See Supplementary Fig. 11a for corresponding in-gel Cy5 fluorescence and anti-Flag and anti-tubulin western blots. *P* values were calculated with Tukey’s multiple comparisons test. Data present mean ± s.e.m (*n* = 3). **b**, Similar experiment to **a** was performed, except that comparison between WT and all-Cys mutant (where all 19 NCBP1 cysteines are mutated to alanines) was made. The bands corresponding to HNEylated-NCBP1 after TeV cleavage (top fluorescence) and the corresponding protein (bottom western blot) are indicated with a red arrow (see also Extended Data Fig. 6c). **c**, T-REX−GCER to assess the effect of NCBP1(WT or C436A)-targeted HNEylation on translation. Inset: quantification of the results on NCBP1(C436A) against all three T-REX technical negative controls. See relevant validations in Supplementary Figs. 9 and 14 that ruled out additional potential interference from technical and biological components. Inset: the corresponding quantification for NCPB1(C436A). *P* values were calculated with Tukey’s multiple comparisons test. Data present mean ± s.e.m. (*n* = 12). **d**, T-REX−GCER for all 18 single mutants compared to WT. Results were quantified in each case for the GCE signal (that is, translation efficiency) measured under T-REX divided by the DMSO control set (see Figs. 2f and 3c and Supplementary Figs. 12–14 for corresponding datasets from which quantification was derived). *P* values were calculated with Tukey’s multiple comparisons test. Data present mean ± s.e.m. (*n* = 3). **e**, Heterotrimeric complex of human NCBP1 and NCBP2 (Protein Data Bank 1H6K) with C436 in red (the 3rd molecule of NCBP2 is hidden behind the pale magenta NCBP1 molecule) (Supplementary Fig. 16). Source data

Although many amino acid side chains react with HNE, cysteine is the most intrinsically reactive coded amino acid^4,31^. Cysteine is most often identified as a kinetically privileged residue^15^. As an essential large subunit of the nuclear cap-binding complex (CBC), NCBP1 is highly conserved from plants to humans (Supplementary Fig. 10a). Human and mouse NCBP1 have 19 conserved cysteines (Supplementary Figs. 6 and 10a–c)^32^, several of which are solvent accessible^33^ (Supplementary Fig. 10b). Each cysteine (Cys) was individually mutated to alanine (Ala). The % LO(NCBP1) did not change across all single (Cys-to-Ala) mutants, relative to wild-type (WT) (Fig. 3a and Supplementary Fig. 11a). Mutation of all 19 cysteines to alanines ablated NCBP1 electrophile sensing (Fig. 3b and Supplementary Fig. 11b). All mutants, including the ‘all-Cys-to-Ala’ mutant, were soluble and expressed to similar levels as Halo fusions (Supplementary Fig. 11a–c). Although many proteins that we and others have investigated have specific electrophile-sensing cysteines^2–4,31,34^, several well-known electrophile responder proteins—including Keap1, the prototypical electrophile sensor protein^35^—harbor multiple cysteines capable of electrophile sensing.

**Extended Data Fig. 6:**
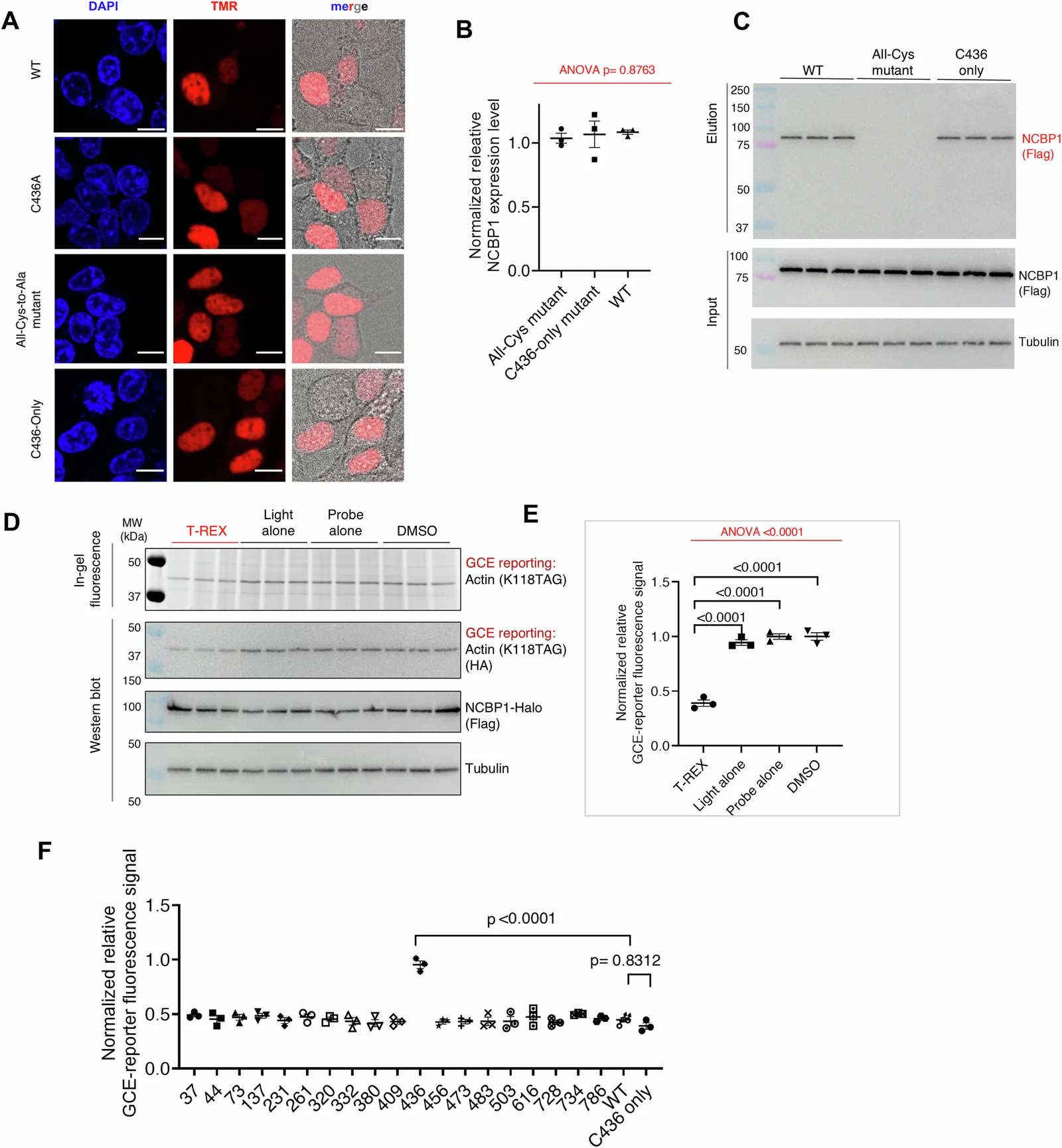
C436 as the sole functional site on NCBP1 that can propagate electrophile signal to protein translation inhibition. **A**) Live imaging shows nuclear localization of NCBP1-wt and mutants. HEK293T expressing NCBP1(wt)-Halo or indicated mutants were subjected to live imaging assay as described in Fig. 2e, using cell-permeable Halo-targetable TMR dye. Images were taken using a Nikon spining-disk confocal microscope equipped with a 100X objective. Scale bar, 10 µm. **B**) Validation of protein expression levels of indicated NCBP1 constructs expressed in HEK293T by western blot. p values were calculated with Tukey’s multiple comparisons test. All data present mean ± SEM. **C**) T-REX in HEK293T coupled to post-lysis Click biotin pulldown assay was performed as in Extended Data Fig. 4B, except that cells were transiently transfected with indicated NCBP1(wt or mutant)-Halo constructs. Briefly, signal in elution blot indicates HNEylated NCBP1 (wt or mutant, post TeV-cleavage that separates Halo and the protein of interest) following T-REX. **D**) and **E**) HEK293T co-transfected with NCBP1(C436-only)-Halo and GCE(actin) reporter were subjected to similar experiments as Fig. 3c (Methods). *p* values were calculated with Tukey’s multiple comparisons test. All data present mean ± SEM. **F**) Quantification of relative translation efficency as reported by GCE. The numbers within x-axis show Cys to Ala single-mutation sites. For **B**, **E**, **F**, *n* = 3 independent expreiments. Source data

Having validated that NCBP1 senses electrophiles through several cysteines, we tested the hypothesis that NCBP1-HNEylation is responsible for translation suppression when HNE is released in the nucleus. We first ascertained that NCBP1-HNEylation did not affect mRNA levels of the actin GCE reporter construct^11,12^ (Extended Data Fig. 5c–e). GCER post-T-REX-assisted NCBP1-HNEylation in cells showed largely similar levels of translation suppression to what we found upon Localis-REX with NLS-Halo (compare Fig. 1c, first plot, to Fig. 2f, inset). We repeated this experiment with each of the NCBP1 Cys-to-Ala single-point mutants (Supplementary Figs. 12 and 13). Intriguingly, NCBP1(C436A)-Halo HNEylation was unable to initiate translation suppression. However, translation suppression was observed in NCBP1(WT)-Halo and all other single-Cys mutants (Fig. 3c–e and Supplementary Fig. 14). Similar results were obtained using BONCAT (Extended Data Fig. 5f). We further confirmed that, in cells expressing either NCBP1(WT)-Halo or NCBP1(C436A)-Halo, basal translation efficiency remained the same (Supplementary Fig. 15). Thus, although several NCBP1 cysteines sensed electrophiles, only C436 was a functional first-responder residue regulating translation. We reported similar situations for other electrophile-responsive proteins, such as CDK9 (ref. ^6^) and HSPB7 (ref. ^27^). Imaging data also showed that both the all-Cys-to-Ala mutant and C436 mutants were largely nuclear localized (Extended Data Fig. 6a).

**Extended Data Fig. 7:**
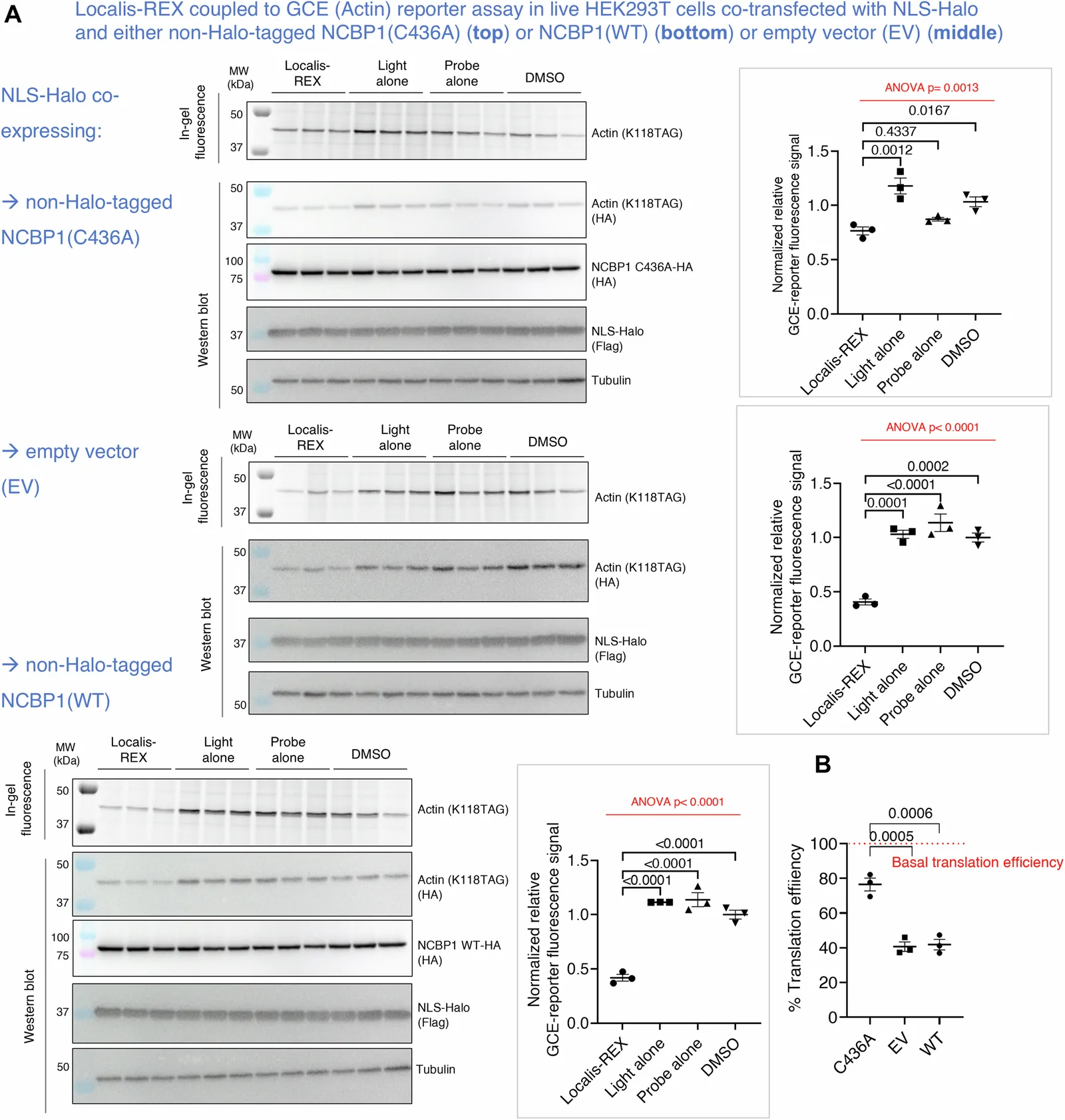
The extent of protein translation suppression induced by Localis-REX-assisted nucleus-targeted HNE release was attenuated in cells overexpressing (non-Halo-tagged) NCBP1(C463A) compared to empty vector or (non-Halo-tagged) NCBP1(wt) overexpressing cells, under otherwise identical conditions. **A**) Top panel: Localis-REX—GCE assay (see Fig. 1a, and Methods) was performed in HEK293T cells ectopically co-overexpressing NLS-Halo and (no-Halo-tagged) NCBP1(C436A)-HA. The data were analyzed using in-gel fluorescence and western blot analyses. Middle and lower panels: the above experiment was replicated using either Empty vector (EV) or NCBP1(wt)-HA in place of NCBP1(C436A). Insets on right show corresponding quantification in each case. p values were calculated with Tukey’s multiple comparisons test (*n* = 3). Data present mean ± SEM. **B**) Relative comparison of the 3 datasets in (A) as fold-change suppression of translation efficiency in cells transfected with either NCBP1(C436A), empty vector (EV), or NCBP1 (WT). Values in each case were obtained by GCE-reporter signals measured under T-REX normalized to that of samples under DMSO control. 100% on the y-axis designates the basel translation efficieny. 59 (±5)% translation suppression from basal translation efficiency was measured in EV-transfected cells compared to 24 (±6)% in cells overexpressing NCBP1(C436A). *p* values were calculated with two-tailed, unpaired Student’s *t*-test (*n* = 3). Source data

To further reinforce our hypothesis that NCBP1 electrophile sensing in the nucleus is a key regulatory axis for protein synthesis downregulation, we replicated Localis-REX in HEK293T cells co-expressing NLS-Halo and non-Halo-fused NCBP1(C436A) and assayed for translational efficiency (Extended Data Fig. 7). We compared in parallel the extent of translation efficiency suppression after Localis-REX nuclear-targeted HNE generation in cells co-transfected with NLS-Halo and either non-Halo-fused NCBP1(WT) or empty vector (that balanced the amount of plasmid encoding non-Halo-fused NCBP1(WT) or NCBP1-(C436A)). Overexpression of NCBP1(C436A) strongly, albeit not fully, blocked the translation suppression observed in response to nucleus-specific HNE buildup in empty vector control or non-Halo-fused-NCBP1(WT)-co-expressing samples under otherwise identical conditions (Extended Data Fig. 6b,c). This outcome is consistent with the data above, which showed that NCBP1 is the main HNE sensor in the nucleus and that its HNEylation at C436 dampened translation. These results further indicated that other sensors, such as IPO5 (Fig. 2c,d), also exist.

**Extended Data Fig. 8:**
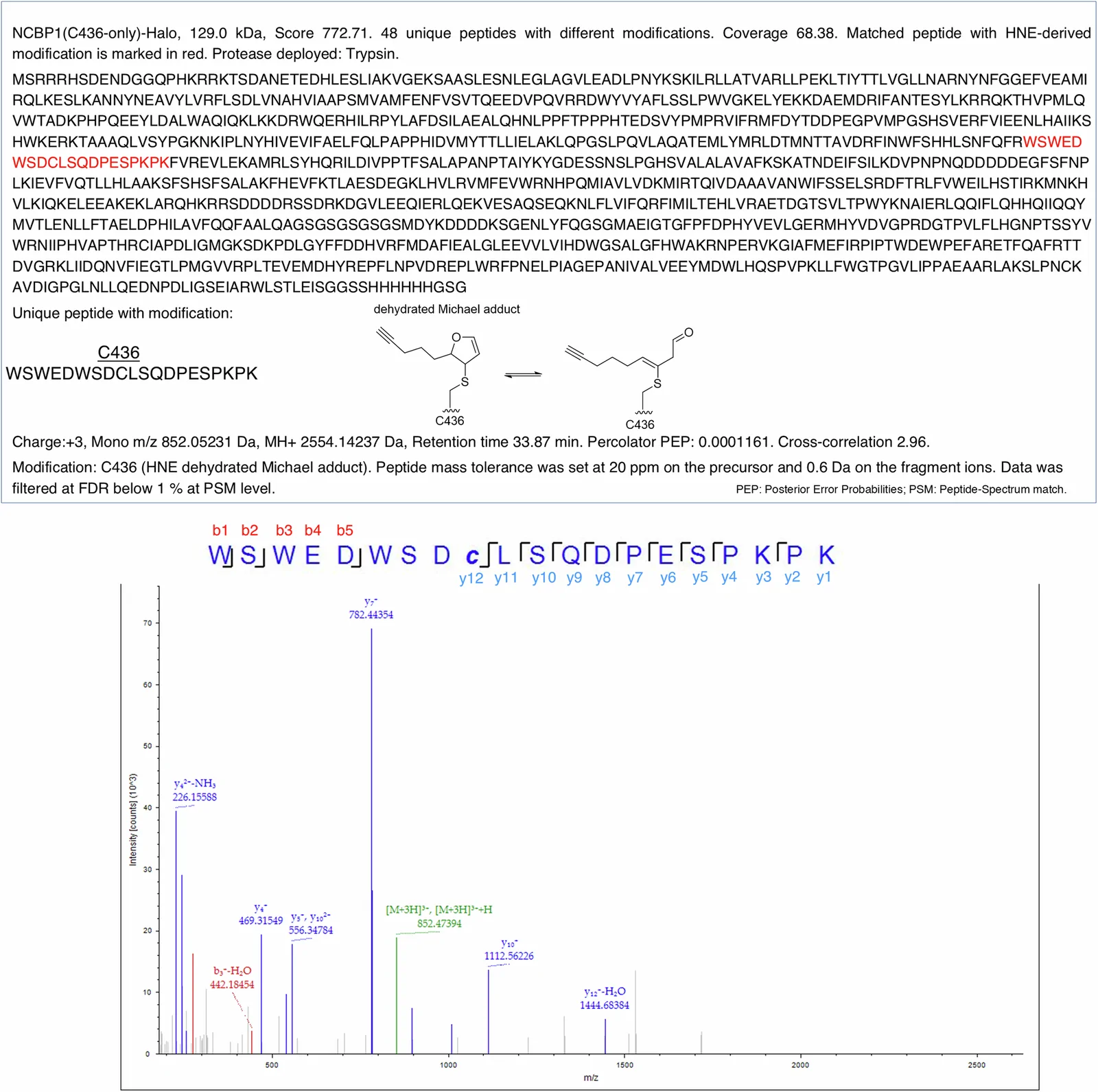
Digest mass spectrometry analysis validated C436 as functional HNEylation site consistent with all other biochemical and cell-based datasets. The experimental workflow and data analysis were described in ‘Mass spectrometry analysis of HNEylation site following T-REX in live HEK293T cells expressing the indicated NCBP1 construct’ (Methods). Source data

All these data point to C436 as a bona fide HNE-sensing residue that functionally couples electrophile sensing to translation. To unambiguously test this hypothesis, we engineered NCBP1-Halo housing C436 as its only cysteine (hereafter termed ‘NCBP1(C436-only)’). NCBP1(C436-only) expression and solubility remained similar to WT and all-Cys-to-Ala mutant (Extended Data Fig. 6b). After T-REX, % LO(NCBP1) also remained similar between NCBP1(C436-only) and NCBP1-WT (Extended Data Fig. 6c). T-REX-coupled GCER demonstrated that NCBP1(C436-only) HNEylation induced the same extent of translation suppression as NCBP1(WT)-Halo HNEylation (Extended Data Fig. 6e,f). After T-REX, we identified a C436-HNEylated peptide within NCBP1(C436-only)-Halo enriched from live HEK293T cells (Extended Data Fig. 8), after enrichment digest mass spectrometry^5,6^. Altogether, these experiments strongly support a model in which translation inhibition caused by nuclear HNE buildup is ascribable to NCBP1(C436)-HNEylation.

### Translation suppression was independent of NCBP2

NCBP1 partners with NCBP2 to form the CBC that performs numerous context-specific operations in eukaryotic mRNA biogenesis, mRNA homeostasis surveillance and mRNA translation initiation^36^. The available structure of NCBP1–NCBP2 heterodimer, with and without a bound 5′ 7-methylguanosine (m^7^G) cap mimic^33,37^, shows that C436 is solvent exposed and distal to the heterodimer interface (Fig. 3e and Supplementary Fig. 16a,b). T-REX on NCBP2-Halo validated that NCBP2 is not an HNE sensor (Supplementary Fig. 16c). Moreover, NCBP1-HNEylation suppressed translation as efficiently when NCBP2 was overexpressed, as in empty-vector-transfected cells (Supplementary Fig. 16d). NCBP1/NCBP2 association was also unperturbed by NCBP1-specific HNEylation (Supplementary Fig. 17).

### Survival and homeostasis-related expression were affected

**Extended Data Fig. 9:**
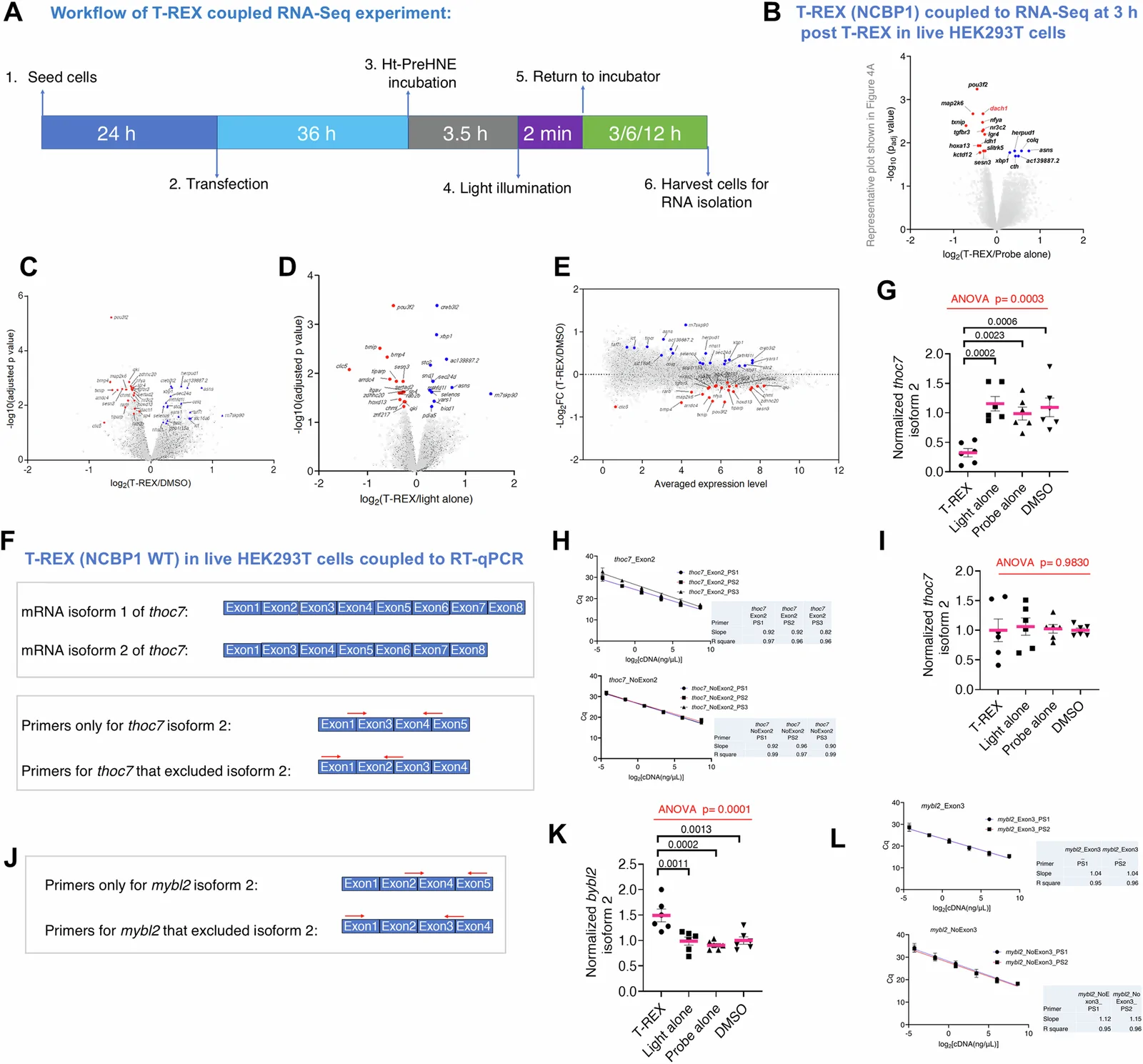
HNEylation of NCBP1 affects mRNA splicing. **A**) Workflow for T-REX-coupled RNA-Seq experiments. **B**–**D)** Volcano and **E**) MA plots derived from RNA-seq analysis showing statistically-significant differentially-expressed genes (SDEs) for T-REX versus indicated controls (*3* *h post T-REX*). For volcano plots (*top 3 plots*): x-axis-log_2_(Fold change (FC) each gene between T-REX and one technical control (probe alone; DMSO; or light alone)); y-axis-adjusted p value [-Log10(padj)]. (top-left plot is identical to Fig. 4a). **Red** dot-genes significantly downregulated by T-REX; **blue** dots-same but upregulated. Names are shown for arbitrarily-selected SDEs. See Supplementary Data 2. In MA scatter plot (*see bottom plot*the x-axis-average expression level of each gene; y-axis-log_2_(FC each gene between T-REX and DMSO). Only hits with p < 0.05 for T-REX versus at least two out of 3 technical controls were considered. The names of hits were marked in the MA plot; **red** dots-downregulated SDEs, **blue** dots-upregulated SDEs. **F**) Schematic of relevant *thoc7* mRNA isoforms (*top*) and qRT-PCR primer-annealing sites (*bottom*). **G**) qRT-PCR results reporting the relative abundance of isoform 2 normalized by abundance of isoform-1. The plot shows quantification of mRNA of thoc7 isoform-2 across different conditions. *p* values were calculated with Tukey’s multiple comparison test. Data present mean ± SEM (n = 6). **H**) Primer efficiency test (n = 6; 3 biological replicates, 2 technical replicates each). **I**) Similar setup/quantification as **G**, except HEK293T were transfected with NCBP1(C436A)-Halo over NCBP1(wt)-Halo. *p* values were calculated with Tukey’s multiple comparison test. Data present mean ± SEM (n = 6). **J**) Schematic of relevant *mybl2* mRNA isoforms and illustration of qRT-PCR primer-annealing sites. **K**) qRT-PCR results on isoform-2 abundance (generated through skipping of exon 2) normalized to other isoforms. Data present mean ± SEM (n = 6). **L**) Two primer sets were designed to quantify mRNA of mybl2 isoform-2 by RT-qPCR analysis Data present mean ± SEM (n = 12). See Supplementary Table 3 for primers. Source data

NCBP1 is essential for CBC function^36,38–42^. Given the CBC’s crucial roles in RNA maturation, we next investigated the effects of NCBP1-HNEylation on RNA levels using RNA sequencing (RNA-seq) at three different timepoints (3 hours, 6 hours and 12 hours) after T-REX-directed NCBP1-specific HNEylation. We compared these changes against all three technical T-REX controls (light alone, REX probe alone and dimethyl sulfoxide (DMSO) vehicle) (Extended Data Fig. 9a and Supplementary Data 2). Pairwise differential analyses were applied to cells that had undergone NCBP1-specific HNEylation relative to each control (Extended Data Fig. 9b–e and Supplementary Figs. 18 and 19). Each set was performed in independent biological quadruplicates. The numbers of significantly differentially expressed (SDE) genes, either upregulated or downregulated, resulting from each comparative set are illustrated in Venn diagrams, along with their identities and thresholds (adjusted *P* values) implemented (Supplementary Fig. 20).

STRING analysis^43^ of upregulated and downregulated SDE genes at each timepoint (Supplementary Fig. 20) showed that several genes lie outside of known interactions. The outputs from early timepoints after NCBP1-specific HNEylation particularly stood out in this regard. Subjecting the same SDE gene list to g:Profiler analysis^44^ showed significant enrichment of defined Gene Ontology subterms under Biological Processes and, to some extent, Molecular Functions (Supplementary Fig. 21) in all timepoints. A closer assessment revealed that approximately 62% (53 out of 86 total) of these top-ranked SDE genes are associated with the following (functions): (1) transcription factors/co-regulators (for example, *dach1*, *pou3f2*, *xbp1* and 10 others); (2) stress/cell death response (for example, *txnip*, *hrk*, *bnip3(l)*, *sesn3*, *egln3* and 15 others); (3) mRNA biogenesis/translation (for example, *snrpd3*, *qki*, *tincr*, *mars1* and *yars1*); and (4) receptor–nucleus signal transduction (for example, *bmp4*, *map2k6*, *cxcr4*, *igfbp5* and 10 others) (Supplementary Data 3). These outputs demonstrate that electrophile modification of NCBP1 has broad-spectrum ramifications for cell homeostasis and survival.

### NCBP1(C436) signaling modulated splicing of several genes

**Fig. 4:**
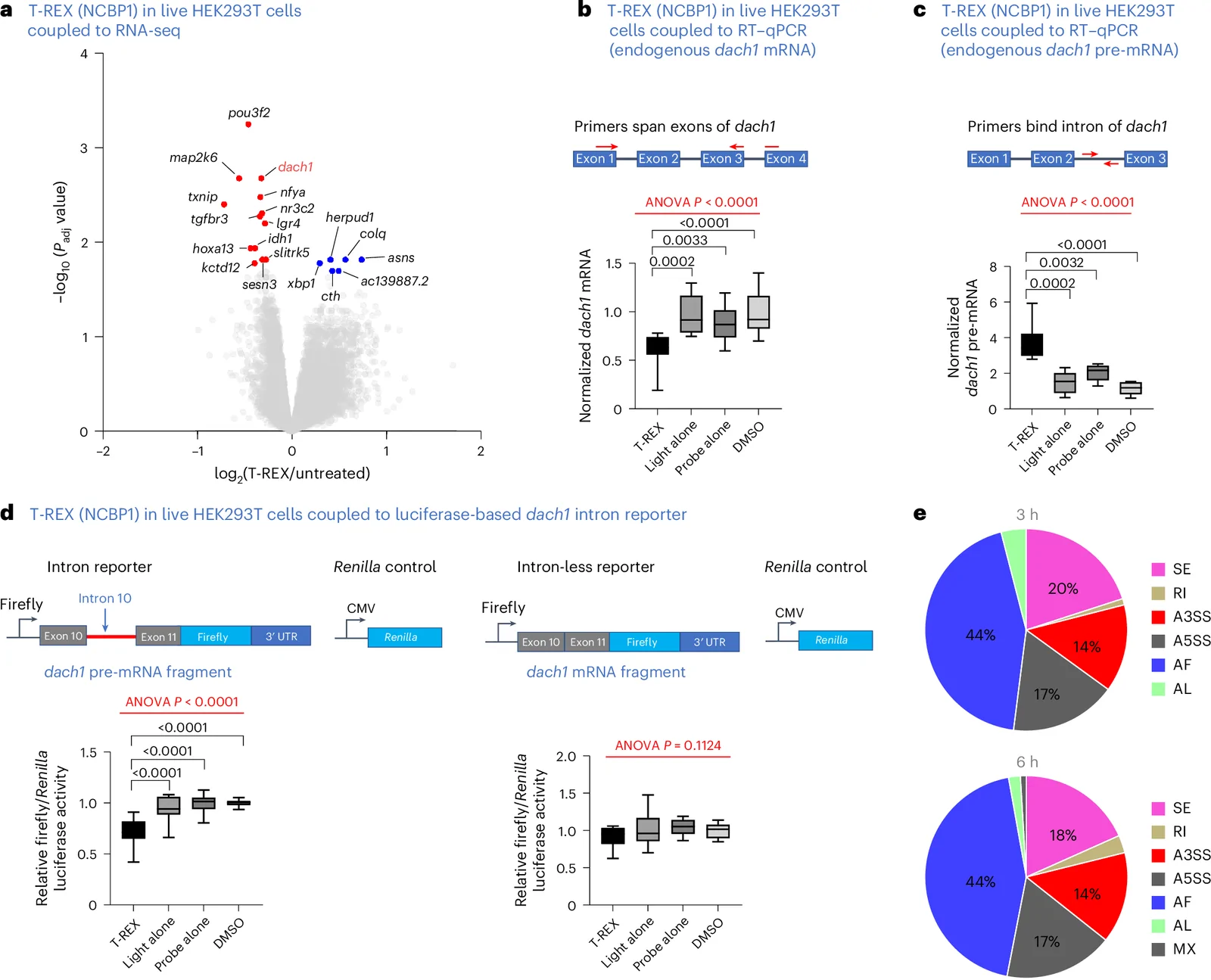
HNEylated NCBP1 modulates splicing. **a**, Differental transcript expression from HEK293T cells subjected to NCPB1-HNEylation by T-REX against non-treated cells (3 hours after T-REX shown; see Extended Data Fig. 9 and Supplementary Figs. 18 and 19 for workflow and additional timepoints and controls). SDE genes are marked in blue (upregulated) and red (downregulated), respectively; non-SDE genes are shown in gray (Supplementary Data 2 and 3). For the corresponding MA scatter plot featuring fold change versus mean expression, see Extended Data Fig. 9e. **b**, Exon-targeted RT−qPCR in HEK293T cells validated suppression of DACH1 mRNA after T-REX-assisted NCBP1-HNEylation, against negative controls. *P* values were calculated with Tukey’s multiple comparison test (*n* = 12). **c**, Intron-targeted RT−qPCR in HEK293T cells revealed upregulation of DACH1 pre-mRNA after T-REX-assisted NCBP1-HNEylation, against negative controls. *P* values were calculated with Tukey’s multiple comparison test (*n* = 12). **d**, Validation by orthogonal reporter assays. Reporters to assay splicing were built by installing the indicated exons from *dach1*, with/without the intron (left and right, respectively), upstream of a firefly luciferase reporter, with *dach1*’s 3′ UTR downstream. HEK293T cells expressing NCBP1(WT)-Halo, CMV-*Renilla* luciferase (internal control) and intron or intron-less reporter were subjected to T-REX against indicated controls, and firefly and *Renilla* luminescence were measured (Methods). Replacing NCBP1(C436A)-Halo with NCBP1(WT)-Halo showed no change of normalized luciferase signal (Supplementary Fig. 24) (*n* = 18). Box and whisker plots: line, median; box, 25th−75th percentiles; whiskers, 1st−99th percentiles. **e**, Alternative splicing events affected by NCBP1-HNEylation. In total, 1,266 genes were analyzed across three timepoints after T-REX-assisted NCBP1-HNEylation, against negative controls, with thresholds for significance defined in the main text and Methods. Data from 3-hour and 6-hour timepoints are shown (Supplementary Data 4 and Table 4). A5(3)SS, alternative 5′/(3′) splice sites; AF, alternative first exon/alternative promoter usage; AL, alternative last exon splicing; h, hours; MX, mutually exclusive exon splicing events; RI, retained intron; SE, exon skipping. Source data

We were further intrigued by the fact that a large number of our top-ranked SDE genes (either upregulated or downregulated SDE genes) (Supplementary Data 3) comprised disease-relevant genes that undergo alternative splicing, yielding protein variants with potentially different functions. We first investigated one such gene, *dach1*, by RT−qPCR (Supplementary Table 3). The *dach1* gene is an essential transcription factor in developmental physiology and disease and was downregulated upon NCBP1-HNEylation (Fig. 4a and Extended Data Fig. 9e). After T-REX in HEK293T cells ectopically expressing NCBP1-Halo, and consistent with our RNA-seq results above (Fig. 4a and Extended Data Fig. 9b,e), RT−qPCR data showed selective depletion of endogenous *dach1* mRNA transcript, against all controls (Fig. 4b, Supplementary Fig. 22 and Supplementary Table 3). By contrast, the corresponding abundance of pre-mRNA (assayed using RT−qPCR primers spanning the intronic region flanked by exons 2 and 3) increased upon NCBP1-HNEylation (Fig. 4c and Supplementary Table 3). These observations were not due to changes in mature *dach1* mRNA stability. As expected, the half-life of the corresponding pre-mRNA was short (approximately 2−12 minutes, preventing precise calculation); yet this also seemed relatively unperturbed (Supplementary Fig. 23). These data indicate that *dach1* downregulation induced by NCBP1-HNEylation was likely due to inhibition of splicing.

To independently substantiate the above findings, we built ratiometric-readout-based *dach1-*targeted dual-luciferase reporter systems^29^, targeted at an intron of amenable size (intron 10, approximately 3 kb), flanked by exons 10 and 11. Thus, either an exon−exon sequence (for intron-less reporter) or an exon−intron−exon sequence (for intron 10 reporter), from *dach1* mRNA, was fused upstream of the firefly luciferase (ff-Luc) reporter construct, whereas the *dach1* 3’ untranslated region (UTR) was maintained downstream of ff-Luc. Co-expressing this construct alongside a constitutive *Renilla* luciferase (R-Luc) reporter construct in HEK293T cells, we found that NCBP1-HNEylation selectively depleted the intron 10 reporter signal (normalized to R-Luc internal control) but had no effect on the intron-less reporter signal (Fig. 4d). These data further pinpoint NCBP1-HNEylation-driven splicing inhibition at intron 10. Notably, when we deployed NCBP1(C436A)-Halo in place of the WT protein, no change in the intron reporter signal was observed after T-REX (Supplementary Fig. 24).

Beyond its essential transcriptional roles, DACH1 has also been implicated in downregulating cytoplasmic translational programs^45^. We generated CRISPR-Cas9-assisted *dach1*-silenced (knockout (KO)) lines (>90% silencing efficiency in both KO lines; Supplementary Fig. 25a). Likely linked to DACH1’s essential role in cell growth, DACH1-KO lines grew significantly slower than the KO-control line or native HEK293T cells (Supplementary Fig. 25b). T-REX–GCER in these lines showed the same extent of translation inhibition across KO-1, KO-2, KO-control and native cells (Supplementary Fig. 25c–e). These data ruled out DACH1 as a player in NCBP1-HNEylation-induced protein synthesis stall.

### NCBP1-HNEylation promoted alternative splicing

Although changes in *dach1* splicing emerged not to be involved in translation downregulation upon NCBP1-HNEylation, our investigations above indicated that NCBP1-HNEylation could affect splicing. The CBC indeed binds pre-mRNA transcripts, after co-transcriptional 5′ m^7^G capping^38,39^. It plays several roles in nuclear RNA processing, including regulation of constitutive splicing obligatory for mRNA maturation^36,38,39^. Structural studies (Supplementary Fig. 16b) hint that NCBP1’s association with NCBP2 is essential for enhancing the latter’s affinity to 5′-capped transcripts^33^. The CBC promotes the initial interaction between spliceosome factors and the 5′ end of the pre-mRNA transcript (at the first intron), augmenting spliceosome assembly and splicing^33,38,39^. To examine if the newly discovered NCBP1 electrophile signaling affects alternative splicing events, we launched a comprehensive genome-wide differential splicing analysis of our RNA-seq datasets. Following the established SUPPA2 pipeline^46^ (Methods), we assigned relative abundances of the splicing events or transcript isoforms (referred to as proportion spliced-in (PSI)^47^) for each transcript across all three timepoints, under four different conditions: T-REX (NCBP1-HNEylation) and three controls (DMSO-alone, light-alone or probe-alone treatment). The difference in the mean PSI (dPSI) between the two chosen conditions (T-REX versus designated control) was subsequently derived for each transcript, for each type of alternative events scored, along with associated adjusted *P* value (*P*_adj_). Events were grouped accordingly: skipping of exon (SE, including both exon inclusion/exclusion, the most common event in mammals^48^); retention of intron (RI); alternative 5’ and 3’ splice site selection (A5SS and A3SS); alternative first exon (that is, alternative promoter usage) (AF) and alternative last exon splicing (AL); and mutually exclusive (MX) exon. Hits that fulfilled the following requirements were designated as ¢significant events¢ at each timepoint: *P*_adj_ ≤ 0.05 and appeared under at least two pairwise comparisons (that is, T-REX versus two different controls) out of the three (Supplementary Data 4). As summarized in Supplementary Table 4, changes in alternative splicing were not correlated with changes in its mRNA expression.

Using the above-mentioned thresholds for significance, our differential splicing analysis showed that AF (44%) followed by SE (20%), A5SS (17%) and A3SS (14%) were the most-preferred alternative events triggered at 3 hours after NCBP1-HNEylation (Fig. 4e). Interestingly, in a report where CBC influences alternative splicing in *Arabidopsis thaliana*^49^ (approximately 29% sequence identity to mouse and human NCBP1), *Arabidopsis* CBC affects 101 alternative splicing events (*P* ≤ 0.1) from the 252 analyzed, with more than 50% of the alternative splicing events being AF^49^, likely consistent with the direct positioning of CBC at 5’ cap and the first splice site of the pre-mRNA transcript^38,39^ and, indeed, broadly consistent with our own results. In our survey, 3 hours after NCBP1-HNEylation, 200 splicing events, across 119 distinct transcripts, were significantly affected among the 1,266 genes analyzed, where transcript counts were determined using Salmon^50^. At 6 hours, the relative proportions of alternative splicing event types remained largely similar, with an additional contribution also from MX exons (Fig. 4e).

For instance, 6 hours after NCBP1-HNEylation, *thoc7* (encoding THO complex subunit 7) underwent a statistically significant loss of alternative splicing (skipping of exon 2 (SE)), leading to isoform 2 (Extended Data Fig. 9f). *Thoc7* encodes a functional spliceosome-associated protein integral to the THO complex that is involved in mRNA nuclear export. We validated this finding using RT−qPCR. Leveraging primers targeting *thoc7* isoform 2, normalized by results using primers targeting FL (isoform 1) (Supplementary Table 3), the relative abundance of exon 2 lacking isoform 2 was significantly reduced after T-REX in HEK293T cells expressing NCBP1-Halo compared to all three technical controls (Extended Data Fig. 9g,h). Replacing NCBP1(WT)-Halo with NCBP1(C436)-Halo ablated the observed reduced abundance of *thoc7* isoform 2 (Extended Data Fig. 9i). Similarly, *mybl2* (encodes myb-like protooncogene 2) underwent a statistically significant gain of SE after NCBP1-HNEylation. This SE process produced isoform 2, which lacks exon 3 (Supplementary Data 4 and Table 4). We validated this SE gain by RT−qPCR (Extended Data Fig. 9j–l and Supplementary Table 3). Notably, alternative splicing events of a significant number of genes associated with ribosomal translation were affected by NCBP1-HNEylation, including eukaryotic translation initiation factor 4E-binding protein 1 (4ebp1 (also known as elf4ebp1)); several ribosomal-subunit proteins (for example, rpl9, rpl32 and mitochondrial ribosomal subunit proteins, for example, mrpl13 and mrpl23); and proteins binding hibernating ribosomes (for example, ccdc124) (Supplementary Data 4).

### eIF2α and 4E-BP1 phosphorylation were unaffected

We also explored the extent to which key players driving translation initiation were involved in this process^36,38,39^. After CBC-dependent translation initiation (CT) and nonsense-mediated mRNA decay (NMD)-associated quality control, steady-state translation cycles are driven by eIF4E-dependent 5’-cap-directed translation initiation (ET), as CBC is replaced by eIF4E. However, the functional boundaries between CT versus ET are becoming increasingly blurred^36,38,39^. Particularly under stress, the CT-based initiation may be more operative^38,39^. Nonetheless, both CT-supported and ET-supported initiation mechanisms require active eIF2α: both are suppressed by phosphorylation of eIF2α, a global stress-responsive means to rewire translational programs. However, additional stress-reponsive translational remodeling programs are emerging^36,38,39,51–54^. A major mechanism includes hypophosphorylation of eIF4E-binding protein 1 (4E-BP1), which elicits ET inhibition with minimal effect on CT^38^. This mechanism likely explains heat shock, late-stage hypoxia and serum starvation-dependent translational responses, where CT is better able to support the cell translational capacity^36,38,39,51–54^. We thus assessed the phosphorylation of 4E-BP1 and eIF2α after NCBP1-HNEylation. Neither was changed significantly (Supplementary Fig. 26).

### NCBP1-HNEylation promoted *s6k1* alternative splicing

**Fig. 5:**
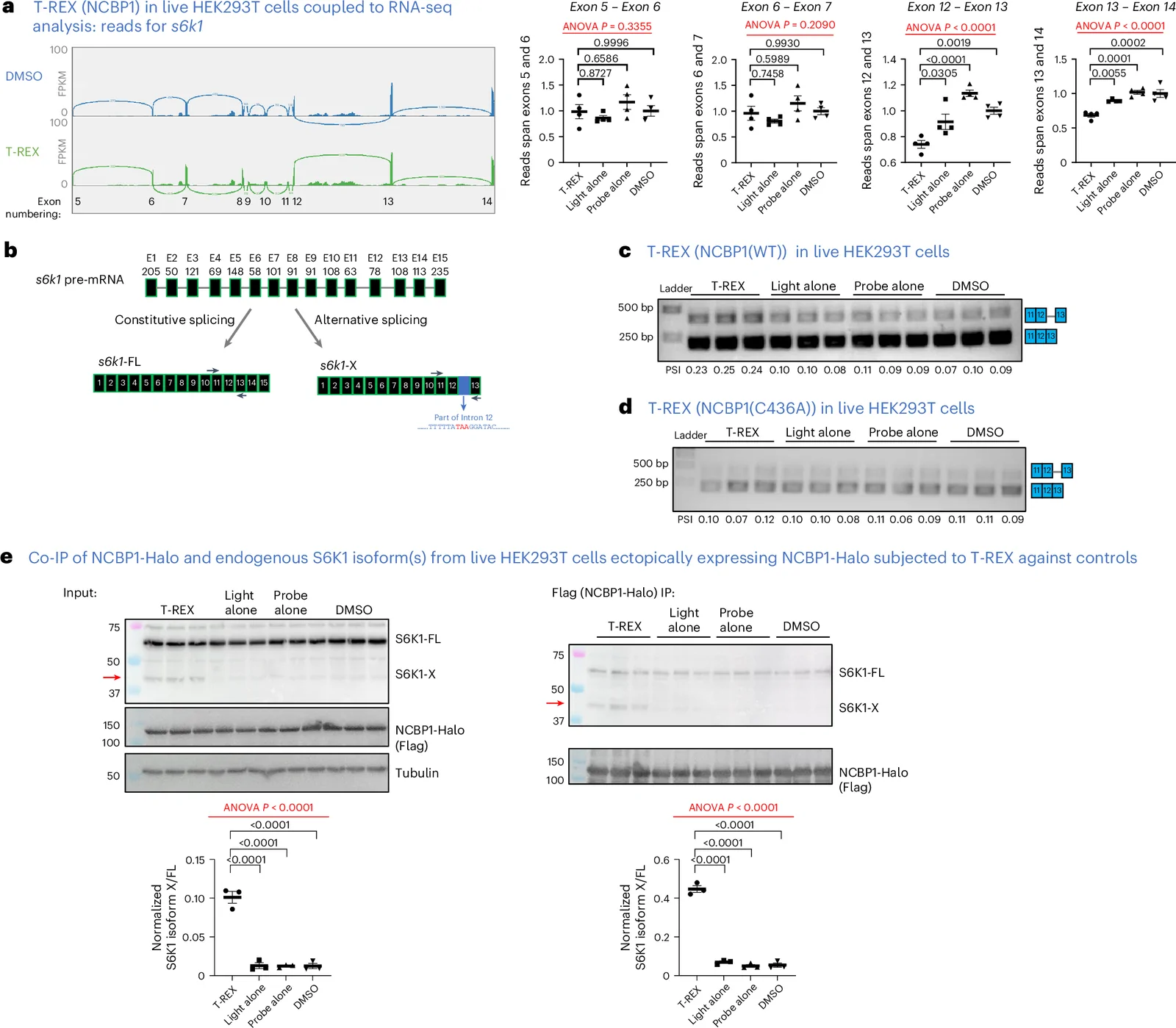
HNEylated NCBP1 generates an alternatively spliced S6K1 protein dominantly inhibits translation. **a**, Representative Sashimi plots^57^ (top) and quantification (bottom) illustrating the reduction in reads for S6K1: exons 12 and 13 and exons 13 and 14, after T-REX-assisted NCPB1-HNEylation against DMSO. No decrease was observed in preceding exons. *P* values were calculated with Tukey’s multiple comparison test. All data present mean ± s.e.m. (*n* = 4). Genomic coordinates on Sashimi plot *x* axes are omitted for clarity. Values within arcs depict the number of junction reads. Sashimi plots are shown for only one out of four biological replicates (T-REX versus DMSO). See plots below for quantification of read spans across indicated exons, across all datasets and under T-REX versus technical controls. FPKM, fragments per kilobase of transcript per million mapped reads. **b**, Exon structure of S6K1 pre-mRNA (top, E1−E15; E indicates exon, and numbers above exons correspond to the number of base pairs and spliced forms; bottom left, S6K1-FL mRNA arising from constitutive splicing; bottom right, S6K1-X, an alternative spliced isoform, formed upon NCBP1-HNEylation (this work)). S6K1-X lacks E14 and E15 and partially lacks ‘E13’ due to alternative splicing at intron 12, which is partly retained. The pair of arrows shown for S6K1-FL and S6K1-X corresponds to data in **c** and **d** (see also Supplementary Fig. 27). **c**, After T-REX-enabled NCBP1-HNEylation, amplicons featuring exon 11−exon 12−exon 13 (bottom band, 215 bp) and exon 11−exon 12−intron 12−exon 13 (top band, 412 bp) were analyzed using primers in **b** (Supplementary Table 3). PSI: Proportion Spliced In. Each band was PCR amplified and validated by sequencing (*n* = 3 each set) (Supplementary Fig. 28). **d**, Similar to **c** except HEK293T cells expressing NCBP1(C436A)-Halo (*n* = 3 each set). **e**, Left, in HEK293T cells, NCBP1-HNEylation upregulated S6K1-X (45 kDa, red arrow). Right, results from Flag immunoprecipitation (Methods and Extended Data Fig. 10a,b). Ratio of S6K1-X:S6K1-FL in lysate versus after Flag immunoprecipitation, normalized by NCBP1-Halo expression, was calculated. *P* values were calculated with Tukey’s multiple comparisons test. All data present mean ± s.e.m. (*n* = 3). E, exon; IP, immunoprecipitation. Source data

These data led us to zoom into specialized factors unique to CBC-bound mRNA translation. Upregulation of CBC-dependent mRNA translation is subsequently driven by a series of signaling actions involving activation of mTORC1 that phosphorylates ribosomal protein S6 kinase 1 (S6K1, also known as p70S6K), promoting the resultant activated p-S6K1’s recruitment and phosphorylation of SKAR (a component of the exon junction complex (EJC))^55^. Beyond translation initiation and quality control, CBC is emerging to function also in the steady-state translation, particularly during stress^38,39,51–53,56^. Interestingly, our RNA-seq data revealed that *rps6kb1* transcript (encoding protein S6K1) was robustly affected after NCBP1-HNEylation: Sashimi plots^57^ showed significantly reduced intronic reads spanning exon 13 and 14, and also exons 12 and 13, but not for the intronic reads spanning upstream exons, such as exons 5 and 6, and exons 6 and 7 (Fig. 5a).

S6K1 has several splice variants, including a kinase-dead FL variant and alternative shorter isoforms arising from alternative events between exons 6 and 7 (thereby resulting in the loss of exons 8−15, along with modified/alternative exon 7)^58^. The short isoforms from both mouse and human—identical to S6K1-FL up to exon 6 but missing more than half of the conserved kinase domain—are considered catalytically inactive. Interestingly, the short isoforms manifest context-specific oncogenic behaviors, whereas the kinase-dead FL variant is tumor suppressive^58,59^. Our analysis (Fig. 5a) showed that NCBP1-HNEylation promoted an alternative exon-skipping event, distinct from those reported (Fig. 5b and Supplementary Fig. 27). Specifically, NCBP1-HNEylation produced S6K1-X that lacks exons 14 and 15 and has an alternatively spliced intron 12 (Supplementary Fig. 27). Quantitative PCR analysis of endogenous S6K1, after NCBP1-HNEylation in HEK293T cells, confirmed selective buildup of S6K1-X-associated amplicon that includes part of intron 12 (264 bp), under T-REX, compared to all three controls (Fig. 5c). A significantly increased PSI^47^ index was found compared to the amplicon lacking the above-mentioned intron 12 constituent. Both amplicons were further validated by sequencing (Supplementary Fig. 28). Notably, when NCBP1(C436A) replaced NCBP1(WT) in the same qPCR assay after T-REX under otherwise identical conditions, HNE-sensing-active but HNE-signaling-deficient NCBP1(C436A) did not produce S6K1-X (Fig. 5d). We proceeded to investigate the functional ramifications of the resulting S6K1-X protein.

### NCBP1-HNEylation produced a truncated S6K1 protein, S6K1-X

**Extended Data Fig. 10:**
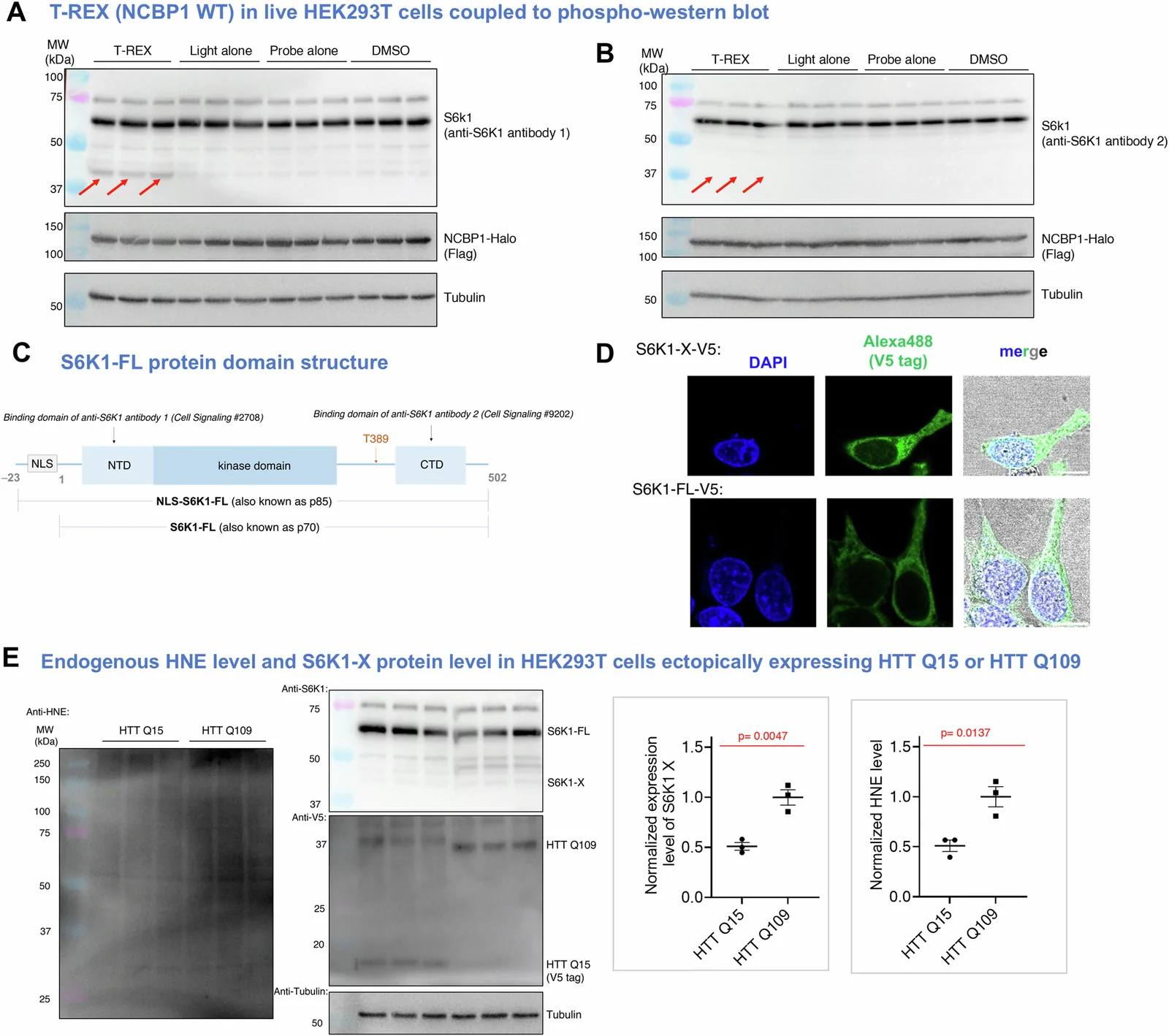
S6K1-X is a suppressor of protein translation. **A**) and **B**) Expression of S6K1 isoform-X, following NCBP1-HNEylation, was examined using two different S6K1 antibodies. See also Fig. 5e. 2 h post T-REX in HEK293T expressing NCBP1-Halo, cells were lysed and analyzed by western blot using indicated antibodies. **C**) Domain structure of S6K1-FL highlighting double (or multiple) bands typically observed by western blot, corresponding to two key forms: S6K1-FL (p70) and NLS-S6K1-FL (p85). S6K1-FL lacks the nuclear localization sequence (NLS) encoding 23 amino acids at the N-terminal end. **D**) S6K1 full length and isoform-X are primarily localized to the cytoplasm. HEK293T were transfected with S6K1-X-V5 or S6K1-FL-V5. 36 h later, the cells were fixed and incubated with blocking buffer. Anti-V5 (Invitrgen R96025, 1:200) incubation (2 h) was followed by washing with DPBS three times and anti-ms IgG Alex Fluor 488 (1:2000) secondry antibody treatment. Cells were again rinsed three times with DBPS followed by treatment with DAPI (2 µg/mL in DPBS, 5 min), and final rinsing with DPBS. Images were taken using a Nikon spining-disk confocal microscope equipped with a 100X objective. Scale bar, 10 µm. **E**) S6K1-X expression was upregulated in a cell-based Huntingtin disease model. HEK293T ectopically expressing either HTT-Q15 (non-disease control) or HTT-Q109 (disease-inducing construct) were analysed by western blot using the indicated antibodies, reporting the extent of proteome HNEylation and endogenous S6K1 and S6K1-X expression levels. Quantification plots are shown on the right. *p* values were calculated using unpaired, two-tailed Student’s *t*-test. All data present mean ± SEM (n = 3). Source data

We examined how NCBP1-HNEylation modulates S6K1-FL and S6K1-X as a function of time and how HNEylated NCBP1 associates with the two S6K1 variants by co-immunoprecipitation. S6K1-X was detected by western blot analysis as early as 2 hours after T-REX-mediated NCBP1-HNEylation. S6K1-X was detected at approximately 45 kDa by an antibody that binds in the N-terminal region (Extended Data Fig. 10a–c). This band was absent in all controls: an antibody that binds S6K1 in the C-terminal region could not detect S6K1-X, although it could detect S6K1-FL. S6K1-X associated with HNEylated NCBP1 more effectively than S6K1-FL (Fig. 5e). However, this association was sensitive to RNAse (Supplementary Fig. 29), indicating that S6K1-X associates with RNA (or another RNA-binding protein). Imaging of HEK293T cells ectopically expressing either S6K1-X or S6K1-FL showed largely cytoplasmic localization (Extended Data Fig. 10d).

### S6K1-X inhibited translation

**Fig. 6:**
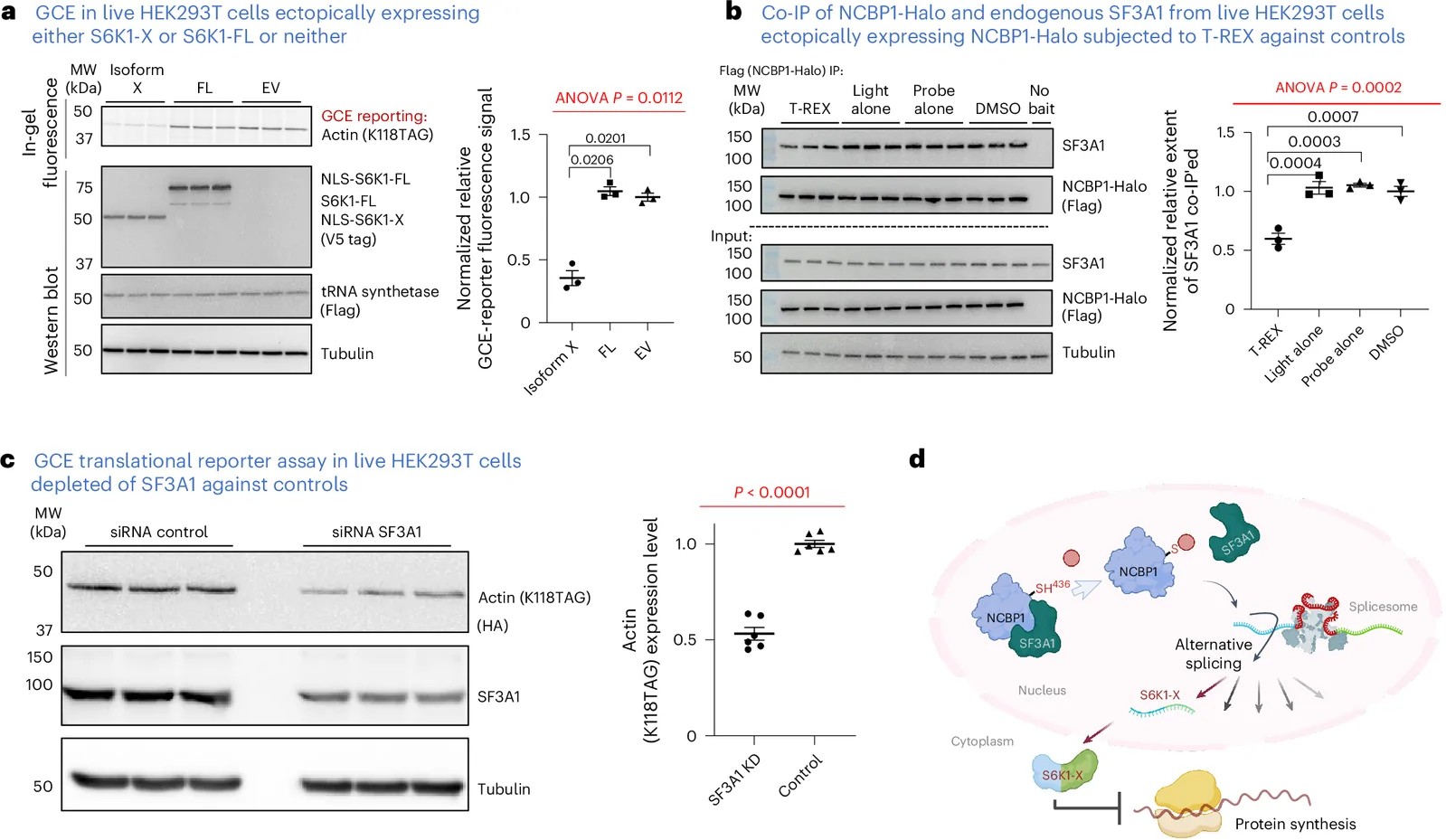
S6K1-X overexpression stalls translation, and SF3A1 is necessary for NCBP1(C436)-HNEylation-dependent S6K1-X formation. **a**, S6K1-X dominantly suppresses translation. HEK293T cells overexpressing S6K1-FL, or S6K1-isoform-X or empty vector (EV), replete with GCER constructs, described elsewhere, were treated with BCNK (50 µM, 4 hours). After lysis, and coupling with tetrazine-Cy5, samples were analyzed by in-gel Cy5 fluorescence reporting protein translation. Anti-V5-tag (detecting S6KL), anti-Flag (tRNA synthetase PylRS) and anti-tubulin (loading control) blots were also performed. Ectopic S6K1 and S6K1-X each have two forms, one with and one without NLS. Thus, the blot shows: NLS-S6K1-FL (74 kDa, also called p85), S6K1-FL (59 kDa), NLS-S6K1-X (51 kDa) and S6K1-X (45 kDa) (Extended Data Fig. 10c). Inset: quantification. *P* values were calculated with Tukey’s multiple comparison test. Data present mean ± s.e.m. (*n* = 3) (Supplementary Fig. 30). **b**, In HEK293T cells, NCBP1 was HNEylated, and then anti-Flag pulldown was performed with results compared to T-REX controls (Methods). Input and elution samples were analyzed by western blot as indicated. Right, quantification of eluted binding partner protein indicated, normalized by NCBP1-Halo expression and tubulin. *P* values were calculated with Tukey’s multiple comparison test. All data present mean ± s.e.m. See Methods for sample size information. **c**, HEK293T cells were co-transfected with GCE(actin) reporter and SF3A1 siRNA (or control siRNA). Thirty-six hours later, cells were treated with BCNK (50 µM, 4 hours). After cell lysis and tetrazine-Cy5-click, samples were analyzed using in-gel fluorescence reporting protein translation efficiency. Western blot was performed using anti-HA (reporting actin(K118TAG)), anti-SF3A1 and anti-tubulin (loading control). *P* values were calculated by an unpaired, two-tailed *t*-test. Data present mean ± s.e.m. (*n* = 6). **d**, NCBP1(C436)-HNEylation induced significant alternative splicing across more than 250 genes (see also Supplementary Table 4 and Fig. 5e). One such event formed S6K1-X. S6K1-X dominantly inhibited translation. Alternative splicing occurs as a result of NCBP1-HNEylation reducing association with SF3A1, a key component of splicesosome. All full-view blots and gels are shown in Source Data Figs. 1–5. IP, immunoprecipitation; KD, knockdown. Source data

Given that the level of S6K1-X produced upon NCBP1-HNEylation was modest (Fig. 5e and Extended Data Fig. 10a,b), we posited that S6K1-X exerted dominant-negative effects on translation. To test this hypothesis, we compared effects of either S6K1-X or S6K1-FL overexpression in HEK293T cells, using cells transfected with an empty vector as control. Consistent with our hypothesis, overexpression of S6K1-X inhibited translation, whereas S6K1-FL had no effect compared to empty vector control (Fig. 6a and Extended Data Fig. 10c). Should the effect of NCBP1-HNEylation be mediated through S6K1-X, increased S6K1-X production by T-REX to NCBP1 in cells already expressing this inhibitory splice variant should have minimal effect on translation. In cells expressing S6K1-FL or empty vector, T-REX-assisted NCBP1-HNEylation caused translation inhibition, as previously observed. However, in cells expressing S6K1-X, NCBP1-HNEylation had no effect (Supplementary Fig. 30). These outcomes are consistent with S6K1-X eliciting a dominant-negative effect on translation, mediated by NCBP1-HNEylation.

### SF3A1-mediated S6K1-X formation

All these data pointed to a mechanistic model where NCBP1(C436)-specific HNEylation in the nucleus triggers a broad-spectrum perturbation of alternative splicing processes, including the formation of S6K1-X, which inhibits translation. S6K1-X’s primarily cytoplasmic location (Extended Data Fig. 10d) and its ability to associate with mRNA (Supplementary Fig. 29) are also consistent with this model. We next explored upstream factor(s) regulating splicing or transcription export modulated by NCBP1-HNEylation. We focused on four known or putative NCBP1 interactors having these functions—namely, ALYREF, PHAX, PRPF4 and SF3A1. The first and second proteins are key transcription export factors for mRNAs and small nuclear RNAs (snRNAs)/small nucleolar RNAs (snoRNAs); the third and fourth are essential splicesome components. T-REX coupled with co-immunoprecipitation assays revealed that NCBP1-HNEylation caused no perturbation of the association between NCBP1 and ALYREF, PHAX or PRPF4 (Supplementary Fig. 31). However, the same process selectively impaired the SF3A1−NCBP1 interaction (Fig. 6b). This result is notable because, unlike ALYREF, PRPF4 and PHAX, no direct evidence of NCBP1’s association with SF3A1 has been demonstrated.

We proposed that NCBP1-HNEylation would reduce interaction with SF3A1, impacting splicing and inhibiting translation. Consistent with this postulate, NCBP1-HNEylation-driven translation suppression was ablated in SF3A1-depleted cells compared to knockdown-control (Supplementary Fig. 32). In SF3A1-deficient HEK293T cells, translation efficiency was reduced compared to knockdown-control (Fig. 6c), a result implying that SF3A1 promotes translation, beyond being important in splicing. SF3A1 silencing is, thus, epistatic with NCBP1-HNEylation in terms of translation suppression.

### S6K1-X is observed in Huntington’s diseased proteomes

To assess the extent to which these findings extend to disease states, we examined models of neurodegeneration that often show elevated levels of protein HNEylation^8,60^. In lines overexpressing Huntington (Htt) disease-causative constructs—namely, Htt Q109 harboring extended polygutamine (poly-Q) mutations—significant levels of endogenous S6K1-X were observed compared to non-diseased control cells overexpressing Htt Q15. As expected, the former diseased cells further manifested enhanced HNEylated proteomes (Extended Data Fig. 10e).

## Discussion

This study presents a hitherto unrecognized mechanism to stall protein synthesis during reactive small-molecule stress in the nucleus. Our data indicate that endogenous electrophilic stress accumulation in the nucleus is predominantly sensed and managed by the large subunit of the CBC, NCBP1, resulting in rapid suppression of translation approximately 2 hours after nuclear-localized electrophile buildup. Such a mechanism provides a new means for stress upregulation in the nucleus to downregulate translation^36,61^. Such a regulatory process is arguably needed because translation is thought to be largely uncoupled from events in the nucleus. We thus establish a clear, coordinated response to nuclear electrophilic stress, distinct from the cytosolic stress response, that martials upregulation of transcription of specific cytoprotective genes. As the nucleus is relatively protected from external reactive small-molecule agents, it is likely that this translation suppression response represents the fact that the buildup of reactive chemicals in the nucleus is mutagenic and potentially highly damaging.

The parallels between this mechanism and translation inhibition enacted by endoplasmic reticulum stress (usually referred to as the unfolded protein response) are not lost on us. In general, endoplasmic reticulum stress responses lead to a general decrease in translation to allow coordinated response to stress. Both also use an unconventional splicing event that helps coordinate the response (S6K1 in the case of nuclear-specific stress sensing and XBP1 during endoplasmic reticulum stress^62^). However, several factors differentiate our mechanism from traditional stress response pathways and highlight the key factors of how substoichiometric electrophile signaling differs from conventional signaling pathways. In our case, NCBP1 is both the initial sensor of electrophile stress and the mediator of S6K1 differential splicing. This mode of the sensor/writer also being the reader is common for reactive small-molecule signaling. In endoplasmic reticulum stress response, a wealth of protein factors martials translational suppression responses. In the case of NCBP1, its electrophile labeling leads to a change in splicing, mediated through association changes with SF3A1, producing a dominant-negative suppressor of translation, S6K1-X.

Although the response mode is particularly intriguing and sets new paradigms on how to martial stress, it shoud be noted that our identified electrophile responder protein is itself enigmatic. Indeed, NCBP1 emerged to be a sensor of electrophilic stress on par with the best sensors that we have characterized. What we did not expect was that none of its cysteines was essential for electrophile sensing (engagement of the electrophile with NCBP1 cysteines); however, only one of its cysteines (C436) was responsible for signaling (changing flux through specific pathways, ushering a phenotype). Given that aberrant translation can play important roles in disease^63–65^, and that we have identified a new electrophile-responsive player linked to this process, these data altogether introduce a new privileged sensor ideal for drug target mining^19,66^. Future work will address this aspect as well as how the other proteins identified in our screen fit into the nuclear electrophile response.

## Methods

### Reagents

HaloTag-targetable photocaged precursor to Ht-PreHNE (no alkyne) and Ht-PreHNE (alkyne) were synthesized as described previously^5^. Tetrazine-Cy5 (CLK-015-05) was from Jena Bioscience; BCNK (sc-8016) was from SiChem; and sulfo-Cy5-azide (B3330), CuTBTA (21050), L-azidohmoalanine (3799), cyanine 5 alkyne (B30B0) and biotin alkyne (C37B0) were from Lumiprobe. Biotin-dEG-azide (PEG4340.0100) was from Iris. Pierce high-capacity streptavidin agarose beads (20361) were from Thermo Fisher Scientific. Anti-Flag M2 affinity gel (A2220), BSA powder and retinoic acid (R2625) were from Sigma-Aldrich. Dithiothreitol (DTT) and tris(2-carboxyethyl)phosphine (TCEP)-HCl were from Gold Biotechnology. Phusion HotStart II polymerase was from Thermo Fisher Scientific. All restriction enzymes were from New England Biolabs (NEB). The plasmid for recombinant expression of TeV protease (Prk793; Addgene, 8827) was from Addgene. N2A and HEK293T cells were from the American Type Culture Collection (ATCC). Protease inhibitor cocktail cOmplete EDTA-free (11873580001) was from Roche. Minimal Essential Media (41090028), DMEM (12491015), Opti-MEM (51985026), Dulbecco’s PBS (DPBS, 14190169), TrypLE Express Enzyme (12605028), 100× pyruvate (100 mM, 11360039), 100× non-essential amino acids (11140035), 100× penicillin−streptomycin (15140122) and NP-40 (85124) were from Life Technologies. FBS was from Sigma-Aldrich (F2442). Light exposure was performed using Spectroline and Camag ultraviolet lamps (Spectroline, ENF-240C, and Camag, lamp 4). The lamps were positioned above a confluent monolayer of cells with power of approximately 5 mW cm^−^^2^ on samples. TransIT-2020 (MIR 5406) was from Mirus Bio. PEI (23966-1) was from Polysciences. Actinomycin D (A9415) was from Sigma-Aldrich. All sterile cell culture plasticware was from TPP or CellTreat. TRIzol reagent (15596018) was from Ambion. Passive lysis buffer for luciferase reporter assay was from Promega. Direct-zol RNA kits were from Zymo Research (R2050). Superscript III (18080085) and RNaseOUT (10777019) enzymes for qPCR experiment were from Thermo Fisher Scientific. SYBR Green Master Mix reagent for RT−qPCR was from Bio-Rad. Sources of antibodies are listed in Supplementary Data 2.

### Plasmid cloning

Ligase-free cloning^16,20^ was used unless specified otherwise. Supplementary Tables 1 and 2 list primers and sequences of localization signals used. All constructs were validated by sequencing.

pPB-MmPylRS-AF-4×PylT_CUA_ and pPB-HA-actin(K118TAG)-4×PylT_CUA_ were gifts from Jason Chin (Medical Research Council Laboratory of Molecular Biology)^12^. psGG_Nrf2-intron^29^ was digested with AflII (NEB) to insert exon 10−intron 10−exon 11 or exon 10−exon 11 and then digested with Xhol (NEB) to insert DACH1 3’ UTR. pCS2+8-NCBP2-HA, pCS2+8-S6K1-FL-V5 and pCS2+8-S6K1-X-V5 were constructed by amplification of NCBP2, S6K1-FL (EPFL Gene Expression Core Facility) and S6K1-X from cDNA after reverse transcription of mRNA isolated from HEK293T cells using primer oligo(dT)20. All primers were from Integrated DNA Technologies.

lentiCRISPRv2 gRNA-1 and gRNA-2 for *DACH1* KO line generation were constructed by insertion of *DACH1* gRNA into lentiCRISPRv2 (Addgene, 52961): *DACH1* gRNA-1, AACCTGGCGGCCGCGAGCAA; *DACH1* gRNA-2, GCCACAGTCACCTCTACCGG; control gRNA, CTTCGAAATGTCCGTTCGGT. *SF3A1* and control small interfering RNAs (siRNAs) were from Dharmacon.

### SDS-PAGE and western blot analysis

SDS-PAGE and blotting were performed as previously described^20^. For primary and secondary antibodies, see Supplementary Table 5.

### Cell culture

HEK293T cells were cultured in MEM + 10% FBS, penicillin−streptomycin, sodium pyruvate and non-essential amino acids. N2A cells were cultured in DMEM plus the same additives at 37 °C in a humidified 5% CO_2_ atmosphere. N2A cells were differentiated with retinoic acid (20 µM, 24 hours) in DMEM + 2% FBS, sodium pyruvate, non-essential amino acids and penicillin−streptomycin. Both cell lines were from ATCC and were validated to be mycoplasma free using the mycoplasma PCR detection kit (Thermo Fisher Scientific).

### Knockout

HEK293T cells in a 10-cm dish at approximately 90% confluence were transfected with 7.5 μg of pCMV-R8.74psPAX2, 750 ng of pCMV-VSV-G and 7.5 μg of plentiCRISPRv2 using TransIT-LT1 (Mirus Bio) following the manufacturer’s protocol. Forty-eight hours after transfection, 10 ml of supernatant was collected and filtered (0.45 µm). Then, 5.8 ml of PBS, 325 μl of 5 M NaCl and 2 ml of PEG-8000 (50% in PBS) were added and incubated overnight (4 °C) with end-over-end rotation. The supernatant was discarded after centrifugation (1,500*g*, 1 hour, 4 °C). Pellet was resuspended in 100 μl of PBS.

HEK293T cells in a six-well plate at approximately 90% confluence were treated with 25 μl of lentiviral particles in 2.5 ml of media, containing 8 μg ml^−1^ polybrene. After 24 hours, media were changed, and cells were incubated for 24 hours. Cells were then incubated in media containing 2 μg ml^−1^ puromycin.

### Localis-REX–GCE in differentiated N2A cells

N2A cells were seeded in a 12-well plate for in-gel fluorescence and transfected with pCS2+8-NLS-Halo, pCS2+8-NES-Halo, pCS2+8-MOMLS-Halo or pCS2+8-ERt-Halo, actin(K118TAG), MmPylRS RNA synthetase (plasmid ratio, 1:1:0.2) and Lipofectamine 3000 reagent as per the manufacturer’s recommendations. For whole proteomic labeling with BCNK, Val-MmPylRS RNA synthetase was used instead of actin(K118TAG) and MmPylRS RNA synthetase (plasmid ratio of Halo:Val-MmPylRS, 1:0.2). Twelve hours later, retinoic acid (20 µM) was added (24 hours) to differentiate N2A cells. Cells were incubated with Ht-PreHNE (10 µM, 2 hours) and rinsed three times. Under Localis-REX conditions, cells were exposed to light (2 minutes, 5 mW cm^−^^2^, 365 nm), the conditions for all the controls were shown below. Cells were incubated with DMSO. Five minutes after light exposure (if used), all cells were incubated with BCNK (60 µM, 4 hours), washed with cold DPBS twice and then frozen in liquid nitrogen.

Localis-REX and T-REX controls are defined as follows:

Light alone: with Ht-PreHNE (in DMSO), with light

Probe alone: with Ht-PreHNE (in DMSO), without light

DMSO: only DMSO, without light

### Localis-REX in differentiated N2A cells for function-guided spatial target ID proteomics analysis

N2A cells were seeded in a 60-mm dish. Twenty-four hours later, approximately 80% confluent cells were co-transfected with pCS2+8-NLS-Halo and Lipofectamine 3000 as per the manufacturer’s recommendations. Twelve hours later, cells were treated with retinoic acid (20 µM, 24 hours) to differentiate N2A cells. Cells were incubated with Ht-PreHNE(alkyne-functionalized) (15 µM, 2 hours) and then rinsed three times. Cells were exposed to light (5 minutes, 5 mW cm^−^^2^, 365 nm). Control experiments were treated identically except that Ht-PreHNE(non-alkyne-functionalized) replaced Ht-PreHNE(alkyne-functionalized). Cells were washed with cold DPBS twice and then frozen in liquid nitrogen.

### BONCAT experiment

HEK293T cells were seeded in 12-well plates (for Cy5-click assay) or 60-mm dishes (for biotin-click pulldown assay). After 24 hours, cells at approximately 80% confluence were transfected with pCS2+8-NLS-Halo, pCS2+8-NCBP1-Halo or pCS2+8-NCBP1(C436A)-Halo as indicated in the corresponding figure legends. TransIT-2020 was used as per the manufacturer’s recommendations. After 36 hours, cells were incubated with Ht-PreHNE(no alkyne) (15 µM, 2 hours) and rinsed three times. During the second wash, the culture medium was changed to DMEM without methionine (Thermo Fisher Scientific, 21013024) and incubated (30 minutes). At the indicated time after light exposure, all experimental groups were incubated with L-AHA (50 µM, 4 hours) in DMEM without methionine. Cells were washed with cold DPBS twice and flash frozen in liquid nitrogen.

### T-REX–GCE in HEK293T cells

HEK293T cells were seeded in 12-well plates. Twenty-four hours later, cells at approximately 80% confluence were co-transfected with pCS2+8-NCBP1-Halo, pCS2+8-Cand1-Halo or pCS2+8-IPO5-Halo, actin(K118TAG) and MmPylRS RNA synthetase (ratio, 1:1:0.2; TransIT-2020). Thirty-six hours later, cells were incubated with Ht-PreHNE(no alkyne) (15 µM, 2 hours) and then rinsed three times. After light exposure, all cells were incubated with BCNK (60 µM, 4 hours). Cells were washed twice with cold DPBS and then frozen in liquid nitrogen.

### T-REX in HEK293T cells

HEK293T cells were seeded in 12-well plates (for in-gel fluorescence/western blot), six-well plates (for RNA-seq) or 60-mm dishes (for Flag pulldown). Twenty-four hours later, cells at approximately 80% confluence were co-transfected with pCS2+8-NCBP1-Halo, pCS2+8-Cand1-Halo or pCS2+8-IPO5-Halo and TransIT-2020. Thirty-six hours later, cells were incubated with Ht-PreHNE(alkyne) (15 µM, 2 hours) and rinsed three times and then exposed to light. At indicated times after light exposure, cells were washed with cold DPBS twice and then frozen in liquid nitrogen.

### Biotin-click and streptavidin pulldown

Cells were harvested following the steps in Localis-REX in differentiated N2A cells for function-guided spatial target ID proteomics analysis or T-REX in HEK293T cells and lysed (50 mM HEPES, 150 mM NaCl (pH 7.6), 1% Nonidet P-40 and 1× Roche cOmplete, mini, EDTA-free protease inhibitor cocktail by freeze−thaw three times). Lysate was centrifuged (20,000*g*, 10 minutes, 4 °C). Protein concentration was normalized (Bradford assay, BSA standard) to 1 mg ml^−1^ using 50 mM HEPES (pH 7.6) and 0.3 mM TCEP. In the T-REX workflow, the samples were subjected to TeV protease (0.2 mg ml^−1^, 30 minutes, 37 °C) as indicated in the figure legends. TeV cleavage was not applicable to Localis-REX. In both workflows, samples were then subjected to click reaction/streptavidin pulldown as described previously^6^.

### Immunofluorescence

N2A cells were seeded in 35-mm glass-bottomed dishes. Twenty-four hours later, cells at approximately 40% conflurence were trasfected with pCS2+8-NLS-Halo, pCS2+8-NES-Halo pCS2+8-MOMLS-Halo or pCS2+8-ERt-Halo and Lipofectamine 3000. After 5 hours, cells were incbuated with retionic acid (20 µM) in DMEM supplemented with 2% FBS (24 hours). Then, cells were treated with Ht-PreHNE (alkyne) (15 µM, 2 hours) and then rinsed three times. Cells were treated with MitoTracker Red (100 nM, 30 minutes) and then rinsed twice. Cells were stained as previously described. Images were taken using a Nikon spinning disk confocal microscope equipped with a ×100 objective.

### Mass spectrometry analysis

#### Sample preparation for liquid chromatography−tandem mass spectrometry

Samples obtained through ‘biotin-click, streptavidin pulldown’ were separated by SDS-PAGE on a 12% polyacrylamide gel and Coomassie blue stained. Each gel lane was sliced, and proteins were digested. The gel pieces were washed twice with 50% ethanol in 50 mM ammonium bicarbonate (AmBic) (20 minutes; Sigma-Aldrich) and dried by vacuum centrifugation. Proteins were reduced (10 mM dithioerythritol; Merck Millipore; 1 hour, 56 °C) followed by a washing−drying step as described above. Proteins were alkylated (55 mM iodoacetamide; Sigma-Aldrich; 45 minutes, 37 °C), followed by a washing−drying step as described above. Proteins were digested overnight at 37 °C using mass spectrometry-grade Trypsin Gold (Promega; 12.5 ng µl^−1^ in 50 mM AmBic supplemented with 10 mM CaCl_2_). Peptides were extracted (70% ethanol and 5% formic acid (Merck Millipore)) twice (20 minutes) and then dried by vacuum centrifugation. Resulting peptides were desalted on StageTips^67^ and dried under a vacuum concentrator.

#### Mass spectrometry

Samples were resuspended in 2% acetonitrile (ACN) (BioSolve) and 0.1% formic acid, and nano-flow separations were performed on a Dionex UltiMate 3000 RSLCnano UPLC system (Thermo Fisher Scientific) online connected with an Orbitrap Exploris 480 Mass Spectrometer or an Orbitrap Lumos Mass Spectrometer (Thermo Fisher Scientific). A capillary precolumn (Acclaim PepMap C18, 3 μm, 100 Å, 2 cm × 75 μm ID) was used for sample trapping and cleaning. A 50-cm-long capillary column (75-μm ID; in-house packed using ReproSil-Pur C18-AQ 1.9-μm silica beads) was then used for analytical separations at 250 nl min^−1^ over 150-minute biphasic gradients. Acquisitions were performed through top speed data-dependent acquisition (DDA). The most intense parent ions were selected and fragmented by higher-energy collision dissociation (HCD) with 30% normalized collision energy (NCE). Selected ions were then excluded for the following 20 seconds.

#### LFQ

Raw data were processed using MaxQuant v.1.6.10.43 (ref. ^68^) against the *Mus musculus* reference proteome database (55,341 protein sequences, Release 2021_02), Halo protein sequence or the *Homo sapiens* reference proteome database (77,027 protein sequences, Release 2021_01). Carbamidomethylation was a fixed modification, whereas methionine oxidation; serine, threonine and tyrosine phosphorylation; N-terminal acetylation; and glutamine to pyroglutamate were considered variable modifications. More than three missed cleavages were allowed, and the ‘Match between runs’ option was enabled. A minimum of two peptides was required for protein identification, and the false discovery rate (FDR) cutoff was 0.01 for both peptides and proteins. LFQ and normalization were performed by MaxQuant using the MaxLFQ algorithm, with standard settings^69^.

Statistical analyses of the label-free data were performed using Perseus v.1.6.15.0 (https://doi.org/10.1038/nmeth.3901) (MaxQuant suite). Reverse proteins, potential contaminants and proteins identified only by sites were filtered out. Protein groups containing at least two valid values in at least one group were conserved for further analysis. Empty values were imputed with random numbers from a normal distribution (width: 0.3; downshift: 1.8). A two-sample *t*-test with permutation-based FDR statistics (250 permutations, FDR = 0.01) was performed to determine significant differentially abundant candidates. Localis-REX-derived LFQ mass spectrometry time-resolved proteomics datasets are summarized in Supplementary Data 1.

### Anti-Flag pulldown

HEK293T cells were seeded in a 60-mm dish; the rest of the steps followed the protocol as detailed in T-REX in HEK293T. At indicated times after light exposure, cells were washed with cold DPBS twice and then frozen in liquid nitrogen. For immunoprecipitation of p-s6k1 bound by NCBP1, 1 hour after T-REX, cells were treated with insulin (100 nM, 1 hour) before harvesting. Cells were lyzed with NET-2 buffer (50 mM Tris-Cl, 100 mM NaCl, 0.1% NP-40 (pH 7.4)) with 1 mM PMSF, 1 mM Na_3_VO_4_, 10 mM NaF, 25 nM calyculin A, 1× Roche protease inhibitor tablet and 100 U ml^−1^ RNAse OUT inhibitor, followed by rapid freeze−thaw cycles (3×). Flag pulldown with anti-Flag M2 affinity agarose gel (Sigma-Aldrich, A2220) was performed as previously described^6^.

### In-gel fluorescence assay

#### For GCE

Cells from one well of a 12-well plate were lysed in 30 µl of buffer containing 50 mM HEPES (pH 7.6), 150 mM NaCl, 1% Nonidet P-40, 1× Roche cOmplete, mini, EDTA-free protease inhibitor cocktail and 0.3 mM TCEP by freeze−thaw (3×). Cells debris was removed by centrifugation (18,000*g*, 8−10 minutes, 4 °C). Lysate concentration was determined using Bradford assay (BSA standard) and diluted to 1 mg ml^−1^ and incubated with tetrazine-Cy5 (1 µM, 30 minutes, 37 °C) and then quenched with 5 µl of 4× Laemmeli dye containing 6% β-mercaptoethanol. After 5-minute incubation at 37 °C, the lysate was subjected to SDS-PAGE. After electrophoresis, the gel was rinsed three times with double-distilled water (5 minutes each) and imaged on a Bio-Rad ChemiDoc MP Imager. Then, the gel was transferred to a PVDF membrane for western blot analysis.

#### For T-REX

All steps in this section were performed under red light unless otherwise stated. In-gel fluorescence assay for T-REX was performed as previously described^6^.

#### For gel-based BONCAT analysis

Cells from one well of a 12-well plate were lysed using freeze−thaw (3×), in 30 µl of 50 mM HEPES (pH 7.6), 150 mM NaCl, 1% Nonidet P-40, 1× Roche cOmplete, mini, EDTA-free protease inhibitor cocktail and 0.3 mM TCEP. Cell debris was removed by centrifugation (18,000*g*, 8−10 minutes, 4 °C). Lysate concentration was determined using Bradford assay (BSA standard) and diluted to 1 mg ml^−1^ and then subjected to biotin-click reaction (final reaction mix: 1.7 mM TCEP, 5% *t*-BuOH, 1% SDS, 1 mM CuSO_4_, 0.1 mM Cu(TBTA) and 10 µM Cy5-alkyne). The samples were incubated (37 °C, 30 minutes) and then quenched with 4× Laemmli dye and 6% β-mercaptoethanol. After 5-minute incubation (37 °C), the lysate was subjected to SDS-PAGE, washed with double-distilled water (3×) and then imaged on a Bio-Rad ChemiDoc MP Imager. Gel was transferred to a PVDF membrane for western blot analysis.

### Luciferase reporter assays

HEK293T cells were transfected with NCBP1-Halo or NCBP1(C436A)-Halo, intron or intron-less firefly luciferase reporter and internal control cytomegalovirus (CMV)-*Renilla* luciferase (in plasmid ratios, 1:1:0.025). Upon T-REX (as shown above), luciferase reporter assays were carried out as previously described^29^.

### RNA-seq

#### Experimental setup and RNA purification

HEK293T cells were seeded in six-well plates for 24 hours. Cells at approximately 80% confluence were co-transfected with pCS2+8-NCBP1-Halo, pCS2+8-Cand1-Halo or pCS2+8-IPO5-Halo, using TransIT-2020 reagent per the manufacturer’s recommendations. Thirty-six hours later, T-REX was performed, followed by RNA purification^29^.

#### RNA-seq analysis

Sample preparation and analyses were performed by the Genomic Technologies Facility at the University of Lausanne. Libraries were prepared from 250 ng of total RNA with TruSeq Stranded mRNA reagents (Illumina). The poly(A) selection step was replaced by a ribosomal RNA depletion step with the QIAseq FastSelect-rRNA HMR Kit (Qiagen). Library preparation was performed on a Sciclone liquid handling robot (PerkinElmer) with a PerkinElmer-developed automated script. Unique dual indexes were used for the barcoding of the libraries. Libraries were quantified (Life Technologies, Qubit), and quality was assessed on a Fragment Analyzer (Agilent Technologies). Sequencing was performed on an Illumina NovaSeq 6000 for 300 cycles (paired-end 150-nucleotide reads). Sequencing data were demultiplexed using bcl2fastq2 Conversion Software (v.2.20, Illumina). T-REX(NCBP1)-mediated RNA-seq datasets are summarized in Supplementary Data 2.

### Differential splicing analysis

Alternative splicing analysis was performed by the Cancer Research UK (CRUK) Cambridge Institute (Ashley Sawle), using SUPPA2 (https://github.com/comprna/SUPPA)^46^. The transcript counts from the raw datasets from RNA-seq analyses (above) were estimated using Salmon^50^, resulting in 1,266 genes to be further analyzed. Counts were normalized to transcripts per million (TPM), prior to further analysis with SUPPA2. Transcript usage and alternative splicing events were measured as PSI—that is, for a given event, the total estimated counts for transcripts including the event were divided by the total estimated counts for all transcripts that could include the event. The list of alternative splicing events with full information (GeneID, PSI of each transcript under each condition, dPSI of difference in the mean PSI between two conditions, each alternative splicing event and its detail and adjusted *P* values) is summarized in Supplementary Data 4 and Table 4. ‘Loss’ and ‘gain’, respectively, indicate splicing events that are less common/lost or more common/gained under the T-REX condition, with respect to the indicated control.

### RT−qPCR

RT−qPCR^29^ and analysis^12^ were carried out as previously described.

### RNA half-life

HEK293T cells expressing NCBP1-Halo were conducted with T-REX and all other three controls and then performed as described. Data were fit to one-phase exponential decay: *y* = (1 − plateau) × exp(−*kx*) + plateau, where *y* is the RNA transcript levels, *k* is the decay constant and *x* is time (GraphPad Prism v.9.4.0).

### Mass spectrometry analysis of HNEylation site

Following the steps described in T-REX in HEK293T cells, cell lysis (3× freeze−thaw) was performed in 500 μl (50 mM HEPES (pH 7.6), 100 mM NaCl, 1% Nonidet P-40, 10 mM imidazole, 5 mM β-mercaptoethanol and Roche protease inhibitor). Lysate was clarified by centrifugation (20,000*g*, 10 minutes, 4 °C). Protein concentration was determined by Bradford assay (BSA standard) and diluted to 1.5 mg ml^−1^ in lysis buffer and incubated with 50 μl of TALON resin (pre-equilibrated with lysis buffer, 1 hour, 4 °C). Resin was washed three times (5 minutes, 500 μl of wash buffer (50 mM HEPES (pH 7.6), 100 mM NaCl, 0.5% Nonidet P-40, 20 mM imidazole and 5 mM β-mercaptoethanol)), and then proteins were eluted with 30 μl of elution buffer (50 mM HEPES, 100 mM NaCl, 200 mM imidazole and 5 mM β-mercaptoethanol). Samples were subjected to SDS-PAGE and stained with colloidal blue. The band of NCBP1-Halo was cut into cubes of approximately 1 × 1 mm and washed (2×) in Milli-Q water and destained in 1:1 v/v 0.1 M AmBic:ACN (30 minutes, 24 °C). Destained gel pieces were dehydrated with ACN and then reduced in 10 mM TCEP (Tris-(2-carboxyethyl)phosphine)/0.1 M AmBic (30 minutes, 24 °C) and alkylated in 50 mM 2-chloroacetamide/0.1 M AmBic (30 minutes, 24 °C). The gel pieces were dehydrated and then digested (16−18 hours, 37 °C) with endoprotease trypsin (Promega) (10 ng µl^−1^ trypsin/10 mM AmBic/10% ACN). Digested peptides were extracted twice from the gel (5% formic acid/ACN (1:2 v/v), 15 minutes, 37 °C) and dried by vacuum centrifugation.

Peptides were separated by UltiMate 3000 RSLCnano liquid chromatography (Thermo Fisher Scientific), coupled in line to a Q Exactive mass spectrometer equipped with an EASY-Spray source (Thermo Fisher Scientific). Peptides were trapped onto a C18 PepMap 100 precolumn (300-µm ID × 5 mm, 100 Å; Thermo Fisher Scientific) using Solvent A (0.1% formic acid in HPLC-grade water). Peptides were further separated onto an EASY-Spray RSLC C18 column (75-µm ID, 50-cm length; Thermo Fisher Scientific) using a 40-minute linear gradient (15% to 38% Solvent B (0.1% formic acid in ACN)), flow rate 200 nl min^−1^. Raw data were acquired in DDA mode. Full-scan mass spectrometry spectra were acquired in the Orbitrap (scan range, 350−1,500 *m*/*z*; resolution, 70,000; AGC target, 3 × 10^6^; maximum injection time, 50 ms). The five most intense peaks were selected for HCD fragmentation at 30% of NCE. HCD spectra were acquired in the Orbitrap at resolution 17,500, AGC target 5 × 10^4^ and maximum injection time 120 ms, with fixed mass at 180 *m*/*z*. Charge exclusion was selected for unassigned and 1+ ions. The dynamic exclusion was set to 5 seconds.

Acquired tandem mass spectrometry (MS/MS) spectra were searched using Sequest HT in Proteome Discoverer (v.1.4), against a custom *H. sapiens* database (Proteome ID UP000005640) downloaded from UniProt (2025_04_29), containing 20,406 protein entries, including 292 common laboratory contaminants and NCBP1-Halo-His sequence. The following variable modifications were considered: carbamidomethylation and HNE modifications (+134.073 Da, 152.084 Da and 154.099 Da) on cysteine residues, in addition to oxidation (M) and acetylation (N-ter). Two missed cleavages were permitted. Peptide mass tolerance was set at 20 ppm on the precursor and at 0.6 Da on the fragment ions. Data were filtered at FDR below 1% at peptide-spectrum match level. Trypsin was set as the endoprotease. All identified HNE-modified MS/MS spectra were manually inspected and validated.

### Reporting summary

Further information on research design is available in the Nature Portfolio Reporting Summary linked to this article.

## Online content

Any methods, additional references, Nature Portfolio reporting summaries, source data, extended data, supplementary information, acknowledgements, peer review information; details of author contributions and competing interests; and statements of data and code availability are available at https://doi.org/10.1038/s41589-025-02135-4.

## Supplementary information

Supplementary Information Supplementary Tables 1−5 and Supplementary Figs. 1−36

Reporting Summary

Supplementary Data 1 LFQ mass spectrometry hits from Localis-REX leveraging nuclear-targeted Halo.

Supplementary Data 2 Timecourse RNA-seq datasets at 3-hour, 6-hour and 12-hour timepoints, after T-REX-assisted NCBP1-HNEylation, against all relevant T-REX controls (light alone, probe alone and DMSO).

Supplementary Data 3 The input list of top-ranked candidate SDE genes and their respective biological functions.

Supplementary Data 4 Timecourse differential splicing analysis: datasets at 3-hour, 6-hour and 12-hour timepoints, after T-REX-assisted NCBP1-HNEylation, against all relevant T-REX controls (light alone, probe alone and DMSO).

## Source data

Source Data Figs. 1–6 Unprocessed western blots and/or gels

Source Data Extended Data Figs. 2, 4−7 and 10 Unprocessed western blots and/or gels

Source Data Figs. 1–6 and Extended Data Figs. 2–10 Statistical source data

## Data Availability

Proteomics data that support the findings of this study (Figs. 1d and 2b,c and Supplementary Data 1) have been deposited to the ProteomeXchange Consortium via the PRIDE partner repository with dataset identifier PXD:051483. The RNA-seq raw data from this study used to generate Figs. 4a,e and 5a, Extended Data Fig. 9, Supplementary Figs. 18–21, Supplementary Data 2–4 and Tables 3 and 4 have been deposited in the Gene Expression Omnibus database under accession code GSE:265951. Source data are provided with this paper.
