# Supplementary material for: NCBP1 stress signaling drives alternative S6K1 splicing inhibiting translation
Source: Nat Chem Biol. 2026 Feb 10;22(10):1628–41. doi: 10.1038/s41589-025-02135-4 (PMC13612227; doi:10.1038/s41589-025-02135-4)
Supplement: Supplementary file 1 [file 41589_2025_2135_MOESM1_ESM.pdf]

nature chemical biology

Supplementary information

https://doi.org/10.1038/s41589-025-02135-4

NCBP1 stress signaling drives alternative S6K1 splicing inhibiting translation

In the format provided by the authors and unedited

SUPPLEMENTARY INFORMATION

Table of Content

Supplementary Tables 1-5 and Data 1-4	pp 1- 20
Supplementary Figures 1-36	pp 21-53

Supplementary Tables

Supplementary Table 1. Primers used for cloning.

plasmid	Primer	Sequence (5'→3')
pCS2+8 -NLS-Halo	Forward primer1	ATGCCAAAAAAGAAGCGCAAGGTATCGGGTCCTAAGAAGAAGCGTAAAGTGTCAGG
	Reverse primer1	GGTTGATTATCGATAAGCTTGATATCGAATTCTTAGCCGGAAATCTCGAGCGTC
	Forward primer2	TTTCTTCCATTTCAGGTGTCGTGAGGATCCGCCACCATGCCAAAAAAGAAGCGCAAGG
	Reverse primer2	GGTTGATTATCGATAAGCTTGATATCGAATTCT
pCS2+8 -NES-Halo	Forward primer1	GAGCTTGCCCTAAAGCTAGCAGGCCTCGATCTATCTGGAATGGCAGAAATCGGTACTGGC
	Forward primer2	TCGCACTCAAACTCGCCGGGCTAGACCTGTCAGGGGAAGAGCTTGCCCTAAAGCTAGCAG
	Forward primer3	TCCATTTCAGGTGTCGTGAGGATCCGCCACCATGGAGGAACTCGCACTCAAACTCGCCG
	Forward primer4	TTCTTCCATTTCAGGTGTCGTGAGGATC
	Reverse primer1	GGTTGATTATCGATAAGCTTGATATCGAATTCTTAGCCGGAAATCTCGAGCGTC
	Reverse primer2	GGTTGATTATCGATAAGCTTGATATCGAATTCT
pCS2+8 -MOMLS-Halo	Forward primer1	TTTCTTCCATTTCAGGTGTCGTGAGGATCCGCCACCATGAAGAGCTTCATTACAAGG
	Reverse primer1	GGTTGATTATCGATAAGCTTGATATCGAATTCTTAGCCGGAAATCTCGAGCGTC
	Forward primer2	TTTCTTCCATTTCAGGTGTCGTGAGGATCCGCCACCATGCCAAAAAAGAAGCGCAAGG
	Reverse primer2	GGTTGATTATCGATAAGCTTGATATCGAATTCT
pCS2+8 -ERt-Halo	Forward primer1	AAGCTGATCATCGATCAGAACGTTTTTATC
	Reverse primer1	GGTTGATTATCGATAAGCTTGATATCGAATTCTTATCACTCCTGCGCGATGCT
	Forward primer2	TTTCTTCCATTTCAGGTGTCGTGAGGATCCGCCACCATGCCAAAAAAGAAGCGCAAGG
	Reverse primer2	GGTTGATTATCGATAAGCTTGATATCGAATTCT
	Forward primer1	TAATTAAAGGCCGGCCAGCGATCGCCGGACCCACCATGTCGCGGCGGCGG

pCS2+8-NCBP1-Flag-TEV-Halo	Reverse primer1	CCTGACTTGTCGTCATCGTCTTTATAATCCATGGCCTGCAGGGCACAGAA
	Forward primer2	ACTTGTTCTTTTTGCAGGATCCACTAGTGGCGCGCCATTAATTAAAGGCCGGCCAGCGA
	Reverse primer2	TCCCTGAGCCCTGGAAATACAAGTTTTCCCCTGACTTGTCGTCATCGTCTTTATAATCC
pCS2+8-Cand1-Flag-TEV-Halo	Forward primer1	TAATTAAAGGCCGGCCAGCGATCGCCGGACCCACCATGGCGAGCGCCTCGTAC
	Reverse primer1	CTGACTTGTCGTCATCGTCTTTATAATCCATACTAGTGTCCATTGATTCCAAGTTAGTAG
	Forward primer2	Same with Forward primer2 of pCS2+8-NCBP1-Flag-TEV-Halo
	Reverse primer2	Same with Reverse primer2 of pCS2+8-NCBP1-Flag-TEV-Halo
pCS2+8-IPO5-Flag-TEV-Halo	Forward primer1	TTAAAGGCCGGCCAGCGATCGCCGGACCCACCATGGCGGCGG
	Reverse primer1	CCTGATCCTGACCCGCTTCCGGATCCCGCAGAGTTCAGGAGCTCCT
	Forward primer2	Same with Forward primer2 of pCS2+8-NCBP1-Flag-TEV-Halo
	Reverse primer2	Same with Reverse primer2 of pCS2+8-NCBP1-Flag-TEV-Halo
pCS2+8-NCBP1 C380A-Flag-TEV-Halo	Forward primer	AGAGCCAGGTTGAAGTTTGGCCAGTTCAATGAGGAGTGTT
	Reverse primer	AACACTCCTCATTGAACTGGCCAAACTTCAACCTGGCTCT
pCS2+8-NCBP1 C332A-Flag-TEV-Halo	Forward primer	AACTGTGCAGCAGCAGTCTTCCTTTCCTTCCAGTGGG
	Reverse primer	CCCACTGGAAGGAAAGGAAGACTGCTGCTGCACAGTT
pCS2+8-NCBP1 C456A-Flag-TEV-Halo	Forward primer	GGTAAGACAACCTCATAGCTTTTTCTAGAACTTCTCTTACAAACTTCGG
	Reverse primer	CCGAAGTTTGTAAGAGAAGTTCTAGAAAAAGCTATGAGGTTGTCTTACC
pCS2+8-NCBP1 C616A-Flag-TEV-Halo	Forward primer	ATTTGCTACGGCAGCAGCATCAACTATTTGTGTACGAATCATCTTATCC
	Reverse primer	GGATAAGATGATTCGTACACAAATAGTTGATGCTGCTGCCGTAGCAAAT
pCS2+8-NCBP1 C728A-Flag-TEV-Halo	Forward primer	GTCCCATCAGTTTCGGCTCGTACTAGGTGCTCGGTC
	Reverse primer	GACCGAGCACCTAGTACGAGCCGAAACTGATGGGAC
pCS2+8-NCBP1 C786A-Flag-TEV-Halo	Forward primer	CGGCCTGCAGGGCAGCGAACTGCTGGAACA
	Reverse primer	TGTTCCAGCAGTTCGCTGCCCTGCAGGCCG
	Forward primer	AGGTTGCTCTCCAAAGAGGCGGCACTCTTTTCTCCTAC

pCS2+8-NCBP1 C44A-Flag-TEV-Halo	Reverse primer	GTAGGAGAAAAGAGTGCCGCCTCTTTGGAGAGCAACCT
pCS2+8-NCBP1 C232A-Flag-TEV-Halo	Forward primer	ATCTGGGCCCACAGGGCATCTAAATACTCTTCTTGTGGATGT
	Reverse primer	ACATCCACAAGAAGAGTATTTAGATGCCCTGTGGGCCCAGAT
pCS2+8-NCBP1 C261A-Flag-TEV-Halo	Forward primer	TGTGCTGCAGTGCTTCAGCCAGGATGCTGTCAAAGG
	Reverse primer	CCTTTGACAGCATCCTGGCTGAAGCACTGCAGCACA
pCS2+8-NCBP1 C320A-Flag-TEV-Halo	Forward primer	CTTCCAGTGGGACTTAATGATGGCGTGAAGATTCTCTTCTATTACA
	Reverse primer	TGTAATAGAAGAGAATCTTCACGCCATCATTAAGTCCCACTGGAAG
pCS2+8-NCBP1 C483A-Flag-TEV-Halo	Forward primer	ATCTCCATACTTGTAAATGGCGGTTGGGTTTGCAGGACAC
	Reverse primer	GTGTCCTGCAAACCCAACCGCCATTTACAAGTATGGAGAT
pCS2+8-NCBP1 C503A-Flag-TEV-Halo	Forward primer	TTTTAAAGGCAACAGCTAAAGCGAGGGCAACAGAATGTCCAG
	Reverse primer	CTGGACATTCTGTTGCCCTCGCTTTAGCTGTTGCCTTTAAAA
pCS2+8-NCBP1 C36A-Flag-TEV-Halo	Forward primer	GCACTCTTTTCTCCTACTTTAGCTATTAAAGATTCCAAATGATCTTCAGTTTCAT
	Reverse primer	ATGAAACTGAAGATCATTTGGAATCTTTAATAGCTAAAGTAGGAGAAAAGAGTGC
pCS2+8-NCBP1 C73A-Flag-TEV-Halo	Forward primer	AGGCGTGCAACTGTAGCAAGAAGCCTTAAGATCTTGCTCT
	Reverse primer	AGAGCAAGATCTTAAGGCTTCTTGCTACAGTTGCACGCCT
pCS2+8-NCBP1 C137A-Flag-TEV-Halo	Forward primer	GCGGCAATCACATGAGCATTCACAAGATCAGATAAAAAACGGACCA
	Reverse primer	TGGTCCGTTTTTTATCTGATCTTGTGAATGCTCATGTGATTGCCGC
pCS2+8-NCBP1 C409A-Flag-TEV-Halo	Forward primer	ACCAATTAATAAACCTGTCTACAGCGGTAGTGTTCATTGTGTCCAAAC
	Reverse primer	GTTTGGACACAATGAACACTACCGCTGTAGACAGGTTTATTAATTGGT
pCS2+8-NCBP1 C436A-Flag-TEV-Halo	Forward primer	CAGGATCTTGACTAAGAGCATCTGACCAATCTTCCCAGCT
	Reverse primer	AGCTGGGAAGATTGGTCAGATGCTCTTAGTCAAGATCCTG
	Forward primer	CTGCTGCAGCCTCTCTATAGCGTTCTTATACCATGGTGTT

pCS2+8-NCBP1 C743A-Flag-TEV-Halo	Reverse primer	AACACCATGGTATAAGAACGCTATAGAGAGGCTGCAGCAG
pCS2+8-NCBP1 C477A-Flag-TEV-Halo	Forward primer	AGGTTGGGTTTGCAGGAGCCAGAGCTGAGAAGGTAG
	Reverse primer	CTACCTTCTCAGCTCTGGCTCCTGCAAACCCAACCT
psGG_10-11-DACH1-3UTR and psGG_10-11No intron-DACH1-3UTR	Forward primer1	CTGTTGGTAAAGCCACCATGATAGTTCAAAAGAGGCTAAA
	Reverse primer1	TTTAGCCTCTTTTGAACTATCATGGTGGCTTTACCAACAG
	Forward primer2	TTGGCATCTTCCATTTGTATTGTCCTTTCAGCATCTGTTC
	Reverse primer2	GAACAGATGCTGAAAGGACAATACAAATGGAAGATGCCAA
	3’UTR_Forward primer1	TCAACGTCTAAGGCCGCGACTCTAGAATCTTTCCTGTTGAAGAAATCCATGTTATAGA
	3’UTR_Reverse primer1	TCTGCTCGAAGCGGCCGGCCCTCGAGGAGATTTCAGACAGTTTTATTAGGAGATTGTTG
	3’UTR_Forward primer2	GGCATGGATAGACACCCTGCTGCTTGCGCCAGCGCCAGGATCAACGTCTAAGGCCGCGA
	3’UTR_Reverse primer2	TAGTTGTGGTTTGTCCAAACTCATCAATGTATCTTATCATGTCTGCTCGAAGCGGCCG
pCS2+8-NCBP2-HA	Forward primer1	ATGTCGGGTGGCCTCCTG
	Forward primer2	TGACACTATAGAATACAAGCTACTTGTTCTTTTTGCCACCATGTCGGGTGGCCTCCTG
	Forward primer3	CGTCGGAGCAAGCTTGATTTAGGTGACACTATAGAATACAAGCTACTTGTTCTTTTTG
	Reverse primer1	CTGGTTCTGTGCCAGTTTTCCATAG
	Reverse primer2	AGGCTCGAGAGGCCTTGAATTCGATTATTACTGGTTCTGTGCCAGTTTTCCATAG
	Reverse primer3	GTCTGGATCTACGTAATACGACTCACTATAGTTCTAGAGGCTCGAGAGGCCTTGAATT
	Forward primer1	GGCGTTCCATTGACGTAAATGGG
	Reverse primer1	CATGCTCCCTGATCCTGACCCGCTTCCGGATCCCTGGTTCTGTGCCAGTTTTCCATAG

pCS2+8-NCBP2-Flag-TEV-Halo		
	Reverse primer2	TTTTCCCCTGACTTGTCGTCATCGTCTTTATAATCCATGCTCCCTGATCCTGACCC
pCS2+8-S6K1-FL-(V5)	Forward primer1	CCTATCCGTACGACGTACCAGACTACGCAGGATCCGGAAGCATGAGGCGACGAAGGAGGC
	Forward primer2	ATTAATTAAAGGCCGGCCAGCGATCGCCGGACCCACCTATCCGTACGACGTACCAGACT
	Forward primer3	ACTTGTTCTTTTTGCAGGATCCACTAGTGGCGCGCCATTAATTAAAGGCCGGCCAGCG
	Reverse primer1	TAGATTCATACGCAGGTGCTCTGG
	Reverse primer2	TCTAGAGGCTCGAGAGGCCTTGAATTCGATTATTATAGATTCATACGCAGGTGCTCTGG
	Reverse primer3	CTTATCATGTCTGGATCTACGTAATACGACTCACTATAGTTCTAGAGGCTCGAGAGGCC
pCS2+8-S6K1-X-(V5)	Forward primer1	Same as Forward primer1 of pCS2+8-S6K1-FL-(V5)
	Forward primer2	Same as Forward primer2 of pCS2+8-S6K1-FL-(V5)
	Forward primer3	Same as Forward primer3 of pCS2+8-S6K1-FL-(V5)
	Reverse primer1	GATTGACAGGTAGAACACCATGTGA
	Reverse primer2	CTAGAGGCTCGAGAGGCCTTGAATTCGATTATTAGATTGACAGGTAGAACACCATGTGA
	Reverse primer3	TTATCATGTCTGGATCTACGTAATACGACTCACTATAGTTCTAGAGGCTCGAGAGGCCT
pCS2+8-NCBP1 C436A-HA	Forward primer1	TAATTAAAGGCCGGCCAGCGATCGCCGGACCCACCATGTCGCGGCGGCGG
	Reverse primer1	TATGCGTAGTCTGGTACGTCGTACGGATAGCTTCCGGATCCGGCCTGCAGGGCACAGAA
	Forward primer2	ACTTGTTCTTTTTGCAGGATCCACTAGTGGCGCGCCATTAATTAAAGGCCGGCCAGCGA
	Reverse primer2	TCTAGAGGCTCGAGAGGCCTTGAATTCGATTATTATGCGTAGTCTGGTACGTCGTACG

pCS2+8-HTT Q15-HA and pCS2+8-HTT Q109-HA	Forward primer1	AAGGCCGGCCAGCGATCGCCGGACCCACCATGGCGACCCTGGAAAAGCT
	Reverse primer1	CGTAGAATCGAGACCGAGGAGAGGGTTAGGGATAGGCTTACCTCGGTGCAGCGGCTCC
	Forward primer2	GTTCTTTTTGCAGGATCCACTAGTGGCGCGCCATTAATTAAAGGCCGGCCAGCGA
	Reverse primer2	TCTAGAGGCTCGAGAGGCCTTGAATTCGATTATTACGTAGAATCGAGACCGAGGAGAG
pCS2+8-XPO1-Flag-TEV-Halo	Forward primer1	AAAGGCCGGCCAGCGATCGCCGGACCCACCATGCCAGCAATTATGACAATGTTAGCA
	Reverse primer1	CCCTGATCCTGACCCGCTTCCGGATCCATCACACATTTCTTCTGGAATCTCATGTG
	Forward primer2	GCAGGATCCACTAGTGGCGCGCCATTAATTAAAGGCCGGCCAGCGAT
	Reverse primer2	CCTGACTTGTCGTCATCGTCTTTATAATCCATGCTCCCTGATCCTGACCCGCTTC
pCS2+8-RAN-Flag-TEV-Halo	Forward primer1	AAAGGCCGGCCAGCGATCGCCGGACCCACCATGGCTGCGCAGGGAGAG
	Reverse primer1	CCCTGATCCTGACCCGCTTCCGGATCCCAGGTCATCATCCTCATCCGGG
	Forward primer2	GCAGGATCCACTAGTGGCGCGCCATTAATTAAAGGCCGGCCAGCGAT
	Reverse primer2	CCTGACTTGTCGTCATCGTCTTTATAATCCATGCTCCCTGATCCTGACCCGCTTC
pPB-MmPylRS-AF-4xPylT$_{CAC}$	PT Overlap R	GTCCGTTCGATCTACATGATCAGGTTTC
	PT Overlap CAC Val F	ATCATGTAGATCGAACGGACTCACAATCCGTTCAGCCG
	PT1 FW	AATAAGGTCTCAGCATCTAGGGCAGGAAGAGGGCCTATTTC
	PT1 RV	AATAAGGTCTCACCATTATGGGCAGGAAGAGGGCCTATTTC
	PT2 FW	AATAAGGTCTCACCATTATGGGCAGGAAGAGGGCCTATTTC
	PT2 RV	AATAAGGTCTCAACTAGAGAAAAACCGCACTTGTCCGGAAA
	PT3 FW	AATAAGGTCTCATAGTTGCGGGCAGGAAGAGGGCCTATTTC
	PT3 RV	AATAAGGTCTCAGATCCATAAAAACCGCACTTGTCCGGAAA
	PT4 FW	AATAAGGTCTCAGATCATGGGGCAGGAAGAGGGCCTATTTC
	PT4 RV	AATAAGGTCTCAATTGATTAAAAACCGCACTTGTCCGGAAA

Supplementary Table 2. Localization sequences used for HaloTag locale in Localis-REX.

Flag-NLS (at N terminal of Halo)	GATTACAAGGATGACGACGATAAGATGGAGGAACTCGCACTCAAACTCGCCGGG CTAGACCTGTCAGGGGAAGAGCTTGCCCTAAAGCTAGCAGGCCTCGATCTATCTG GA
Flag-NES (at N terminal of Halo)	GATTACAAGGATGACGACGATAAGATGGAGGAACTCGCACTCAAACTCGCCGGG CTAGACCTGTCAGGGGAAGAGCTTGCCCTAAAGCTAGCAGGCCTCGATCTATCTG GA
MOMLS-Flag (at N terminal of Halo)	ATGAAGAGCTTCATTACAAGGAACAAGACAGCCATTTTGGCAACCGTTGCTGCTAC AGGTACTGCCATCGGTGCCTACTATTATTACAACCAATTGCAACAGGATCCACCGG TCGCCACCGATTACAAGGATGACGACGATAAGATG
ERt-V5 tag-Flag (at C terminal of Halo)	GATTATAAAGATGATGATGATAAAGGTAAGCCTATCCCTAACCCTCTCCTCGGTCT CGATTCTACGAGATCTCTCCTGGGTGGCGCCCTGGCGAACTTGTTTGTGATAGTTG GGTTTGCAGCCTTTGCTTACACGGTCAAGTACGTGCTGAGGAGCATCGCGCAGGA G

Supplementary Data 1. LFQ-MS hits from Localis-REX leveraging nuclear-targeted Halo.

Please see the enclosed excel file labeled '**Supplementary Data 1 (Localis-REX_proteomics-hits)'**.

[Note: The 6 protein hits shown in Main Figure 1D, constitute top-ranked hits consistently captured across 3 independent biological replicate-runs, whereas entries in Supplemental Table S3 are ranked in the order of highest-to-lowest 'average' LFQ scores. Thus, for instance, protein such as Tln1 does not appear within the list in Figure 1D, since its LFQ score in the second replicate experiment is only 6.72, although the average score (7.68) is high].

Supplementary Data 2. Time-course RNA-Seq datasets at 3, 6, and 12 h time-points, following T-REX-assisted NCBP1-HNEylation, against all relevant T-REX controls (light alone, probe alone, and DMSO).

Please see the enclosed excel file labeled '**Supplementary Data 2 (T-REX_NCBP1_RNASeq-hits)'**.

[Note: Within each tab of this excel file, hit transcripts with adj p values < 0.05, are highlighted in red].

Supplementary Data 3. The input list of top-ranked candidate SDEs and their respective biological functions.

Please see the enclosed excel file labeled '**Supplementary Data 3 (SDEs_STRING_gProfiler)'**.

Supplementary Table 3. RT-qPCR primers

gene	Primer set	Sequence (5'→3')
**DACH1 mRNA**	Forward primer	CAATGACTGCACCAACGCAA
	Reverse primer	GGAAGTTCCAGTCCGACACT
**DACH1 Pre-mRNA**	Forward primer	GTCACCATTTTAAACTTTATTAGGGTTCAGC
	Reverse primer	ATATGTGGCATCAAAGAGAGGAAATTTATATCA
**Actin (K118TAG)**	Forward primer	CCAGACTACGCAGGCTTCGA
	Reverse primer	AATCCTTCTGACCCATGCCCA
**POUSF2**	Forward primer	GCTCTTGGCCACTCTCCATT
	Reverse primer	TTGCCTTCGATAAAGCGGGT
**SNRPD3**	Forward primer	GTAGGCCAGAGCCGAACTC
	Reverse primer	TCCCCCGATATACCTCACCG
**S6K1**	Forward primer	CTGGAGCACCCCCATTCACT
	Reverse primer_E11_12	GATGAGCTTGAACTTCTCCAGCG
	Reverse primer_E13_14	CCACATATGTAAAACCCAGAAAGACCTG
**THOC2 isoform2**	Forward primer	GTGACTGACGATATAGCCAGTACCA
	Reverse primer	GCCAAAGCATCATATTCTTGGCGA
**THOC7 Isoform2 excluded**	Forward primer	TGGGAGCCGTGACTGACG
	Reverse primer	GCTCCAGCTATGCTACATTCTATTTCC
**MYBL2 Isoform2**	Forward primer	CCATGAGGAGAACCGCACTGA
	Reverse primer	GTGCCAGCGTTCACGGC
**MYBL2 Isoform2 excluded**	Forward primer	CCAGCCACTTCCCTAACCG
	Reverse primer	CAGTGTCCACTGCTTTGTGC
**GAPDH**	Forward primer	GACAGTCAGCCGCATCTTCT
	Reverse primer	AAATGAGCCCCAGCCTTCTC

Supplementary Data 4. Time-course differential splicing analysis: datasets at 3, 6, and 12 h time-points, following T-REX-assisted NCBP1-HNEylation, against all relevant T-REX controls (light alone, probe alone, and DMSO).

Please see the enclosed excel file labeled '**Supplementary Data 4(differential splicing analysis)'**. Events at each time point were deemed significant if they appear at least twice (against 3 independent biological replicates) across the 3 comparisons, namely, T-REX vs. light alone control, T-REX vs. probe alone control, and T-REX vs. DMSO vehicle control. The tables shown are already filtered for adjusted p-value <0.05 (adjusted using the Benjamin-Hochberg method).

Supplementary Table 4. Schematic list of genes that altogether undergo statistically-significant alternative splicing (AS) events at either 3 or 6 h following NCBP1-HNEylation (data from Supplementary Data 4: differential splicing analysis), against their respective statistically significant (SS) differential expression (DE) changes following NCBP1-HNEylation (data from Supplementary Data 2: T-REX_NCBP1_RNA-Seq-hits)

**Gene name**	**AS event type**	**AS direction post T-REX** (against corresponding controls indicated in Supp. Data 4)	**Time (h) post T-REX**	**Do these genes show differential expression (DE)?** (note: unless otherwise indicated, all SS DE genes below were found only in the 12 h post T-REX against DMSO control set)
CCDC124	AF	Loss	3	N
ABCA13	A5	Loss	3	N
ABCA13	SE	Loss	3	N
ACAT1	AF	Gain	3	N
ACAT1	AF	Gain	3	N
ACAT1	AF	Gain	3	N
ACAT1	SE	Loss	3	N
ANKRD16	SE	Gain	3	N
ARRDC2	AF	Gain	3	N
ATF4	A3	Gain	3	Upregulation, (12h, T-REX against Light alone control), Upregulation, (12h, T-REX against DMSO
ATF4	A3	Gain	3	Upregulation, (12h, T-REX against Light alone control), Upregulation, (12h, T-REX against DMSO
ATF4	A5	Loss	3	Upregulation, (12h, T-REX against Light alone control), Upregulation, (12h, T-REX against DMSO
ATF4	SE	Loss	3	Upregulation, (12h, T-REX against Light alone control), Upregulation, (12h, T-REX against DMSO
ATRAID	A5	Gain	3	N
BMPR1A	A3	Gain	3	N
BOLA1	A3	Loss	3	N
C1orf122	A5	Gain	3	N
C20orf27	SE	Gain	3	N
CCDC124	AF	Loss	3	N
CCT4	SE	Gain	3	Y (Downregulation)
CD83	AF	Loss	3	N
CDKN2A	AF	Loss	3	Y (Downregulation)

CHCHD3	AF	Gain	3	N
CKB	RI	Loss	3	N
COPB1	AF	Loss	3	N
COPB1	AL	Gain	3	N
COPB1	AL	Gain	3	N
COPB1	SE	Loss	3	N
COTL1	SE	Gain	3	Y (Downregulation)
COTL1	SE	Gain	3	Y (Downregulation)
COX6A1	SE	Loss	3	N
CRIPT	AF	Loss	3	N
DDX5	A5	Gain	3	N
DDX5	AF	Gain	3	N
DDX5	AF	Gain	3	N
DDX5	AF	Gain	3	N
DDX5	AF	Gain	3	N
DDX5	AF	Gain	3	N
DDX5	AF	Gain	3	N
DDX5	SE	Gain	3	N
DIDO1	A5	Loss	3	N
DIDO1	AF	Loss	3	N
DNTTIP1	SE	Gain	3	N
E2F3	A3	Gain	3	Y (Downregulation)
E2F3	AF	Gain	3	Y (Downregulation)
EIF4EBP1	AF	Loss	3	Upregulation, (12h, T-REX against Light alone control), Upregulation, (12h, T-REX against DMSO
EIF4EBP1	AF	Gain	3	Upregulation, (12h, T-REX against Light alone control), Upregulation, (12h, T-REX against DMSO
EMC6	A5	Loss	3	Y (Downregulation)
FAM168B	A5	Gain	3	N
FAM3C	A5	Loss	3	N
FLYWCH2	A5	Loss	3	N
FOXO3	AF	Loss	3	N
GAR1	AF	Loss	3	Y (Downregulation)
GAR1	AF	Loss	3	Y (Downregulation)
GTF2E2	AF	Gain	3	N
HCFC1	A3	Loss	3	N
HCFC1	A3	Loss	3	N
HLA-C	A3	Loss	3	N
INPPL1	A3	Gain	3	N
INPPL1	SE	Loss	3	N
ISCA2	A3	Gain	3	N
ISCA2	SE	Gain	3	N
KEAP1	AF	Loss	3	N
KEAP1	AF	Loss	3	N
LIMK1	A3	Gain	3	N
LIN7C	SE	Gain	3	Y (Downregulation)

LRP3	AF	Gain	3	N
MAP4K5	A5	Gain	3	N
MAP4K5	A3	Gain	3	N
MARCHF6	AF	Loss	3	N
MARCHF6	AF	Loss	3	N
MARCHF6	AF	Loss	3	N
MARCHF6	AF	Gain	3	N
MARCHF6	AF	Gain	3	N
MBD3	A3	Gain	3	N
MEA1	AF	Loss	3	Downregulation, (12h, T-REX against probe alone control), Downregulation, (12h, T-REX against DMSO)
MEA1	A5	Loss	3	Downregulation, (12h, T-REX against probe alone control), Downregulation, (12h, T-REX against DMSO)
MEA1	AF	Gain	3	Downregulation, (12h, T-REX against probe alone control), Downregulation, (12h, T-REX against DMSO)
MEX3C	AF	Loss	3	N
MGARP	A5	Gain	3	N
MGARP	AF	Gain	3	N
MGARP	SE	Gain	3	N
MGAT4C	A3	Gain	3	N
MGAT4C	A3	Gain	3	N
MIEN1	A5	Loss	3	N
MRPL13	A5	Loss	3	N
MRPL23	AF	Loss	3	N
MRPL23	AF	Loss	3	N
MRPL23	RI	Gain	3	N
MRPL3	A5	Gain	3	Y (Downregulation)
MRPL3	A5	Loss	3	Y (Downregulation)
MYBL2	SE	Gain	3	N
NA	A5	Gain	3	N
NDUFAF3	AF	Gain	3	N
NDUFAF3	AF	Loss	3	N
NDUFAF3	AF	Loss	3	N
NKAIN3	SE	Gain	3	N
NKAIN3	SE	Loss	3	N
PANK1	AF	Loss	3	N
PAPPA	AL	Loss	3	N
PAPPA	SE	Gain	3	N
PCOTH	AF	Loss	3	N
PKDCC	A3	Loss	3	N
PKMYT1	AL	Loss	3	N
PKMYT1	A3	Gain	3	N
PKN1	AF	Gain	3	N
PLEKHG4B	SE	Loss	3	N
PRKCSH	A5	Loss	3	N

PRKCSH	A5	Loss	3	N
PRKCSH	AL	Loss	3	N
PRNP	A3	Gain	3	N
PSMD3	SE	Loss	3	N
PTMS	AF	Loss	3	N
PTMS	AF	Loss	3	N
PTMS	AF	Loss	3	N
PTP4A1	AF	Loss	3	N
PTP4A1	AF	Loss	3	N
PTP4A1	AF	Loss	3	N
PTTG1IP	SE	Gain	3	Y (Downregulation)
RAB4A	A3	Gain	3	N
RFX7	SE	Gain	3	N
RNPS1	AF	Loss	3	N
RNPS1	AF	Loss	3	N
RNPS1	AF	Loss	3	N
RPA2	A5	Gain	3	N
RPA2	AF	Gain	3	N
RPA2	AF	Gain	3	N
RPL29	A5	Gain	3	N
RPL29	A5	Gain	3	N
RPL29	A5	Gain	3	N
RPL29	A5	Gain	3	N
RPL29	AF	Loss	3	N
RPL29	AF	Loss	3	N
RPL29	AF	Gain	3	N
RPL32	A3	Loss	3	N
RPL4	AL	Gain	3	N
RPL9	A3	Gain	3	N
RPL9	A5	Gain	3	N
RPL9	AF	Gain	3	N
RPL9	SE	Gain	3	N
RPL9	SE	Gain	3	N
RPL9	SE	Gain	3	N
RPS6	A5	Loss	3	N
RSPH6A	A3	Gain	3	N
SF3B2	SE	Loss	3	Y (Upregulation)
SIGIRR	A5	Gain	3	N
SLC16A1	AF	Gain	3	Y (Downregulation)
SLC16A1	AF	Gain	3	Y (Downregulation)
SLC25A3	A5	Loss	3	Y (Downregulation)
SLC7A3	A3	Gain	3	N
SLC7A5	AF	Gain	3	Y (Upregulation)
SLC7A5	AF	Loss	3	Y (Upregulation)
SMNDC1	AF	Gain	3	Y (Downregulation)
SMNDC1	AF	Gain	3	Y (Downregulation))

SMNDC1	AF	Gain	3	Y (Downregulation)
SNRPD1	A3	Loss	3	N
STK17B	AF	Loss	3	N
STK17B	AF	Gain	3	N
STPG4	AF	Gain	3	N
STPG4	AF	Gain	3	N
STPG4	AF	Gain	3	N
STPG4	AF	Gain	3	N
TARS2	AF	Loss	3	N
TARS2	SE	Gain	3	N
TCEAL8	SE	Gain	3	N
THOC7	SE	Gain	3	N
TIMM13	A5	Loss	3	N
TIMP1	SE	Gain	3	N
TMED4	AL	Gain	3	N
TMEM250	AF	Gain	3	N
TMEM44-AS1	A3	Gain	3	N
TMUB1	AF	Gain	3	N
TNR	SE	Gain	3	N
TOMM40	A3	Gain	3	N
TOP2A/Y_RNA	AF	Loss	3	N
TST	AF	Gain	3	N
TTN	SE	Gain	3	N
TTN	SE	Gain	3	N
TTN	SE	Loss	3	N
TXLNG	A5	Loss	3	Y (Downregulation)
UBN2	AF	Gain	3	N
UFM1	A5	Loss	3	N
UQCC2	SE	Gain	3	N
VAMP8	AF	Loss	3	N
VPS37C	AF	Loss	3	N
VPS37C	AF	Loss	3	N
VPS37C	AF	Loss	3	N
XACT	AF	Gain	3	N
YDJC	AL	Loss	3	N
YDJC	A5	Loss	3	N
YDJC	SE	Loss	3	N
YIPF5	SE	Gain	3	N
ZFR	SE	Loss	3	N
ZNF319	A5	Loss	3	N
ZNF444	A3	Loss	3	N
ZNF460	AF	Loss	3	Y (Upregulation)
ZNF460	AF	Loss	3	Y (Upregulation)
ZNF460	AF	Loss	3	Y (Upregulation)
ZNF764	A3	Loss	3	N
ZNF768	AF	Gain	3	N

ACO1	A5	Gain	6	N
ACO1	SE	Loss	6	N
ACO1	SE	Gain	6	N
ARHGAP5	AF	Loss	6	N
ARHGAP5	AF	Loss	6	N
ARPP19	SE	Gain	6	Y (Downregulation)
B4GALT2	A5	Loss	6	N
B4GALT2	AF	Loss	6	N
BATF3	AF	Loss	6	N
BEND4	A5	Loss	6	Y (Downregulation)
BOLA1	A3	Gain	6	N
C19orf53	AF	Gain	6	Y (Downregulation)
C19orf53	AF	Loss	6	Y (Downregulation)
C19orf53	A5	Loss	6	Y (Downregulation)
C20orf27	SE	Loss	6	N
CBL	SE	Gain	6	N
CBX6	A3	Gain	6	Y (upregulation)
CCNT2	RI	Loss	6	N
CCZ1	A3	Gain	6	Y (Downregulation)
CCZ1	SE	Gain	6	Y (Downregulation)
CD83	AF	Gain	6	N
CDKN2A	AF	Gain	6	Y (Downregulation)
COX20	A3	Loss	6	N
CSTF1	AF	Loss	6	N
DAD1	A5	Gain	6	N
DAZAP2	SE	Loss	6	Y (Downregulation)
DEGS1	AF	Loss	6	N
DIRAS1	AF	Loss	6	N
ECE1	AF	Loss	6	N
EEF1A2	RI	Gain	6	N
EIF1	SE	Gain	6	Upregulation, (12h, T-REX against probe alone control), Upregulation, (12h, T-REX against DMSO
EIF3E	AF	Loss	6	Downregulation, (3h, T-REX against DMSO control), Downregulation, (12h, T-REX against DMSO
EIF3E	A5	Gain	6	Downregulation, (3h, T-REX against DMSO control), Downregulation, (12h, T-REX against DMSO
EIF4EBP1	AF	Gain	6	Upregulation, (12h, T-REX against Light alone control), Upregulation, (12h, T-REX against DMSO
EMC10	A5	Loss	6	N
EMC10	SE	Gain	6	N
EMC4	A3	Gain	6	N
EMC4	SE	Loss	6	N
EMC6	A5	Loss	6	Y (Downregulation)
ERBB3	AF	Gain	6	N
ETS2	AF	Gain	6	N

ETS2	AF	Loss	6	N
FAM168B	A5	Loss	6	N
FLNC	SE	Loss	6	N
GNB2	AF	Gain	6	N
GNB2	AF	Loss	6	N
GNB2	A3	Gain	6	N
GPT2	A5	Gain	6	N
HES6	A3	Loss	6	N
HLTF	A3	Loss	6	N
IPO13	A3	Gain	6	N
IRX3	SE	Gain	6	N
ISCA2	A3	Gain	6	N
ISCA2	SE	Gain	6	N
IVNS1ABP	SE	Loss	6	Y (Downregulation)
KLC2	A3	Loss	6	N
KPNA1	A5	Gain	6	N
KPNA1	A5	Loss	6	N
KPNA1	AL	Gain	6	N
LANCL2	SE	Gain	6	N
LBX2-AS1	A3	Loss	6	N
LIN7C	SE	Gain	6	Y (Downregulation)
LINC02631	A5	Gain	6	N
LMBRD2	A3	Loss	6	N
MAP7D1	RI	Loss	6	N
MAPK1	SE	Gain	6	N
MAPRE2	A5	Loss	6	Y (Downregulation)
MEA1	AF	Loss	6	Downregulation, (12h, T-REX against probe alone control), Downregulation, (12h, T-REX against DMSO)
MEA1	AF	Loss	6	Downregulation, (12h, T-REX against probe alone control), Downregulation, (12h, T-REX against DMSO)
MEA1	A5	Gain	6	Downregulation, (12h, T-REX against probe alone control), Downregulation, (12h, T-REX against DMSO)
MEX3C	AF	Gain	6	N
MGARP	AF	Gain	6	N
MID1IP1	A5	Gain	6	N
MID1IP1	SE	Gain	6	N
MRPL13	A5	Loss	6	N
MRPL36	AF	Gain	6	N
MRPL51	A5	Loss	6	N
MRPL51	A5	Gain	6	N
MRPL51	AF	Loss	6	N
MYBL2	SE	Gain	6	N
NAA15	AF	Gain	6	Y (Downregulation)
NACA	AF	Gain	6	N
NDUFAF3	AF	Gain	6	N

NDUFAF3	AF	Loss	6	N
NKD2	SE	Loss	6	N
NR6A1	A3	Gain	6	N
NR6A1	A3	Loss	6	N
NRBF2	SE	Gain	6	N
NRGN	A3	Gain	6	N
ODC1	A5	Gain	6	N
ODC1	AF	Loss	6	N
PFDN1	AL	Loss	6	N
PFDN1	SE	Gain	6	N
PHF10	AF	Gain	6	N
PHYH	AF	Loss	6	N
PKN1	AF	Loss	6	N
PLXNB2	A5	Gain	6	N
POLR2G	AF	Loss	6	N
PPP1R10	AF	Gain	6	N
PRKAB1	AF	Loss	6	N
PTGES3	AF	Gain	6	Y (Downregulation)
PTGES3	MX	Loss	6	Y (Downregulation)
PTGES3	SE	Gain	6	Y (Downregulation)
PTMA	AF	Gain	6	Y (Downregulation)
PTMA	AF	Gain	6	Y (Downregulation)
PTMA/U4	AF	Gain	6	N
R3HDM4	A3	Gain	6	N
RACK1	AF	Loss	6	N
RACK1	RI	Loss	6	N
RAN	A5	Gain	6	Y (Downregulation)
RAN	SE	Gain	6	Y (Downregulation)
RAN	A5	Gain	6	Y (Downregulation)
RASSF8	AF	Gain	6	N
RGS19	AF	Loss	6	N
RNF139	AF	Gain	6	N
RPL32	A3	Gain	6	N
RPL32	A3	Loss	6	N
RPL32	A3	Loss	6	N
RPL9	A3	Gain	6	N
RPL9	A5	Gain	6	N
RPL9	AF	Gain	6	N
RPL9	SE	Gain	6	N
RPL9	SE	Gain	6	N
RPS14	AF	Loss	6	N
RPS2	AF	Gain	6	N
RPS2	MX	Loss	6	N
RPS2	RI	Gain	6	N
RPS6	A5	Loss	6	N
SBNO2	A3	Gain	6	N

SBNO2	A3	Gain	6	N
SETD1B	A5	Loss	6	N
SLC7A5	AF	Gain	6	Y (upregulation)
SNU13	AF	Gain	6	Y (Downregulation)
SNU13	AF	Gain	6	Y (Downregulation)
SNU13	SE	Gain	6	Y (Downregulation)
SRCAP	A5	Loss	6	N
SRCAP	AF	Gain	6	N
STPG4	AF	Gain	6	N
TARS2/MIR6878	AF	Loss	6	N
TARS2/MIR6878	SE	Gain	6	N
TBC1D14	AF	Gain	6	N
TCEAL8	SE	Gain	6	N
TERF2IP	A5	Loss	6	N
TEX45	AF	Loss	6	N
THOC7	SE	Loss	6	N
TIMP1	SE	Gain	6	N
TMEM14C	AF	Loss	6	N
TMEM14C	SE	Gain	6	N
TMEM14C	SE	Gain	6	N
TMEM250	AF	Gain	6	N
TMEM250	RI	Loss	6	N
TOMM40	A3	Gain	6	N
TOP2A	AF	Loss	6	N
TP53	AF	Gain	6	Y (upregulation)
TP53	AF	Gain	6	Y (upregulation)
TP53	AF	Gain	6	Y (upregulation)
TP53	AF	Loss	6	Y (upregulation)
TP53	AF	Loss	6	Y (upregulation)
TP53	AF	Loss	6	Y (upregulation)
TRAF2/MIR4479	AF	Loss	6	N
TRIM23	A5	Gain	6	N
TRMT112	A3	Loss	6	N
TSC22D3	A3	Loss	6	N
TSC22D3	AF	Loss	6	N
TSC22D3	AF	Loss	6	N
TSN	AF	Gain	6	N
TSN	AF	Gain	6	N
TSN	AF	Gain	6	N
TSN	AF	Gain	6	N
TSN	AF	Gain	6	N
TSPAN3	AF	Gain	6	N
TSPAN3	AF	Gain	6	N
TSPEAR-AS2	AL	Loss	6	N
TST	AF	Gain	6	N
TTK	A5	Loss	6	N

TWF2-DT	A3	Gain	6	N
TXLNG	A5	Loss	6	Y (Downregulation)
UBE2N	A5	Gain	6	Downregulation, (12h, T-REX against probe alone control), Downregulation, (12h, T-REX against DMSO)
UBFD1	A5	Loss	6	N
UHRF1	AF	Loss	6	N
UHRF1	AF	Gain	6	N
UQCC2	SE	Gain	6	N
USP22	AF	Gain	6	N
USP22	AF	Loss	6	N
XIST	AF	Loss	6	N
YDJC	A5	Loss	6	N
YDJC	SE	Loss	6	N
YIPF5	A3	Gain	6	N
YIPF5	A5	Loss	6	N
YIPF5	A5	Gain	6	N
YIPF5	SE	Gain	6	N
YWHAE	AL	Loss	6	N
ZBTB34	SE	Loss	6	Y (upregulation)
ZBTB7A	AF	Loss	6	N
ZFR/MIR579	SE	Loss	6	N
ZNF580	AF	Loss	6	Y (Downregulation)
ZNF704	AF	Gain	6	N
ZNF764	A3	Loss	6	N
ZNF764	A3	Gain	6	N
ZNF768	A3	Gain	6	N
ZNF768	AF	Gain	6	N

Supplementary Table 5. Antibodies for Western blot.

**Antibody**	**Source**	**Dilution**
Anti-HaloTag (mouse)	Promega G9211	1:1000 (WB)
mouse monoclonal Anti Flag	Sigma, F3165	1:5000 (WB)
Anti-HA (HRP conjugated) (Rat)	Sigma H3663	1:1000(WB)
V5 Tag Monoclonal Antibody	Invitrgen R96025	1:5000 (WB) 1:200 (IF)
Anti-β-actin (HRP conjugated) (mouse)	Sigma A3854	1:10000 (WB)
Horse anti-mouse IgG (HRP-conjugated)	Cell Signaling #7076	1:5000 (WB)
Goat anti-rabbit IgG (HRP-conjugated)	Cell Signaling #7074	1:5000 (WB)
Anti-tubulin (HRP conjugated)	Cell Signaling #12351	1:5000 (WB)
Anti- S6K1 (Rab) (antibody 1)	Cell Signaling # 2708	1:2000 (WB)
Anti- S6K1 (Rab) (antibody 2)	Cell Signaling # 9202	1:2000 (WB)
Anti- phospho-S6K1 (Rab)	Cell Signaling # 9234	1:1000 (WB)
Anti- 4EBP1	Cell Signaling # 9452	1:2000 (WB)
Anti- phospho-4EBP1	Cell Signaling # 2855	1:1000 (WB)
Anti- eif2alpha	Cell Signaling # 9722	1:2000 (WB)
Anti- phospho-eif2alpha	Cell Signaling # 9721	1:1000 (WB)
Anti- DACH1	Fisher Sentific AG #16877613	1:1000 (WB)
Anti-IPO5	Abcam # ab187175	1:100 (IF)
Anti-Halo (rb pAb)	Promega G9281	1:1000 (IF)
Anti- Calnexin (ms mAb)	Santa Cruz sc-23954	1:200 (IF)
Anti-Cand1	Insight Biotech, sc137055	1:100 (IF)
Anti-SF3A1	Proteintech, 15858-1-AP	1: 2000 (WB)
Anti-ALYREF	Cell Signaling # 12655	1:1000 (WB)
Anti-PRP4	Abcam ab201684	1:1000 (WB)
Anti-PHAX	Proteintech 16481-1-AP	1:800 (WB)
Donkey Anti-Mouse IgG Alexa Fluor 568	Abcam ab175472	1:2000 (IF)
Donkey Anti-Rabbit IgG Alexa Fluor 488	Invitrogen, A21206	1:2000 (IF)

Supplementary Figures

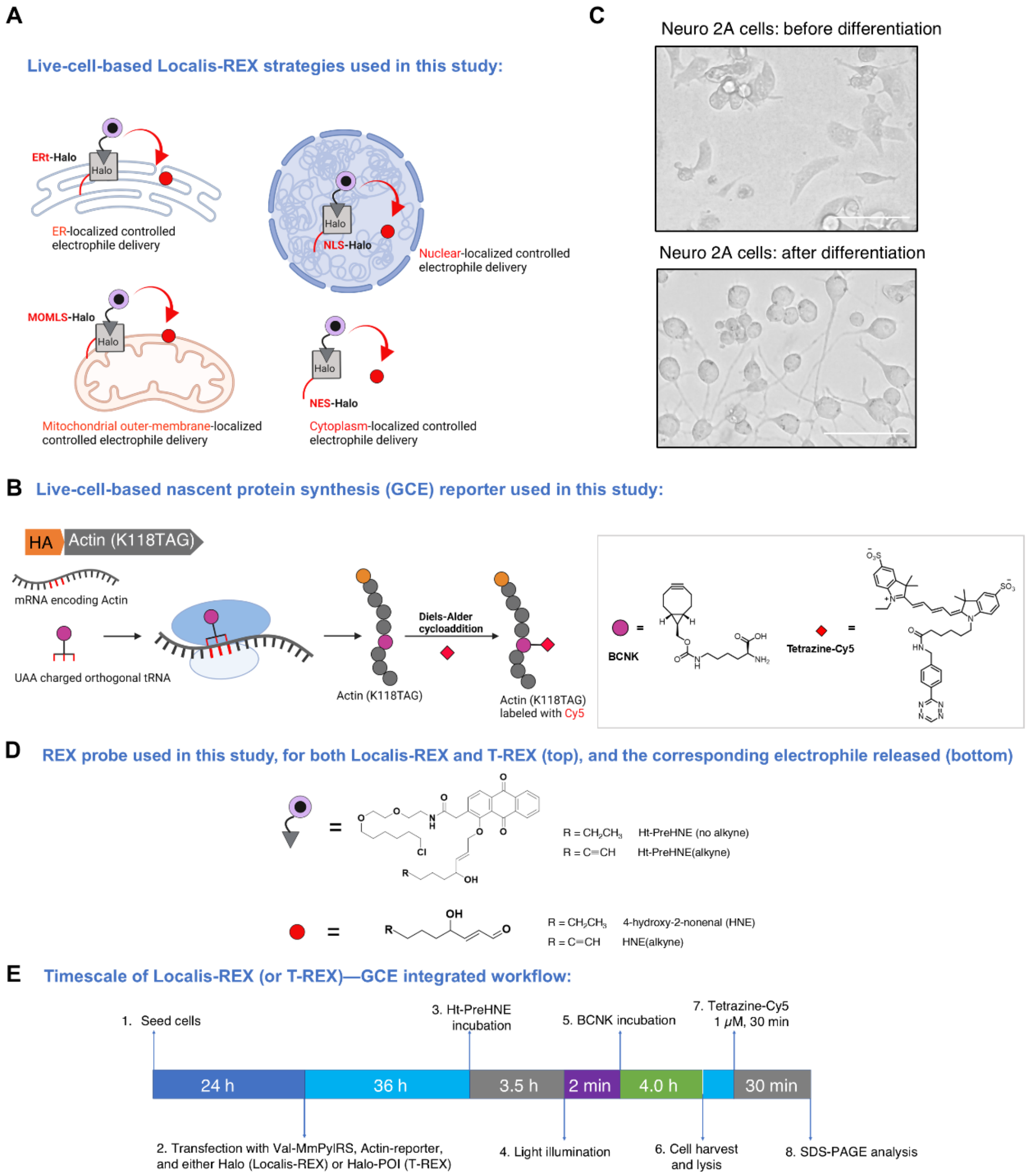

Supplementary Fig. 1. Localis-REX, T-REX, and GCE general workflows. A) Localis-REX setup: differentiated N2A cells ectopically expressing HaloTag and localized to 4 different subcellular locales as illustrated. The approach enables functional-electrophile responders in the specific locale, to be mapped with unparalleled control in timing, ligand chemotype and dosage, and locale. NLS, nuclear localization sequence; NES, nuclear export sequence, MOMLS, mitochondrial out membrane localization sequence; and ERt, ER-targeting sequence. **B)** GCE setup: Amber codon UAG was installed in synthetic mRNA of actin, to replace the codon encoding Lysine at 118th amino-acid position. Only after incubation with unnatural amino acid BCNK (see inset on the right for chemical structure), was translation of synthetic mRNA triggered, generating BCNK-integrated synthetic actin. Post cell lysis, a fluorophore can be attached to the synthetic actin, through Diels-Alder cycloaddition. *Inset:* Structure of BCNK and Tetrazine-Cy5. **C)** Representative bright field images of cultured mouse Neuro 2A (N2A) cells, before

(*top*) and after (*bottom*) treatment with retinoic acid (20 µM, 24 h; differentiation inducer). Scale bar 100 µm. **D)** REX probes used in this study, for both localis-REX and T-REX (*top*), and the corresponding electrophile released (*bottom*). **E)** The workflow for GCE reporter assay, See **Method** for detail procedure.

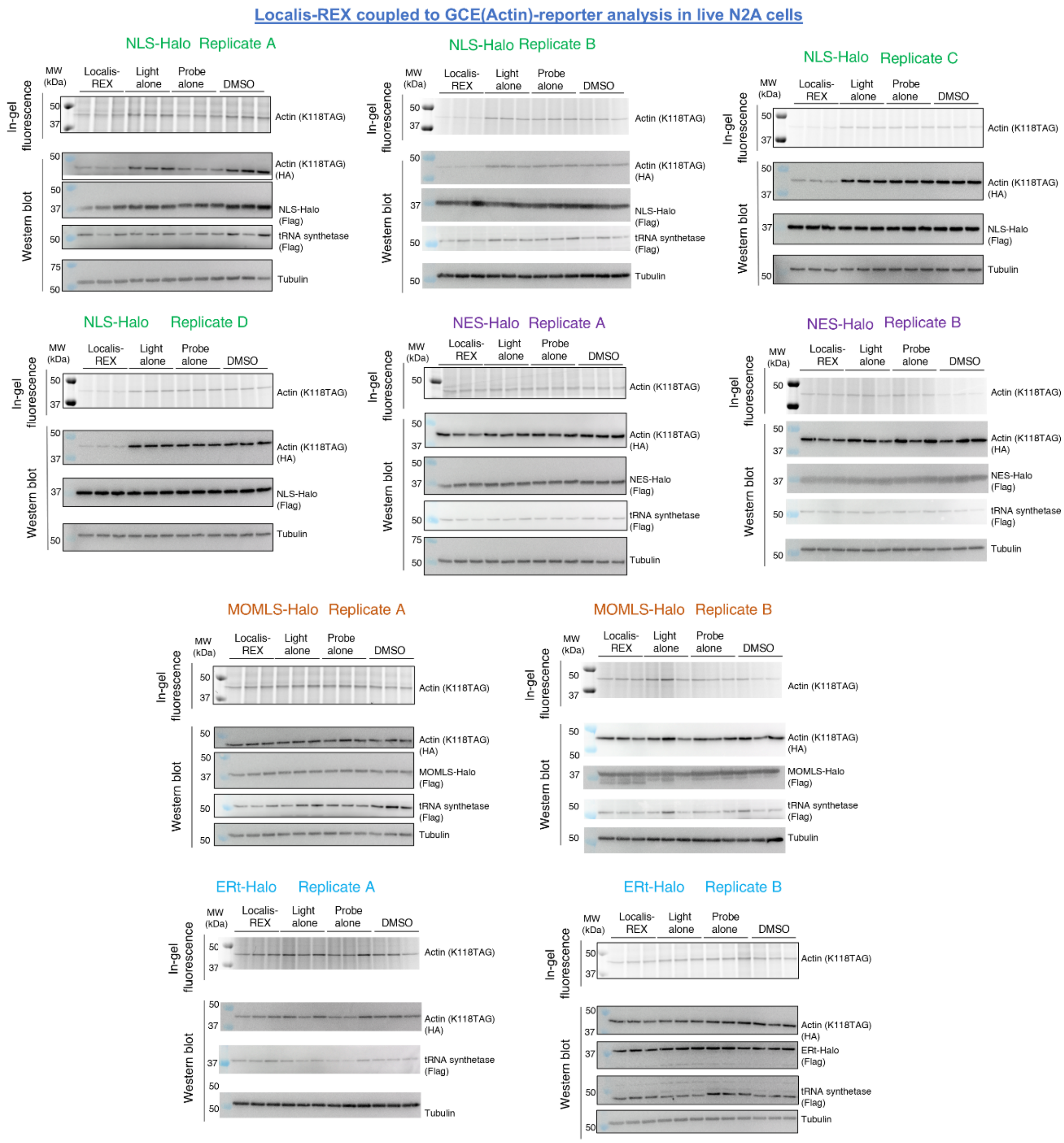

Supplementary Fig. 2. GCE reporter assay, following Localis-REX at different subcellular locales in differentiated N2A cells, shows a prominent reduction of global protein synthesis in response to nuclear-targeted electrophile delivery. Localis-REX—GCE assay (see **Fig. 1A-B** and **Methods**) was performed in differentiated N2A cells expressing either NLS-Halo, NELS-Halo, MOMLS-Halo, or ERt-Halo, and the data were analyzed by in-gel fluorescence and western blot (see **Methods** and also **Supplementary Fig. 1E** for details). NLS, nuclear localization sequence; NES, nuclear export sequence, MOMLS, mitochondrial out membrane localization sequence; and ERt, ER-targeting sequence. Light alone, Probe alone, and DMSO are technical negative controls associated with REX-technologies. See also **Fig. 1B-C**.

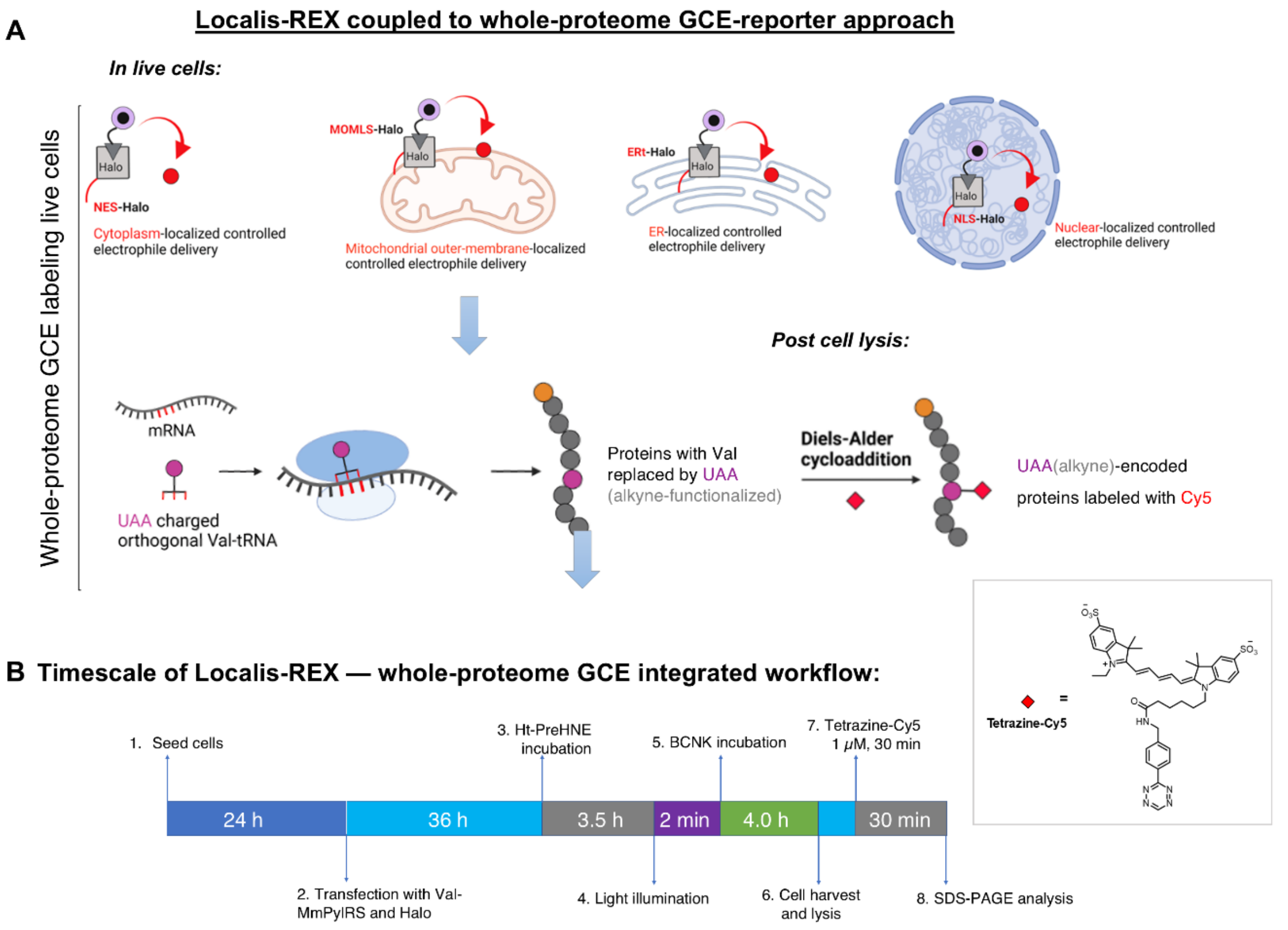

Supplementary Fig. 3. Schematic diagram and workflow of Localis-REX coupled to whole-protemoe GCE labeling assay. Experimental details were described in **Methods**. See also **Supplementary Fig. 4**.

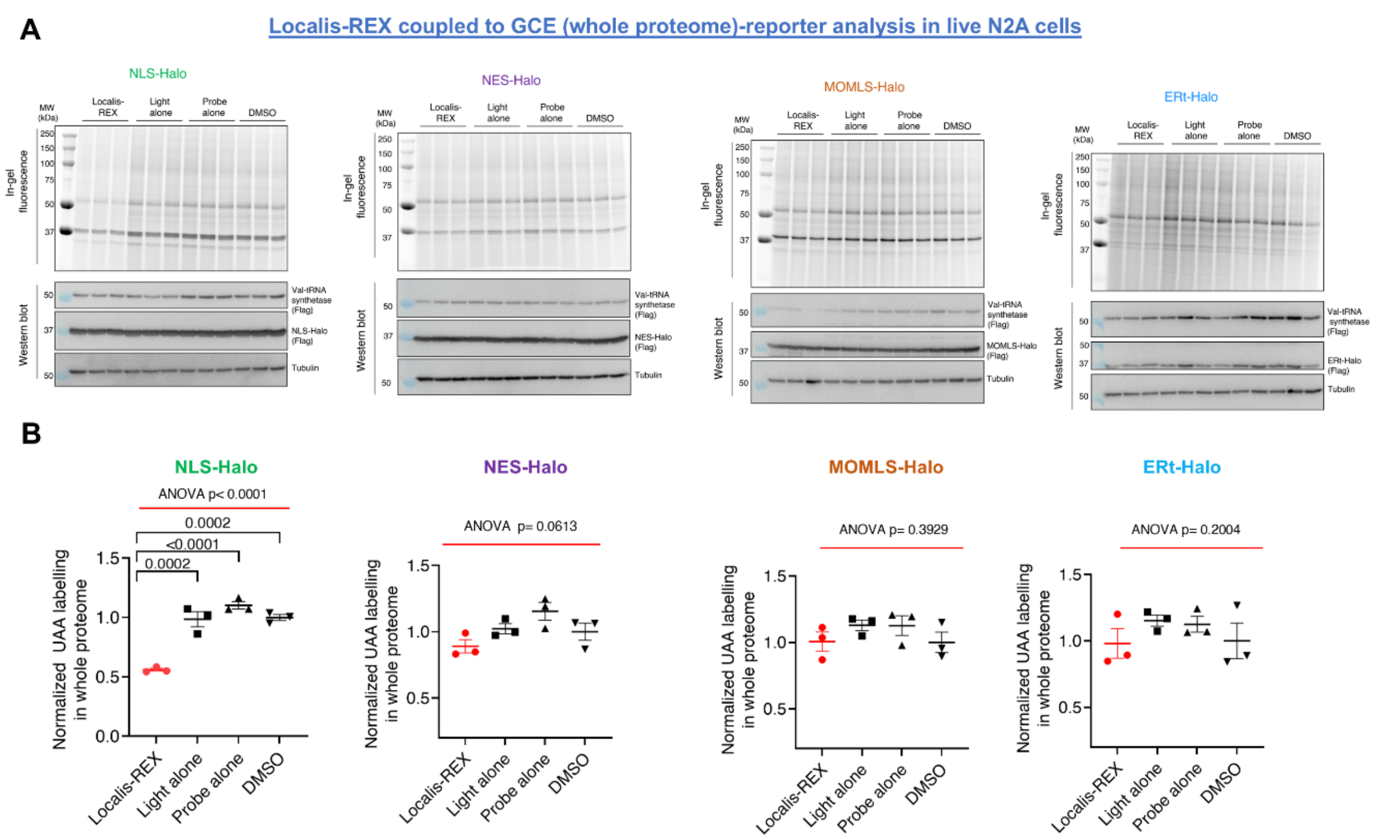

Supplementary Fig. 4. GCE rseporter assay reporting on whole proteomic labeling, following Localis-REX at different subcellular locales, shows a prominent reduction of global protein synthesis in response to nuclear-targeted electrophile delivery. A) The experiment was performed similarly as in **Supplementary Fig. 2**, except the omission of GCE-Actin reporter construct and the deployment of Val-MmPylRS as tRNA synthetase, whereby BCNK competed with valine to be incorporated into whole nascent proteome. (See also Schematic Workflow in **Supplementary Fig. 3**). See (B) for quantification. **B)** 'Normalized relative GCE-reporter signal' (y-axis) corresponds to relative values derived from the in-gel fluorescence signal of Cy5, normalized by that of anti-tubulin. p values were calculated using Tukey's multiple comparisons test (*n*=3). All data present mean ±SEM.

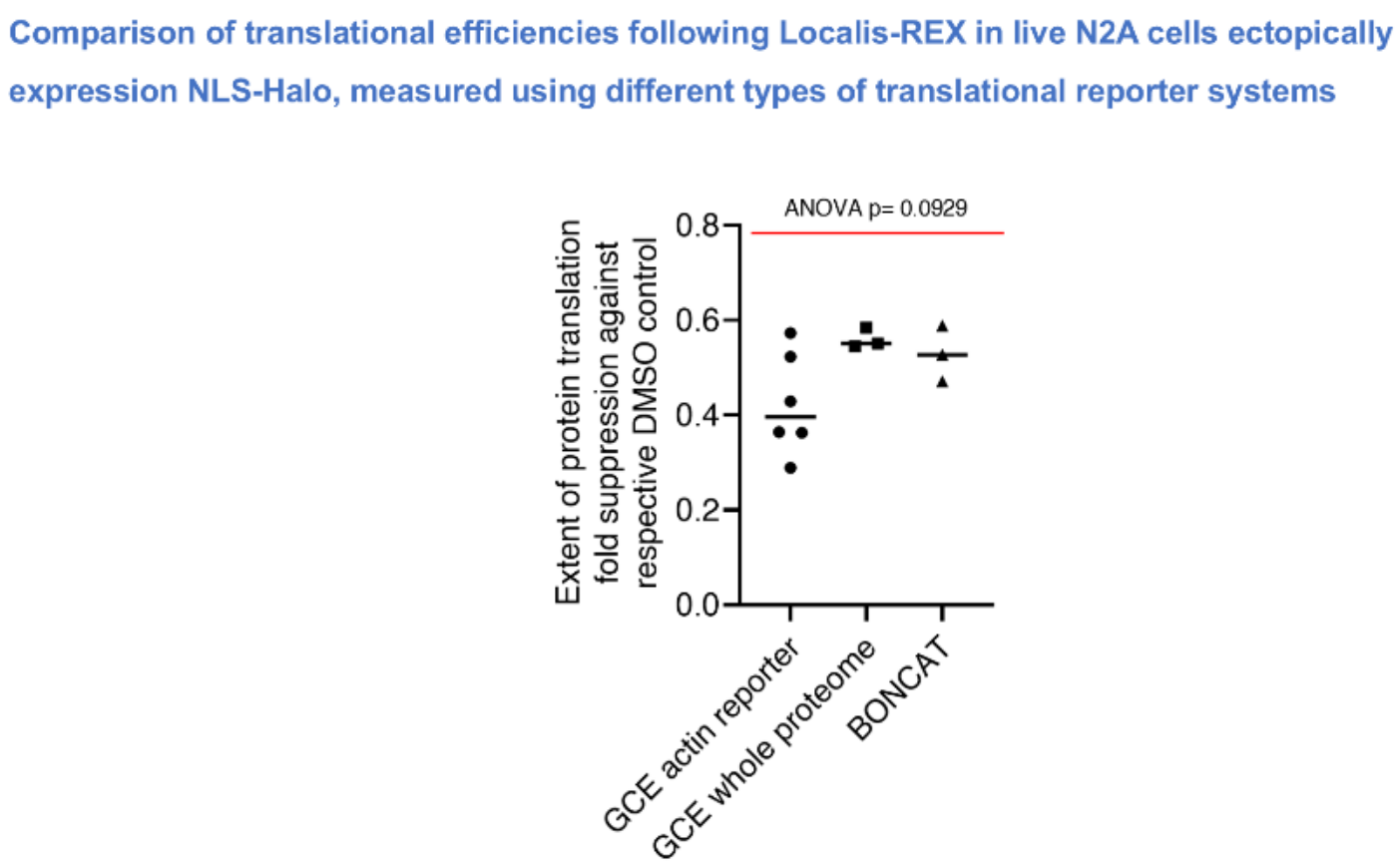

Supplementary Fig. 5. The extent of protein translation suppression is similar across 3 different translation reporter assays, GCE actin reporter, GCE whole proteome, and BONCAT. The degree of protein translation fold suppression upon Localis-REX in live N2A cells extopically expressing NLS-Halo (y-axis) corresponds to the relative values of either BCNK or AHA labelling, normalized to DMSO. Quantification of data shown in **Main Fig. 1B-C** and **Supplementary Fig. 2, 4 and Extended Data Fig. 3**. p values were calculated using Tukey's multiple comparisons test (*n*=6 for GCE actin reporter and n=3 for the rest). All data present mean ±SEM.

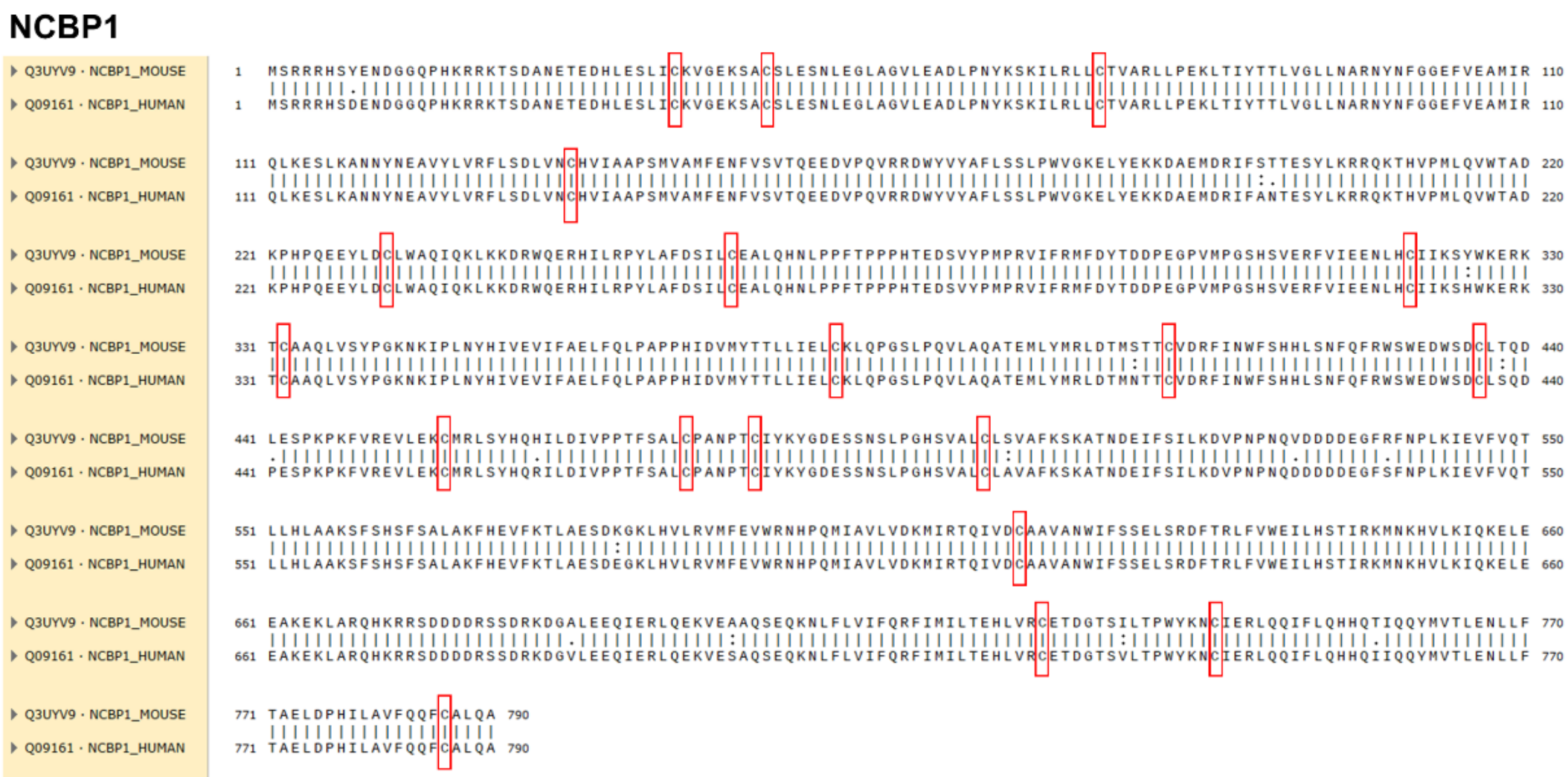

Supplementary Fig. 6. **Sequence alignment of human and mouse NCBP1 shows conservation of all cysteines**. Sequence alignment of human and mouse NCBP1 using SnapGene 6.1.1. Cysteines were highlighted in red boxes.

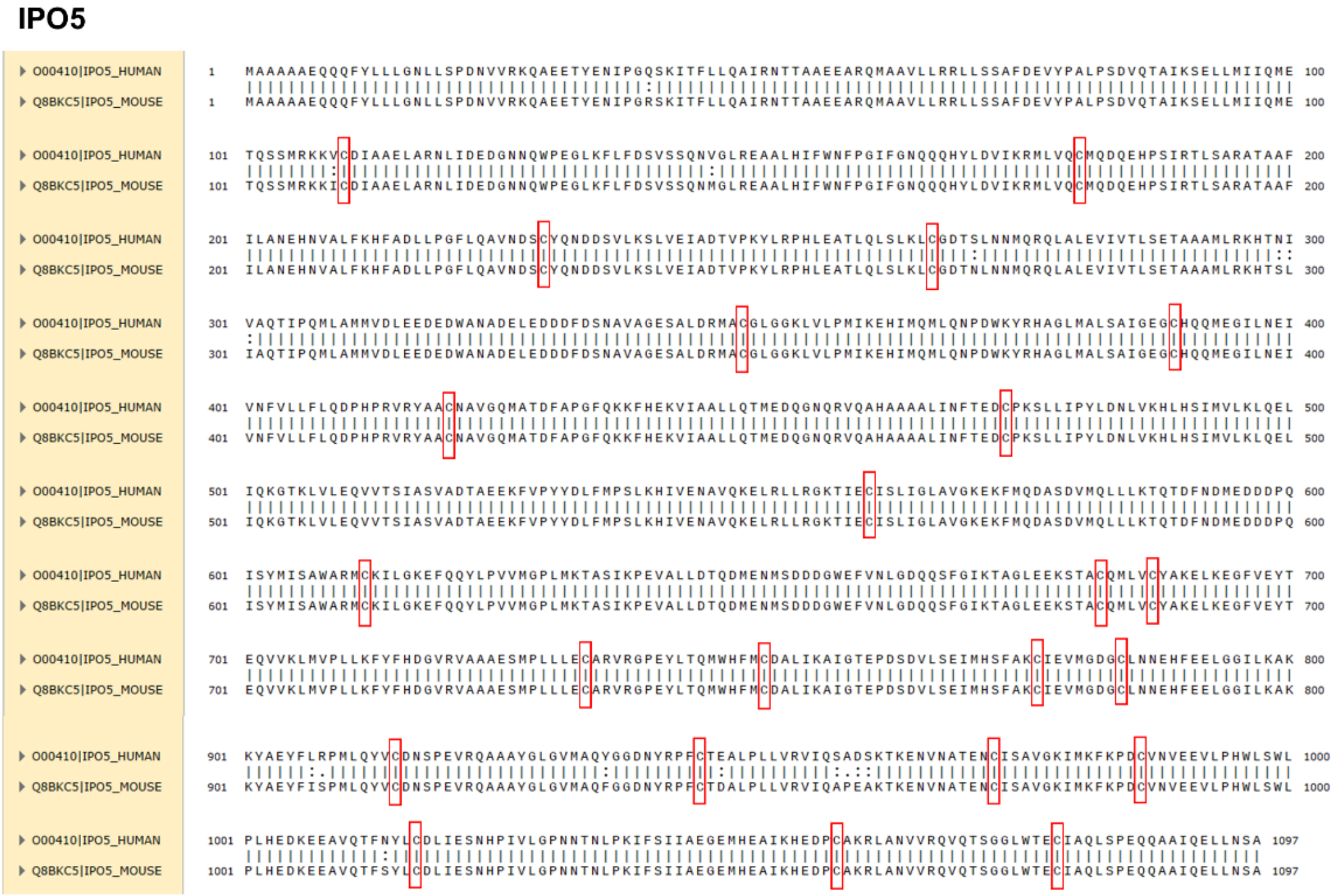

Supplementary Fig. 7. Sequence alignment of human and mouse IPO5 shows conservation of all cysteines. Sequence alignment of human and mouse IPO5 using SnapGene 6.1.1. Cysteines were highlighted in red boxes

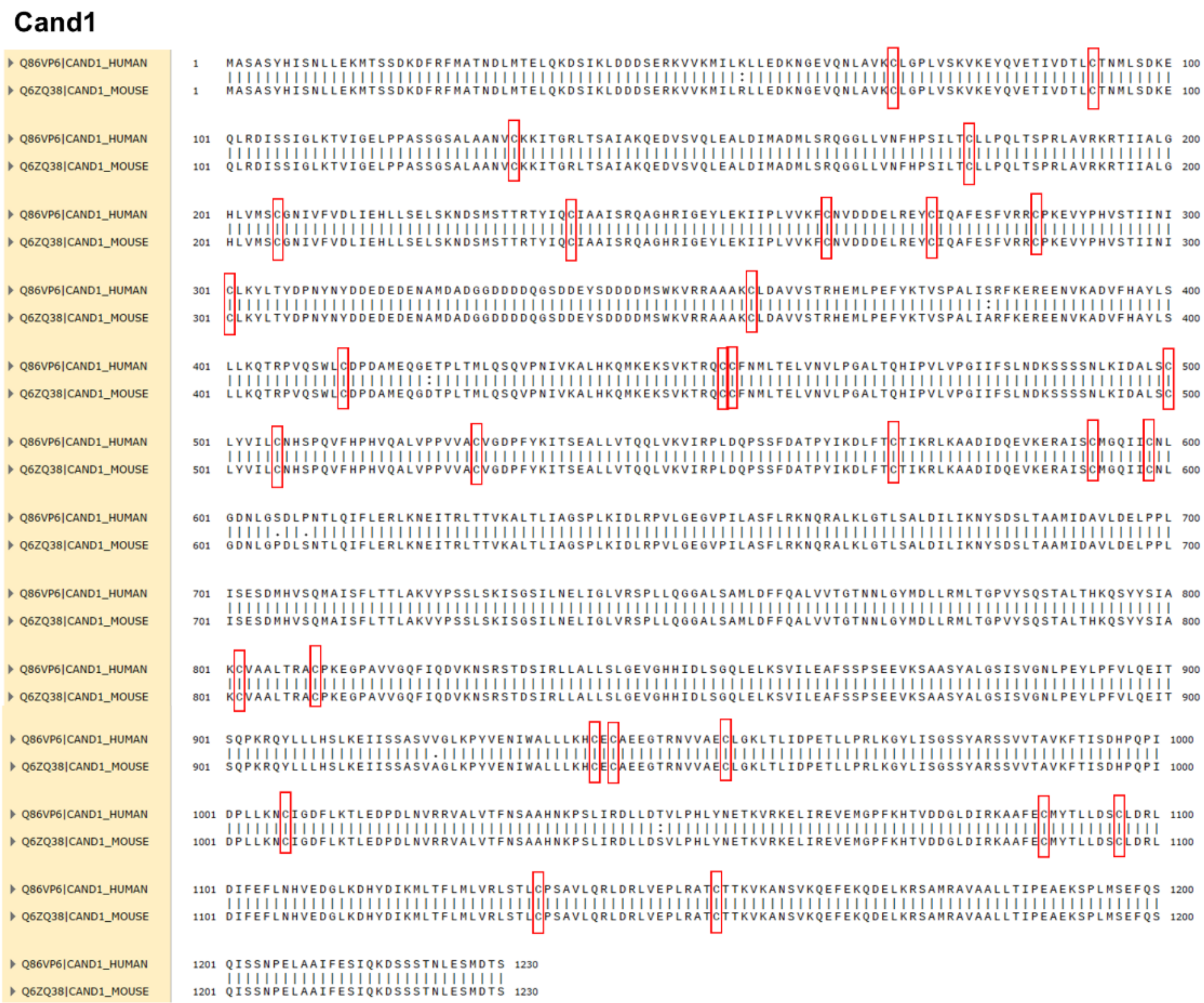

Supplementary Fig. 8. Sequence alignment of human and mouse Cand1 shows conservation of all cysteines. Sequence alignment of human and mouse Cand1 using SnapGene 6.1.1. Cysteines were highlighted in red boxes.

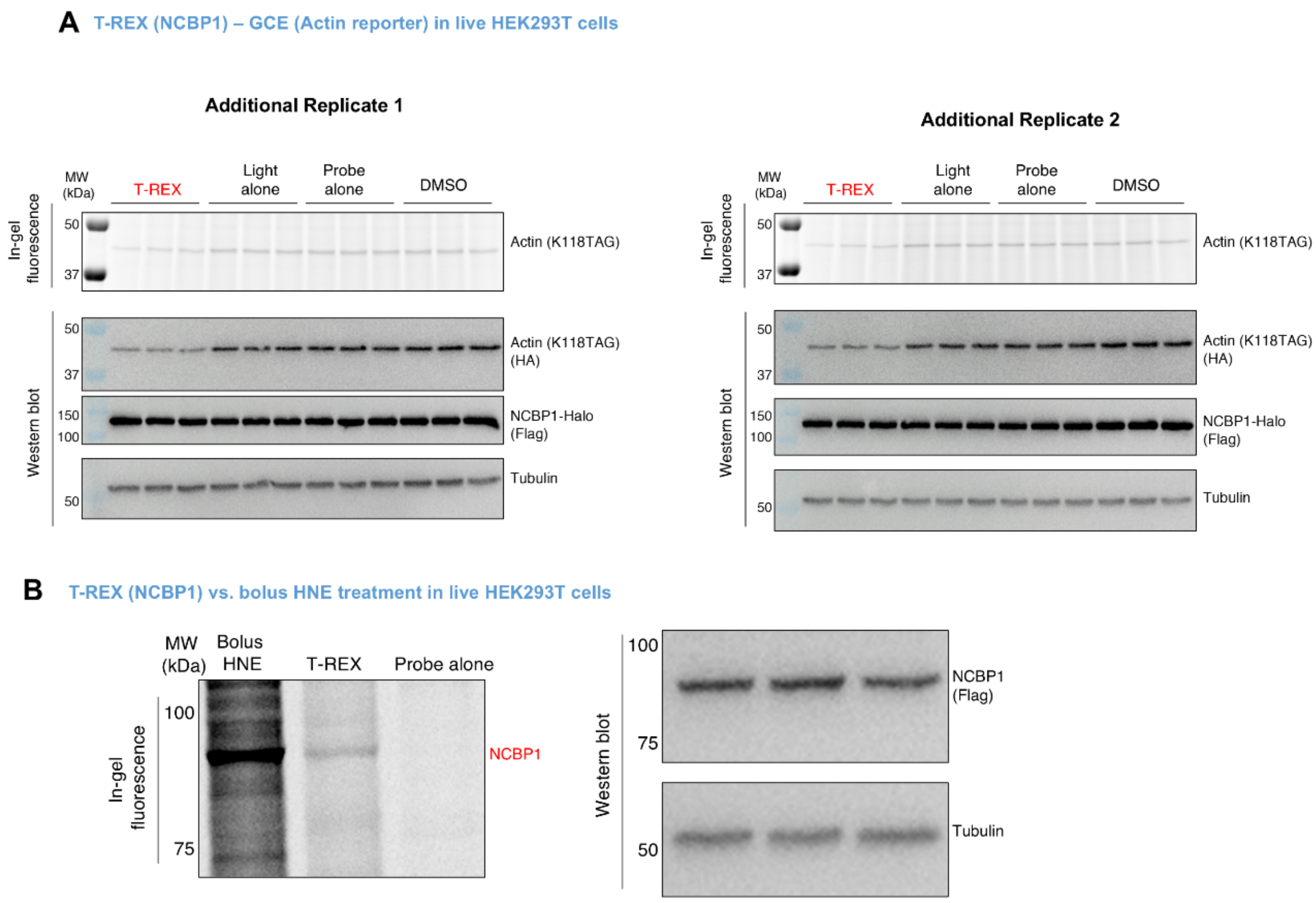

Supplementary Fig. 9. Additional replicates of T-REX-GCE(actin) reporter assay involving NCBP1(wt)-Halo. Side-by-side comparsion between bolus HNE(alkyne) treatment and T-REX contrasts the relative extent of background non-specific modification with respect to HNEylation of ectopically expressed NCBP1-Halo. A) Additional replicates of experiments in **Fig. 2F**. **B)** HEK293T extopically expressing NCBP1(wt)-Halo were either subjected to T-REX or photocaged probe-alone control (see **Methods**), or HNE(alkyne) (10 µM) treatment. In-gel fluorescence (top) and western blot (bottom) analyses were conducted following identical procedure as described in **Fig. 2B**.

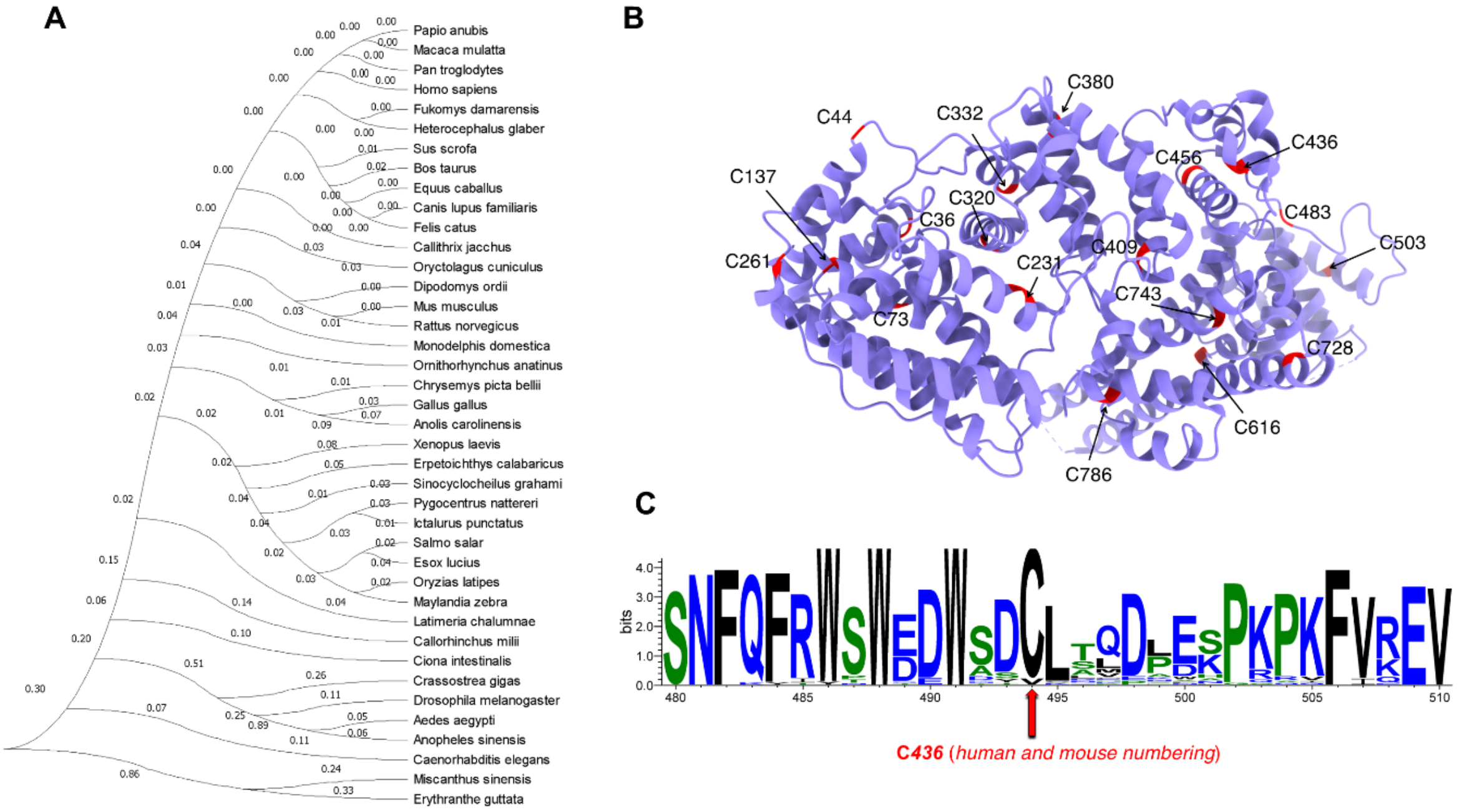

Supplementary Fig. 10. Phylogenetic and structural analyses of NCBP1 alone and in complex with NCBP2. **A)** The amino-acid conservation tree of NCBP1 across 414 eukaryotic species (constructed using MEGA). The number next to each node (ranging from 0 to 1, from lowest possible confidence to highest possible confidence) represents the phylogenetic confidence of the tree topology. **B)** Available single crystal X-ray structure of NCBP1 from human. Only NCBP1 is selectively shown here, from the 2.00 Å resolution single crystal structure of heterotrimeric complex of NCBP1 and NCBP2 from human (PDB: 1H6K). Also see **Fig. 3E**. 19 cysteine residues fully conserved between human and mouse NCBP1 (**Supplementary Fig. 6**) are indicated. **C)** Sequence logos featuring regions flanking the functional electrophile-signaling site discovered in this work (C436) within NCBP1. Sequence logo was generated using WebLogo.

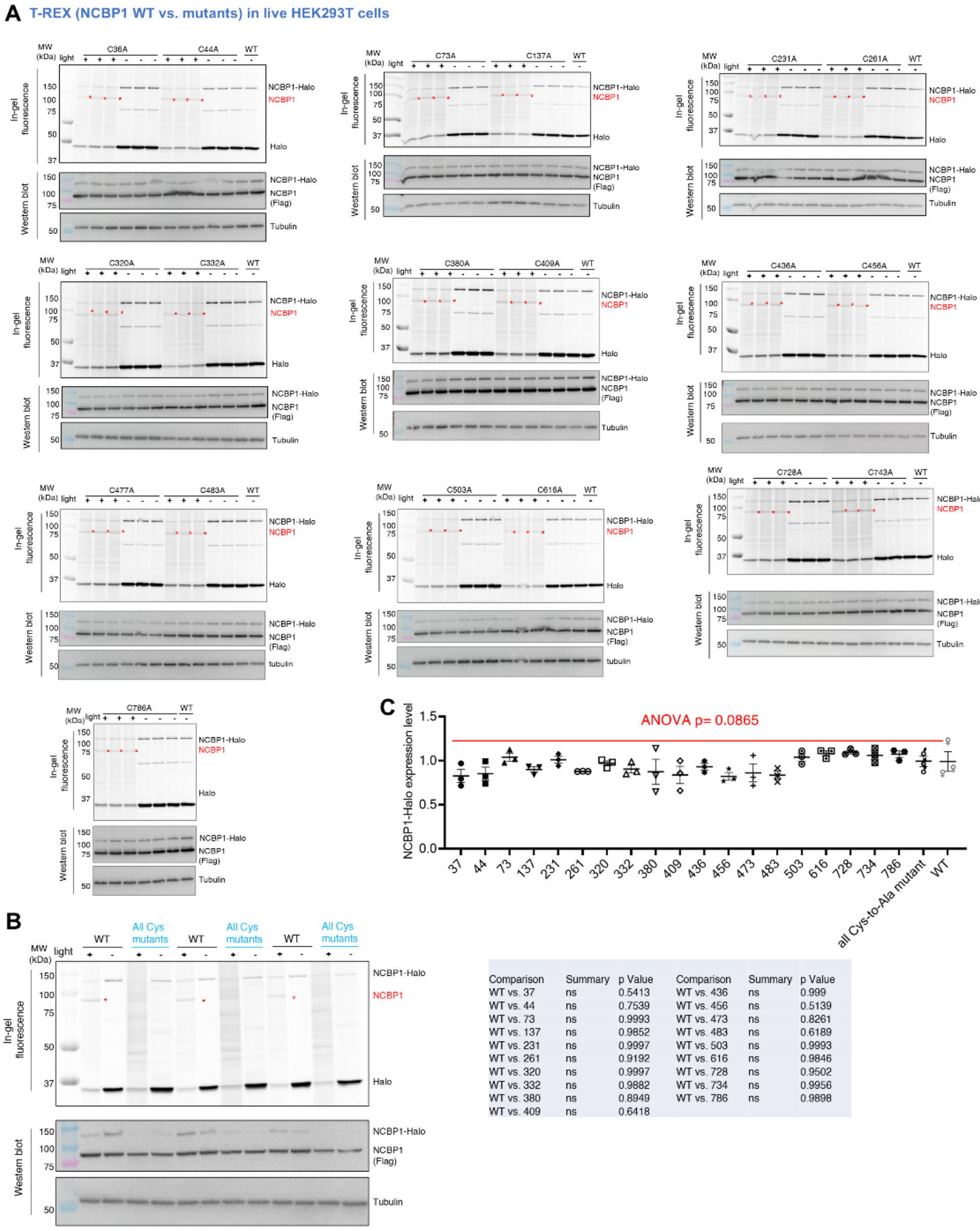

Comparison	Summary	p Value	Comparison	Summary	p Value
WT vs. 37	ns	0.5413	WT vs. 436	ns	0.999
WT vs. 44	ns	0.7539	WT vs. 456	ns	0.5139
WT vs. 73	ns	0.9993	WT vs. 473	ns	0.8261
WT vs. 137	ns	0.9852	WT vs. 483	ns	0.6189
WT vs. 231	ns	0.9997	WT vs. 503	ns	0.9993
WT vs. 261	ns	0.9192	WT vs. 616	ns	0.9846
WT vs. 320	ns	0.9997	WT vs. 728	ns	0.9502
WT vs. 332	ns	0.9882	WT vs. 734	ns	0.9956
WT vs. 380	ns	0.8949	WT vs. 786	ns	0.9898
WT vs. 409	ns	0.6418			

Supplementary Fig. 11. Functional mutagenesis coupled to T-REX evaluates potential HNE-sensing site(s) within NCBP1. See also **Fig. 3A-B** and **Supplementary Table 1**. **A)** HEK293T ectopically expressing NCBP1-Flag-TEV-Halo (wt, or indicated Cys-to-Ala single-mutant) were subjected to T-REX (**Methods**. Also see **Fig. 2A-C** and **3A**). Briefly, datasets for each mutant show results from T-REX vs. control (without light exposure), indicated by '+' and '-', respectively) in three independent biological replicates. The band of HNEylated NCBP1 (post TeV-protease-assisted separation of NCBP1-Flag and Halo) was marked by *. See **Fig. 3A** for the corresponding relative ligand-occupancy quantification across all mutants studied vs. wild-type. Tubulin were used as a loading control. **B)** An independent biological replicate to that shown in **Fig. 2B**. **C)** Quantification of NCBP1-Halo ectopic expression levels across all 19 Cys mutants and wild-type (wt). The numbers show Cys to Ala single-mutation sites. The table below shows statistical analysis and power across pairwise comparison against wild-type. p values were calculated with Tukey's multiple comparisons test (*n*=3).

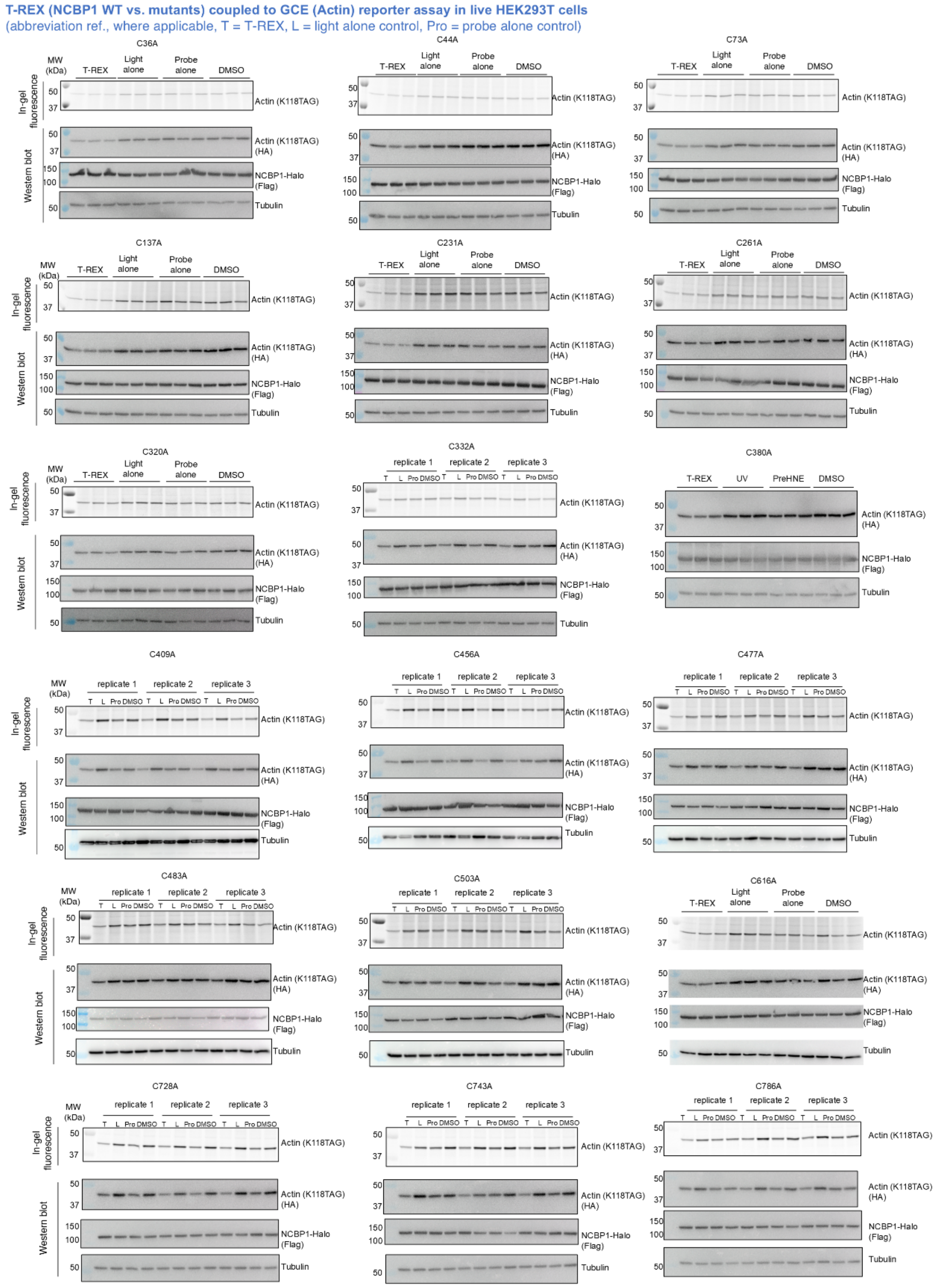

Supplementary Fig. 12. T-REX coupled to GCE reporter assay leveraging mutant- vs. wt-NCBP1-Halo revealed that only C436-specific HNEylation functionally suppresses protein translation. See also **Fig. 2F** and **3C-D.** Live-cell-based T-REX—GCE assay, as illustrated in **Supplementary Fig. 1E** workflow, was executed in HEK293T expressing (indicated NCBP1-mutant)-Halo (See **Methods**). See **Fig. 2F** and **3C** for comparison with results using NCBP1 (wt)-Halo, and corresponding quantification.

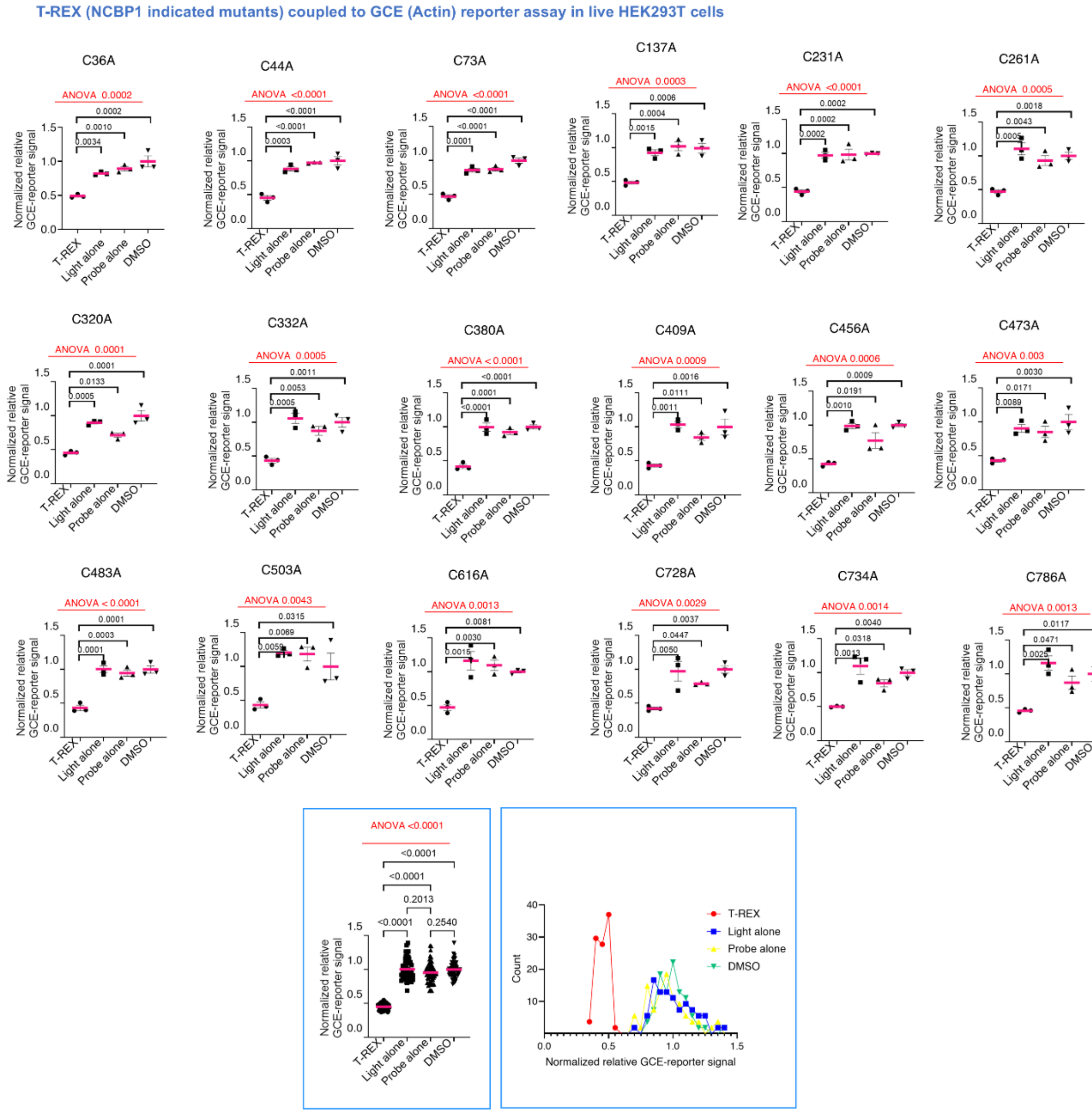

Supplementary Fig. 13. Quantification of experimental results from Supplementary Fig. 12. y-axis indicates relative values of GCE(actin) reporter signal normalized by NCBP1-Halo expression and tubulin expression levels. *P* values were calculated using Tukey's multiple comparison test (*n*=3). All data present mean ± SEM. The two boxed insets (bottom right) represent quantification of all the above data from all mutants combined, in two different representations (left: dot plot; right: histogram plot).

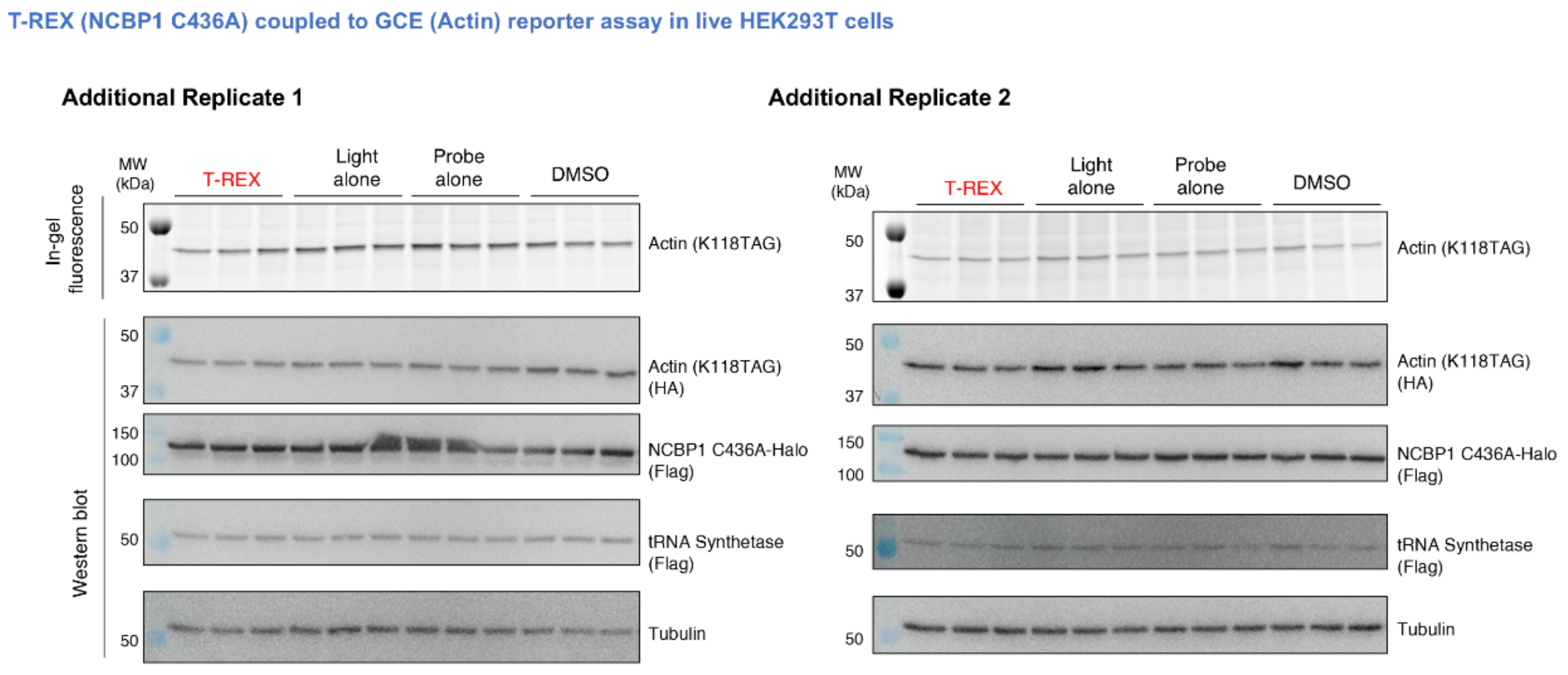

Supplementary Fig. 14. **T-REX coupled to GCE(actin) reporter assay involving NCBP1(C436A)-Halo.** Additional independent biological replicates to that shown in **Fig. 3C**.

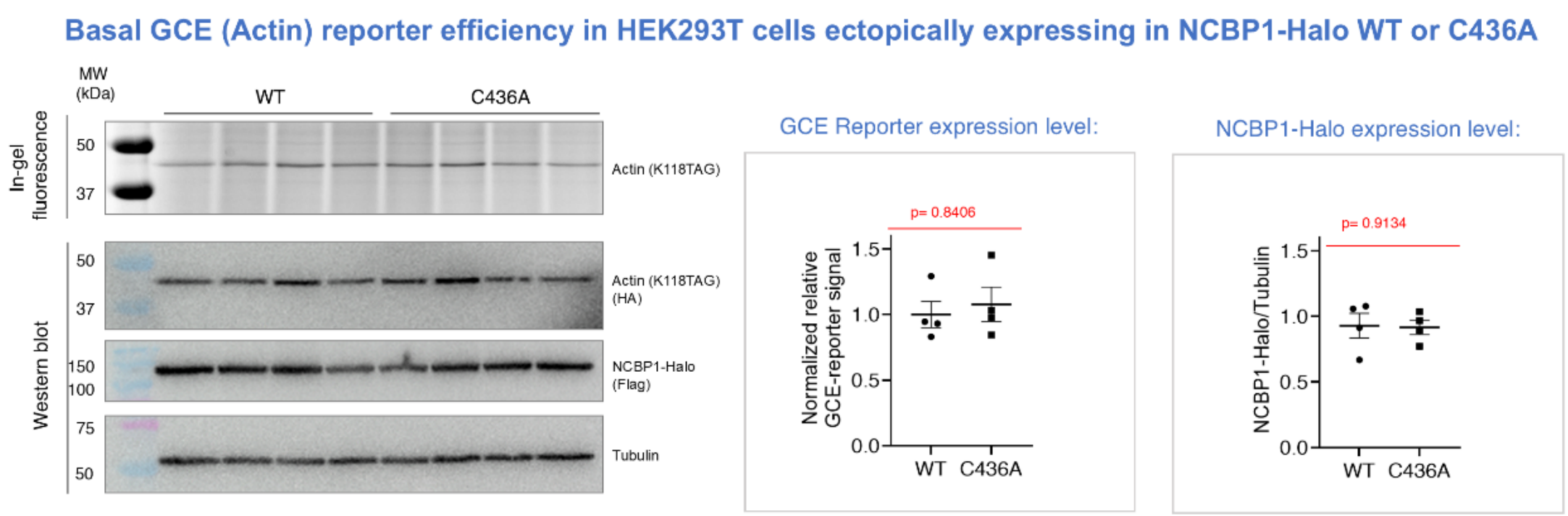

Supplementary Fig. 15. **The basal level of translation efficiency remained the same between cells expressing NCBP1(wt)-Halo and those expressing NCBP1(C436A)-Halo.** Live-cell-based GCE assay, as illustrated in **Supplementary Fig. 1E**, was executed in HEK293T expressing NCBP1(wt)-Halo or NCBP1 (C436A)-Halo (see **Methods**). Basel level of translation efficiency was examined for DMSO-treated control samples, as reported by Actin(K118TAG) expression level, normalized against NCBP1 (wt or C436A) and against loading control (tubulin). Insets show quantification of GCE reporter expression level and NCBP1-Halo expression level normalised over that of tubulin. p values were calculated with unpaired two-tailed student's *t*-test (*n*=4). All data present mean ±SEM.

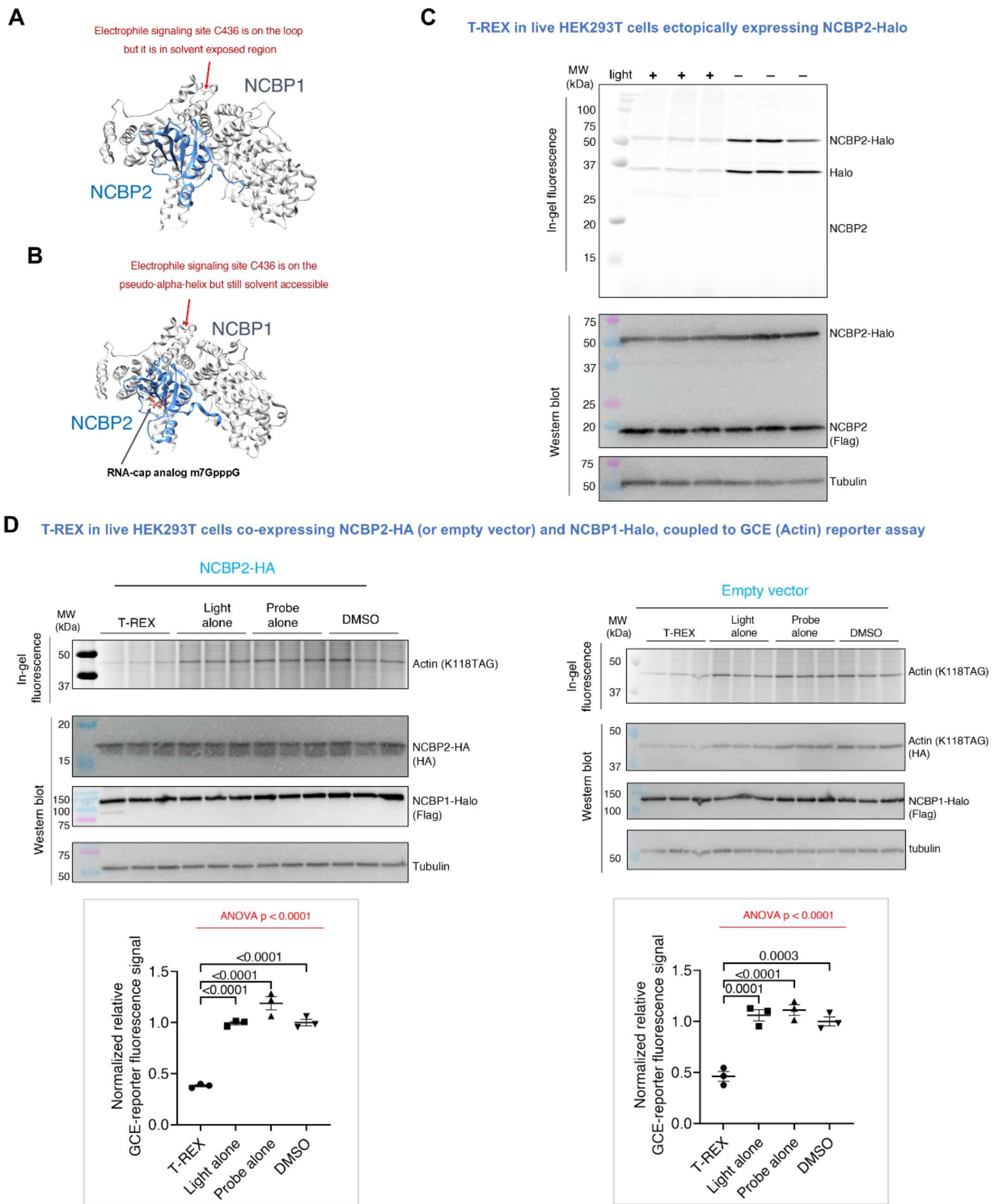

Supplementary Fig. 16. NCBP1 binding partner, NCBP2, has no effect on the extent of translation suppression induced by NCBP1-HNEylation. A and B. Crystal structures of NCBP1 and NCBP2, bound **(A)** and unbound **(B)** to capped mRNA. C436 (indicated with a red arrow) is in the solvent-exposed region in both cases. PDB: 1N52 for **A** and 1N54 for **B**. **C.** HEK293T expressing NCBP2-Flag-TeV-Halo were subjected to T-REX (as described in **Methods** and in **Fig. 2B-C** and **3A**). In-gel fluorescence analysis showed no detection of HNE-modified NCBP2. **D.** HEK293T co-expressing NCBP1-Flag-Halo, and either NCBP2-HA or empty vector (EV), replete with GCE-reporter constructs, were subjected to T-REX-GCE (as described in **Methods**). Cells were treated with BCNK (50 µM, 4 h). Post cell lysis, and coupling with tertrazine-Cy5, samples were analyzed by in-gel fluorescence (Cy5) reporting protein translation. *Insets at the bottom:* Quantification. p values were calculated using Tukey's multiple comparisons test (*n*=3). All data present mean ±SEM.

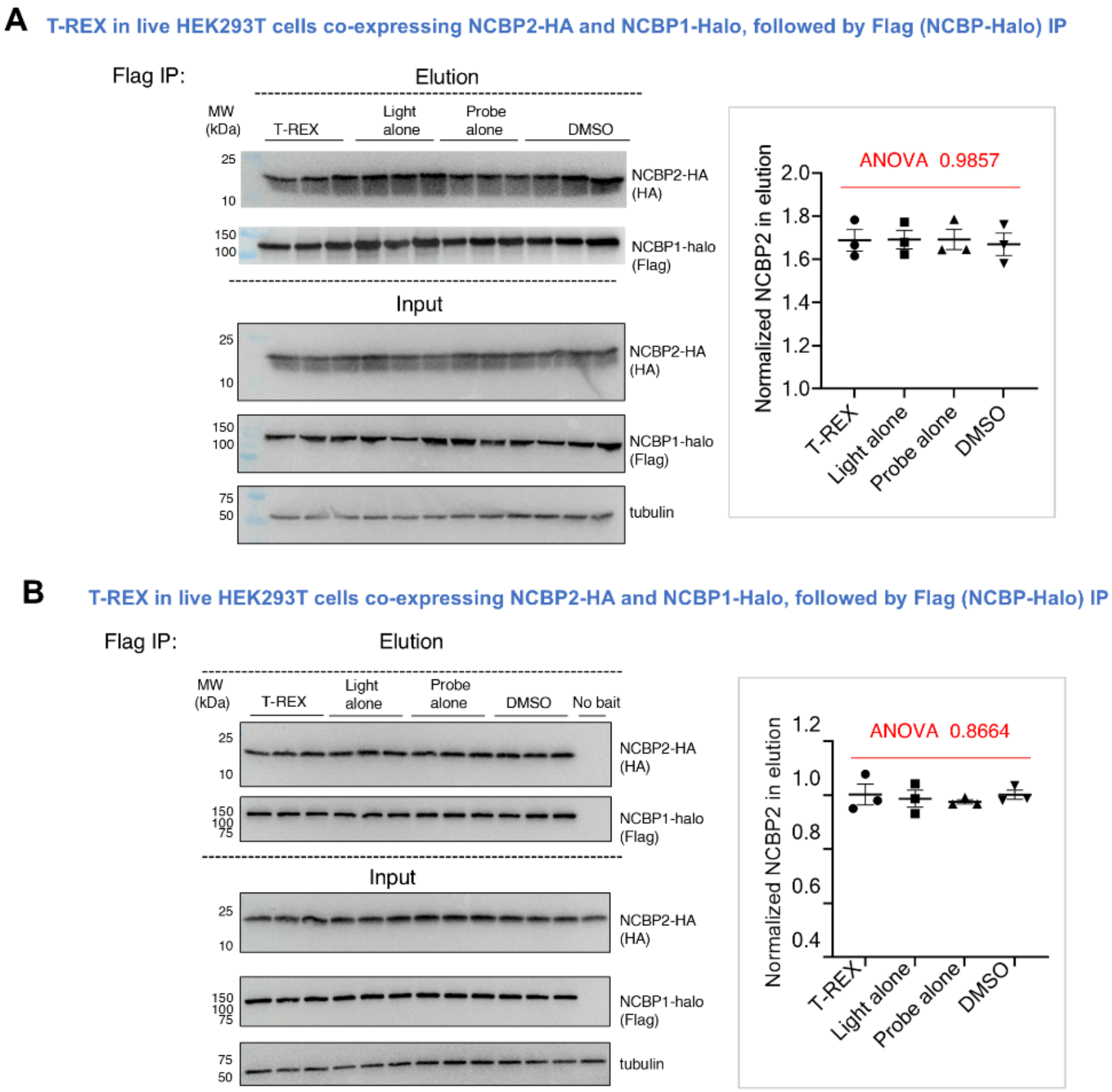

Supplementary Fig. 17. NCBP1-NCBP2 intreaction is not affected by NCBP1-HNEylation. A) Following T-REX-enabled NCBP1-HNEylation in HEK293T (see **Methods** for details) ectopically expressing NCBP1-Flag-Halo and NCBP2-HA, against indicated T-REX technical controls, Anti-Flag pulldown (see **Methods** for detail) was performed. Input and elution samples were subjected to SDS-PAGE, and western blot analyses using indicated antibodies. *__Right panel__* quantification of the protein level of eluted NCBP2-HA normalized by NCBP1-Halo expression*. p* values were calculated using Tukey's multiple comparison test (*n*=3). All data present mean±SEM. **B)** The experiment was performed identically to (**A**) above, except the addition of 'no bait' control (i.e., HEK293T cells transfected with empty vector in place of NCBP2-HA). *__Right panel:__* quantification of the protein level of eluted NCBP2-HA normalized by NCBP1-Halo expression*. p* values were calculated using Tukey's multiple comparison test (*n*=3). All data present mean±SEM.

T-REX (NCBP1) coupled to RNA-Seq at 6 h post T-REX in live HEK293T cells

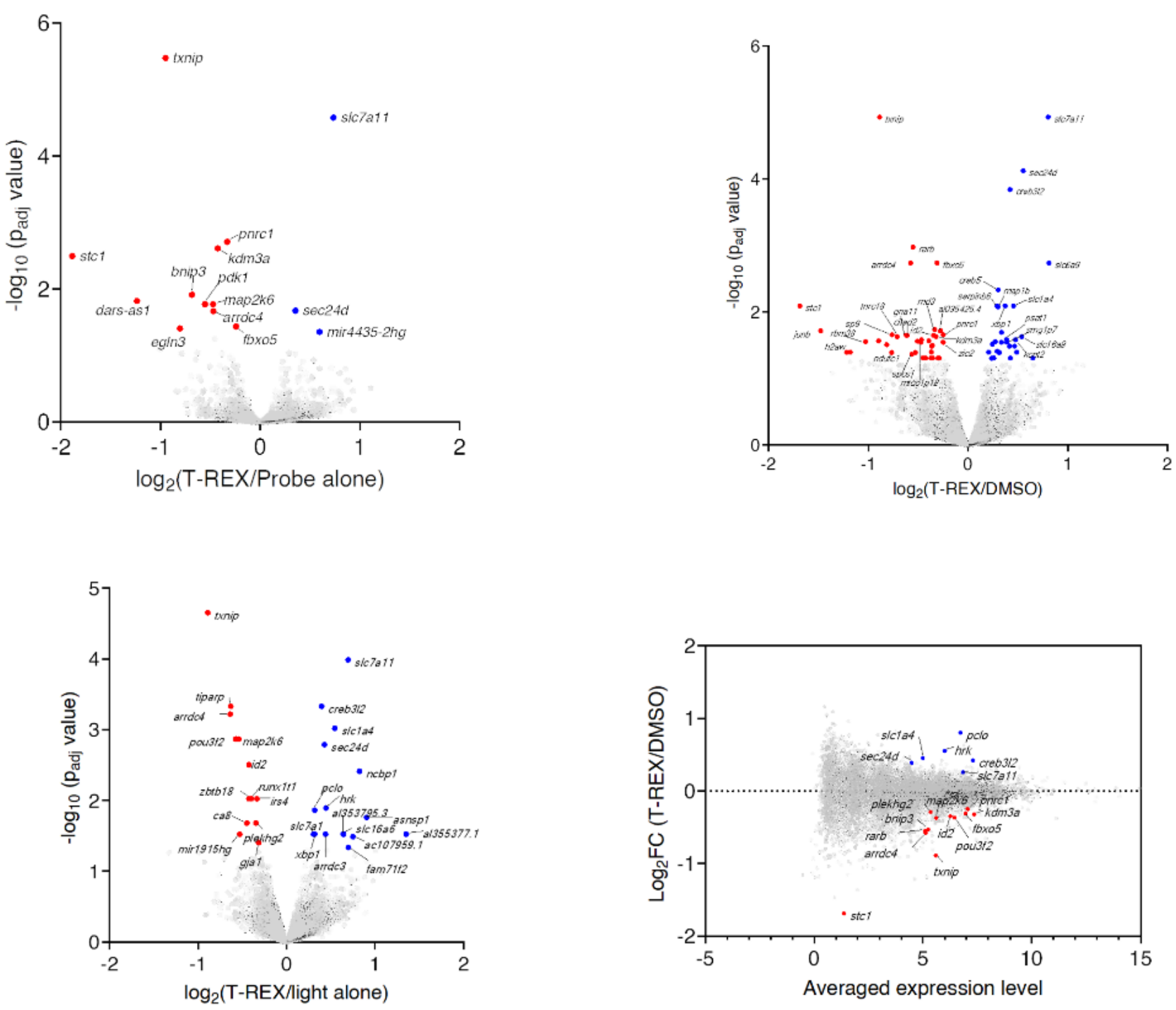

Supplementary Fig. 18. Volcano and MA plots derived from RNA-seq analysis showing statistically-significant differentially-expressed genes (SDEs) under T-REX with respect to indicated controls (*6 h post T-REX*). As in **Extended Data Fig. 9B-E**, except datasets are from 6 h time-point post T-REX. Select genes that were downregulated SDEs under T-REX were marked with **red** dots, and upregulated SDEs, with **blue** dots. Gene names were shown for arbitrarily-selected SDEs. See also **Supplementary Data 2**.

T-REX (NCBP1) coupled to RNA-Seq at 12 h post T-REX in live HEK293T cells

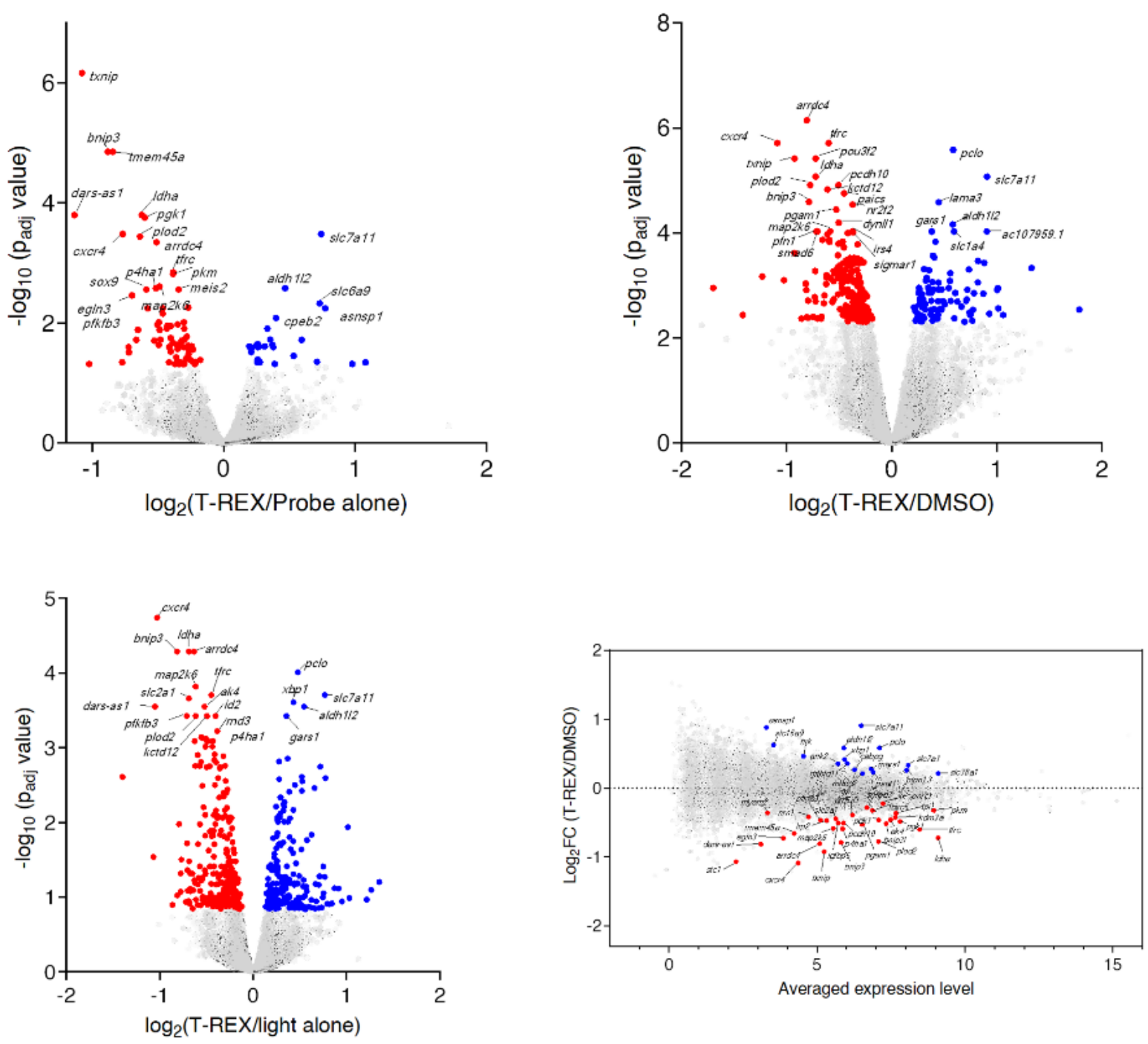

Supplementary Fig. 19. Volcano and MA plots derived from RNA-seq analysis showing statistically-significant differentially-expressed genes (SDEs) under T-REX with respect to indicated controls (*12 h post T-REX*). As in **Extended Data Fig. 9B-E**, except datasets are from 12 h time-point post T-REX. Select genes that are downregulated SDEs under T-REX were marked with **red** dots, and upregulated SDEs, with **blue** dots, and gene names are shown for arbitrarily-selected SDEs. See also **Supplementary Data 2**.

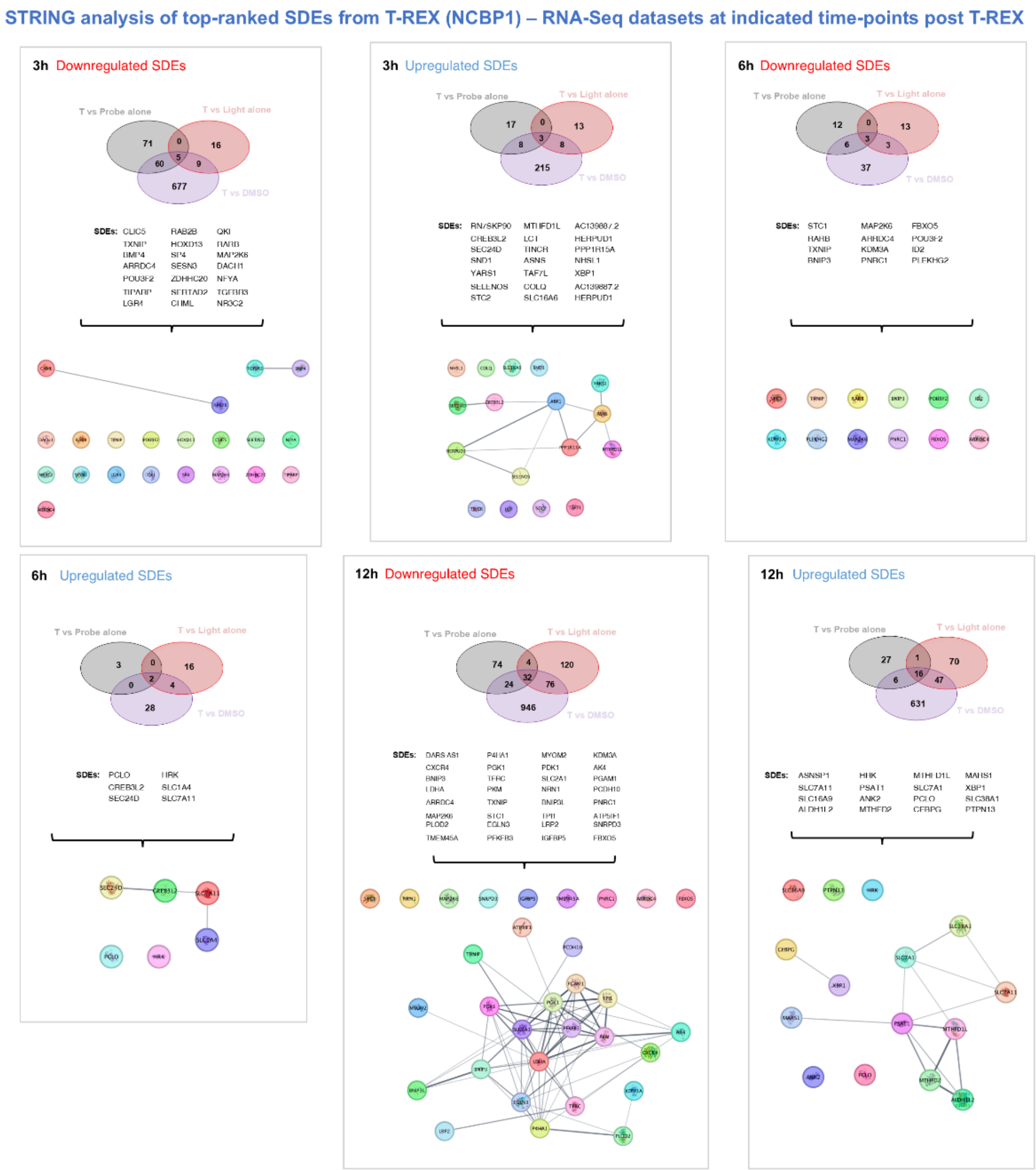

Supplementary Fig. 20. STRING analysis of top-ranked SDEs from RNA-seq datasets. The numbers within each Venn diagram correspond to the number of differentially-expressed genes (DEGs) altered in response to NCBP1-HNEylation, with respect to each indicated control group (light alone, probe alone, or DMSO treatments), in an either down- or upregulated manner, at a given time-point post NCBP1-HNEylation. [For instance, (71 (unique) +60 (overlapping with DMSO) +5 (overlapping with light alone and DMSO) DEGs were downregulated following T-REX with respect to probe-alone control, 3h post uncaging]. Subsequently, DEGs manifesting $p \leq 0.05$ under at least two experimental sets, out of the 3 sets of comparisons deployed (namely: T-REX against the 3 respective controls), were considered as statistically-significant DEGs (SDEs), which are listed underneath each Venn diagram, along with the output from STRING analysis, using default cut-off thresholds in Cytoscape (v3.10.1). See also **Supplementary Data 3**.

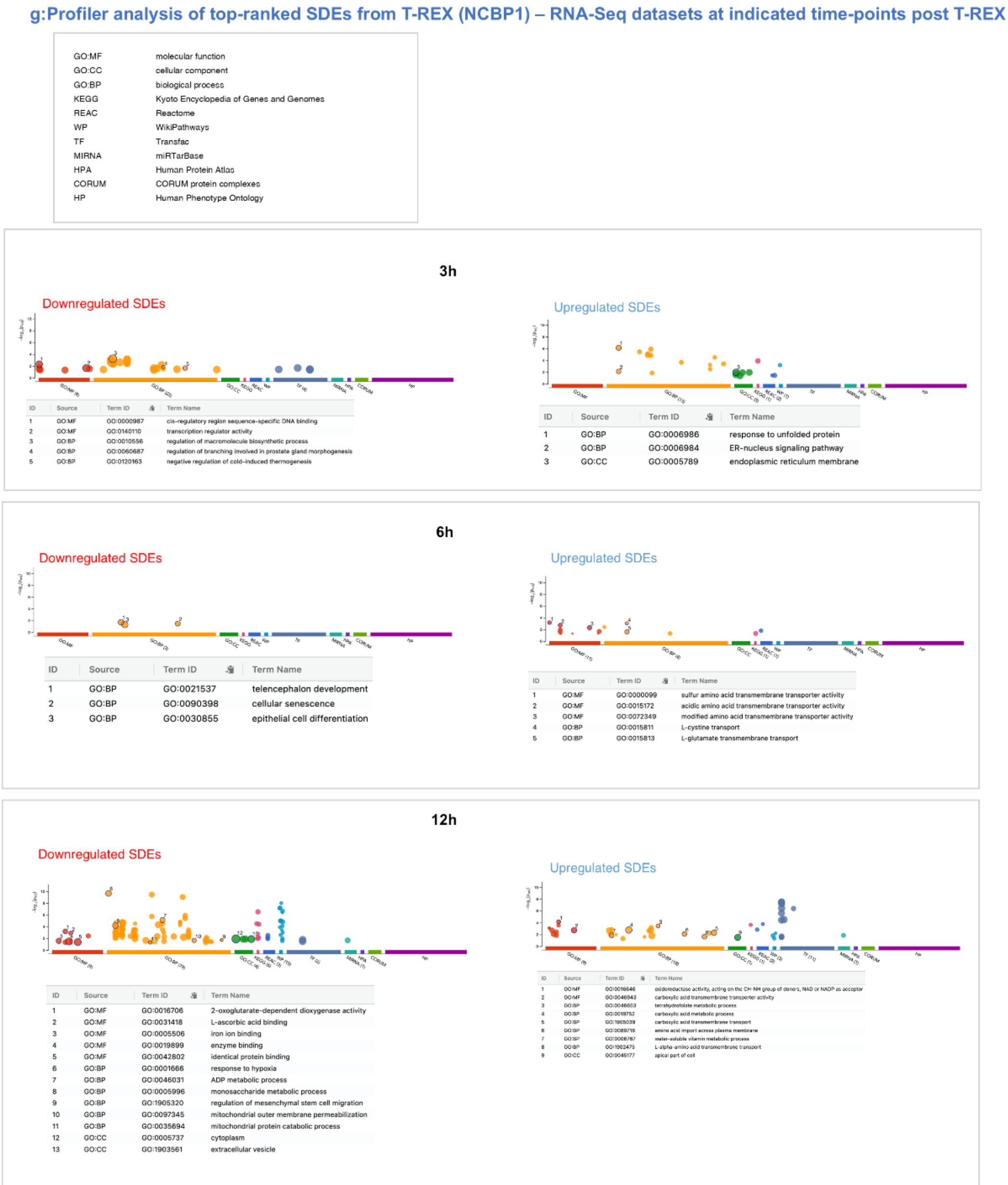

Supplementary Fig. 21. *g:Profiler* analysis of top-ranked SDEs from RNA-seq datasets. SDEs shown in the **Supplementary Fig. 20** were subjected to online software *g:Profiler* (https://bio.tools/gprofiler). See also **Supplementary Data 3**.

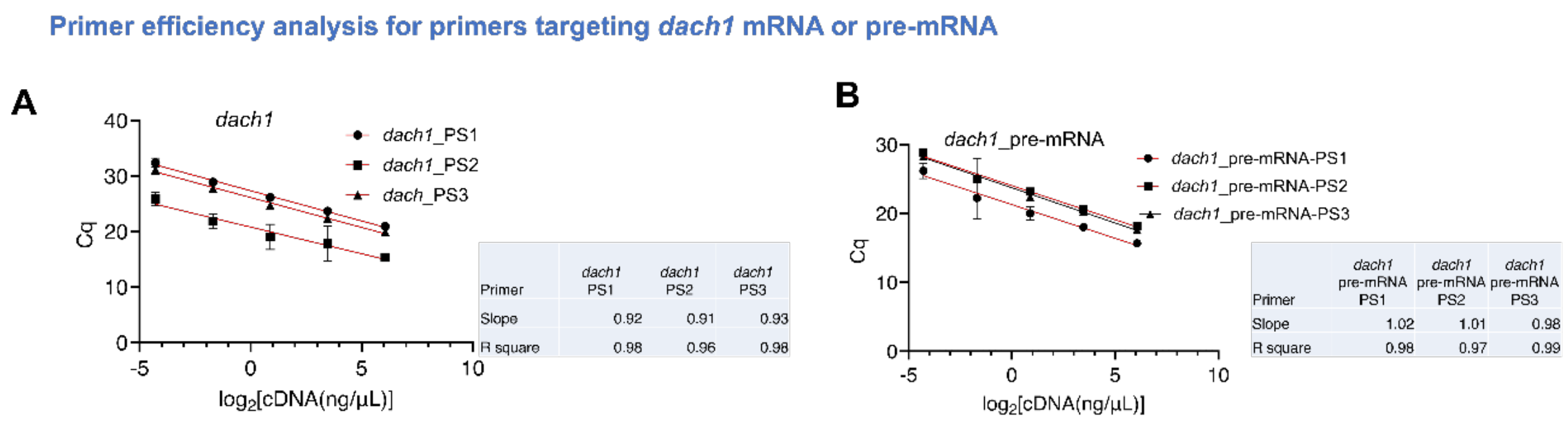

Supplementary Fig. 22. Primer efficiency tests of *DACH1* mRNA and pre-mRNA. Three sets of primers were designed and tested for each indicated gene as described in **Methods**. Briefly, five or six serial dilutions (1:6) of cDNA obtained from HEK293T were tested to obtain the quantification for cycle (Cq) values. [n = 6 replicates (3 biological replicates with 2 technical replicates); error bars represent SEM; linear regression analysis was performed, yielding the results shown in the table]. Primer efficiency values of 90–110% were considered as the selection criteria for primer sets. The respective primer set (PS) 1 was selected in each case: (A) *dach1* mRNA and (B) *dach1* pre-mRNA. See also **Supplementary Table 3**.

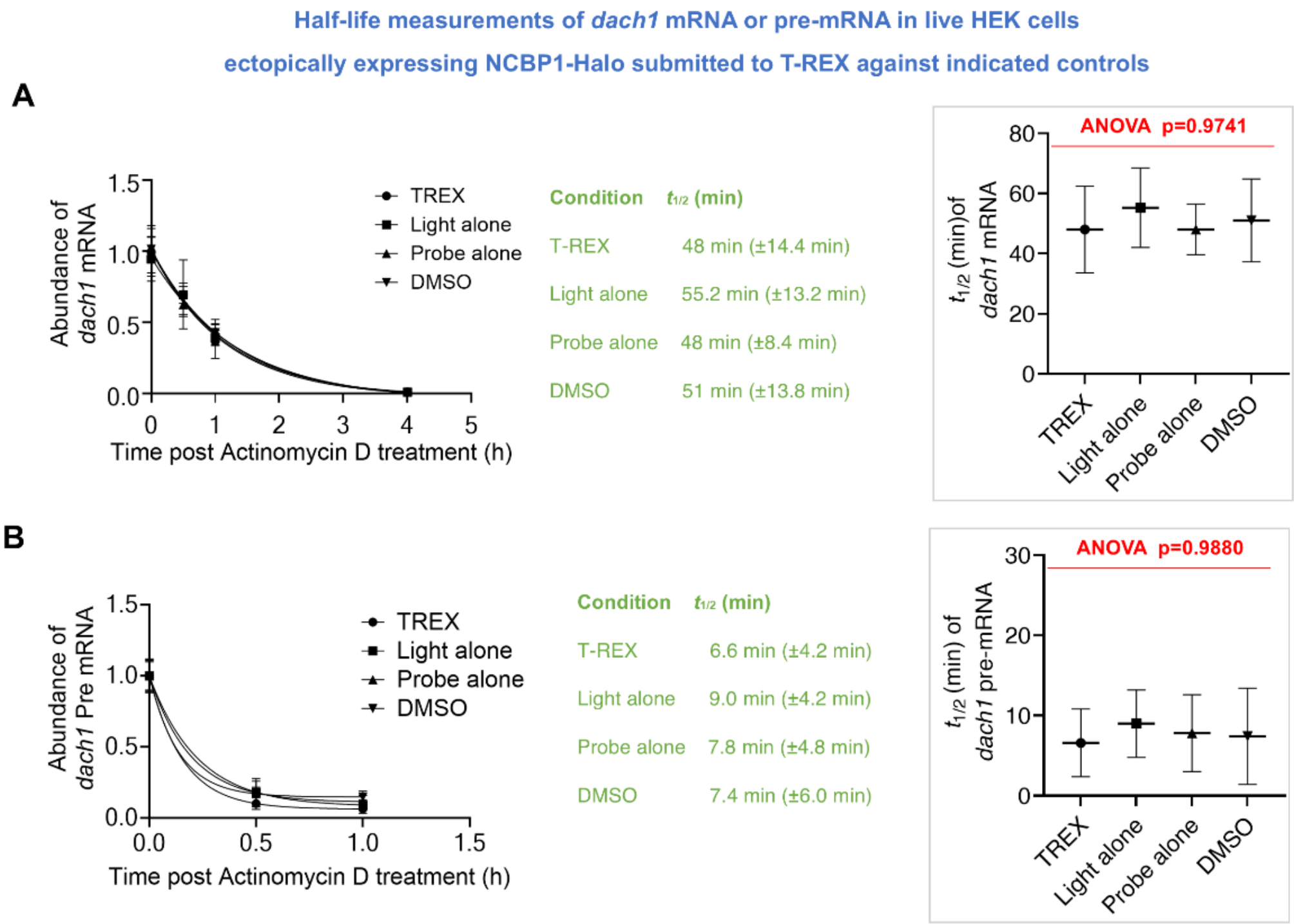

Supplementary Fig. 23. NCBP1-HNEylation does not significantly perturb the half-life of *dach1*-pre-mRNA and -mRNA HEK293T expressing NCBP1-Halo were exposed to T-REX and all other three controls, then Actinomycin D (5 µg/mL) was incubated into cells for the time period indicated (see detail protocol in **Methods**). RNA was extracted and the initial level of mRNA or pre-mRNA was set at 1, and data for other time points were normalized against that from time zero. Data were fit to one-phase exponential decay: y = (1-Plateau)*exp (-*k*x) + Plateau, where y is the RNA transcript levels, *k* is the rate constant, and x is the time, using Prim v9.4.0. **A)** results for *dach1*-pre-mRNA **B)** results for *dach1*-mRNA. $t_{1/2}$ of pre-mRNA decay shown in **A**) ($t_{1/2}$ under T-REX is 6.6 min (±4.2 min), $t_{1/2}$ under Light alone is 9.0 min (±4.2 min), $t_{1/2}$ under Probe alone is 7.8 min (±4.8 min), $t_{1/2}$ of DMSO is 7.4 min (±6.0 min). $t_{1/2}$ of mRNA decay shown in **B**) ($t_{1/2}$ under T-REX is 48 min (±14.4 min), $t_{1/2}$ under Light alone is 55.2 min (±13.2 min), $t_{1/2}$ under Probe alone is 48 min (±8.4 min), $t_{1/2}$ of DMSO is 51 min (±13.8 min). *p* values were calculated withTukey's multiple comparison test (*n*=9). All data present mean ± SEM.

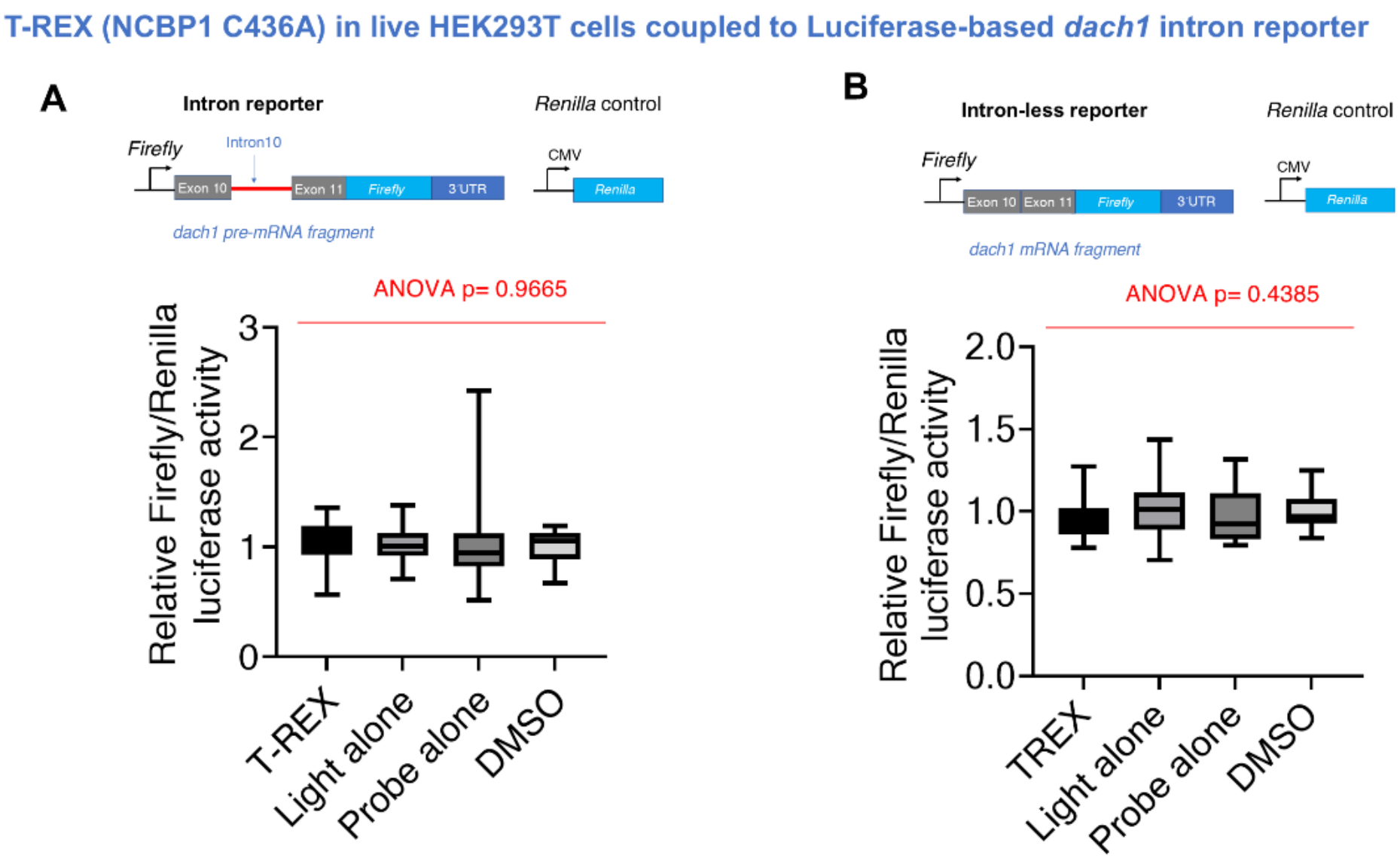

Supplementary Fig. 24. NCBP1(C436A), HNE-signaling-dead mutant, is recalcitrant to NCBP1-HNEylation-induced perturbation of DACH1 splicing observed with NCBP1(wt). See also **Fig. 4B-D.** The experimental setup was identical to that in **Fig. 4D** expect that HEK293T cells were transfected with NCBP1(C436)-Halo in place of NCBP1(wt)-Halo. **A)** Schematic of the *firefly* luciferase intron reporter was used, and **B)** Schematic of the *firefly* luciferase intron-less reporter was used. *p* values were calculated using ANOVA and Tukey's multiple comparison test (*n*=18). All data present mean±SEM. Box and whisker plots: line-median; box-25-75 percentiles; whiskers-1-99 percentiles.

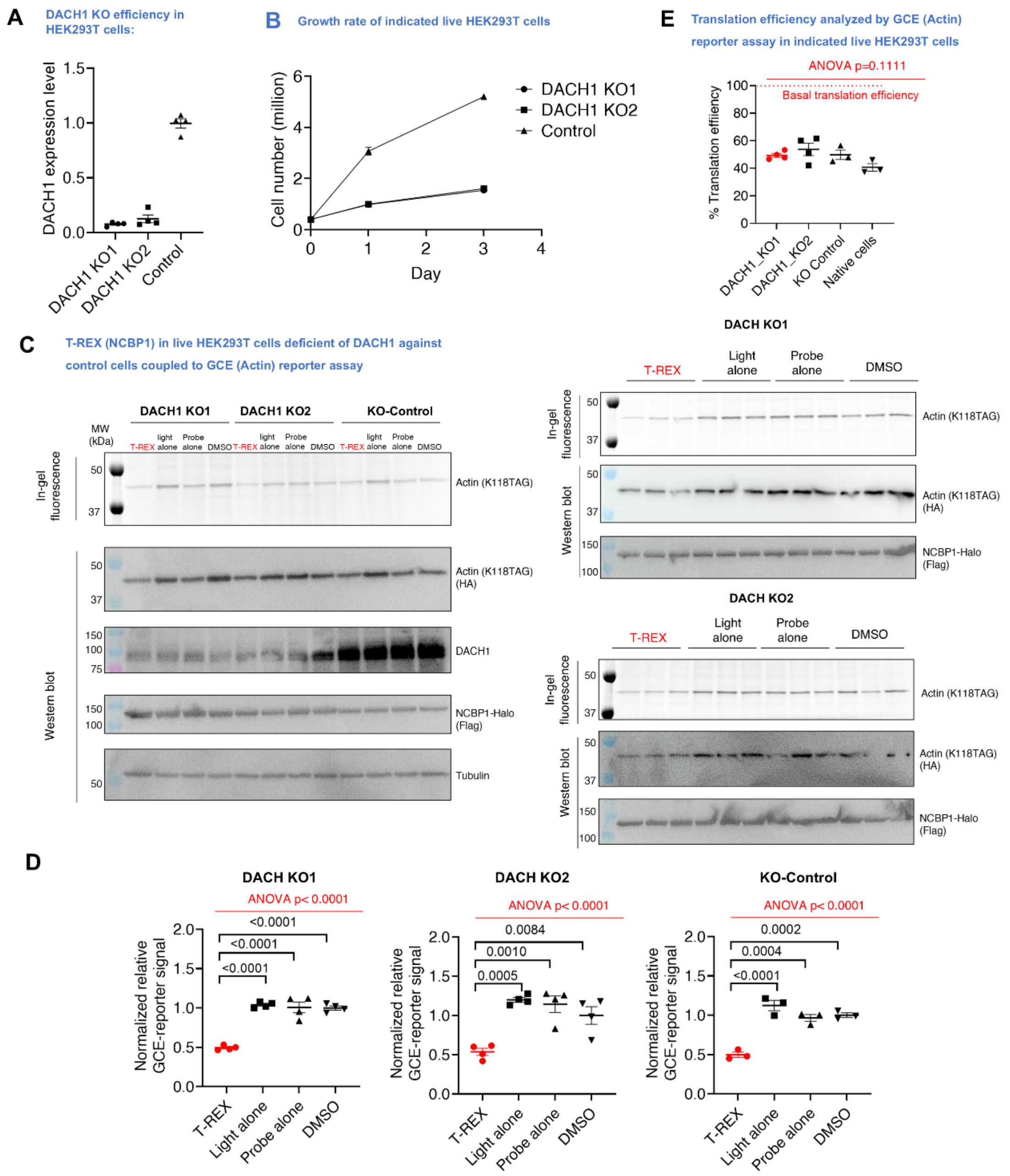

Supplementary Fig. 25. HNEylation of NCBP1 downregulates protein translation independent of DACH1. A) Two different DACH1 knockout (KO) and KO-control HEK293T lines were generated as described in **Methods**. KO efficiencies (92 ± 1% for KO line 1 and 88 ± 4% for KO line 2) were analyzed by western blot using anti-DACH1 antibody (showed in **B**). Tubulin served as loading control. n=4. **B)** HEK293T deficient of DACH1 grew slower compared to KO control. 0.4 Million HEK293T with DACH1 KO1, KO2 or control cells were seeded in individual 60 mm dish. The cells were harvested and counted using Countess II Cell Counter (ThermoFisher) at the indicated time. n=3. **C)** Representative gels and blots from T-REX–GCE integrated assay (see **Methods** and **Supplementary Fig. 1E**) conducted in DACH1 KO 1, 2 and KO-control lines**. D)** Data quantification is shown in **B**. *p* values were calculated using Tukey's multiple comparison test (n=4,4,3). **E)** Relative extent of translation efficiency under T-REX normalized to DMSO control. Data of native cells are derived from **Fig. 2F** (DMSO condition). 100% on the

y-axis designates the basal translation efficiency. *p* values were calculated using Tukey's multiple comparison test. All data present mean±SEM. *n*=4,4,3,3 independent expreiments.

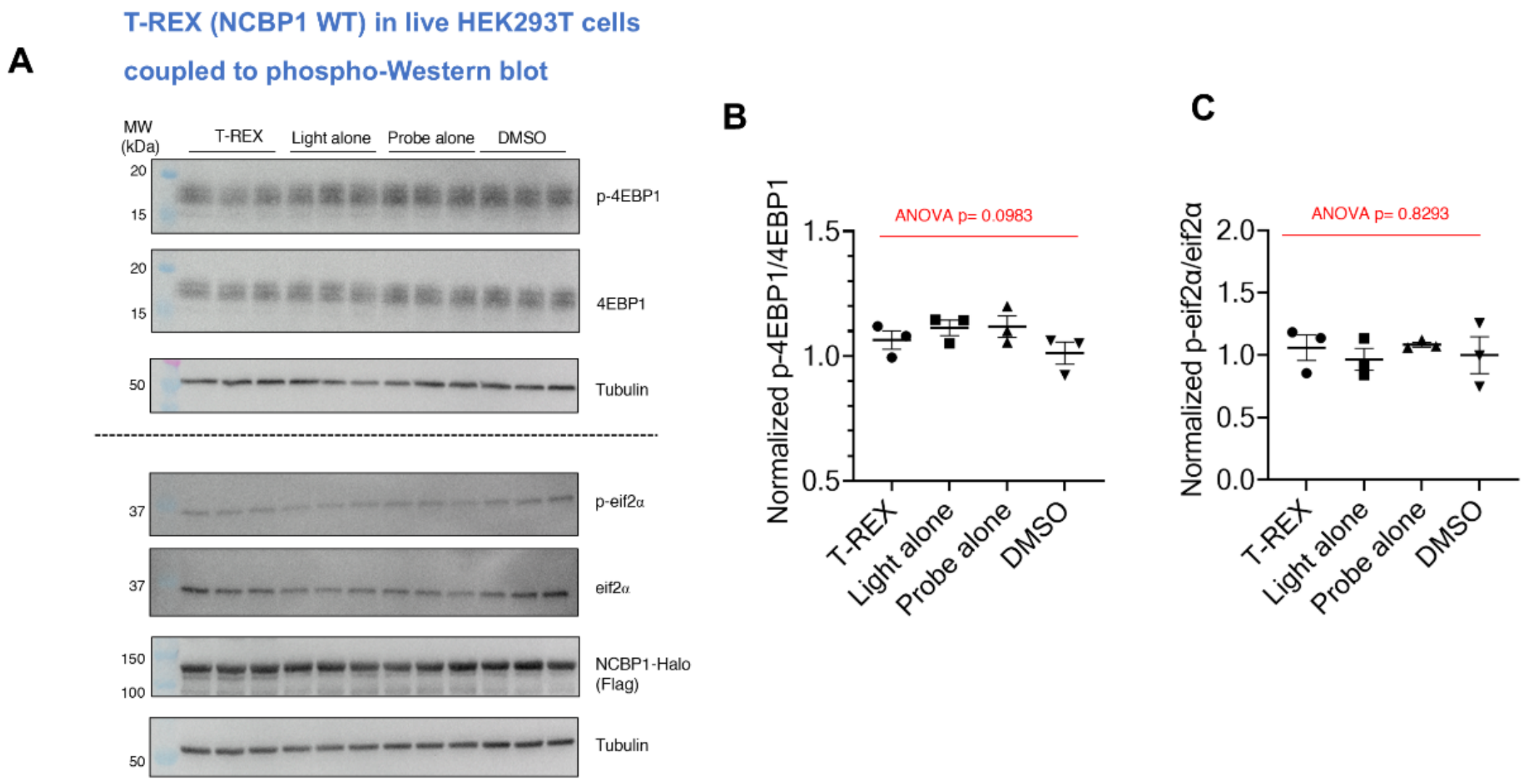

Supplementary Fig. 26. NCBP1-HNEylation does not affect phosphorylation of endogenous 4EBP1 and eIF2α. The expression levels of endogenous 4EBP1 and eIF2α, and those of the respective phosphorylated proteins, were analyzed 2 h post T-REX in HEK293T expressing NCBP1-Halo, against indicated controls. Post cell lysis, indicated antibodies were used to perform western blot analyses. See **Methods** for detailed lysis conditions to preserve phosphorylation states of proteins. **A)** Representative western blots: 4EBP1 (*top*); eIF2α (*bottom*). **B-C)** Quantification. *p* values were calculated using Tukey's multiple comparison test (*n*=3). All data present mean±SEM.

Sequence alignment of S6K1-isoform 1 and isoform X

isoform 1	MRRRRRRDGFYPAPDFRDREAEDMAGVFDIDLDQPEDAGSEDELEEGGQ	1-49
isoform X	MRRRRRRDGFYPAPDFRDREAEDMAGVFDIDLDQPEDAGSEDELEEGGQ	1-49
isoform 1	LNESMDHGGVGPYELGMEHCEKFEISETSVNRGPEKIRPECFELLRVLGK	50-99
isoform X	LNESMDHGGVGPYELGMEHCEKFEISETSVNRGPEKIRPECFELLRVLGK	50-99
isoform 1	GGYGKVFQVRKVTGANTGKIFAMKVLKKAMIVRNAKDTAHTKAERNILEE	100-149
isoform X	GGYGKVFQVRKVTGANTGKIFAMKVLKKAMIVRNAKDTAHTKAERNILEE	100-149
isoform 1	VKHPFIVDLIYAFQTGGKLYLILEYLSGGELFMQLEREGIFMEDTACFYLAE	150-201
isoform X	VKHPFIVDLIYAFQTGGKLYLILEYLSGGELFMQLEREGIFMEDTACFYLAE	150-201
isoform 1	ISMALGHLHQKGIIYRDLKPENIMLNHQGHVKLTDFGLCKESIHDGTVTHT	202-252
isoform X	ISMALGHLHQKGIIYRDLKPENIMLNHQGHVKLTDFGLCKESIHDGTVTHT	202-252
isoform 1	FCGTIEYMAPEILMRSGHNRAVDWWSLGALMYDMLTGAPPFTGENRKKTI	253-302
isoform X	FCGTIEYMAPEILMRSGHNRAVDWWSLGALMYDMLTGAPPFTGENRKKTI	253-302
	Coded by Exon 11	
isoform 1	DKILKCKLNLPPYLTQEARDLLKKLLKRNAASRLGAGPGDAGEVQAHPFF	303-352
isoform X	DKILKCKLNLPPYLTQEARDLLKKLLKRNAASRLGAGPGDAGEVQAHPFF	303-352
	Coded by Exon 12 / Coded by Exon 13	
isoform 1	RHINWEELLARKVEPPFKPLLQSEEDVSQFDSKFTRQTPVDSPDDSTLSE	353-402
isoform X	RHINWEELLARKVEPPFKPLLVHHPDLCLHVHMVFYLCAYLWPNFPFL	353-400
	Coded by Exon 14	
isoform 1	SANQVFLGFTYVAPSVLESVKEKFSFEPKIRSPRRFIGSPRTPVSPVKFSP	403-453
	Coded by Exon 15	
isoform 1	GDFWGRGASASTANPQTPVEYPMETSGIEQMDVTMSGEASAPLPIRQPN	454-502
isoform 1	SGPYKKQAFPMISKRPEHLRMNL	503-525

Supplementary Fig. 27. Alignment of amino acid sequences of S6K1 full length (FL) and S6K1-isoform X Sequences coded by exons 11, 12, 13, 14, 15 are indicated using lines of different color. The sequence at the C-terminus of isoform X, which is different from isoform 1 due to alternative splicing, is highlighted in red. **Note:** Kinase domain and ATP-binding site within S6K1-**FL** remained intact in S6K1-**X**, and so did the mTORC1 stimulatory phosphorylation site (T389, highlighted in yellow).

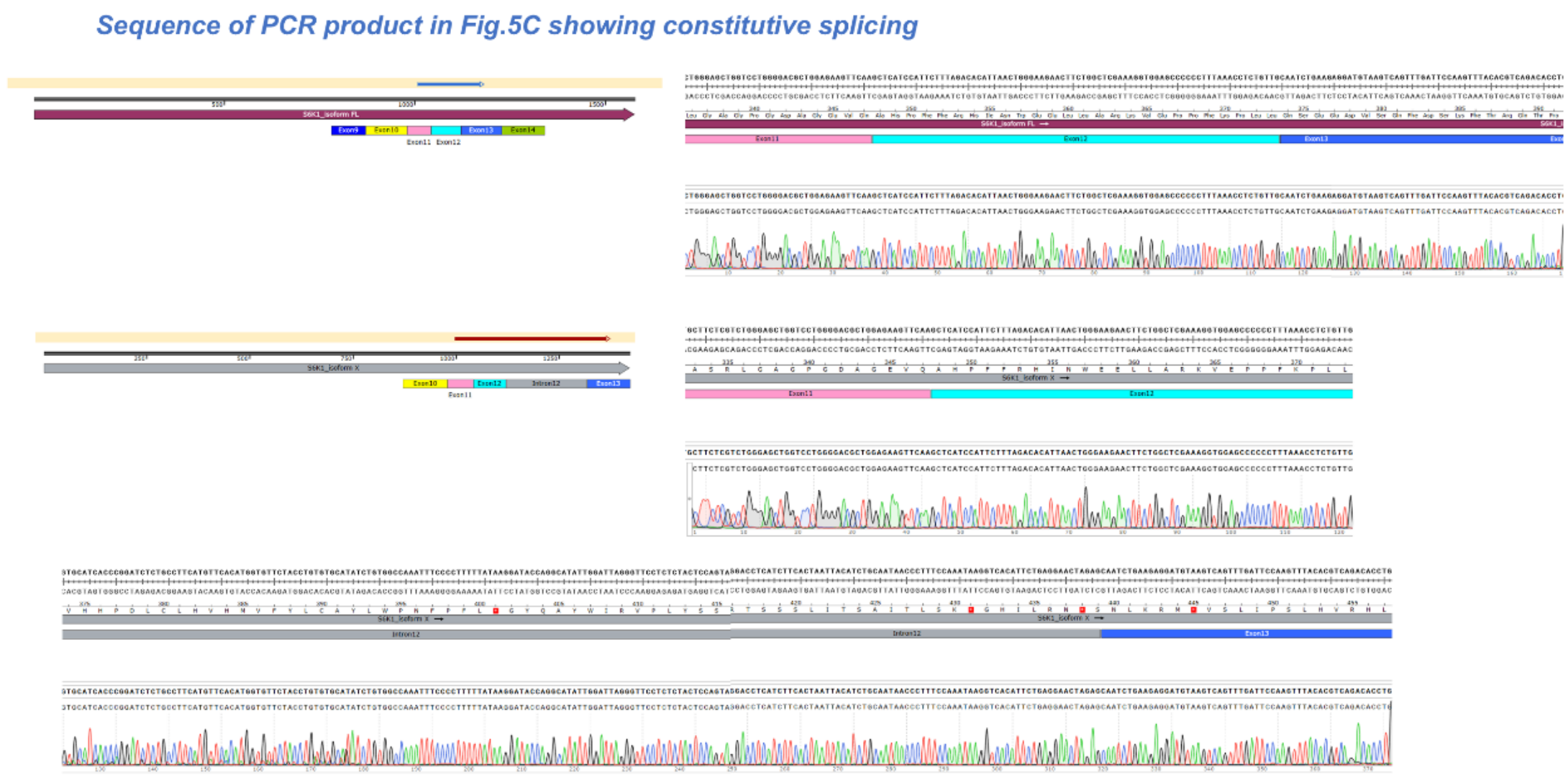

Supplementary Fig. 28. (Note: raw sequencing output data in this figure are split over two pages for clarity). Relating to datasets in Fig. 5C-D, sequencing validation of S6K1 full length (FL) and isoform X. The requisite amplicons (the lower band in the gel in **Fig. 5C from S6K1 FL** and the upper band in the gel in **Fig. 5C from S6K1 isoform X**) were excised and gel-extracted (A9285, Promega), and sequenced using the corresponding forward primer used in the experiment that gave rise to the dataset shown in **Fig. 5C-D**. The sequencing results covering regions E11—E12—E13 are shown.

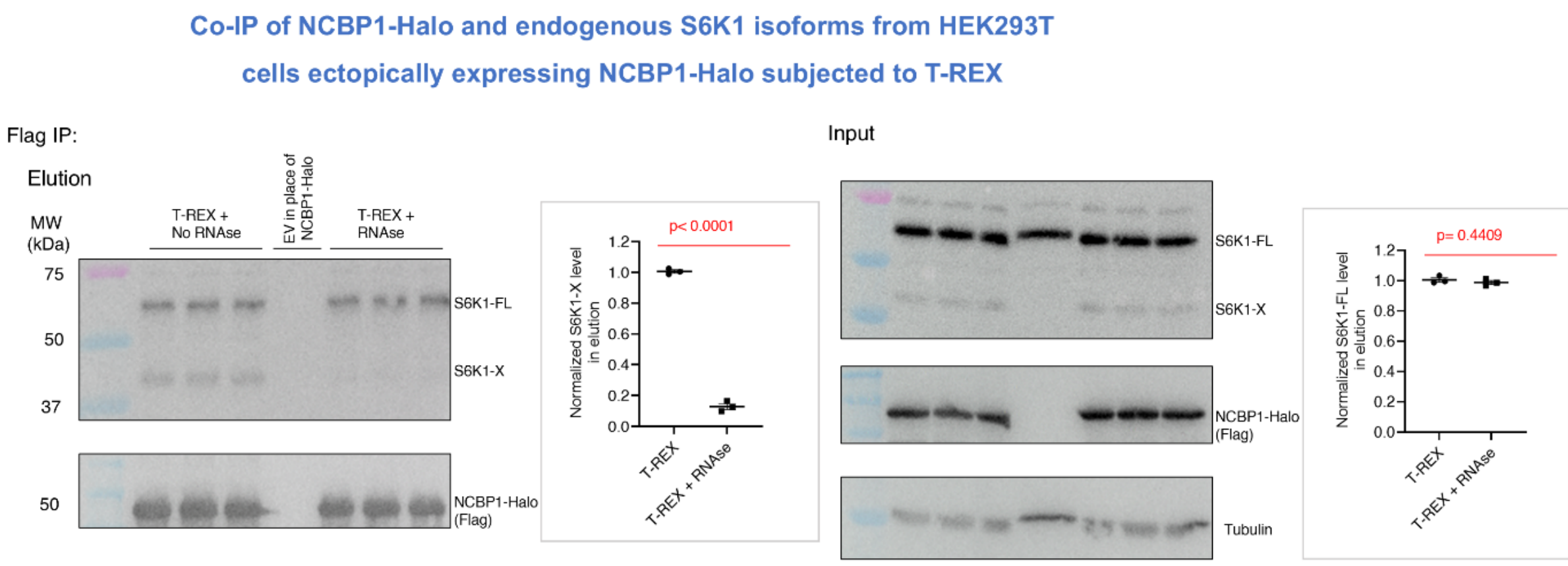

Supplementary Fig. 29. S6K1-X–NCBP1 interaction, but not S6K1–NCBP1 interaction, is RNA dependent. The live-cell-based T-REX experiments were performed similarly as described in **Fig. 5E**, except that post lysis, samples were treated with either RNase A (100 ug/mL) or buffer alone and incubated (30 min, 37°C), prior to further incubation with Flag beads. *Insets* show quantification *p* values were calculated with an unpaired, two-tailed t-test (n=3). All data present mean ±SEM.

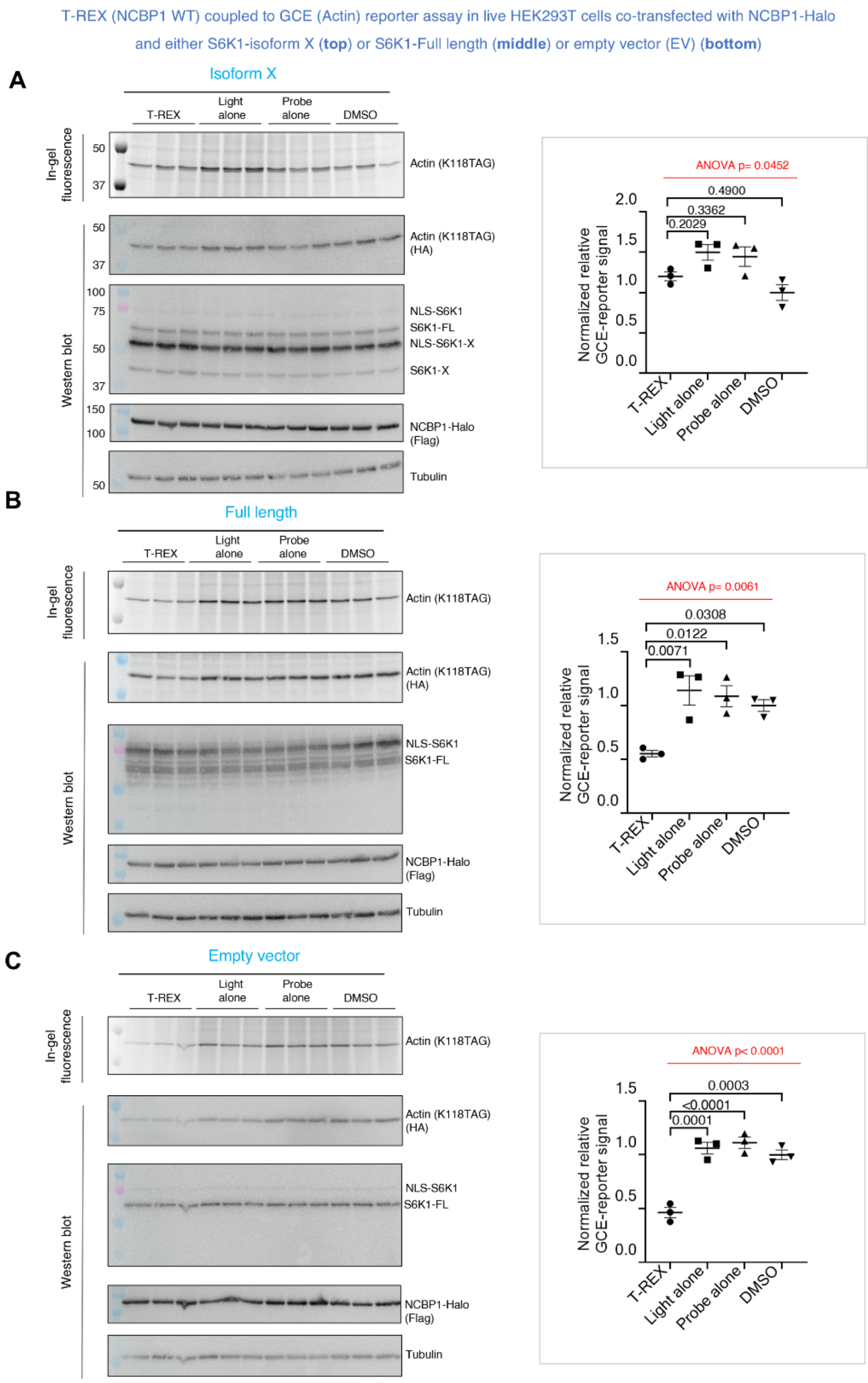

Supplementary Fig. 30. Effects of overexpression of full-length S6K1 and truncated isoform S6K1-X on NCBP1-HNEylation-induced suppression of protein translation. HEK293T were co-transfected with plasmids encoding either **(A)** S6K1-isoform X, **(B)** S6K1-full length (FL), or **(C)** empty vector (EV), and GCE-reporter constructs as described in **Methods** and **Supplementary Fig. 1E** for T-REX—GCE integrated workflow. The ratio of plasmids of S6K1-FL or S6K1-X (or EV): NCBP1-Halo : Actin (K118TAG) : tRNA synthetase is 1:1:1:0.2. The cells were subjected to T-REX against indicated controls, followed by treatment with BCNK (50 µM, 4 h). Post cell lysis, and coupling with tertrazine-Cy5, the samples were analyzed by in-gel fluorescence and western blot analyses using indicated

antibodies. *Insets* in each case show respective quantification. p values were calculated using Tukey's multiple comparison test (*n*=3). All data present mean±SEM.

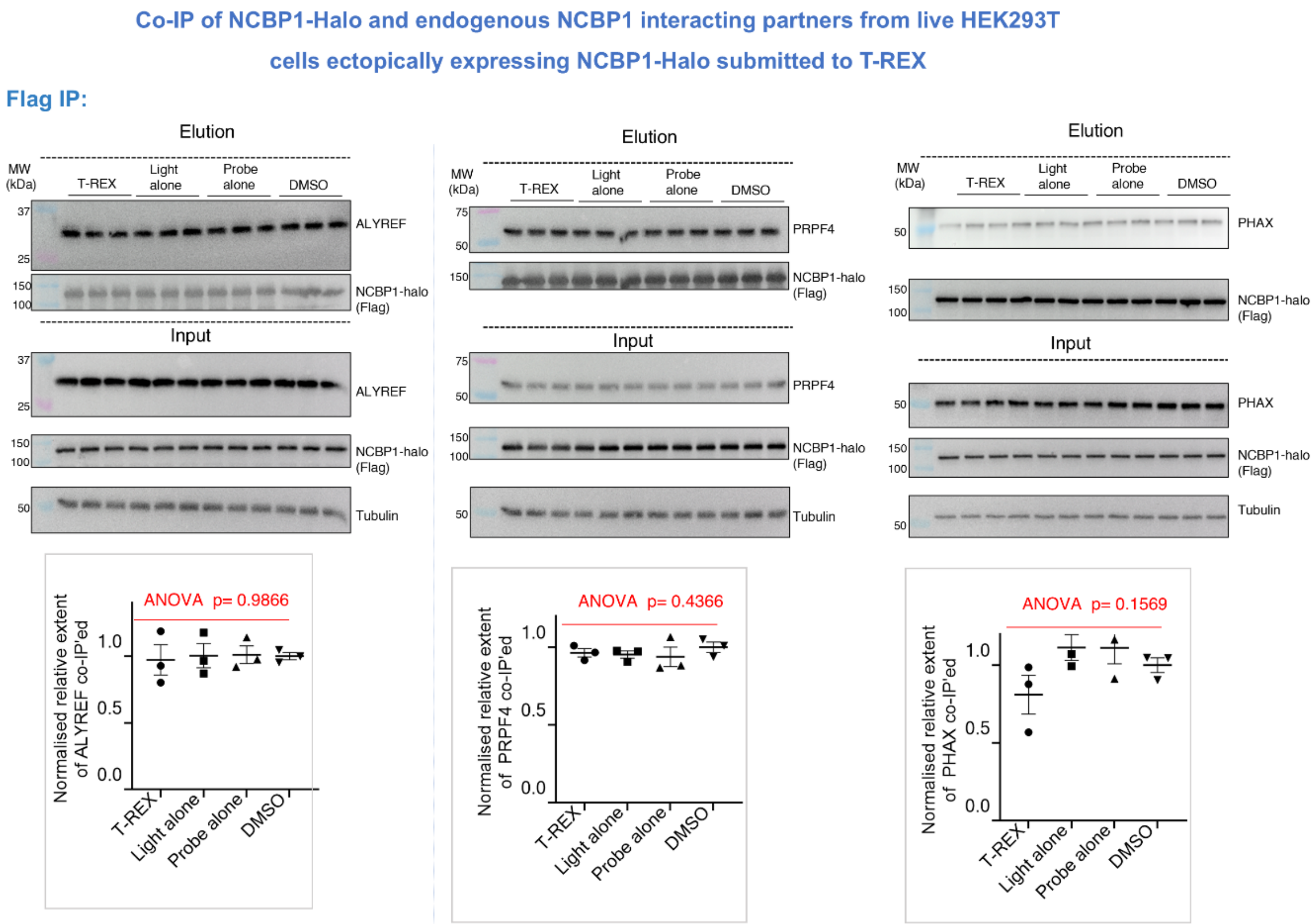

Supplementary Fig. 31. Relating to Fig. 6B, investigating the possible HNE-dependent effects on association between NCBP1 and either ALYREF, PRPF4, or PHAX shows no statistically significant change in association upon NCBP1-HNEylation. The experiment was performed similarly to **Fig. 6B**. Following T-REX-enabled NCBP1-HNEylation in HEK293T (see **Methods** for details) expressing NCBP1-Flag-Halo, against indicated T-REX technical controls, Anti-Flag pulldown (see **Methods** for details) was performed. Input and elution samples were subjected to SDS-PAGE, and western blot analyses using indicated antibodies. *Right panel* quantification of the relative extent of ALYREF, PRPF4, or PHAX co-eluted with NCBP1-Flag-Halo in the absence and presence of NCBP1-HNEylation. Data were normalized by NCBP1-Halo expression. *p* values were calculated using ANOVA and Tukey's multiple comparison test (*n*=3). All data present mean±SEM.

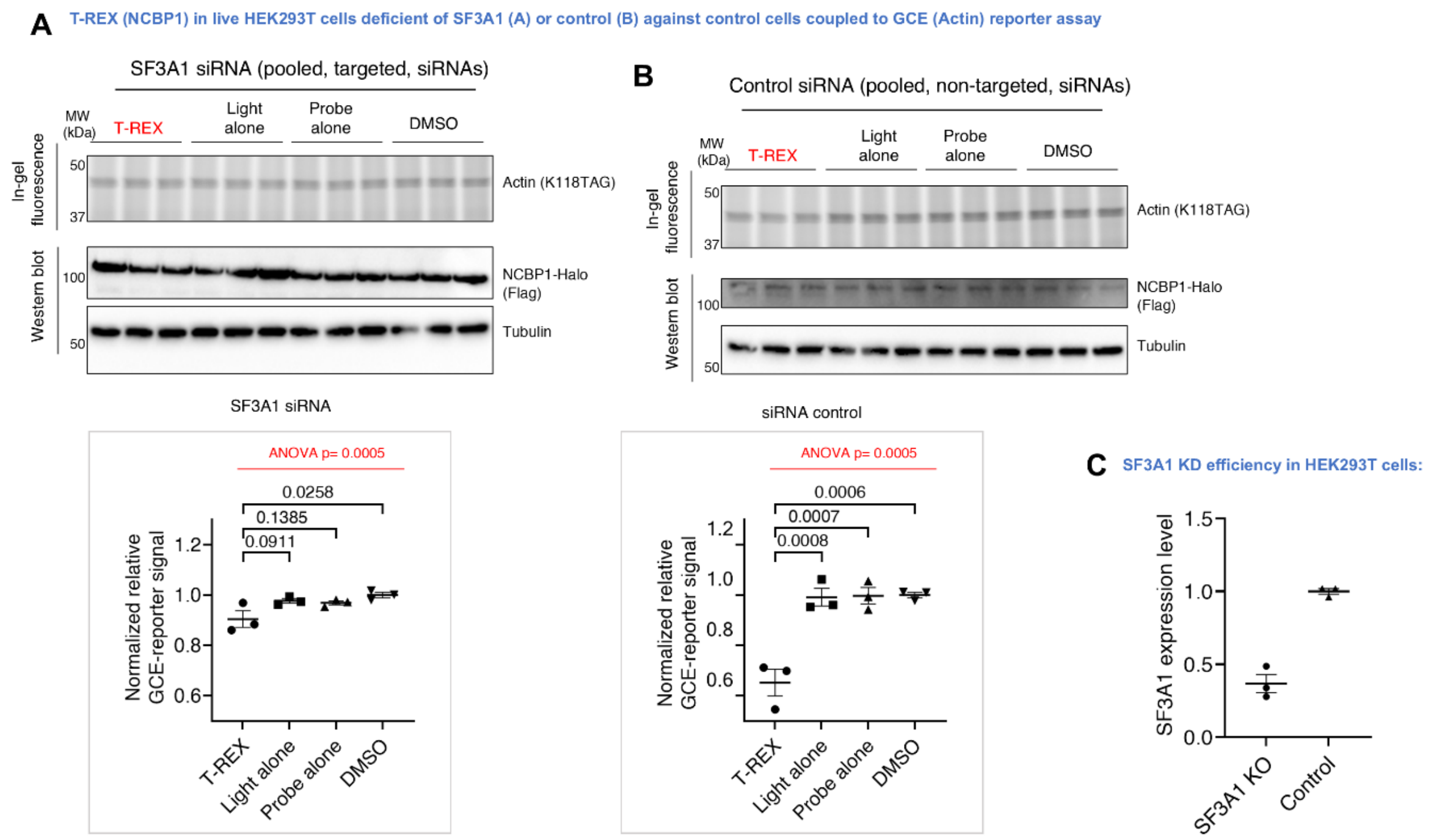

Supplementary Fig. 32. SF3A1 depletion ablated translation inhibition driven by NCBP1-HNEylation. HEK293T co-transfected with NCBP1-Halo and GCE(actin) reporter constructs were subjected to either SF3A1 siRNA (**A**), or non-targeted control siRNA (**B**), using DharmaFECT Duo Transfection Reagent following manufacturer's protocol. T-REX-GCE assay was performed as described in **Methods**. Data quantification is shown in *Right panel*. *p* values were calculated using Tukey's multiple comparison test (n=3). All data present mean±SEM. **C)** KO efficiency of SF3A1 (63 ± 7%) analyzed by western blot using anti-SF3A1 antibody (also see **Fig. 6C**).

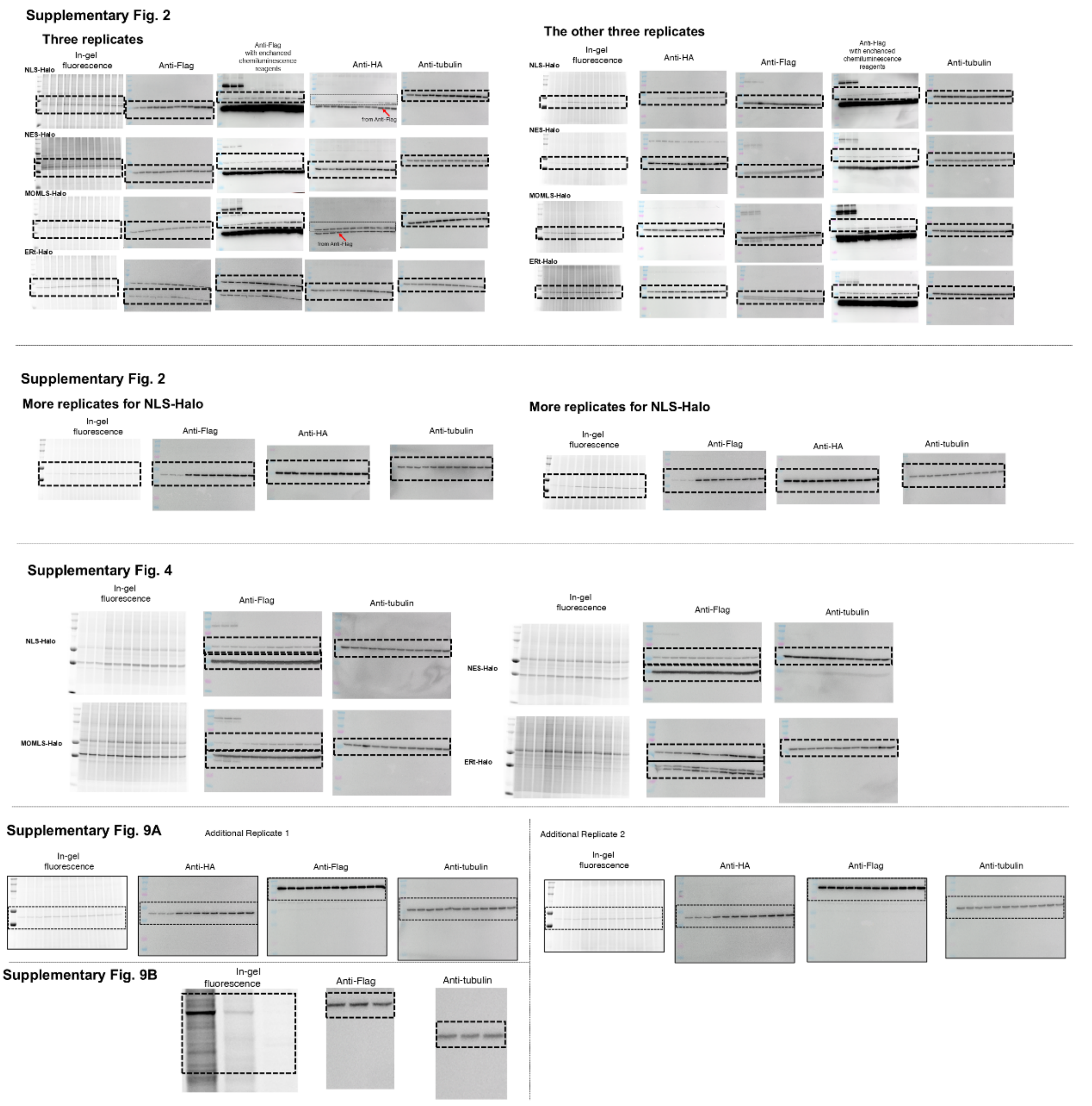

Supplementary Fig. 33. Unprocessed western Blots for Supplementary Figures.

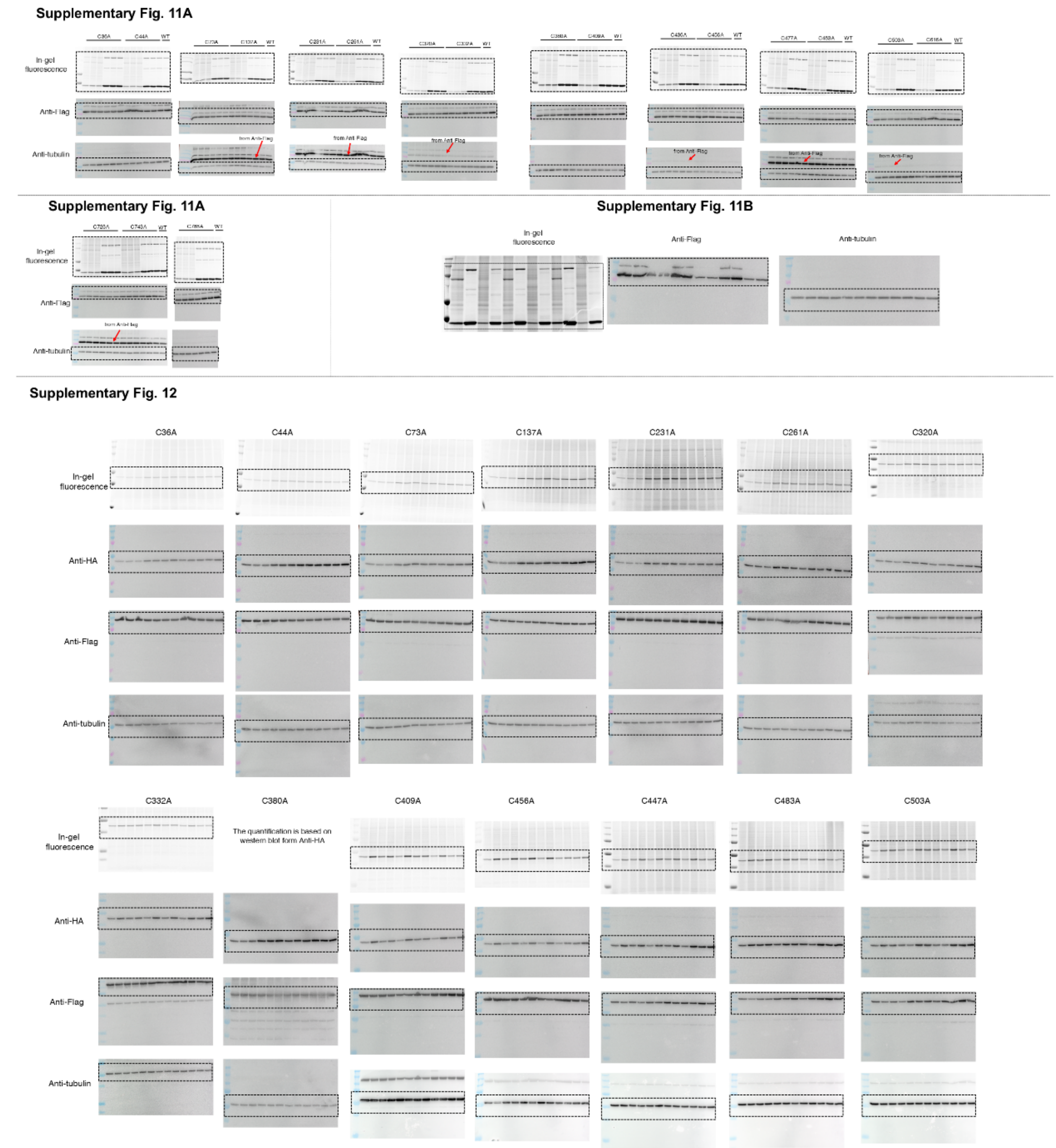

Supplementary Fig. 34. Unprocessed western Blots for Supplementary Figures.

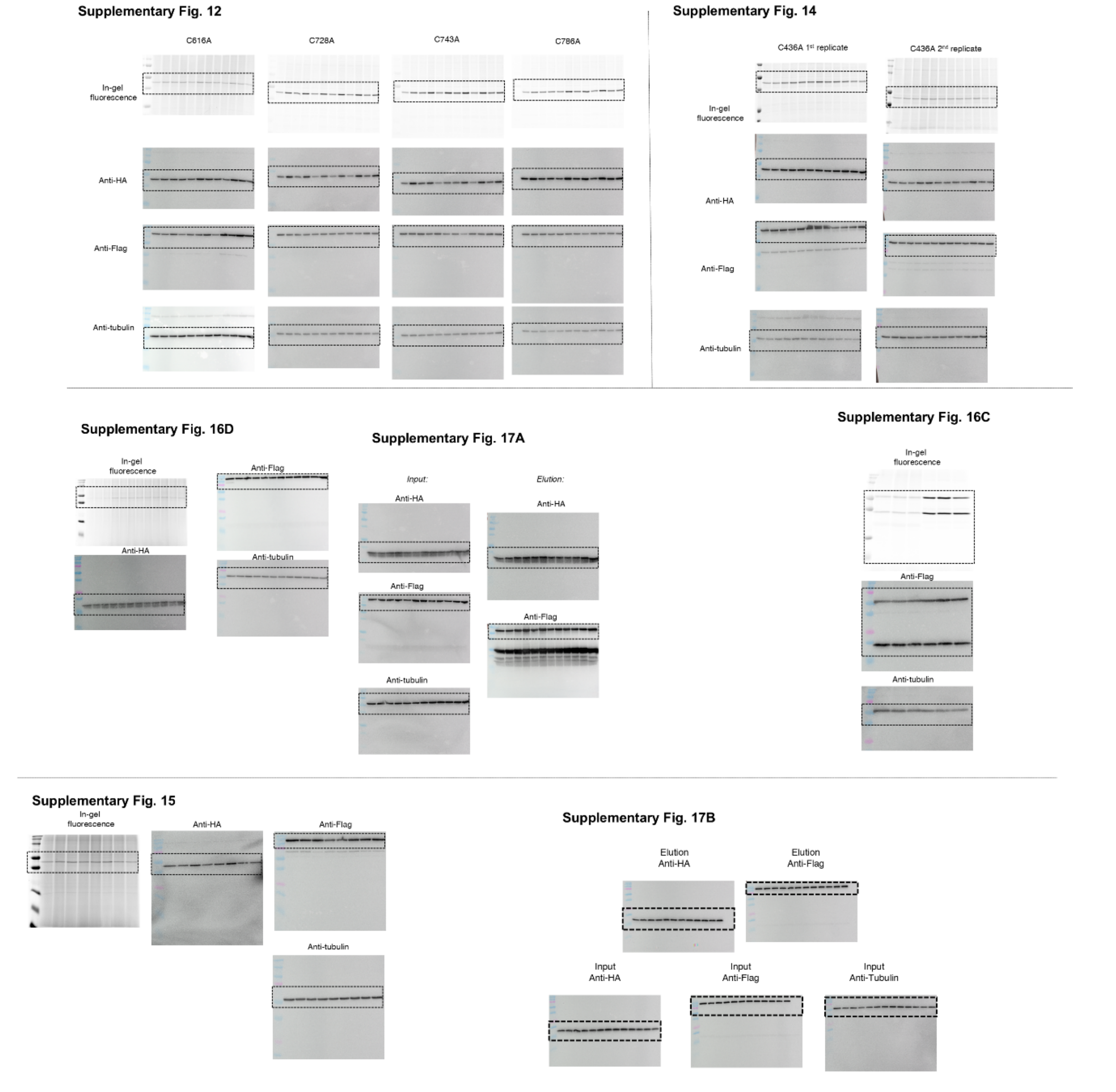

Supplementary Fig. 35. Unprocessed western Blots for Supplementary Figures.

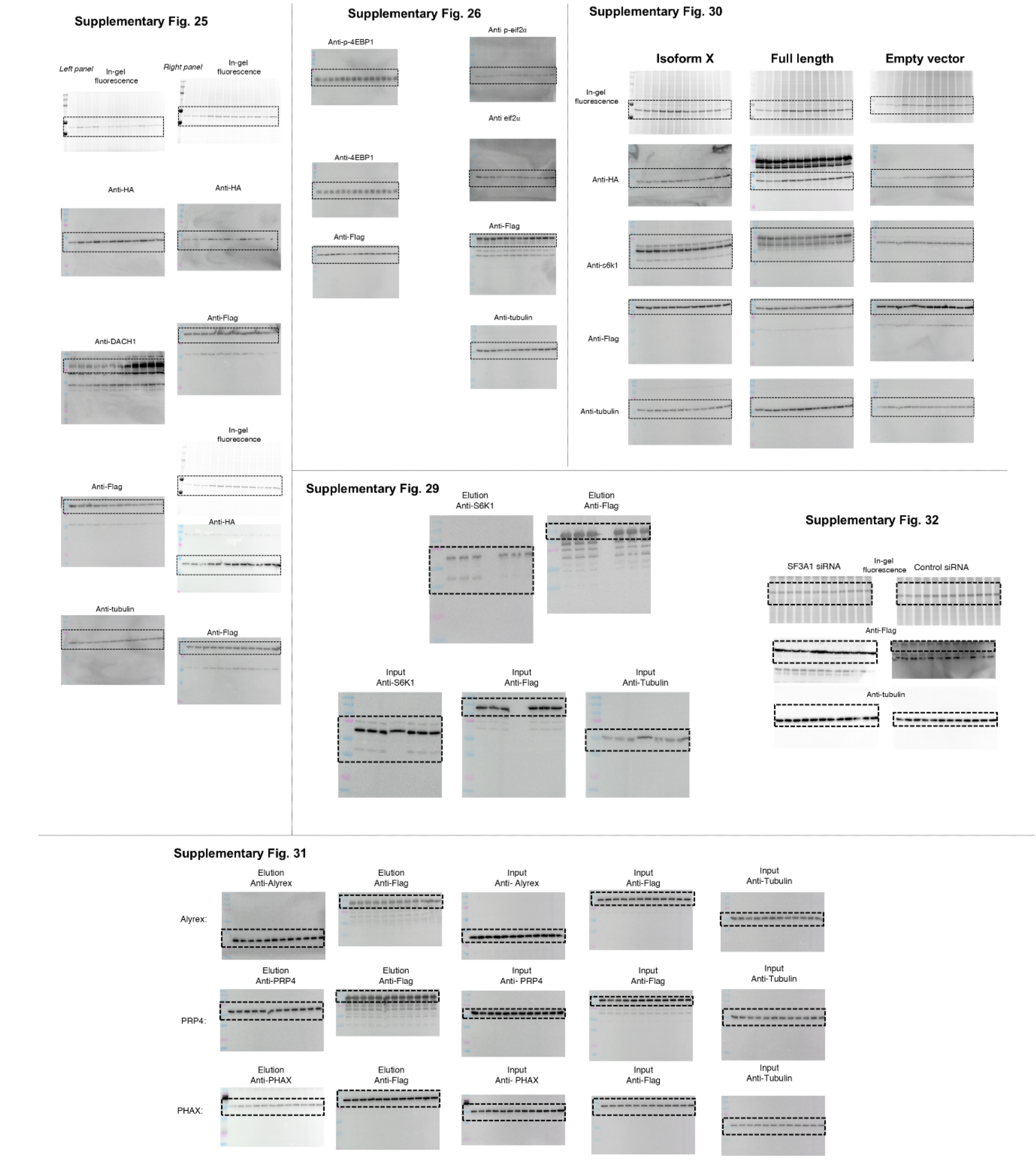

Supplementary Fig. 36. Unprocessed western Blots for Supplementary Figures.
