# Supplementary material for: NCBP1 stress signaling drives alternative S6K1 splicing inhibiting translation
Source: Nat Chem Biol. 2026 Feb 10;22(10):1628–41. doi: 10.1038/s41589-025-02135-4 (PMC13612227; doi:10.1038/s41589-025-02135-4)
Supplement: Supplementary file 2 [file 41589_2025_2135_MOESM2_ESM.pdf]

nature research

Corresponding author(s): Marcus J. C. Long, Yimon Aye
Last updated by author(s): 29th Nov 2025

# Reporting Summary

Nature Research wishes to improve the reproducibility of the work that we publish. This form provides structure for consistency and transparency in reporting. For further information on Nature Research policies, see Authors & Referees and the Editorial Policy Checklist.

## Statistics

For all statistical analyses, confirm that the following items are present in the figure legend, table legend, main text, or Methods section.

| n/a | Confirmed | |
|---|---|---|
| ☐ | ☒ | The exact sample size (*n*) for each experimental group/condition, given as a discrete number and unit of measurement |
| ☐ | ☒ | A statement on whether measurements were taken from distinct samples or whether the same sample was measured repeatedly |
| ☐ | ☒ | The statistical test(s) used AND whether they are one- or two-sided<br>*Only common tests should be described solely by name; describe more complex techniques in the Methods section.* |
| ☒ | ☐ | A description of all covariates tested |
| ☐ | ☒ | A description of any assumptions or corrections, such as tests of normality and adjustment for multiple comparisons |
| ☐ | ☒ | A full description of the statistical parameters including central tendency (e.g. means) or other basic estimates (e.g. regression coefficient) AND variation (e.g. standard deviation) or associated estimates of uncertainty (e.g. confidence intervals) |
| ☐ | ☒ | For null hypothesis testing, the test statistic (e.g. $F$, $t$, $r$) with confidence intervals, effect sizes, degrees of freedom and $P$ value noted<br>*Give P values as exact values whenever suitable.* |
| ☒ | ☐ | For Bayesian analysis, information on the choice of priors and Markov chain Monte Carlo settings |
| ☒ | ☐ | For hierarchical and complex designs, identification of the appropriate level for tests and full reporting of outcomes |
| ☒ | ☐ | Estimates of effect sizes (e.g. Cohen's *d*, Pearson's *r*), indicating how they were calculated |

*Our web collection on statistics for biologists contains articles on many of the points above.*

## Software and code

Policy information about availability of computer code

| | |
|---|---|
| Data collection | Evolution capture/Fusion FX6 Edge (Vilbur) software for western blots; NIS Element AR (Nikon) for all imaging experiments; Gen5 2.06 (BioTek) for all microplate assays; QuantStudio-7 Pro for qRT-PCR; Nextseq System Suite (Illumina) for RNA-seq |
| Data analysis | ImageJ for all western blot and fluorescence quantification; Microsoft Excel for general data processing; Graphpad Prism 10 for statistical analysis and data presentation; Maxquant (MaxLFQ algorithm) for LFQ-MS time-resolved proteomics; cutadapt v1.8, tophat v2.1, cufflinks v2.2 (cuffnorm/cuffdiff) for time-resolved RNA-Seq; SUPPA2 and Salmon for differential splicing analysis as described in Methods |

For manuscripts utilizing custom algorithms or software that are central to the research but not yet described in published literature, software must be made available to editors/reviewers. We strongly encourage code deposition in a community repository (e.g. GitHub). See the Nature Research guidelines for submitting code & software for further information.

## Data

Policy information about availability of data

All manuscripts must include a data availability statement. This statement should provide the following information, where applicable:

- Accession codes, unique identifiers, or web links for publicly available datasets
- A list of figures that have associated raw data
- A description of any restrictions on data availability

Proteomics data that support the findings of this study (Fig. 1D, 2B and 2C Supplementary Table 3) have been deposited to the ProteomeXchange Consortium via the PRIDE partner repository with the dataset identifier PXD:051483. The RNA-seq raw data from this study used to generate Fig. 4A, 4E and 5A, Extended Data Fig. 9, Supplementary Fig. 18-21, and Supplementary Table 4-8 have been deposited in the Gene Expression Omnibus (GEO) database under accession code (GSE:265951).

# Field-specific reporting

Please select the one below that is the best fit for your research. If you are not sure, read the appropriate sections before making your selection.

☒ Life sciences ☐ Behavioural & social sciences ☐ Ecological, evolutionary & environmental sciences

For a reference copy of the document with all sections, see nature.com/documents/nr-reporting-summary-flat.pdf

# Life sciences study design

All studies must disclose on these points even when the disclosure is negative.

**Sample size**: Sample sizes were not predetermined by statistical methods. Sample sizes were chosen based on our experience and commonly accepted sample sizes for similar experiments and analyses in the field. Sample sizes for all data sets are clearly listed in Methods.

**Data exclusions**: No data were excluded.

**Replication**: All experiments were conducted with sufficient biological replicates (minimally n=3 independent biological replicates) to ensure rigor. Generally, the pathways we identified were confirmed with orthogonal approaches (e.g. genetic knockdown/knockout and pharmacological modulators of key proteins). Additionally, identified electrophile-protein responders were confirmed in two independent cell lines of mouse and human origins. Conserved importance of the discovered electrophile responsivity and site of electrophile sensing is shown using both mouse and human proteins empirically and further via phylogenetic analysis across numerous eukaryotic species.

**Randomization**: Experiments were set up with cells derived from a common stock and divided equally without bias. Treatment groups for all experiments were assigned randomly.

**Blinding**: The majority of data in this study is derived from western blotting or in gel fluorescence data. All replicates are included in the manuscript. For such data we do not believe that blinding is relevant. The REX-GCE work-flow as well as key findings have now been replicated by colleagues in other laboratories, including co-author student researchers based in VISTEC Institute, Thailand.

# Reporting for specific materials, systems and methods

We require information from authors about some types of materials, experimental systems and methods used in many studies. Here, indicate whether each material, system or method listed is relevant to your study. If you are not sure if a list item applies to your research, read the appropriate section before selecting a response.

## Materials & experimental systems

| n/a | Involved in the study | |
|---|---|---|
| ☐ | ☒ | Antibodies |
| ☐ | ☒ | Eukaryotic cell lines |
| ☒ | ☐ | Palaeontology |
| ☐ | ☒ | Animals and other organisms |
| ☒ | ☐ | Human research participants |
| ☒ | ☐ | Clinical data |

## Methods

| n/a | Involved in the study | |
|---|---|---|
| ☒ | ☐ | ChIP-seq |
| ☒ | ☐ | Flow cytometry |
| ☒ | ☐ | MRI-based neuroimaging |

## Antibodies

**Antibodies used**: Described in detail in Supplemental Table S8.

**Validation**: The vast majority of western blots in this paper studies ectopic HALO-FLAG-POI fusions. Specificity is demonstrated by the fact that no FLAG-specific band was observed without transfection, and that the FLAG band shifted molecular weight based on removal of the HALO tag by TeV protease treatment. For endogenous proteins, these were primarily validated by KO and showing reduction of the specific band, and this was consistent with the manufacturer's own data. All antibodies were validated by the respective manufacturers using lysates from multiple different cells lines and/or recombinant proteins. Please also see the table cited above that lists the details of all antibodies used.

## Eukaryotic cell lines

Policy information about cell lines

**Cell line source(s)**: HEK293T: ATCC (CRL-3216TM); Mouse Neuro 2A: ATCC (CCL-131TM).

**Authentication**: HEK293T cells were authenticated by ATCC as described on their website: http://www.lgcstandards-atcc.org/products/all/CRL-3216.aspx?geo_country=ch

| | |
|---|---|
| | Mouse Neuro 2A were authenticated by ATCC as described on their website: https://www.atcc.org/products/ccl-131 |
| Mycoplasma contamination | All cell lines consistently tested negative for mycoplasma contamination during all of our trimonthly mycoplasma tests (Venor GeM mycoplasma detection kit, Sigma). |
| Commonly misidentified lines (See ICLAC register) | HEK293T, Mouse Neuro 2A are not listed in the ICLAC register. |

## Animals and other organisms

Policy information about studies involving animals; ARRIVE guidelines recommended for reporting animal research

| | |
|---|---|
| Laboratory animals | N/A |
| Wild animals | N/A |
| Field-collected samples | N/A |
| Ethics oversight | N/A |

Note that full information on the approval of the study protocol must also be provided in the manuscript.
