# Supplementary material for: NCBP1 stress signaling drives alternative S6K1 splicing inhibiting translation
Source: Nat Chem Biol. 2026 Feb 10;22(10):1628–41. doi: 10.1038/s41589-025-02135-4 (PMC13612227; doi:10.1038/s41589-025-02135-4)

Source Data Fig. 1

Figure 1B

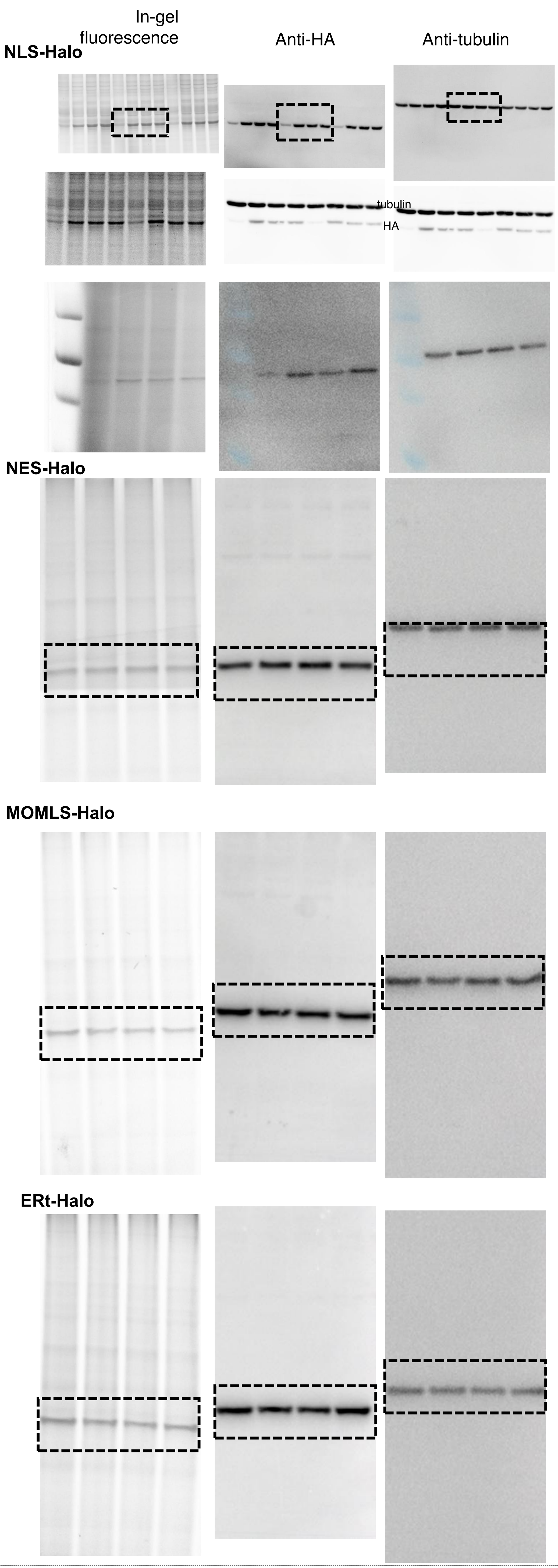

Figure 2B

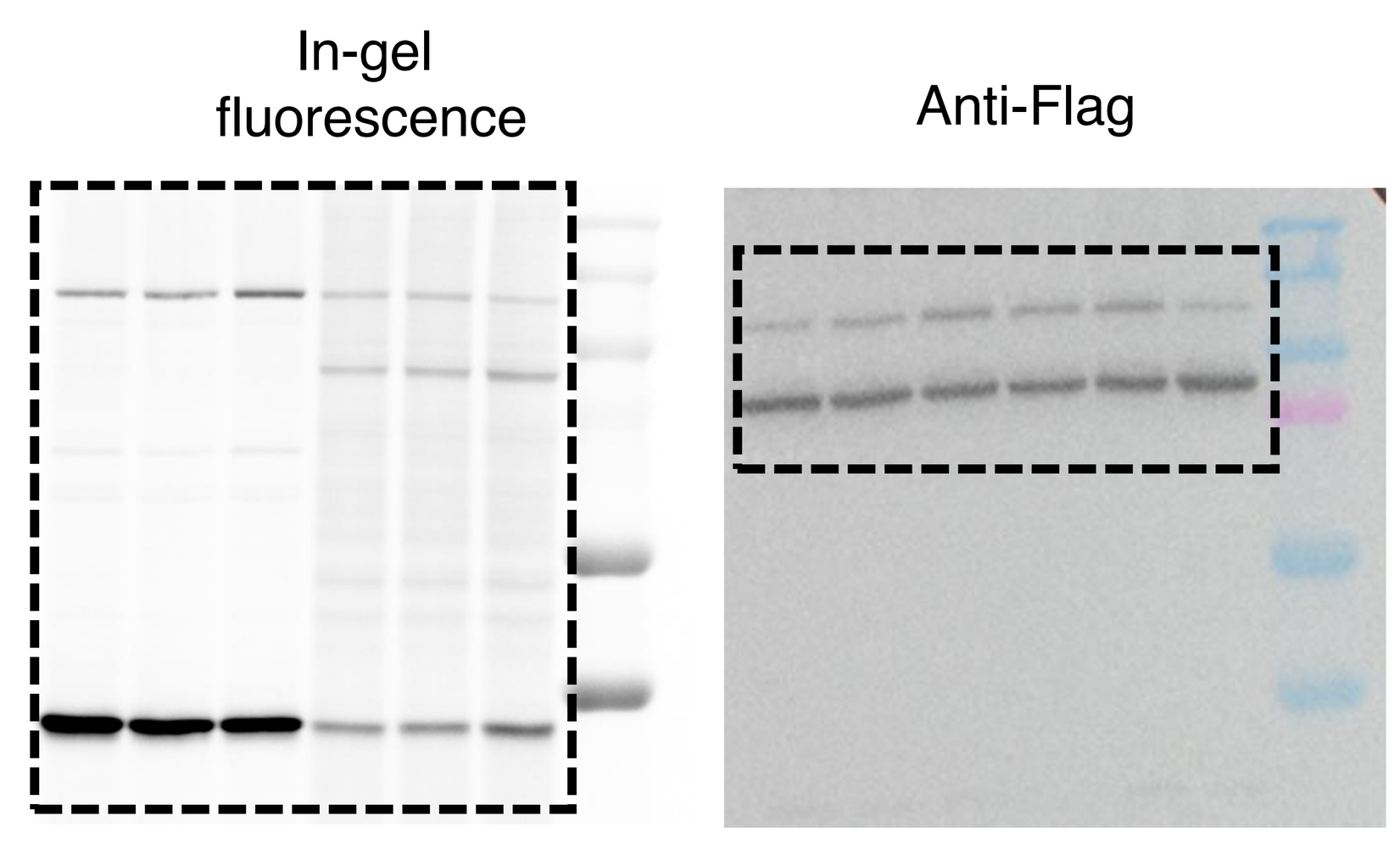

Figure 2C Cand1

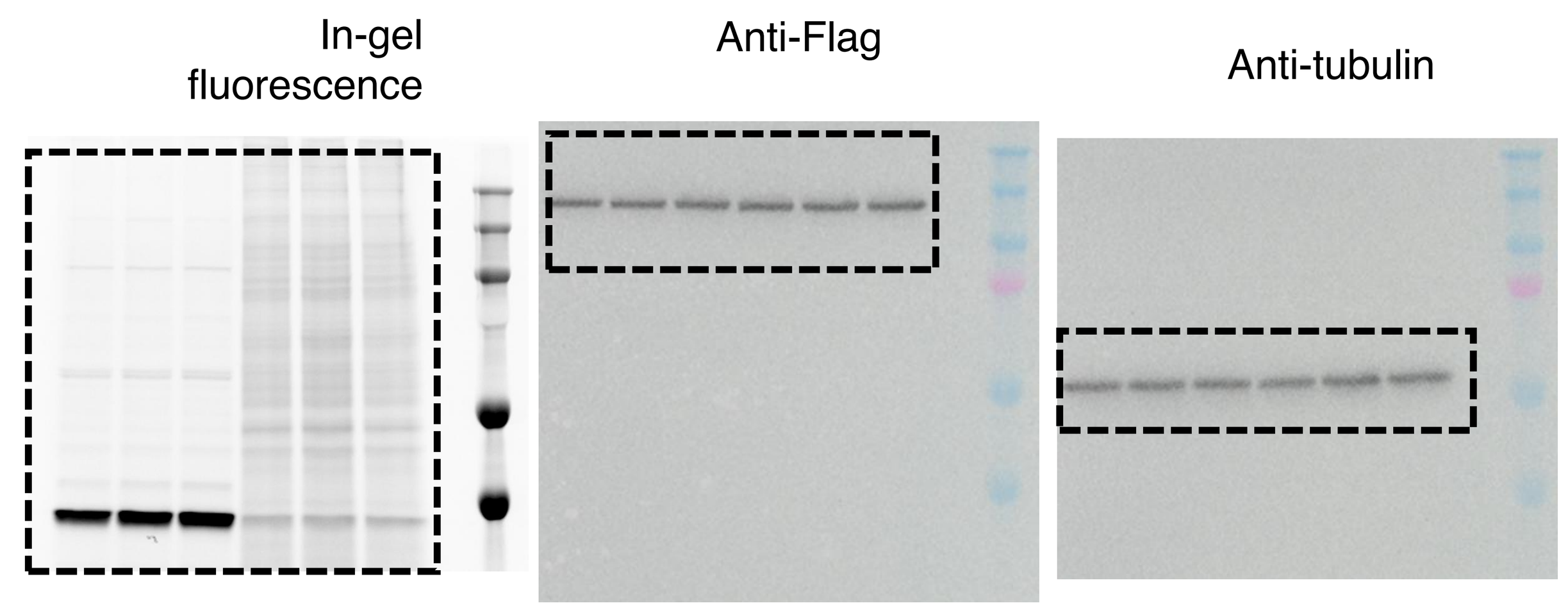

Figure 2C IPO5

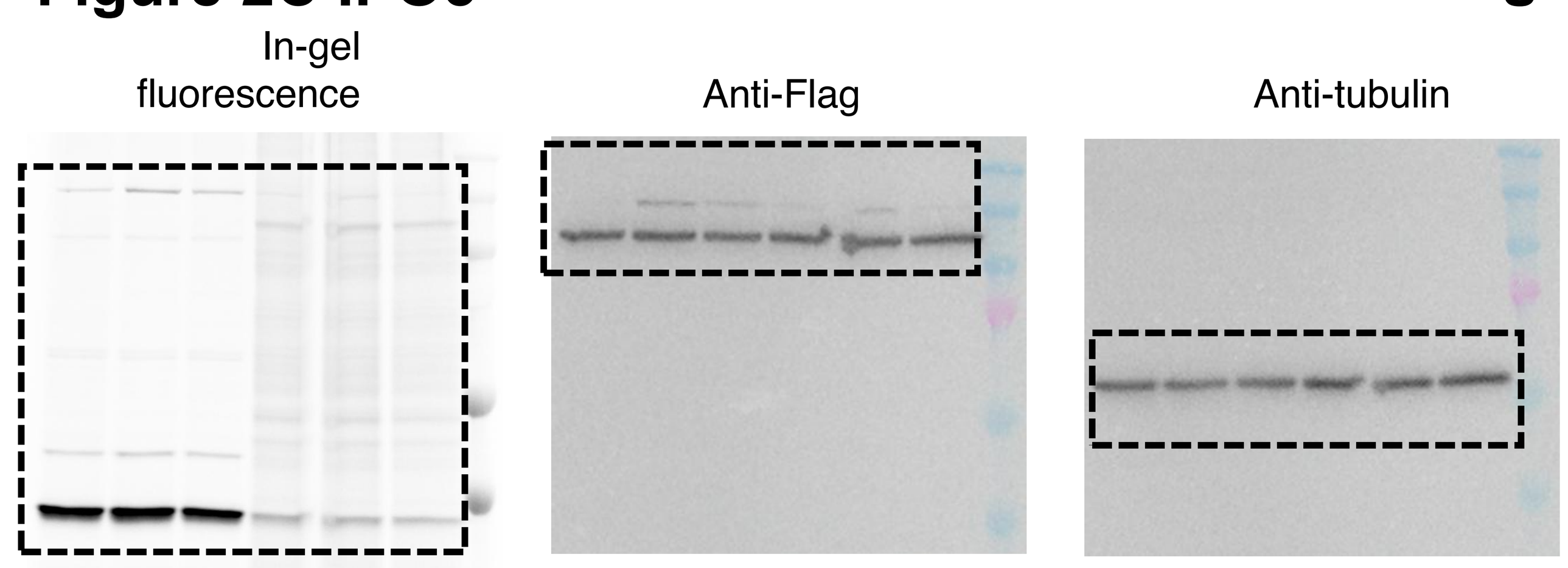

Figure 2F

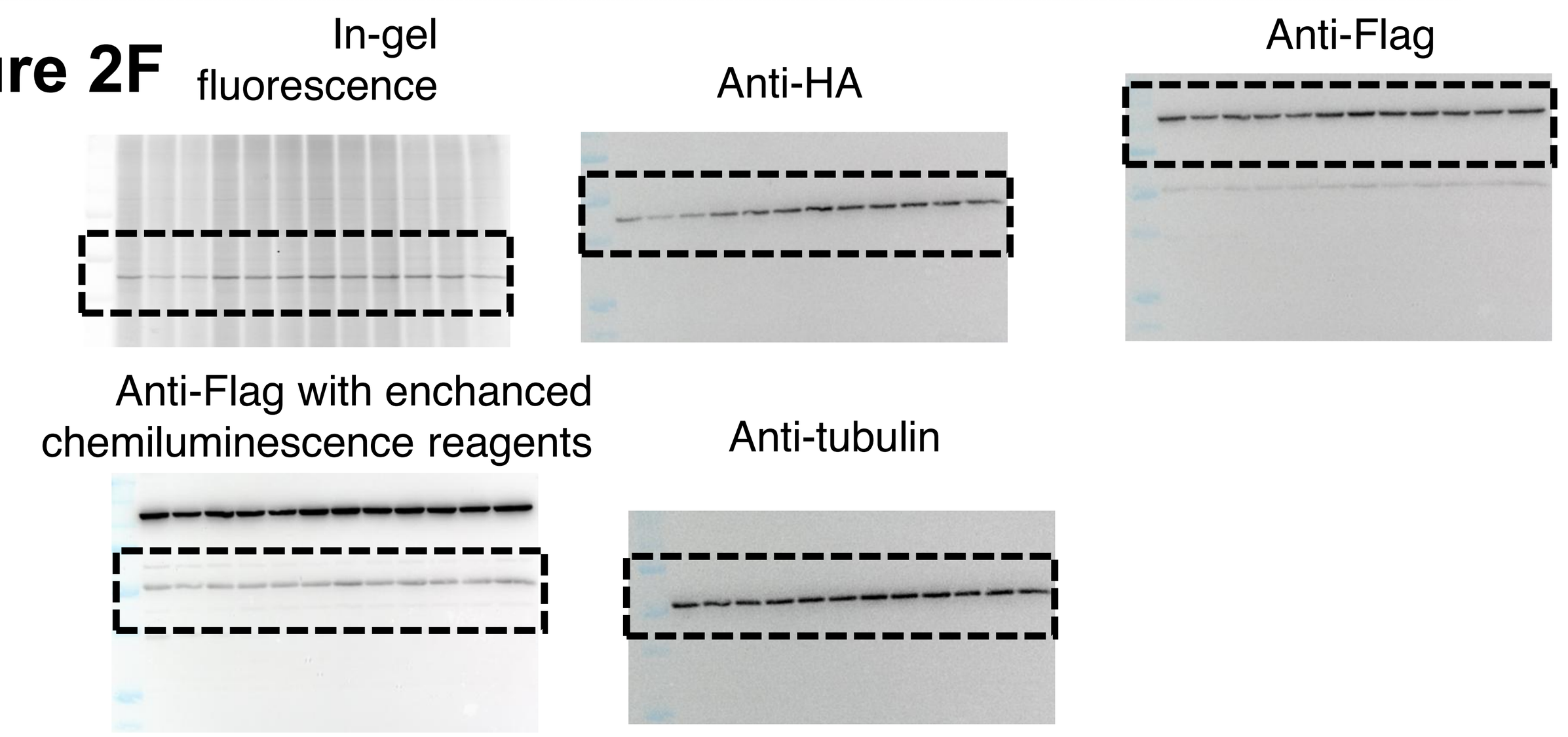

Figure 2G

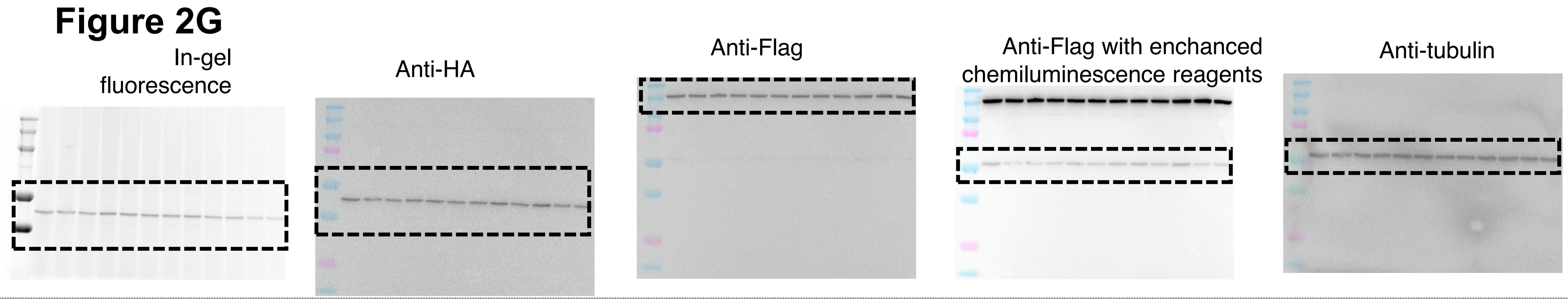

Figure 3B

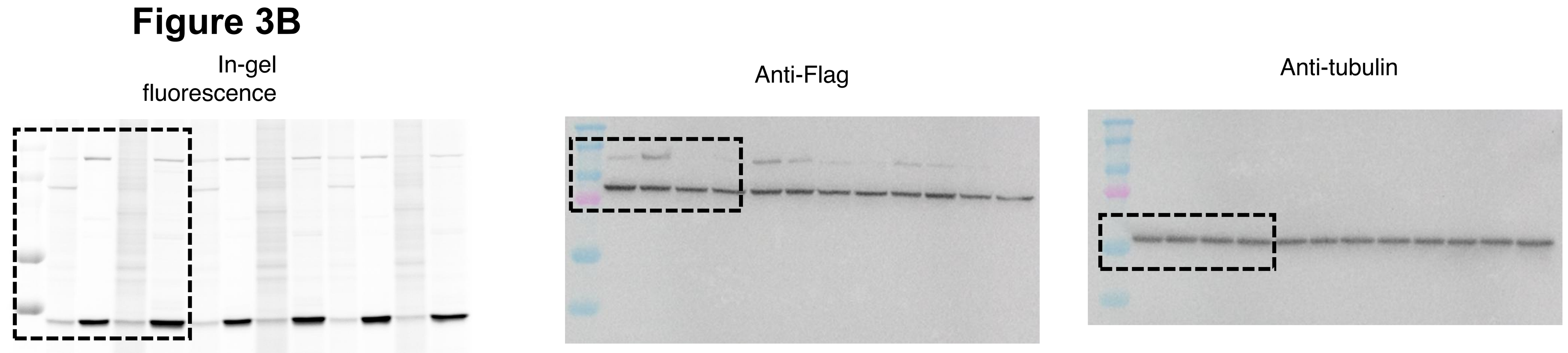

Figure 3C NCBP1 WT

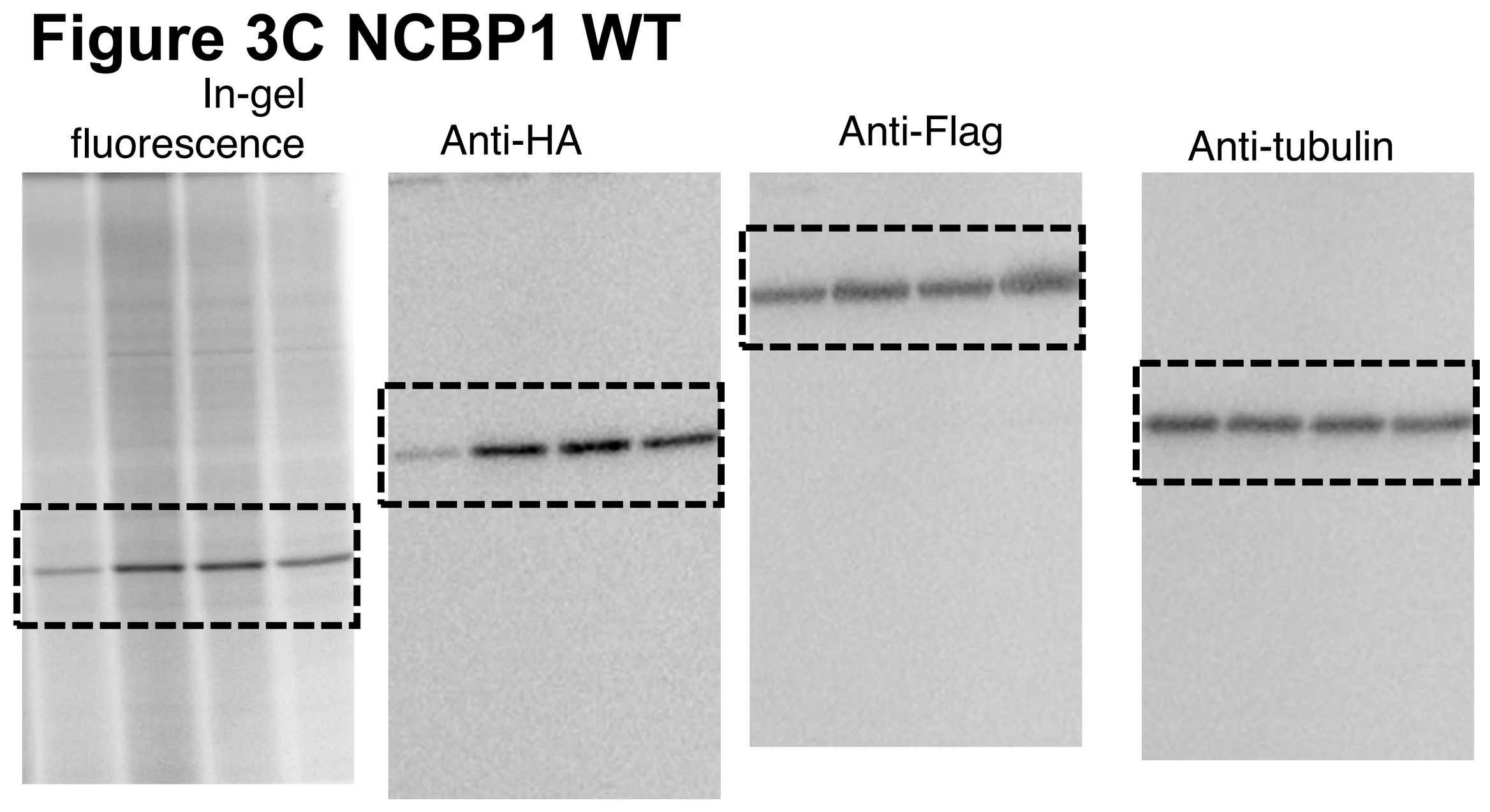

Figure 3C NCBP1 C436A

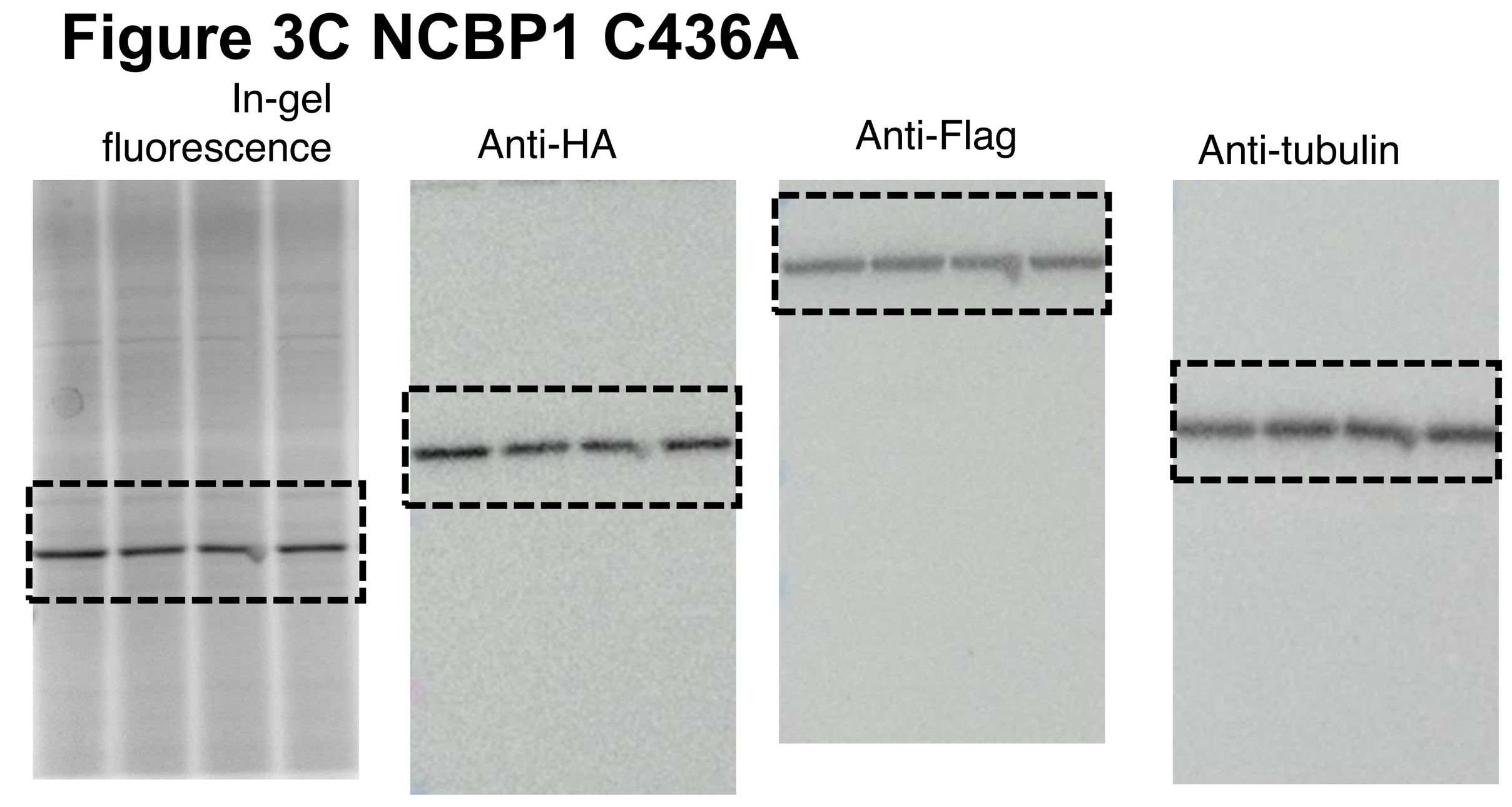

Figure 5E input

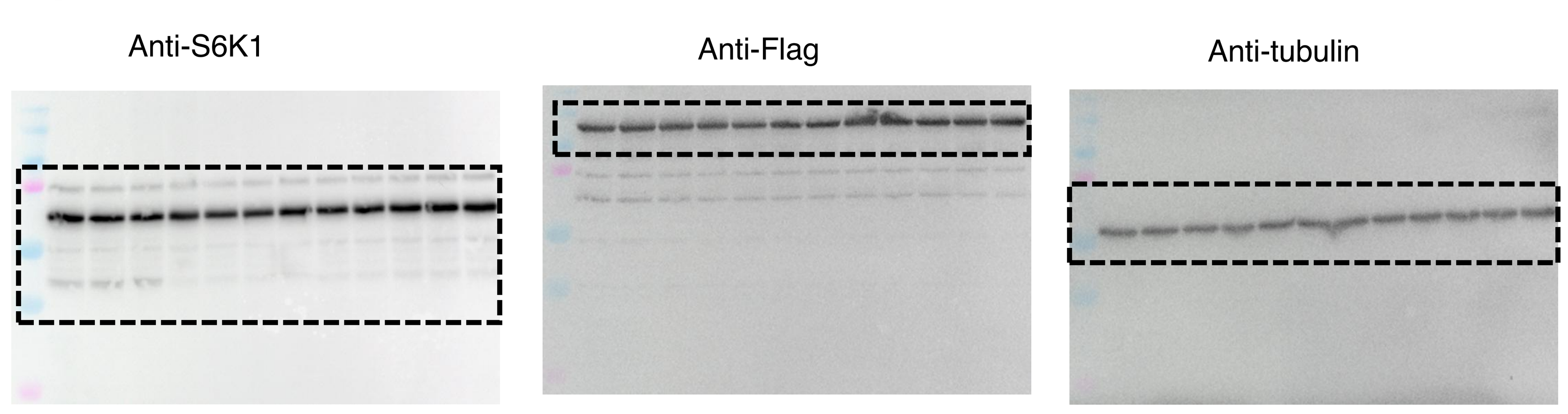

Figure 5E Flag IP

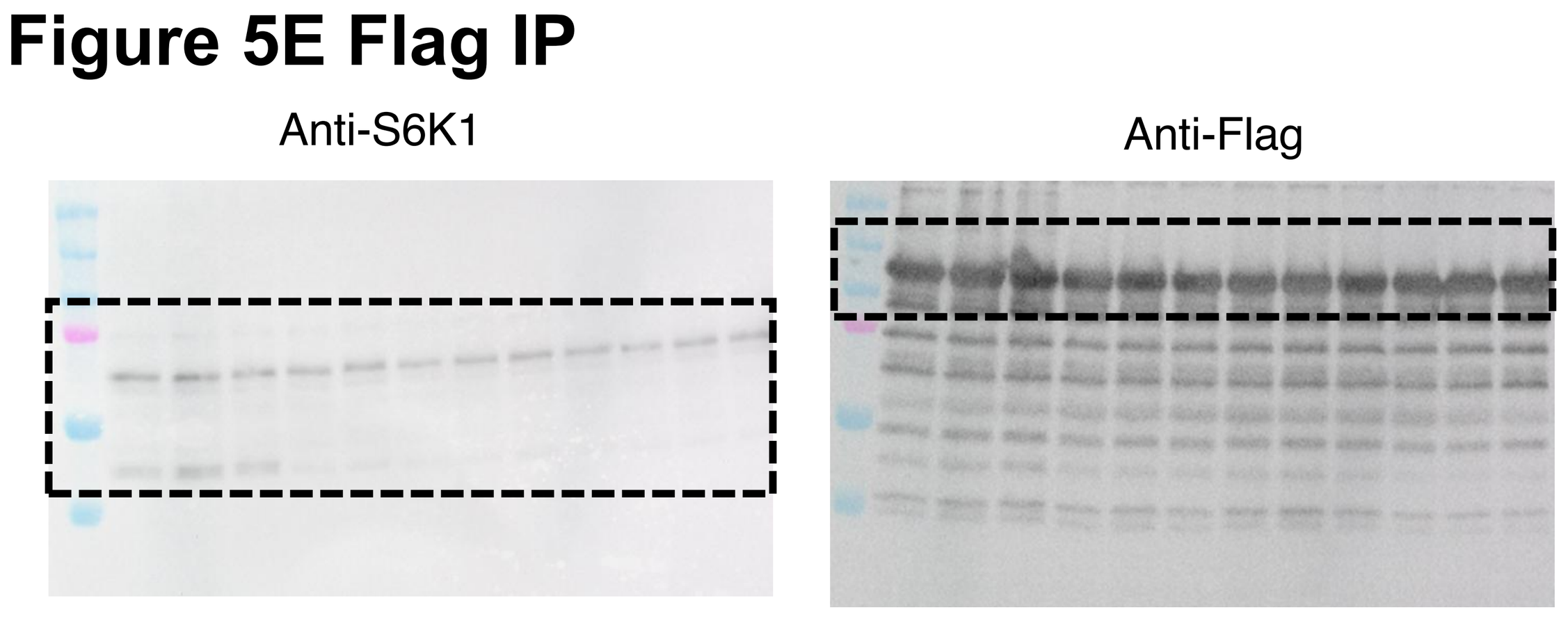

Figure 6A

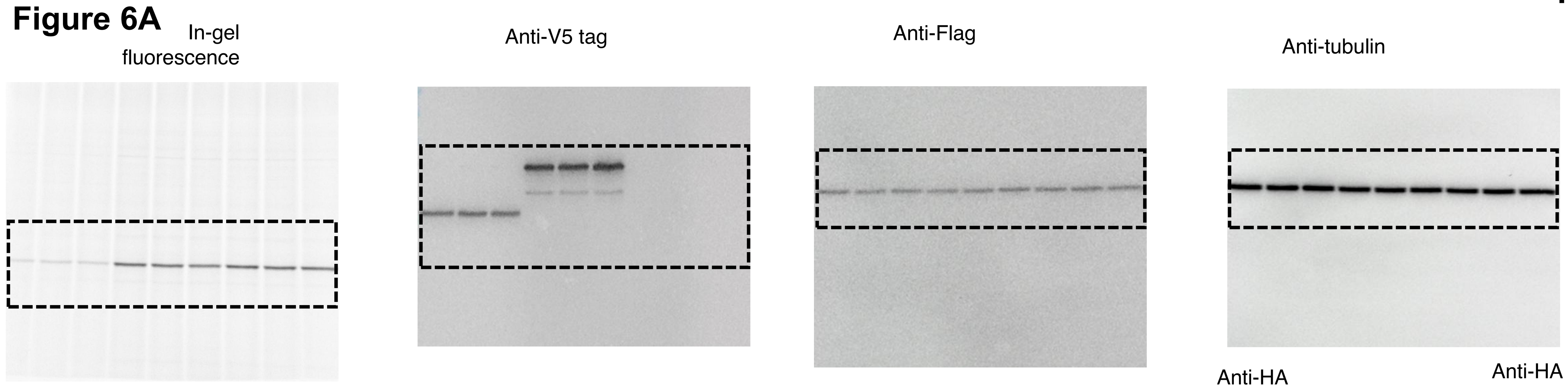

Figure 6C

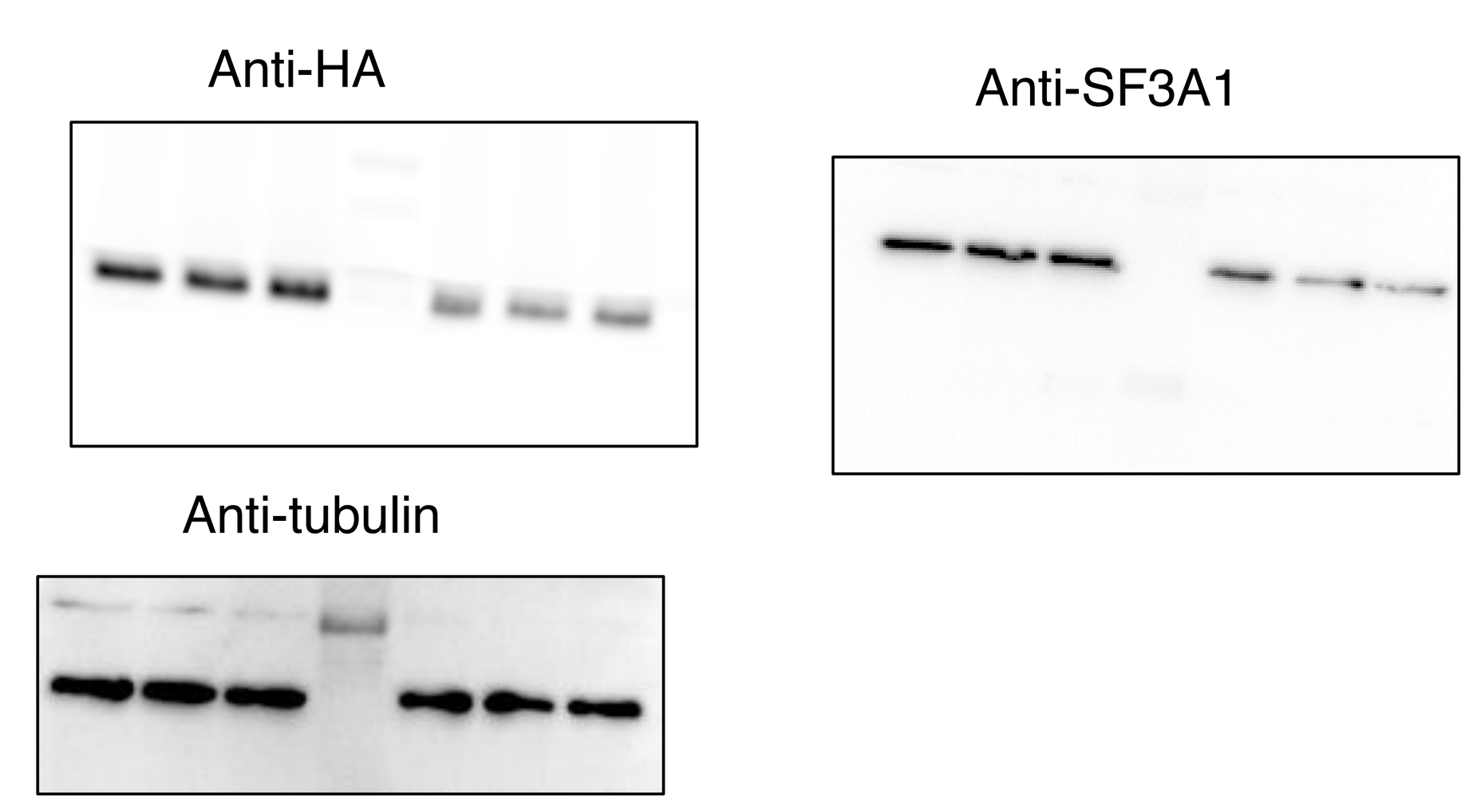

Figure 6B

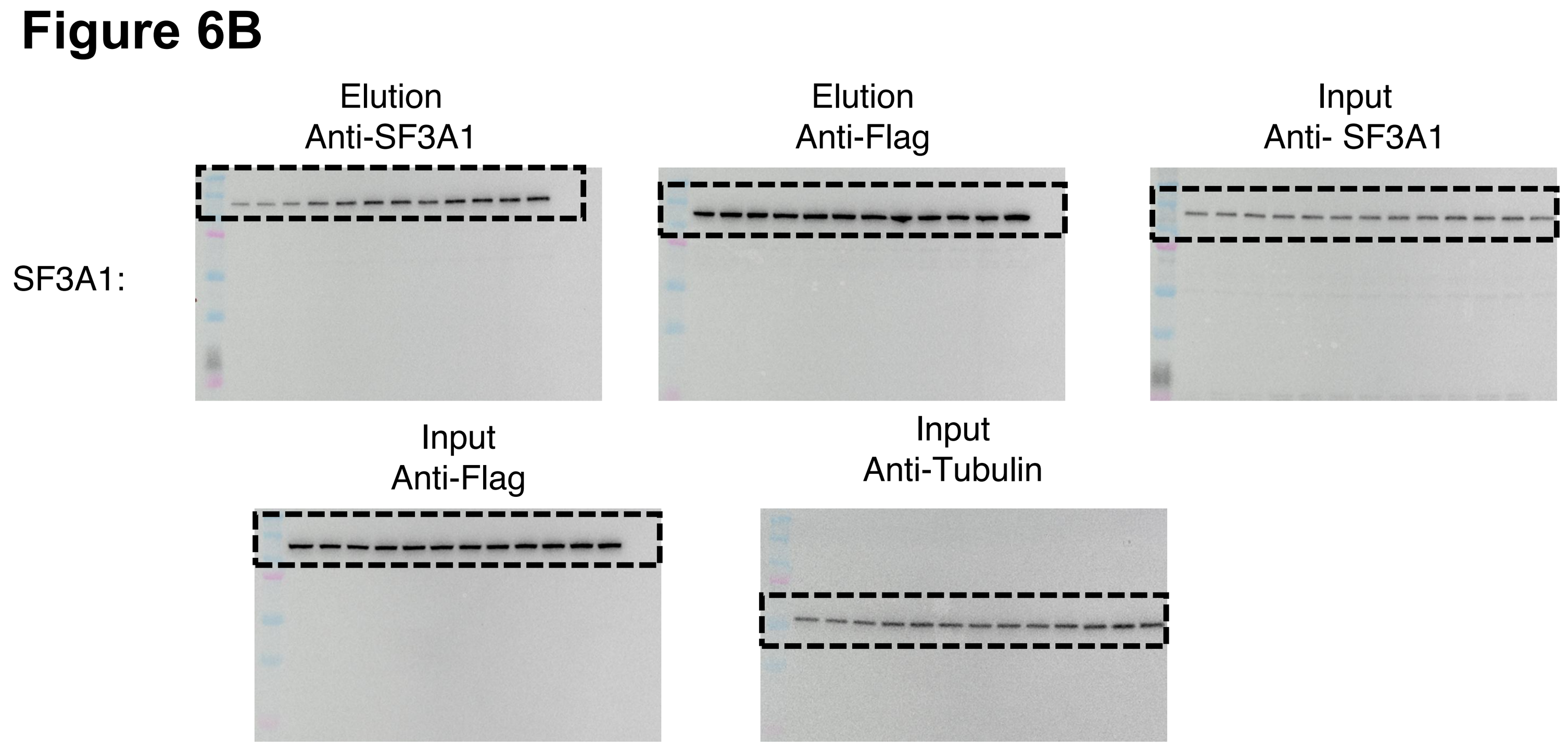

Figure 6C

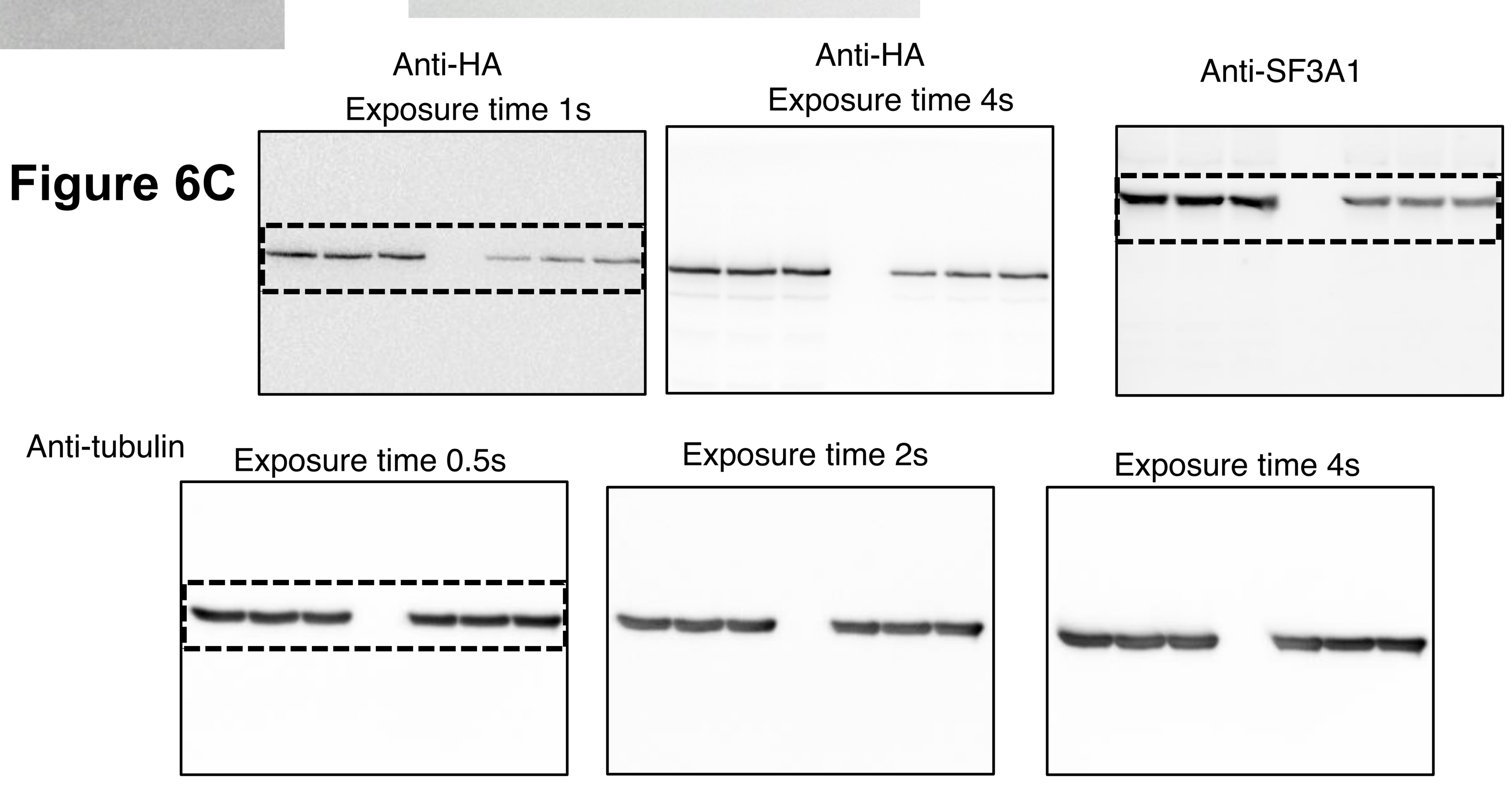

Supplement: Supplementary file 3 [file 41589_2025_2135_MOESM7_ESM.pdf]
