# Supplementary material for: NCBP1 stress signaling drives alternative S6K1 splicing inhibiting translation
Source: Nat Chem Biol. 2026 Feb 10;22(10):1628–41. doi: 10.1038/s41589-025-02135-4 (PMC13612227; doi:10.1038/s41589-025-02135-4)

Extended Data Fig. 2C

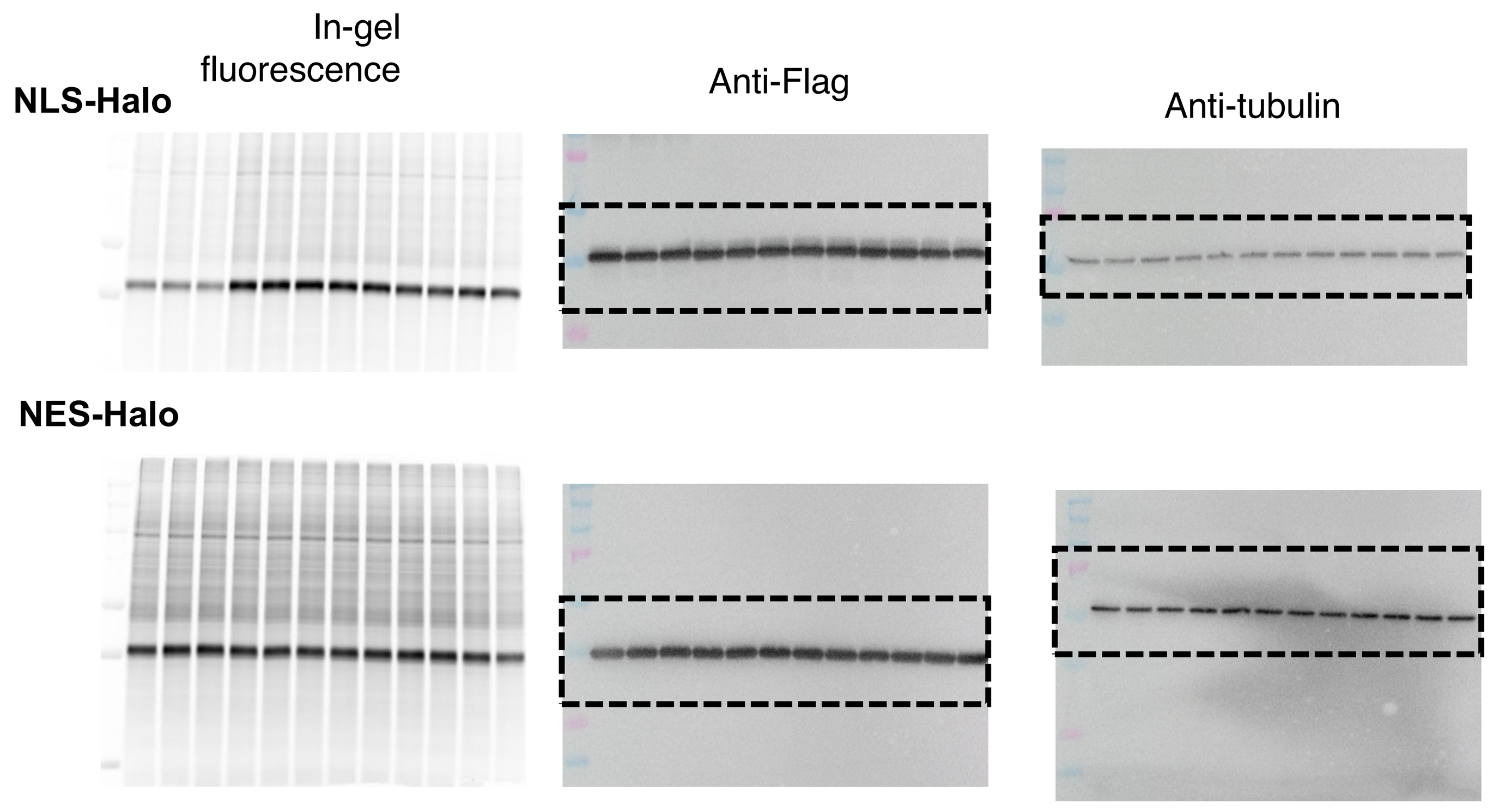

Extended Data Fig. 4B

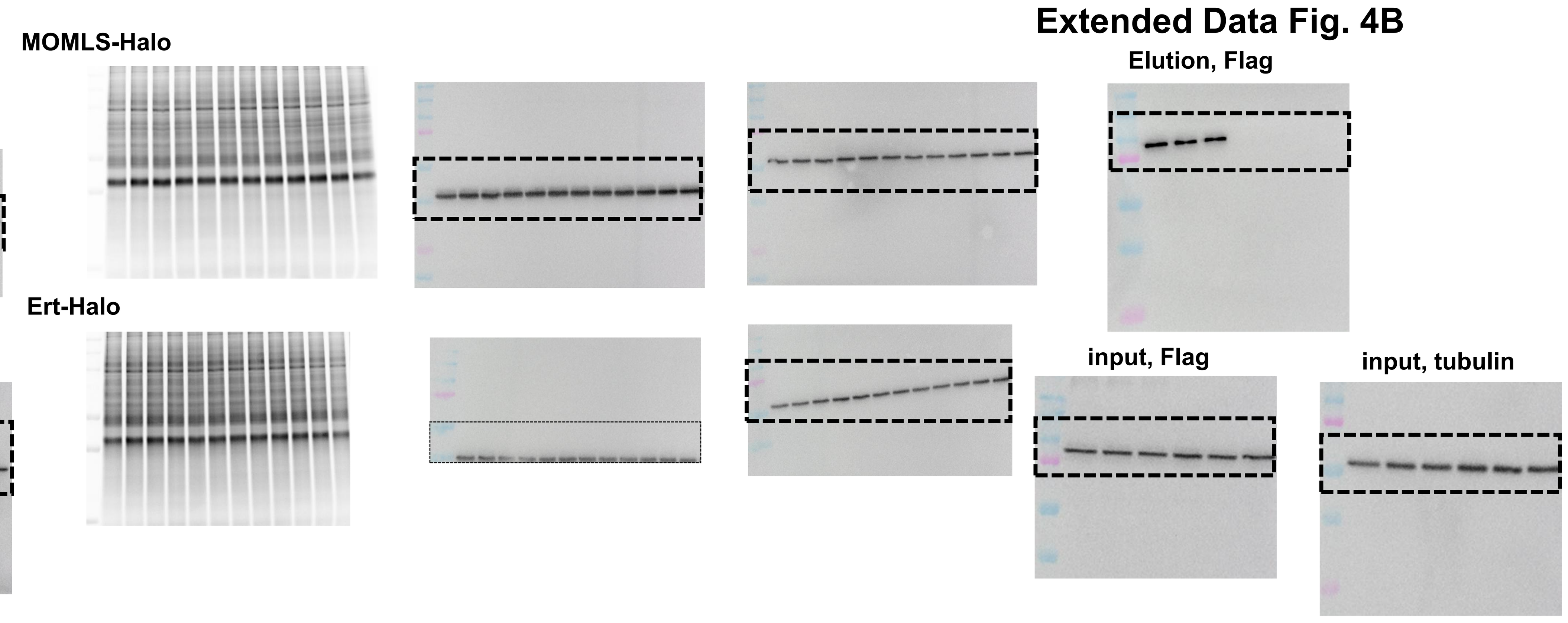

Extended Data Fig. 4C

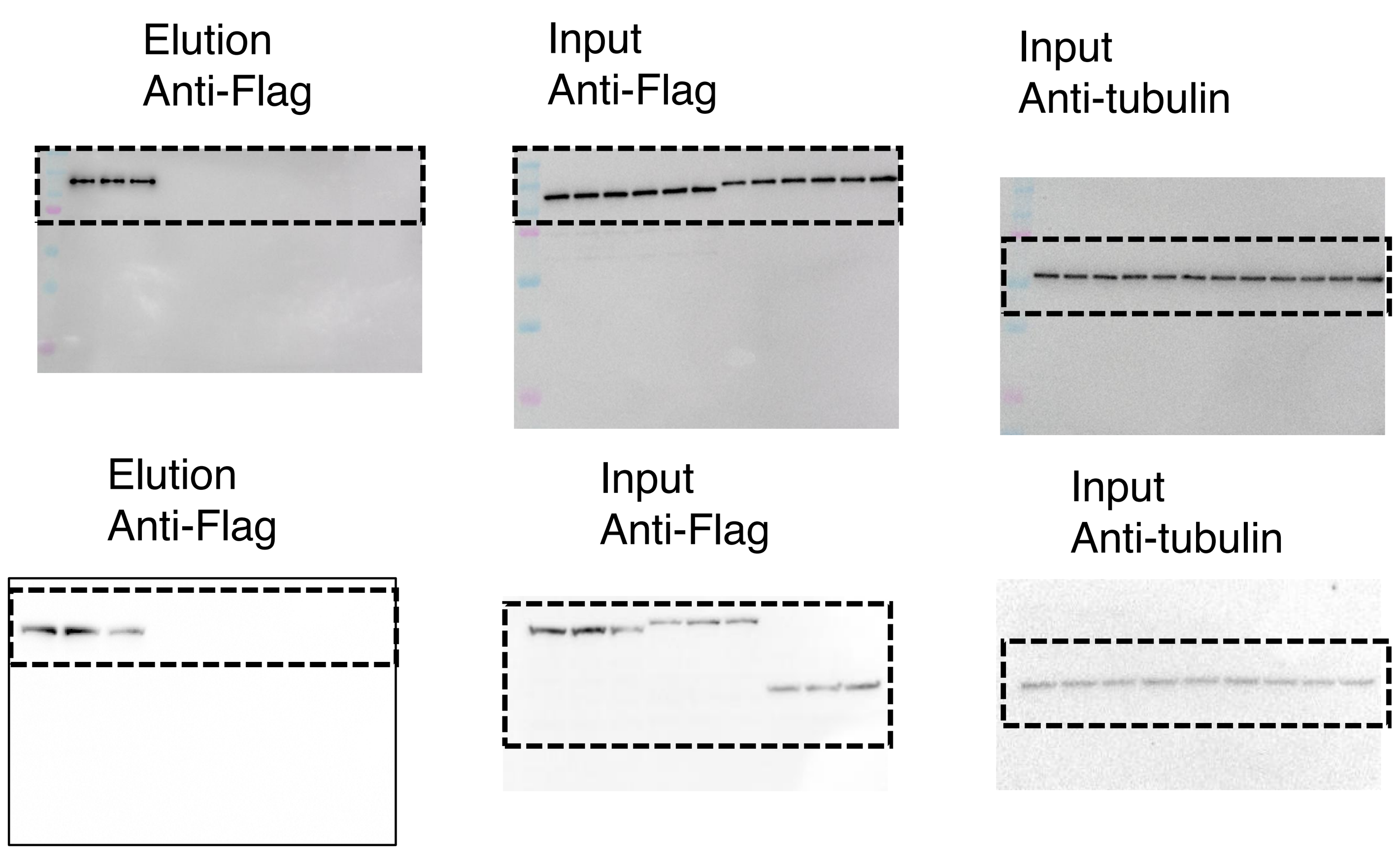

Extended Data Fig. 4D

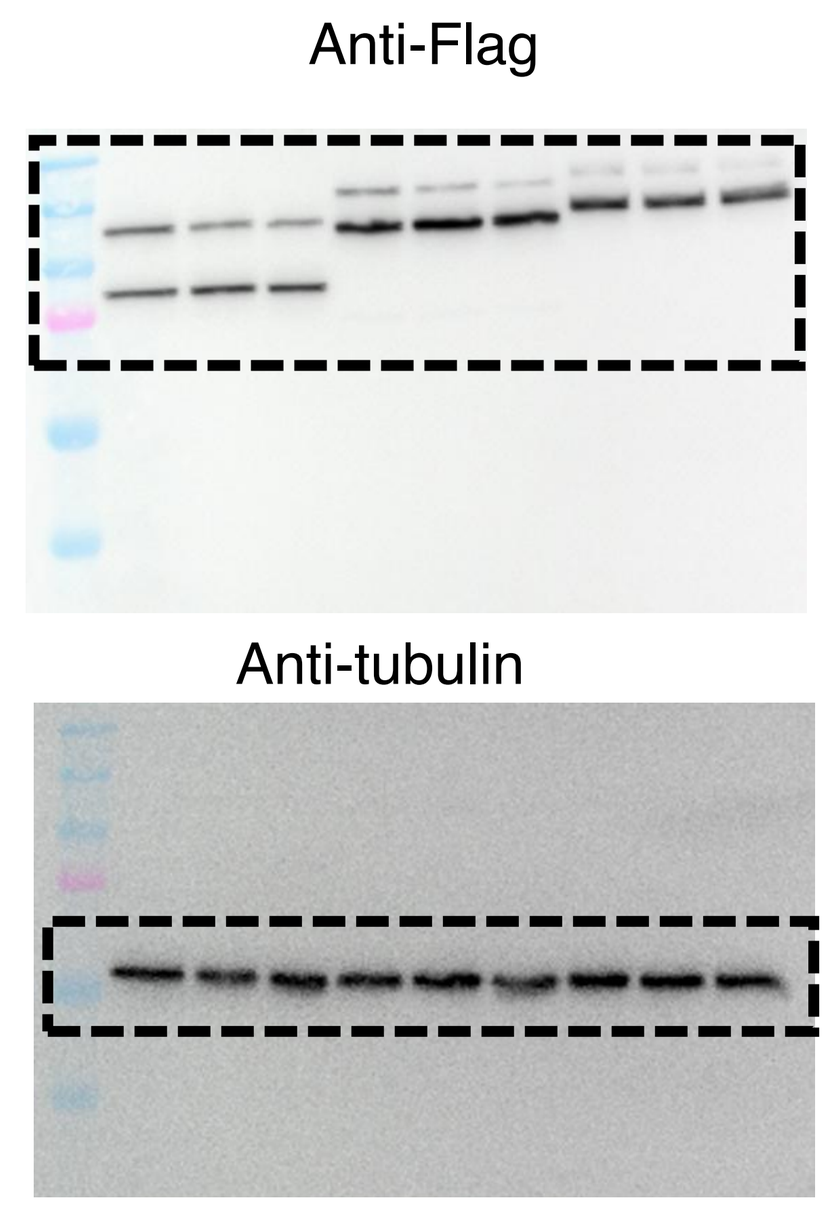

Extended Data Fig. 5B

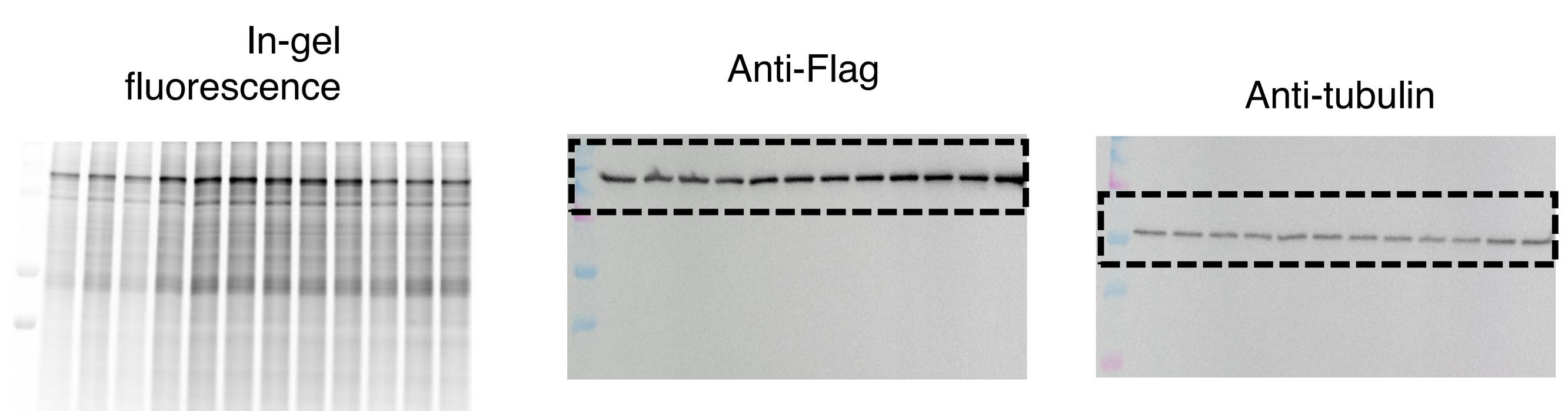

Extended Data Fig. 5F

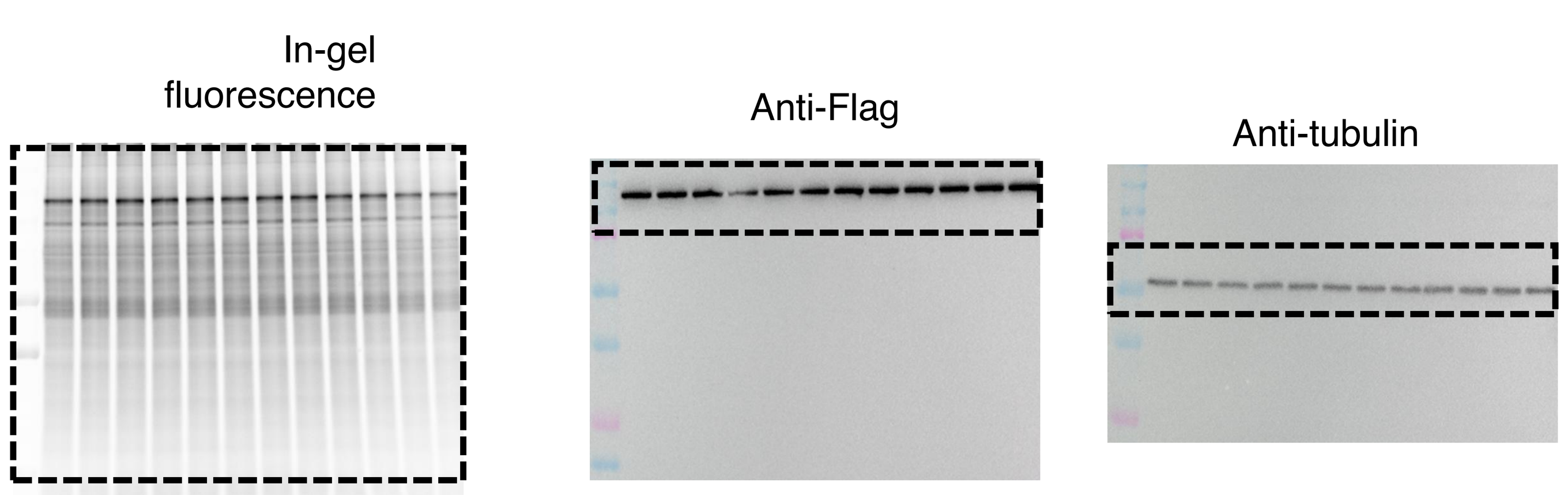

Extended Data Fig. 6C

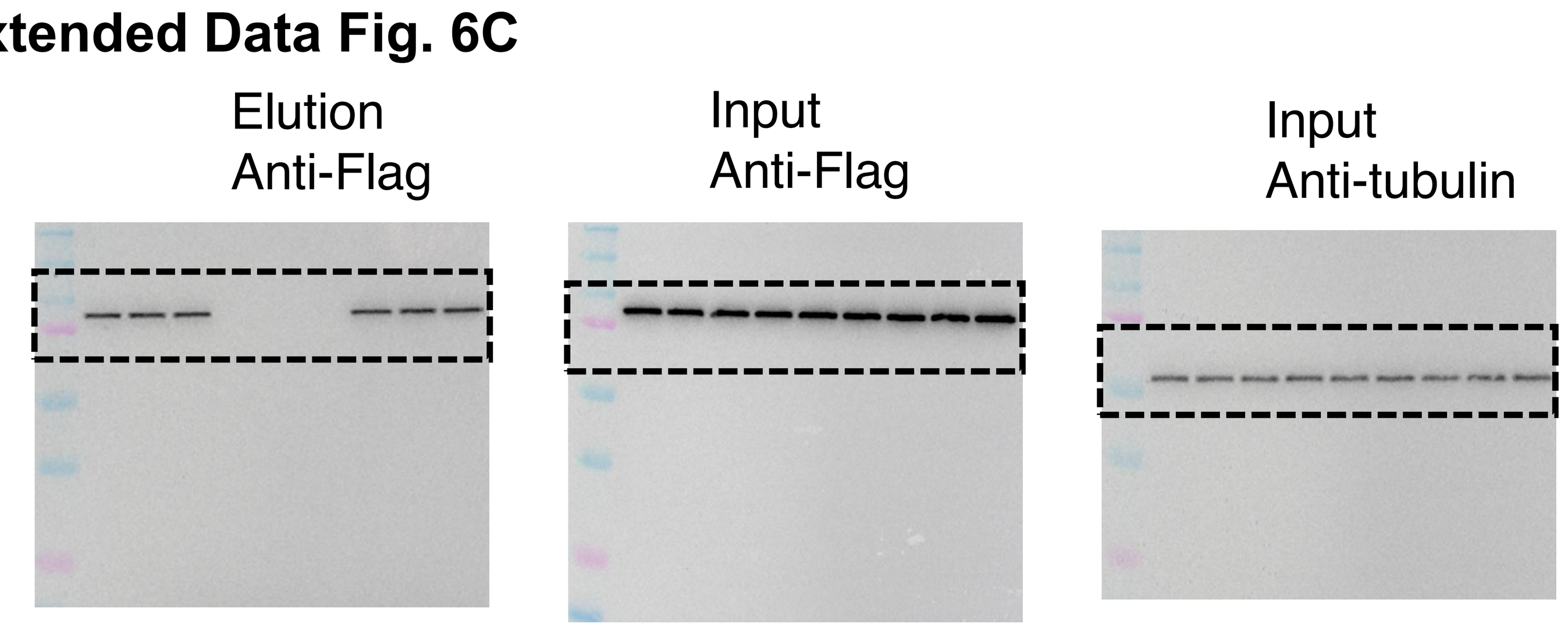

Extended Data Fig. 7

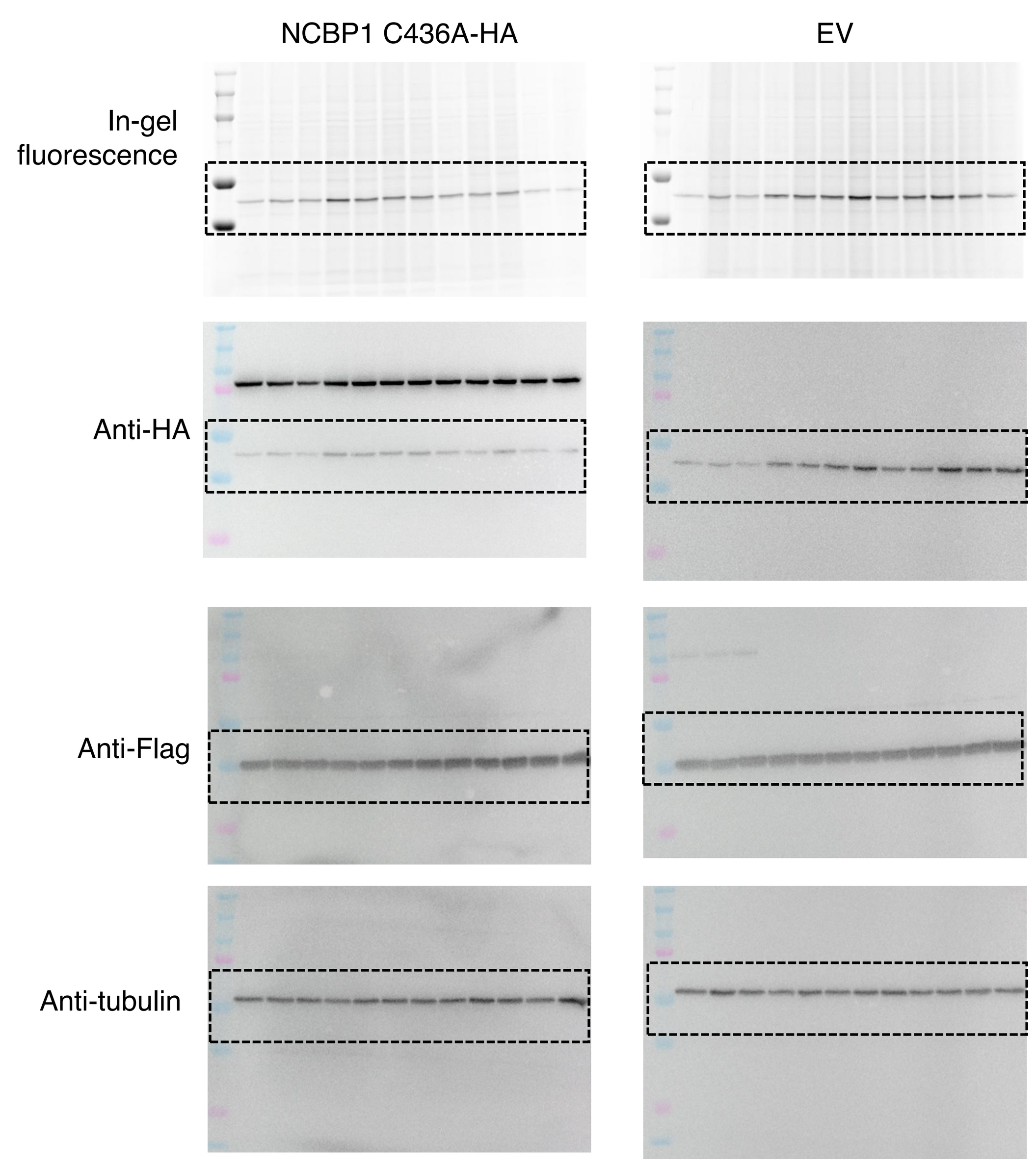

Extended Data Fig. 6D

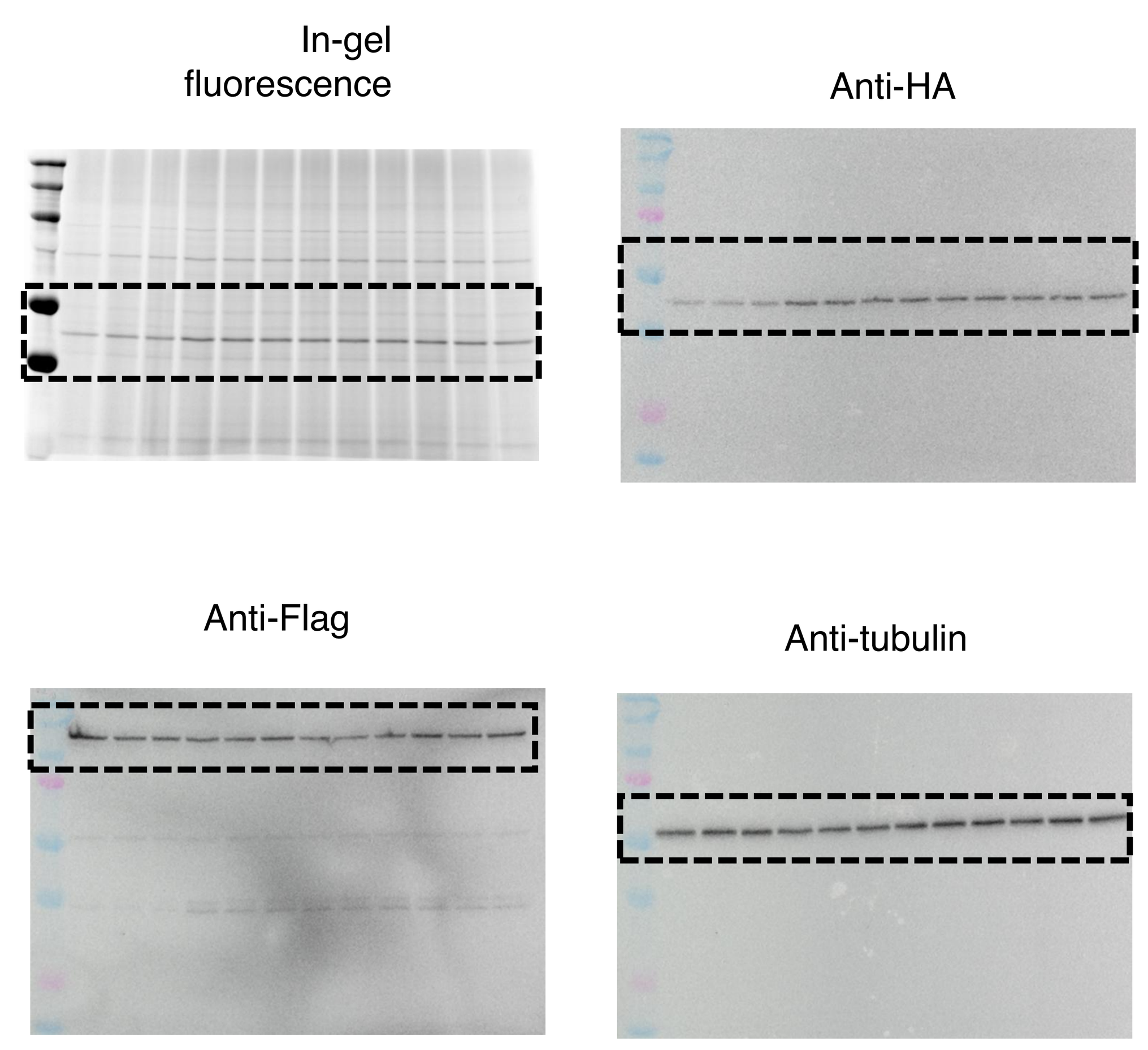

Extended Data Fig. 10B

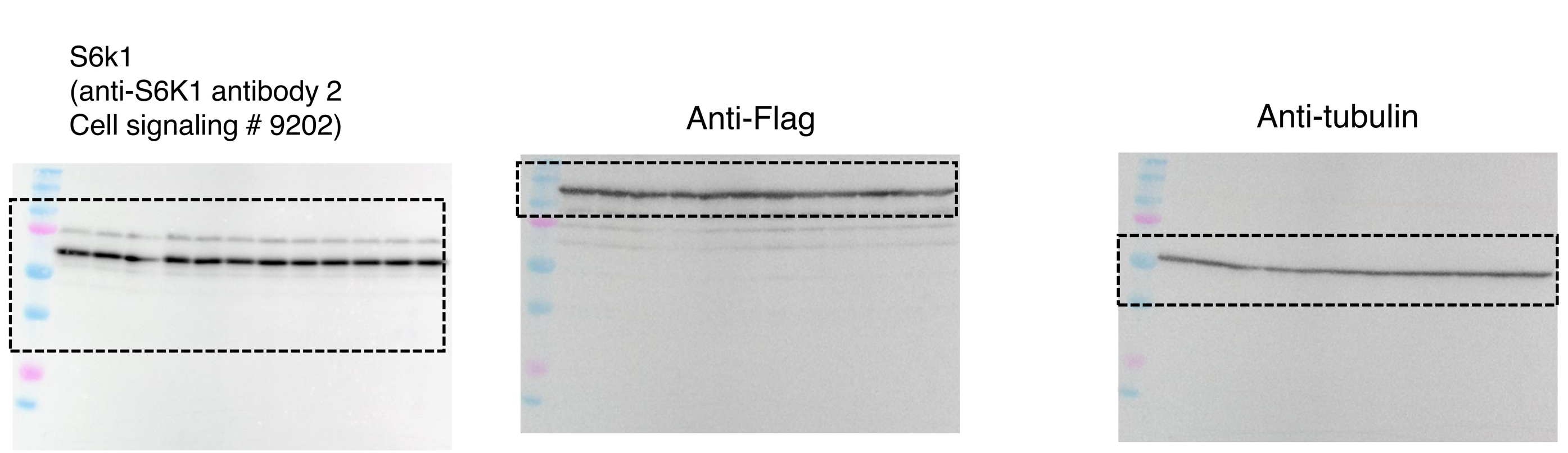

Extended Data Fig. 10E

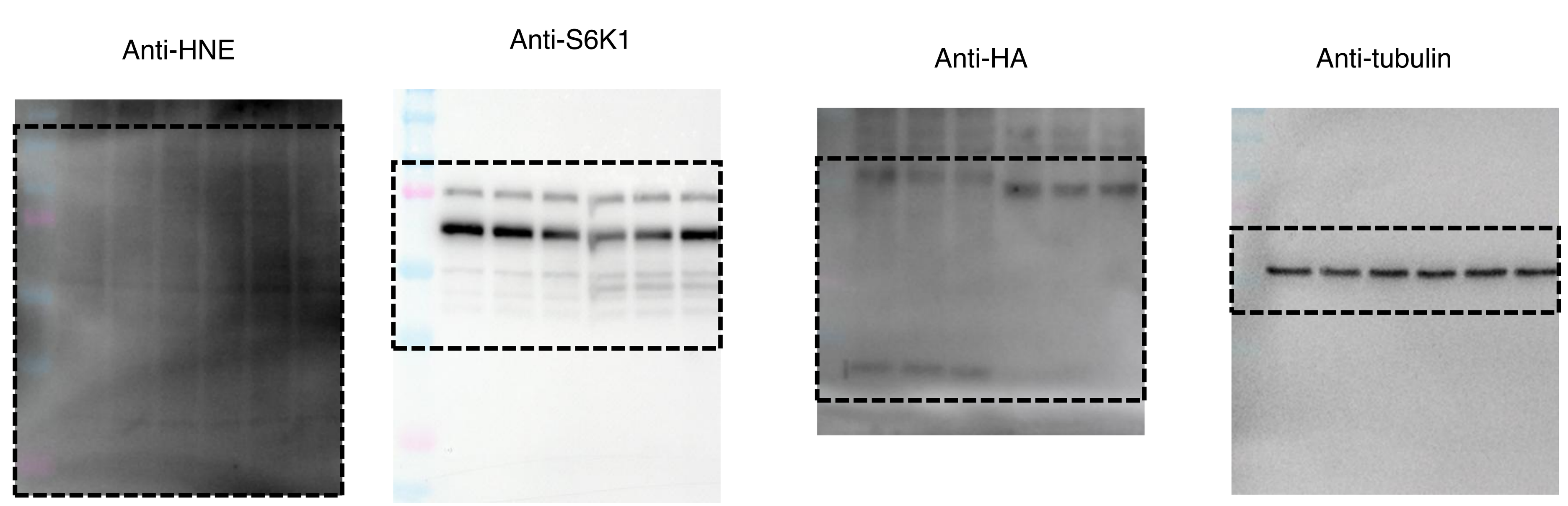

Supplement: Supplementary file 4 [file 41589_2025_2135_MOESM8_ESM.pdf]
